# Prevalence of antibody drug conjugated–induced nausea and vomiting (ADCINV) in patients with cancer

**DOI:** 10.1007/s00520-026-10674-2

**Published:** 2026-05-02

**Authors:** Ronald Chow, Daniel Zhang, Samy Kannout, Andreas Ma, Cindy Zheng, Ayden Blayne, Aaron Dou, Sumeet Talwar, Rouhi Fazelzad, Hope S. Rugo, Christina H. Ruhlmann, Hirotoshi Iihara, Mary Louise Affronti, Jennifer Leigh, Florian Scotté, Lawson Eng

**Affiliations:** 1 Princess Margaret Cancer Centre, University Health Network, Toronto, ON Canada; 2 Odette Cancer Centre, Sunnybrook Health Sciences Centre, Toronto, ON Canada; 3 Temerty Faculty of Medicine, University of Toronto, Toronto, ON Canada; 4 Centre for Evidence-Based Medicine, University of Oxford, Oxford, UK; 5 Siebel School of Computing and Data Science, University of Illinois Urbana-Champaign, Champaign, IL USA; 6 Department of Medical Oncology, City of Hope Comprehensive Cancer Center, Duarte, CA USA; 7 Department of Oncology, Odense University Hospital, Odense, Denmark; 8 Department of Pharmacy, Gifu University Hospital, 1-1 Yanagido, Gifu, Gifu, 501-1194 Japan; 9 Cancer Center, Gifu University Hospital, 1-1 Yanagido, Gifu, Gifu, 501-1194 Japan; 10 Duke University School of Nursing, Duke University, Durham, NC USA; 11 Département Interdisciplinaire D’Organisation Des Parcours Patients, Gustave-Roussy, Université Paris-Saclay, 94800 Villejuif, France

**Keywords:** Antibody drug conjugate, Nausea, Vomiting

## Abstract

**Introduction:**

Antibody–drug conjugates (ADC) have emerged as an important part of systemic treatment across disease sites. To date, there is no robust pooled prevalence estimate of nausea and vomiting induced by ADCs. Establishing such estimates is essential to determine if ADC-induced nausea and vomiting (ADCINV) represents a clinically significant problem warranting further research into antiemetic prophylaxis. Our aim is to report the prevalence of reported ADCINV across literature.

**Methods:**

A systematic search of Medline, Embase, Cochrane CENTRAL and Web of Science was conducted from database inception until September 24, 2025. Articles were included if they reported nausea and/or vomiting due to ADCs used in cancer treatment, in the abstract. Pooled prevalence was reported. Subgroup analysis was conducted by ADC. Meta-regression was conducted by age and sex. Quality assessment was conducted. Type I error was set at 0.05.

**Results:**

A total of 209 studies with 15,493 patients were included. Thirty-nine percent (95%CI, 36–42%) of patients experience any nausea, and 26% (95%CI, 23–29%) experience any vomiting. Younger patients are more likely to experience nausea; each 10-year increase in age is associated with a 12% decrease in nausea rates. Higher emetogenic ADCs include trastuzumab deruxtecan, sacituzumab govitecan, brentuximabvedotin and patritumab deruxtecan. Lower emetogenic agents include disitimab vedotin, telisotuzumab vedotin and rovalpituzumab tesirine.

**Discussion:**

This is the first study to report prevalence of ADCINV across ADCs. It is a prevalent adverse effect akin to chemotherapy-induced nausea and vomiting that should be viewed as clinically relevant and further investigated to help develop optimal strategies related to antiemetics.

**Supplementary Information:**

The online version contains supplementary material available at 10.1007/s00520-026-10674-2.

## Introduction

Nausea and vomiting are among the most distressing adverse effects of systemic therapy, with the ability to impact quality of life and even cause treatment non-adherence [1]. Nausea and vomiting are conventionally reported in the literature in the overall phase (0 to 120 h), and also three phases, after initiation of treatment: (i) acute phase, occurring in the first 24 h; (ii) delayed phase, occurring between 24 and 120 h; and (iii) long delayed phase, occurring after 120 h [2].

Chemotherapy-induced nausea and vomiting (CINV) has been extensively researched in the past decades. Different chemotherapies have varying emetogenic profiles; highly emetogenic agents (HEC) include cisplatin and anthracycline/cyclophosphamide combination (emesis risk > 90% of patients), and moderately emetogenic agents (MEC) include doxorubicin and carboplatin (emesis risk 30–90%) [3]. Despite guidelines recommending emetic prophylaxis, patients still experience CINV. Among patients receiving HEC, one-quarter (24%) experience CINV in the acute phase, half (49%) in the delayed phase and one-third (31%) in the long-delayed phase [2]. Among patients receiving MEC, less than one-tenth (7%) experience CINV in the acute phase, over one-third (38%) in the delayed phase and one-quarter (24%) in the long-delayed phase [2]. As a result, decades of research have focused on developing and testing antiemetic agents for prophylactic use [4].

In recent years, antibody–drug conjugates (ADC) have emerged as an important innovation in systemic cancer treatment [5]. By combining a monoclonal antibody with a cytotoxic payload, ADCs allow for more targeted delivery of chemotherapy, improving efficacy while potentially limiting systemic toxicity [6]. The hematologic and metabolic safety profile of ADCs are increasingly well described, but the burden of nausea and vomiting is not well characterized [7]. To date, there is no robust pooled prevalence estimate of ADC-induced nausea and vomiting (ADCINV).

The aim of this study is to systematically summarize all published primary research on ADC in cancer treatment, to summarize the prevalence of ADCINV.

## Methods

This was a systematic review, prospectively registered on PROSPERO (CRD420251156227).

### Outcomes

The primary outcomes were the prevalence of ADC-induced nausea, and prevalence of ADC-induced vomiting, among patients treated with ADCs. The secondary outcomes were the prevalence of severe nausea and prevalence of severe vomiting, defined as Grade ≥ 3 on the CTCAE grading tool [8].

### Search strategy

Comprehensive searches were developed with an information specialist (RF), and conducted in Medline, Embase, the Cochrane Central Register of Controlled Trials (CENTRAL) and Web of Science from inception to September 24, 2025. Search strategies combined controlled vocabulary and free-text terms for the concepts of (i) nausea and vomiting, and (ii) antibody drug conjugates approved for the treatment of cancer, including both trade and generic names of individual drugs. ADCs approved for the treatment of cancer were noted from the US National Institutes of Health’s online database, accessed on August 20, 2025 [9]. Searches were restricted to human studies. Full search strategies are available in the Appendix.

### Eligibility criteria

All search results underwent a two-stage screening process. At level 1 (title and abstract) screening, studies were eligible for further screening if they reported on the use of an ADC for the treatment of cancer and provided data on nausea and/or vomiting. At level 2 (full text) screening, studies were included if they were primary research articles; review articles, commentaries and editorials were excluded. When multiple publications reported on the same underlying study, the publication with the largest number of patients was included. Pooled analysis were excluded, if the individual studies were available for inclusion.

Screening was performed in duplicate and independently. One reviewer (DZ) developed a software environment in which search results were processed using the large language model (LLM) of ChatGPT-5, as per the specified eligibility criteria. ChatGPT-5 was provided the abstract (in level 1) and the full text article (in level 2), with a system prompt that asked it to extract specific data fields that were relevant to this study. The LLM returned extracted data in JSON format, and a corresponding reasoning summary and confidence score (low, medium, high). Similar methodology has been previously used, with high degree of sensitivity and specificity on-par to human reviewers [10]. Independent human screening (RC) was conducted in parallel. Discrepancies between LLM-assisted and human screening decisions were reviewed jointly. The human reviewer examined the model’s reasoning output, and, where necessary, ChatGPT was further queried to clarify its decision logic. Disagreements were resolved through discussion between human and ChatGPT, to achieve consensus. If consensus could not be achieved, a third reviewer (LE) assisted in discussions and achieving consensus.

### Data extraction

For each included study, we noted study design, patient demographics (age, sex, cancer type), investigated ADC, use of antiemetic prophylaxis and our pre-specified primary and secondary outcomes. The primary and secondary outcomes were extracted for the overall study period and not according to the antiemetic endpoints of acute, delayed, long-delayed or overall phases, as this was not available in the underlying studies. Data extraction was likewise performed in duplicate and independently (DZ, RC), and disagreements resolved through consensus, with involvement of a third reviewer (LE) as needed.

### Quality assessment

Studies were evaluated for quality based on study design. Observational studies were evaluated using the Cochrane Risk of Bias in Non-randomized Studies of Exposures (ROBINS-E) [11], while interventional studies were evaluated using either the Risk of Bias in Non-randomized Studies of Interventions (ROBINS-I) [12] or the Cochrane Risk of Bias tool version 2 (RoB 2) [13]. Quality assessment was visually presented using robvis [14].

### Data synthesis

A descriptive synthesis was conducted of study and patient characteristics. Forest plots of study-specific prevalence estimates were generated. A pooled prevalence and corresponding 95% confidence interval (95%CI) was calculated per outcome, weighted based on study sample size and using a random effects model. Leave-one-out analysis was conducted to determine if there were any studies with significant weight to the pooled prevalence rate. Subgroup analyses were conducted by ADC, if more than one study reported on the outcome. Meta-regression was conducted by the study’s mean age, percentage of participants reported as female, number of patients in study and median follow-up duration of study. Type I error was set at 0.05. All analyses were conducted using Stata18.0 BE.

## Results

**Fig. 1:**
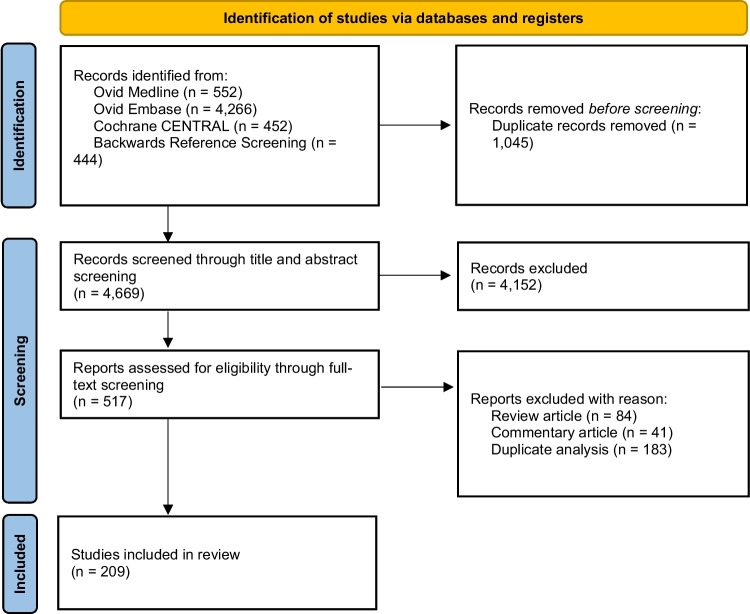
PRISMA flow diagram

A total 5714 references were identified through database search. After removal of duplicates, 4669 references underwent level 1 screening. A total of 517 full text articles underwent level 2 screening. Ultimately, 209 [15–222] were included in this review (Fig. 1).

Table 1 reports individual study characteristics. Eighty-four (41%) were international trials. Among single-country trials, 62 (30%) articles originated from the USA, 35 (17%) from China, 5 (2%) from Japan and 2 (1%) each from the UK, Italy and Australia. Among the 152 that reported a primary cancer site, the most studied cancer site group setting was lymphoma and myeloma (*n* = 55, 36%), followed by breast (*n* = 30, 20%), lung (*n* = 17, 11%), genitourinary (*n* = 17, 11%) and gastrointestinal (*n* = 16, 11%). Across all studies, there were a total 15,493 patients. The mean number of patients per trial was 74 (standard deviation 99). The mean percentage of patients identified as female per trial was 53% (standard deviation 27%). Over two-thirds of studies (*n* = 145, 69%) enrolled patients who had previously received systemic therapy. One-tenth (*n* = 22, 11%) of studies administered ADCs alongside other systemic therapy. Only five studies explicitly reported using antiemetic prophylactic regimens—two reported using 5-HT_3_ receptor antagonists, and three reported using steroids. Average follow-up (*n* = 51 studies) was a median of 11.4 months.

**Table 1:** Study characteristics

Study	Study type	Country	Drug (generic)	Cancer type and stage	Trial ID	*n*	% female	Age	Antiemetic prophylaxis	Prior lines of therapy
[16] (Medicina)	Observational study	Turkey	Gemtuzumab ozogamicin	Acute myeloid leukemia, relapsed or refractory	NR	24	33.3	Mean: 47.63 (± 16.93)	NR	Systemic therapy
[17] (J Clin Oncol)	Non-randomized interventional	USA	Ozuriftamab vedotin	Head and neck, recurrent or metastatic squamous cell carcinoma	NCT03504488	31	NR	NR	NR	Systemic therapy
[18] (J Clin Oncol)	Non-randomized interventional	International	Inotuzumab ozogamicin	Lymphoma and myeloma, relapsed or refractory CD22 + B cell non-Hodgkin’s lymphoma	NR	79	41	Median: 60	NR	NR
[19] (Ann Oncol)	Non-randomized interventional	USA	Rovalpituzumab tesirine	DLL3-expressing advanced solid tumors, relapsed/refractory	NCT02709889	31	NR	NR	NR	Systemic therapy
[20] (Clin Cancer Res)	Non-randomized interventional	International	Tak-264	Gastrointestinal malignancies, advanced/metastatic	NCT01577758	41	34	Median: 60	NR	NR
[22] (Invest New Drugs)	Non-randomized interventional	International	Tak-264	Pancreatic adenocarcinoma, advanced/metastatic	NCT02202785	43	53	Median: 61	NR	Systemic therapy
[21] (Invest New Drugs)	Non-randomized interventional	International	Tak-264	Gastrointestinal, metastatic or recurrent adenocarcinoma of the stomach or gastroesophageal junction	NCT02202759	38	18	Median: 63	NR	Systemic therapy
[23] (J Clin Oncol)	Non-randomized interventional	China	LM-302	Gastrointestinal, advanced gastric/gastroesophageal junction cancer	NCT05161390	135	NR	NR	NR	Systemic therapy
[24] (Blood)	Non-randomized interventional	USA	Brentuximab vedotin	Systemic mastocytosis, advanced	NR	10	60	Median: 72.5	NR	NR
[25] (ESMO Open)	Non-randomized interventional	International	Puxitatug samrotecan	Breast, advanced/recurrent HR +/HER2 −	NCT05123482	57	NR	Median: 57	NR	Systemic therapy
[26] (Ann Oncol)	Non-randomized interventional	USA	Sacituzumab govitecan	Multiple epithelial cancers, stage IV (metastatic)	NCT01631552	495	67.5	Median: 61	NR	Systemic therapy
[27] (J Clin Oncol)	Randomized interventional	International	Datopotamab deruxtecan	Breast, inoperable/metastatic HR-positive/HER2-negative	NCT05104866	732	98.8	NR	NR	Systemic therapy
[28] (Blood)	Non-randomized interventional	USA	Brentuximab vedotin	Cutaneous T cell lymphoma, stage ≥ IB	NCT02616965	7	28	Median: 64	NR	Systemic therapy
[29] (J Clin Oncol)	Observational study	France	Trastuzumab deruxtecan	Breast, metastatic/uNResectable HER2-low	NR	22	100	Median: 57.9	NR	Systemic therapy
[30] (J Clin Oncol)	Observational study	Brazil	Trastuzumab emtansine	Breast, metastatic	NR	73	NR	NR	NR	NR
[31] (ESMO Open)	Observational study	International	Trastuzumab deruxtecan	Breast, advanced	NR	27	NR	Median: 74	NR	Systemic therapy
[32](Ann Oncol)	Non-randomized interventional	International	Rovalpituzumab tesirine	Lung, small-cell lung cancer, previously treated	NCT03000257	31	NR	NR	NR	Systemic therapy
[33] (J Clin Oncol)	Non-randomized interventional	International	Telisotuzumab vedotin	Lung, locally advanced/metastatic non-small-cell lung cancer	NCT03539536	136	NR	NR	NR	Systemic therapy
[34] (Hemasphere)	Non-randomized interventional	International	Camidanlumab tesirine	Classical Hodgkin lymphoma, relapsed/refractory	NCT04052997	117	38	Median: 37	NR	Systemic therapy
[35] (Cancer Chemother Pharmacol)	Non-randomized interventional	International	CMB-401	Gynecologic, platinum-sensitive recurrent epithelial ovarian carcinoma	NR	21	100	Mean: 64	NR	Systemic therapy
[38] (Blood)	Non-randomized interventional	USA	Brentuximab vedotin	Hodgkin lymphoma, relapsed/refractory	NR	16	44	Median: 33	NR	NR
[37] (J Clin Oncol)	Observational study	China	Disitamab vedotin	Urothelial carcinoma, metastatic	NR	63	28.6	Mean: 67	NR	NR
[36] (J Clin Oncol)	Non-randomized interventional	China	BC3195	Solid tumors, advanced	NCT05957471	9	44	Median: 59	NR	NR
[39] (J Clin Oncol)	Non-randomized interventional	China	TQB2103	Advanced solid tumors, advanced/metastatic	NCT05867563	59	NR	NR	NR	NR
[40] (Blood)	Non-randomized interventional	International	GSK2857916	Multiple myeloma, relapsed/refractory	NCT02064387	24	50	Median: 60	NR	Systemic therapy
[41] (J Clin Oncol)	Non-randomized interventional	International	ABBV-706	Advanced solid tumors, advanced/metastatic	NCT05599984	191	NR	NR	NR	NR
[42] (EHA Library)	Non-randomized interventional	NR	IMGN779	Acute myeloid leukemia, relapsed or refractory	NR	17	53	Median: 62	NR	NR
[43] (Invest New Drugs)	Non-randomized interventional	USA	ASG-5ME	Pancreatic adenocarcinoma and gastric adenocarcinoma, metastatic	NR	50	NR	NR	NR	Systemic therapy
Coward et al., 2025 (J Clin Oncol)	Non-randomized interventional	International	AMT-116	Advanced solid tumors, advanced	NCT05725291	8	NR	NR	NR	NR
[45] (J Clin Oncol)	Non-randomized interventional	USA	Dstp3086s	Prostate, metastatic castration-resistant	NCT01283373	84	0	Median: 68	NR	Systemic therapy
[46] (Leukemia)	Non-randomized interventional	USA	Gemtuzumab ozogamicin	Acute myeloid leukemia and high-risk myelodysplastic syndrome, newly diagnosed and relapsed/refractory	NCT00882102	110	43	Median: 70	NR	NR
[47] (Blood)	Non-randomized interventional	International	IMGN632	Acute myeloid leukemia or blastic plasmacytoid dendritic cell neoplasm, relapsed/refractory	NR	74	NR	Median: 69	Steroid	Systemic therapy
[48] (Blood)	Non-randomized interventional	International	Pivekimab sunirine	Acute myeloid leukemia, newly diagnosed	NR	50	NR	Median: 74	NR	NR
[49] (Lancet Oncol)	Non-randomized interventional	International	Tisotumab vedotin	Multiple solid tumours, advanced/metastatic	NCT02001623	147	69	Median: 59 (IQR: 52–67)	NR	NR
De Miguel et al., 2024 (Ann Oncol)	Non-randomized interventional	International	ABBV-400	Lung, advanced/metastatic non-squamous EGFR wildtype NSCLC	NCT05029882	48	NR	Median: 66	NR	Systemic therapy
[51] (Clin Cancer Res)	Non-randomized interventional	International	ABBV-085	Sarcoma, advanced or metastatic	NCT02565758	85	NR	Median: 58	NR	NR
[52] (Eur J Hosp Pharm)	Observational study	NR	Sacituzumab govitecan	Breast, metastatic triple-negative breast cancer	NR	25	NR	Median: 62	NR	NR
[53] (Hemasphere)	Non-randomized interventional	International	Polatuzumab vedotin	B cell non-Hodgkin lymphoma, relapsed/refractory	NCT03671018	22	NR	Median: 70	NR	Systemic therapy
[54] (Ann Oncol)	Non-randomized interventional	International	DS-7300	Advanced solid tumors, advanced/metastatic	NCT04145622	127	13.4	Median: 67	NR	NR
[55] (Hematological Oncol)	Observational study	Spain	Brentuximab vedotin	Peripheral T cell lymphoma, Ann Arbor stage III–IV in 70%	NR	20	NR	Median: 66	NR	NR
[56] (J Clin Oncol)	Non-randomized interventional	USA	Brentuximab vedotin	Cutaneous T cell lymphoma (mycosis fungoides, primary cutaneous anaplastic large-cell lymphoma) and lymphomatoid papulosis; mixed stages	NCT01352520	54	50	Median: 59.5	NR	Systemic therapy
[57] (Cancer Res)	Non-randomized interventional	International	Zanidatamab	Breast, uNResectable locally advanced or metastatic HER2 +/HR +	NCT04224272	34	NR	Median: 52	NR	Systemic therapy
[58] (Clin Cancer Res)	Non-randomized interventional	USA	Brentuximab vedotin	Hodgkin lymphoma and systemic ALCL, relapsed/refractory CD30-positive	NR	44	30	Median: 33	NR	Systemic therapy
[59] (Blood)	Non-randomized interventional	USA	Brentuximab vedotin	Hodgkin lymphoma, stage I–IV	NCT01716806	27	48	Median: 78	NR	NR
[60] (J Clin Oncol)	Non-randomized interventional	USA	Enfortumab vedotin	Urothelial carcinoma, locally advanced or metastatic	NCT05524545	20	NR	NR	NR	Systemic therapy
[61] (J Clin Oncol)	Non-randomized interventional	USA	MLN2704	Prostate cancer, metastatic castration-resistant	NR	23	NR	Median: 66	NR	Systemic therapy
[62] (J Clin Oncol)	Non-randomized interventional	China	9MW2921	Solid tumors, advanced	NCT05990452	39	79.5	Mean: 55.6	NR	Systemic therapy
[63] (Int J Cancer)	Non-randomized interventional	USA	PF-06647263	Triple-negative breast cancer, metastatic	NCT02078752	60	90	NR	NR	Systemic therapy
[64] (Cancer)	Non-randomized interventional	USA	Lorvotuzumab mertansine	Pediatric CD56-expressing solid tumors, relapsed or refractory	NR	61	42.6	Median: 13.9	Steroid	Systemic therapy
[65] (Clin Cancer Res)	Non-randomized interventional	International	DS-6157A	Gastrointestinal stromal tumor, advanced/metastatic	NCT04276415	34	44.1	Median: 60.5	NR	Systemic therapy
[66] (Clin Cancer Res)	Non-randomized interventional	International	Lifastuzumab vedotin	Non-small-cell lung cancer and platinum-resistant ovarian cancer, Advanced/metastatic	NCT01363947	87	72	Median: 64	NR	Systemic therapy
[67] (Blood)	Pooled analysis	International	Brentuximab vedotin	Hodgkin lymphoma, Post-allogeneic stem cell transplant relapse	NCT01026233; NCT01026415; NCT00947856	25	48	Median: 32	NR	Systemic therapy
[68] (Blood)	Non-randomized interventional	International	Loncastuximab tesirine	B cell non-Hodgkin lymphoma, relapsed/refractory	NCT02669017	183	37.7	Median: 63	NR	Systemic therapy
[69] (Gynecol Oncol)	Non-randomized interventional	USA	Tamrintamab pamozirine	Epithelial ovarian carcinoma, platinum-resistant/refractory	NCT02539719	74	100	Median: 65	Steroid	Systemic therapy
[72] (J Clin Oncol)	Non-randomized interventional	USA	DS-6000A	Renal cell carcinoma and ovarian cancer, advanced	NCT04707248	22	NR	Median: 63.5	NR	Systemic therapy
[70] (J Clin Oncol)	Non-randomized interventional	USA	Patritumab deruxtecan	Breast, metastatic	NCT04699630	60	98.3	Median: 57.5	NR	NR
[73] (Int J Gynecol Cancer)	Non-randomized interventional	NR	XMT-1592	Ovarian cancer and non-small-cell lung cancer, refractory	NR	31	NR	NR	NR	NR
[71] (J Clin Oncol)	Non-randomized interventional	USA	Emiltatug ledadotin	Advanced/metastatic triple-negative breast cancer, HR +/HER2 − breast cancer, ovarian cancer, endometrial cancer and adenoid cystic carcinoma type 1	NCT05377996	130	NR	Median: 55	NR	Systemic therapy
[74] (Ann Oncol)	Non-randomized interventional	USA	Rovalpituzumab tesirine	Small-cell lung cancer, extensive stage	NCT02819999	26	NR	Median: 66	NR	Systemic therapy
[75] (Br J Cancer)	Observational study	UK	Sacituzumab govitecan	Breast, metastatic triple-negative breast cancer	NR	132	99.2	Median: 56	NR	Systemic therapy
[76] (J Clin Oncol)	Non-randomized interventional	USA	Anetumab ravtasine	Mesothelin-expressing solid tumors, advanced/metastatic	NCT01439152	148	64	Mean: 60.4 (± 12.6)	NR	Systemic therapy
[77] (Blood)	Observational study	China	Polatuzumab vedotin	Diffuse large B cell lymphoma, untreated and relapsed/refractory	NR	55	NR	NR	NR	NR
[78] (J Clin Oncol)	Non-randomized interventional	International	MYTX-011	Lung, previously treated locally advanced or metastatic NSCLC	NCT05652868	59	NR	Median: 67	NR	NR
[79] (Ann Oncol)	Non-randomized interventional	International	Telisotuzumab vedotin	Lung, locally advanced/metastatic non-small-cell lung cancer	NCT03539536	136	NR	NR	NR	Systemic therapy
[80] (Ann Oncol)	Non-randomized interventional	International	Telisotuzumab vedotin	Lung, locally advanced/metastatic	NCT02099058	38	66	Median: 60	NR	Systemic therapy
[81] (Blood)	Non-randomized interventional	International	Camidanlumab tesirine	Lymphoma and myeloma, relapsed/refractory	NCT02432235	56	35.7	Median: 53.5	NR	NR
[83] (Blood Adv)	Non-randomized interventional	USA	Loncastuximab tesirine	B cell acute lymphoblastic leukemia, relapsed/refractory	NCT02669264	35	46	Median: 55	NR	Systemic therapy
[83] (EHA Library)	Non-randomized interventional	NR	Inotuzumab ozogamicin	Acute lymphoblastic leukemia and CML lymphoid blast phase, relapsed/refractory	NCT02311998	18	NR	Median: 62	NR	Systemic therapy
[84] (JTO Clin Res Rep)	Non-randomized interventional	Netherlands	Trastuzumab emtansine	NSCLC, stage IV	NCT03784599	27	NR	NR	NR	Systemic therapy
[85] (J Clin Oncol)	Non-randomized interventional	China	BAT8006	Ovarian cancer, advanced (platinum-resistant)	NCT05378737	52	NR	NR	NR	Systemic therapy
[87] (J Clin Oncol)	Non-randomized interventional	China	MRG002	Breast, advanced/metastatic	NCT04742153	56	100	Median: 55	NR	Systemic therapy
[86] (J Clin Oncol)	Non-randomized interventional	China	BAT8007	Advanced solid tumors, advanced/metastatic	NCT05879627	16	NR	NR	NR	NR
[88] (J Clin Oncol)	Non-randomized interventional	USA	CX-2029	Advanced solid tumors	NCT03543813	34	41	Median: 59	NR	Systemic therapy
[89] (J Thorac Oncol)	Randomized interventional	International	Rovalpituzumab tesirine	Lung, extensive-stage small-cell lung cancer	NCT03033511	748	NR	Median: 64	NR	Systemic therapy
[90] (Ann Oncol)	Non-randomized interventional	Japan	Tusmitamab ravtansine	Advanced solid tumors, locally advanced/metastatic	NCT03324113	16	37.5	Median: 57	NR	NR
[183] (Blood)	Non-randomized interventional	USA	Brentuximab vedotin	Hodgkin lymphoma, relapsed/refractory	NR	29	38	Median: 36	NR	Systemic therapy
[91] (Clin Lymphoma Myeloma Leuk)	Non-randomized interventional	International	Coltuximab ravtansine	Lymphoma and myeloma, relapsed or refractory B cell acute lymphoblastic leukemia	NCT01440179	36	39	Median: 50	NR	Systemic therapy
[92] (Blood)	Non-randomized interventional	USA	Indatuximab ravtansine	Multiple myeloma, relapsed/refractory	NR	64	NR	NR	NR	NR
[93] (Hematological Oncol)	Non-randomized interventional	International	Brentuximab vedotin	Cutaneous T cell lymphoma (mycosis fungoides), not stated	NR	19	NR	NR	NR	NR
[94] (Cancer Chemother Pharmacol)	Non-randomized interventional	USA	Tak-164	Gastrointestinal, advanced/metastatic	NCT03449030	31	58.1	Median: 58	NR	Systemic therapy
[95] (Oncologist)	Randomized interventional	International	AGS-16C3F	Renal cell carcinoma, metastatic	NCT02639182	133	26.3	NR	NR	Systemic therapy
[96] (Int J Gynecol Cancer)	Non-randomized interventional	NR	TORL-1–23	Advanced solid tumors, advanced	NCT05103683	45	88.9	NR	NR	Systemic therapy
[97] (Cancer Res)	Non-randomized interventional	International	BYON3521	Solid tumors, locally advanced or metastatic	NCT05323045	8	12.5	Median: 61	NR	NR
[98] (Cancer Res)	Non-randomized interventional	International	Datopotamab deruxtecan	Breast, advanced/metastatic triple-negative	NCT03401385	43	NR	Median: 53	NR	Systemic therapy
[99] (Am J Gastroenterol)	Observational study	USA	Brentuximab vedotin	Lymphoma and myeloma, not reported	NR	64	NR	Median: 55	NR	NR
[100] (Blood) ^**100**^	Non-randomized interventional	Australia	Belantamab mafodotin	Multiple myeloma, relapsed and refractory	NR	55	38	Median: 69.7	NR	Systemic therapy
[101] (Invest New Drugs)	Non-randomized interventional	International	ABBV-176	Gastrointestinal, advanced or metastatic solid tumors	NCT03145909	19	57.9	Median: 56	NR	NR
[102] (Cancer Res) ^**102**^	Non-randomized interventional	International	GQ1001	HER2-positive advanced solid tumors, advanced	NCT04450732	32	NR	NR	NR	Systemic therapy
[103] (J Clin Oncol)	Non-randomized interventional	International	EBC-129	Pancreatic ductal adenocarcinoma, locally advanced or metastatic	NCT05701527	21	NR	Mean: 63	NR	Systemic therapy
[104] (Blood)	Observational study	China	Brentuximab vedotin	CD30-positive lymphoma, not stated	NR	66	34.9	Median: 40	NR	Systemic therapy
[105] (J Clin Oncol)	Non-randomized interventional	China	SYS6010	Gastrointestinal, advanced	NR	25	NR	NR	NR	Systemic therapy
[107] (Ann Oncol)	Non-randomized interventional	USA	DMUC5754A	Ovarian cancer (platinum-resistant) and uNResectable pancreatic cancer, advanced/metastatic	NCT01335958	77	NR	NR	NR	Systemic therapy
[106] (Gynecol Oncol)	Non-randomized interventional	USA	DMUC4064A	Ovarian cancer, platinum-resistant	NCT02146313	65	100	Median: 62	NR	NR
[5, 108, 110, 111] (Ann Oncol)	Non-randomized interventional	International	IBI343	Gastric/gastro-esophageal junction adenocarcinoma, advanced/metastatic	NCT05458219	159	NR	NR	NR	Systemic therapy
[5, 108, 110, 111] (Blood)	Observational study	China	Brentuximab vedotin	Lymphoma (CD30-positive), mixed stages	NR	115	NR	NR	NR	NR
[109] (J Clin Oncol)	Observational study	China	Disitamab vedotin	Bladder cancer, high-risk non–muscle-invasive	NR	30	NR	NR	NR	Surgery
[5, 108, 110, 111] (J Clin Oncol)	Non-randomized interventional	China	JSKN003	Advanced solid tumors, advanced	NCT05744427	46	NR	NR	NR	Systemic therapy
[112] (Lancet Haematol)	Non-randomized interventional	International	Brentuximab vedotin	Lymphoma and myeloma, relapsed or refractory	NCT01492088	36	31	Median: 14 (IQR: 10.5–15.5)	NR	Systemic therapy
[113] (Eur J Cancer)	Randomized interventional	International	Sacituzumab govitecan	Breast, metastatic triple-negative breast cancer	NCT02574455	419	NR	NR	NR	Systemic therapy
[114, 116] (Ann Oncol	Non-randomized interventional	China	BL-B01D1	Biliary tract carcinoma, locally advanced or metastatic	NCT05262491	39	NR	NR	NR	Systemic therapy
[115] (Cancer Res)	Non-randomized interventional	NR	SYS6010	Advanced solid tumors, advanced	NR	232	NR	NR	NR	Systemic therapy
[114, 116] (J Clin Oncol)	Non-randomized interventional	China	MHB036C	Solid tumors, locally advanced or metastatic	NR	26	NR	NR	NR	NR
[117] (J Clin Oncol)	Non-randomized interventional	International	ATG-022	Gastric cancer, advanced/metastatic	NCT05718895	37	NR	Median: 61	NR	Systemic therapy
[118] (Clin Cancer Res)	Non-randomized interventional	International	Cofetuzumab pelidotin	Advanced solid tumors, locally advanced/metastatic	NCT02222922	137	84.7	NR	5HT3RA	Systemic therapy
[119] (Cancer Res)	Randomized interventional	International	Sacituzumab govitecan	Breast, metastatic HR +/HER2 −	NCT03901339	543	NR	NR	NR	Systemic therapy
[120] (Front Pharmacol)	Observational study	Italy	Brentuximab vedotin	Hodgkin’s lymphoma, not reported	NR	2	NR	Median: 16	NR	NR
[121] (Invest New Drugs)	Non-randomized interventional	International	AMG172	Clear cell renal cell carcinoma, stage IV	NCT01497821	37	13.5	NR	NR	Systemic therapy
[122] (Invest New Drugs)	Non-randomized interventional	USA	CDX-014	Renal cell carcinoma, advanced/metastatic	NCT02837991	16	6	Median: 67	NR	Systemic therapy
[123] (Clin Lymphoma Myeloma Leuk)	Non-randomized interventional	USA	Brentuximab vedotin	Classical Hodgkin lymphoma, relapsed/refractory	NCT02744612	39	33	Median: 33	NR	Systemic therapy
[125] (Ann Oncol)	Non-randomized interventional	International	Puxitatug samrotecan	Multiple solid tumors, advanced/metastatic	NCT05123482	46	NR	Median: 56	NR	Systemic therapy
[124] (J Clin Oncol)	Non-randomized interventional	International	Datopotamab deruxtecan	Urothelial cancer, stage III/IV (locally advanced/metastatic)	NCT03401385	40	NR	NR	NR	Systemic therapy
[126] (Cancer Research Communications)	Non-randomized interventional	International	Zilovertamab vedotin	Multiple solid tumors, metastatic	NCT04504916	102	86	Median: 60	NR	Systemic therapy
[127] (Blood Adv)	Non-randomized interventional	USA	Inotuzumab ozogamicin	Acute lymphoblastic leukemia, post-allo-HCT in complete remission at high risk of relapse	NCT03104491	18	50	Median: 44	NR	Systemic therapy
[128] (Clin Cancer Res)	Non-randomized interventional	International	Sacituzumab govitecan	Head and neck, locally recurrent or metastatic	NCT03964727	43	23	Median: 62	NR	Systemic therapy
[129] (J Clin Oncol)	Non-randomized interventional	International	Mirvetuximab soravtansine	Gynecologic, platinum-resistant epithelial ovarian cancer	NCT01609556	37	NR	NR	NR	Systemic therapy
[130] (J Clin Oncol)	Non-randomized interventional	International	DB-1303	Multiple solid tumors, advanced/metastatic	NCT05150691	85	NR	NR	NR	Systemic therapy
[131] (J Clin Oncol)	Non-randomized interventional	International	ABBV-011	Lung, relapsed/refractory small-cell lung cancer	NCT03639194	99	50	Median: 63	NR	Systemic therapy
[132] (Blood)	Non-randomized interventional	USA	Brentuximab vedotin	Mycosis fungoides, relapsed or refractory	NR	33	21	Median: 61	NR	NR
[133] (Ann Oncol)	Non-randomized interventional	Japan	Telisotuzumab vedotin	Advanced solid tumors, advanced	NCT03311477	9	44	Median: 58	NR	NR
[134] (J Clin Oncol)	Non-randomized interventional	Japan	MORAB-202	Ovarian cancer, platinum-resistant	NCT03386942	45	NR	NR	NR	Systemic therapy
[135] (Blood)	Randomized interventional	International	Belantamab mafodotin	Multiple myeloma, relapsed/refractory	NCT03525678	95	NR	NR	NR	Systemic therapy
[52] (J Clin Oncol)	Non-randomized interventional	USA	Sacituzumab govitecan	Breast, metastatic triple negative	NCT04039230	26	NR	Median: 54	NR	Systemic therapy
[136] (Ann Oncol)	Non-randomized interventional	International	SGN-PDL1V	PDL1-expressing solid tumors, advanced	NCT05208762	55	45.5	Median: 60	NR	NR
Oliveira et al., [223] (Ann Oncology)	Non-randomized interventional	Spain	Patritumab deruxtecan	Breast, early (HR-positive/HER2-negative)	NCT04610528	78	100	Mean: 52	NR	NR
[137] (Ann Oncol)	Randomized interventional	Japan	Trastuzumab deruxtecan	Gastric cancer, not specified	jRCTs031200336	58	NR	NR	NR	NR
[138] (Ann Oncol)	Non-randomized interventional	USA	Glembatumumab vedotin	Melanoma and skin, Advanced/metastatic	NCT02302339	62	45	Median: 67	NR	Systemic therapy
[139] (J Clin Oncol)	Observational study	USA	Sacituzumab govitecan	Urothelial carcinoma, locally advanced/metastatic	NR	86	30	Median: 71	NR	Systemic therapy
[140] (J Clin Oncol)	Observational study	USA	Sacituzumab govitecan	Urothelial cancer, locally advanced/metastatic	NR	220	27	Median: 66	NR	Systemic therapy
[141] (J Clin Oncol)	Non-randomized interventional	Australia	JSKN003	Advanced/metastatic solid tumors	NCT05494918	32	NR	NR	NR	Systemic therapy
[142, 143] (Ann Oncol)	Non-randomized interventional	International	DS-9606A	Multiple solid tumors, locally advanced/metastatic	NCT05394675	40	60	Median: 58	NR	Systemic therapy
[142, 143] (Blood)	Non-randomized interventional	USA	Inotuzumab ozogamicin	Acute lymphoblastic leukemia (Ph +), newly diagnosed	NR	18	50	Median: 61	NR	NR
[144] (Blood)	Non-randomized interventional	International	IMGN632	Blastic plasmacytoid dendritic cell neoplasm, relapsed/refractory	NR	23	26	Median: 73	NR	Systemic therapy
[145] (J Clin Oncol)	Non-randomized interventional	International	BG-C9074	Advanced solid tumors, locally advanced or metastatic	NCT06233942	55	NR	NR	NR	NR
[146] (Clin Cancer Res)	Non-randomized interventional	International	AGS15E	Urothelial carcinoma, metastatic	NCT01963052	93	20.4	Median: 67	NR	Systemic therapy
[147] (Invest New Drugs)	Non-randomized interventional	USA	SGN-CD70A	Non-Hodgkin lymphoma (DLBCL, MCL, FL3b), relapsed/refractory	NCT02216890	20	20	Median: 64.5	NR	Systemic therapy
[148] (Ann Oncol)	Non-randomized interventional	International	Patritumab deruxtecan	Breast, advanced/metastatic HR +/HER2 −	NCT04965766	99	NR	Median: 57 (IQR: 48.0–66.0)	NR	Systemic therapy
[149] (ESMO Open)	Observational study	Italy	Trastuzumab emtansine	Breast, early (residual disease after neoadjuvant)	NR	202	NR	Median: 52 (IQR: 45–59)	NR	Systemic therapy
[150] (Nat Med)	Non-randomized interventional	International	Patritumab deruxtecan	Solid tumor leptomeningeal metastases, metastatic	NCT05865990	20	90	Median: 51.5	NR	Systemic therapy
[151] (Clin Cancer Res)	Non-randomized interventional	USA	Cofetuzumab pelidotin	Breast, metastatic triple-negative breast cancer (including ER-low, HER2-negative)	NCT03243331	18	100	Median: 53	NR	Systemic therapy
[152] (J Thorac Oncol)	Non-randomized interventional	International	Enapotamab vedotin	Non-small-cell lung cancer, stage III/IV	NCT02988817	26	42.3	Median: 65.5	NR	Systemic therapy
[153] (Blood) ^**153**^	Non-randomized interventional	USA	Brentuximab vedotin	Hodgkin lymphoma, relapsed/refractory	NR	14	35.7	Median: 34	NR	Systemic therapy
[154] (ESMO Open)	Observational study	China	Sacituzumab govitecan	Breast, stage II–III (HER2-negative)	NR	21	NR	NR	NR	NR
[155] (Gynecol Oncol)	Non-randomized interventional	USA	XMT-1536	Multiple solid tumors likely to express NaPi2b, advanced/metastatic	NCT03319628	59	81.4	NR	NR	NR
[156] (Blood)	Non-randomized interventional	International	Cevostamab	Multiple myeloma, relapsed/refractory	NCT03275103	167	NR	Median: 66	NR	Systemic therapy
[157] (Ann Oncol)	Non-randomized interventional	International	SKB264	Solid tumors, locally advanced or metastatic	NCT04152499	18	NR	NR	NR	NR
[158] (J Clin Oncol)	Non-randomized interventional	USA	CBX-12	Solid tumors, advanced or metastatic	NCT04902872	42	NR	NR	NR	NR
[159] (Invest New Drugs)	Non-randomized interventional	USA	PF-06650808	Breast cancer, advanced/metastatic	NCT02129205	40	87.5	Mean: 57.2	NR	Systemic therapy
[160] (J Clin Oncol)	Non-randomized interventional	International	Enfortumab vedotin	Urothelial carcinoma, metastatic	NCT02091999	112	NR	Median: 67	NR	Systemic therapy
[161] (Ann Oncol)	Non-randomized interventional	International	ABBV-637	Non-small-cell lung cancer, relapsed/refractory EGFR-mutated	NCT04721015	42	NR	Median: 65	NR	Systemic therapy
[162] (Lancet Oncol)	Non-randomized interventional	China	CMG901	Gastric or gastro-oesophageal junction cancer, advanced	NCT04805307	107	47	Median: 56 (IQR: 44.0–64.0)	NR	Systemic therapy
[163] (Invest New Drugs)	Non-randomized interventional	International	DEDN6526A	Melanoma and skin, uNResectable stage III–IV (metastatic)	NCT01522664	53	32	Median: 65	NR	Systemic therapy
[164] (Blood)	Non-randomized interventional	International	WVT078	Multiple myeloma, relapsed/refractory	NCT04123418	23	NR	Median: 64	NR	Systemic therapy
[165] (Oncol Res Treat)	Non-randomized interventional	International	HKT288	Ovarian cancer and renal cell carcinoma, advanced/metastatic	NCT02947152	9	44.4	Median: 70	NR	NR
[166] (Blood)	Non-randomized interventional	USA	STRO-001	Lymphoma and myeloma, relapsed/refractory	NCT03424603	25	40	Median: 64	NR	Systemic therapy
[167] (Ann Oncol)	Non-randomized interventional	International	AZD5335	Ovarian cancer, platinum-resistant recurrent	NCT05797168	28	NR	Median: 62	NR	Systemic therapy
[168] (Invest New Drugs)	Non-randomized interventional	USA	PF-06263507	Solid tumors, locally advanced or metastatic	NCT01891669	26	58	NR	NR	Systemic therapy
[169] (Invest New Drugs)	Non-randomized interventional	USA	Brentuximab vedotin	Multiple CD30-positive solid tumors, advanced/metastatic	NCT01461538	63	46	Median: 64	NR	Systemic therapy
[170] (J Clin Oncol)	Non-randomized interventional	International	Datopotamab deruxtecan	Non-small-cell lung cancer, Locally advanced or metastatic	NCT03401385	210	NR	NR	NR	Systemic therapy
[171] (Ann Oncol)	Non-randomized interventional	International	IBI354	Gynecologic, advanced	NCT05636215	129	NR	Median: 57	NR	NR
[172] (J Clin Oncol)	Non-randomized interventional	International	Gemtuzumab ozogamicin	Acute myeloid leukemia, first relapse	NR	142	NR	Median: 61	NR	Systemic therapy
[173] (Cancer Res)	Non-randomized interventional	China	BL-M07D1	Breast and other solid tumors, locally advanced or metastatic	NCT05461768	107	91	Median: 54	NR	Systemic therapy
[173, 174] (Blood)	Non-randomized interventional	China	BL-M11D1	Acute myeloid leukemia, relapsed/refractory	NR	39	NR	Median: 53.9	NR	NR
[175] (Clin Lymphoma Myeloma Leuk)	Non-randomized interventional	International	PF-08046044	Lymphoma and myeloma, relapsed/refractory	NCT06254495	15	47	Median: 41	NR	Systemic therapy
[176] (Ann Oncol)	Non-randomized interventional	USA	Sacituzumab govitecan	Breast, stage I–III triple-negative	NCT04230109	50	100	Median: 48.5	NR	NR
[177] (Blood)	Non-randomized interventional	USA	Zilovertamab vedotin	Lymphoma and myeloma, relapsed/refractory (DLBCL, MCL, Richter transformation)	NCT03833180	54	43	Median: 70	NR	Systemic therapy
[178] (Blood)	Non-randomized interventional	USA	Vadastuximab talirine	Acute myeloid leukemia, relapsed/refractory and treatment-naive older adults	NCT01902329	131	NR	Median: 73	NR	NR
[179] (J Clin Oncol)	Non-randomized interventional	International	Telisotuzumab vedotin	Non-small-cell lung cancer, advanced/metastatic	NCT02099058	48	47.9	Median: 65	NR	NR
[180] (Hemasphere)	Randomized interventional	International	Brentuximab vedotin	Classical Hodgkin lymphoma, relapsed/refractory	NCT04378647	86	39.5	Median: 39	NR	Systemic therapy
[181] (Ann Oncol)	Non-randomized interventional	International	Tusmitamab ravtansine	Advanced solid tumors, advanced/metastatic	NCT02187848	43	NR	NR	NR	NR
[182] (Invest New Drugs)	Non-randomized interventional	USA	Vorsetuzumab mafodotin	CD70-positive relapsed/refractory non-Hodgkin lymphoma or metastatic renal cell carcinoma	NCT01015911	58	24	Median: 60.5	NR	Systemic therapy
[183] (Clin Lymphoma Myeloma Leuk)	Non-randomized interventional	USA	Brentuximab vedotin	Classical Hodgkin lymphoma, relapsed/refractory	NR	29	37.9	Median: 35.9	NR	Systemic therapy
[185] (J Oncol Pharm Prac)	Observational study	Uk	Trastuzumab deruxtecan	Breast, advanced or metastatic HER2-positive	NR	36	NR	NR	NR	Systemic therapy
[185] (Clin Cancer Res)	Non-randomized interventional	USA	DLYE5953A	Multiple solid tumors, locally advanced or metastatic	NCT02092792	68	81	Median: 58	NR	Systemic therapy
[186] (J Clin Oncol)	Non-randomized interventional	USA	Cantuzumab mertansine	Advanced solid tumors (primarily colorectal and pancreatic; some NSCLC), refractory	NR	37	43.2	Median: 54	5HT3RA	Systemic therapy
[187] (Ann Oncol)	Non-randomized interventional	USA	Ladiratuzumab vedotin	Breast, metastatic (TNBC and HR +/HER2 −)	NCT01969643	81	100	Median: 55	NR	NR
[188] (Ann Oncol)	Non-randomized interventional	International	Trastuzumab deruxtecan	Multiple HER2-expressing solid tumors, advanced/metastatic	NCT02564900	137	NR	NR	NR	Systemic therapy
[190] (J Thorac Oncol)	Non-randomized interventional	International	Trastuzumab deruxtecan	Lung, advanced NSCLC	NR	12	NR	Median: 58.5	NR	Systemic therapy
[190] (Clin Cancer Res)	Non-randomized interventional	International	ABBV-838	Multiple myeloma, relapsed/refractory	NCT02462525	75	41.3	Median: 64	NR	Systemic therapy
[191] (Br J Haem)	Case series	Uk	Belantamab mafodotin	Multiple myeloma, relapsed/refractory	NR	13	NR	Mean: 60	NR	Systemic therapy
[194] (Blood)	Non-randomized interventional	USA	VLS-101	Lymphoma and myeloma, relapsed/refractory	NR	32	40.6	Median: 70	NR	Systemic therapy
[192, 193, 197] (Cancer Res)	Non-randomized interventional	NR	SHR-A1921	Advanced solid tumors, advanced/metastatic	NCT05154604	38	NR	NR	NR	Systemic therapy
[192, 193, 197] (J Clin Oncol)	Non-randomized interventional	China	SYSA1801	Gastrointestinal, resistant/refractory advanced solid tumors	NCT05009966	33	NR	Median: 59	NR	Systemic therapy
[192, 193, 197] (J Clin Oncol)	Non-randomized interventional	China	HS-20093	Mixed solid tumors, advanced	NCT05276609	53	NR	NR	NR	Systemic therapy
[197] (J Coll Physicians Surg Pak)	Observational study	China	Trastuzumab deruxtecan	Breast, stage IV (metastatic)	NR	11	100	Median: 43	NR	Systemic therapy
[196] (J Clin Oncol)	Randomized interventional	China	TQB2102	Breast, recurrent/metastatic	NCT06115902	73	100	Median: 53	NR	Systemic therapy
Waqar et al., [198] (J Clin Oncol) [199]	Non-randomized interventional	International	Datopotamab deruxtecan	Lung, advanced or metastatic non-small-cell lung cancer (first line)	NCT04612751	40	NR	NR	NR	NR
[199] (Blood)	Non-randomized interventional	USA	MGTA-117	Acute myeloid leukemia or MDS-EB, relapsed/refractory	NCT05223699	6	NR	NR	NR	Systemic therapy
[200] (Blood)	Case series	USA	Luveltamab tazevibulin	Acute myeloid leukemia, relapsed/refractory CBFA2T3::GLIS2	NR	25	NR	Median: 2	NR	Systemic therapy
[201] (J Clin Oncol)	Non-randomized interventional	USA	Rinatabart sesutecan	Endometrial cancer, metastatic or uNResectable	NCT05579366	64	NR	NR	NR	Systemic therapy
[204] (Ann Oncol)	Non-randomized interventional	China	HS-20089	Advanced solid tumors, advanced/metastatic	NCT05263479	44	NR	NR	NR	NR
Wu et al., 2024 (Cancer Res)	Non-randomized interventional	NR	BL-B01D1	Breast, locally advanced or metastatic	NCT05470348	42	NR	NR	NR	NR
[202] (J Clin Oncol)	Non-randomized interventional	International	IBI130	Triple-negative breast cancer, Stage IV	NCT05923008	71	85.9	Median: 60	NR	NR
[205] (J Clin Oncol)	Non-randomized interventional	China	RC48-ADC	Urothelial carcinoma, locally advanced or metastatic	NCT04073602	19	NR	Median: 64	NR	Systemic therapy
[206] (Cancer Med)	Observational study	China	Disitamab vedotin	Urothelial carcinoma, locally advanced or metastatic	NR	38	23.7	Median: 67.5	NR	Systemic therapy
[207](J Clin Oncol)	Non-randomized interventional	China	Disitamab vedotin	Advanced solid tumors, advanced	NCT0511550	52	NR	NR	NR	NR
[208] (J Clin Oncol)	Non-randomized interventional	China	JSKN016	Metastatic triple-negative breast cancer	NCT06592417	6	NR	Median: 45.7	NR	Systemic therapy
[209] (Blood)	Randomized interventional	USA	Brentuximab vedotin	Diffuse large B cell lymphoma, stage IV	NCT01925612	51	NR	NR	NR	NR
[210] (Ann Oncol)	Non-randomized interventional	NR	BL-B01D1	Urothelial carcinoma, locally advanced or metastatic	NCT05785039	32	NR	NR	NR	Systemic therapy
[211] (Cancer Sci)	Non-randomized interventional	Japan	Tisotumab vedotin	Cervical cancer, recurrent/metastatic	NCT03913741	17	100	Median: 47	NR	Systemic therapy
[212] (N Engl J Med)	Non-randomized interventional	USA	Brentuximab vedotin	Relapsed or refractory CD30-positive lymphomas (Hodgkin’s lymphoma and systemic ALCL)	NCT00430846	45	38	Median: 36	NR	Systemic therapy
[213] (J Clin Oncol)	Non-randomized interventional	International	Brentuximab vedotin	Hodgkin lymphoma, relapsed or refractory after autologous stem-cell transplantation	NCT00848926	102	53	Median: 31	NR	Systemic therapy
[215] (Ann Oncol)	Non-randomized interventional	China	BL-B01D1	Lung, locally advanced or metastatic non-small-cell lung cancer	NCT05194982	114	NR	NR	NR	Systemic therapy
[214] (J Clin Oncol)	Non-randomized interventional	China	9MW2821	Multiple advanced solid tumors	NCT05216965	260	NR	NR	NR	Systemic therapy
[216] (J Clin Oncol)	Non-randomized interventional	China	BAT8006	Gynecologic, platinum-resistant ovarian cancer	NCT05378737	131	NR	NR	NR	Systemic therapy
[217] (Ann Oncol)	Non-randomized interventional	International	YL201	Advanced solid tumors, metastatic/locally advanced	NCT05434234; NCT06057922	276	NR	NR	NR	NR
[218] (J Clin Oncol)	Non-randomized interventional	China	BAT8008	Advanced solid tumors	NCT05620017	170	NR	NR	NR	Systemic therapy
[219] (J Clin Oncol)	Non-randomized interventional	China	SHR-A2102	Advanced solid tumors, locally advanced unresectable or metastatic	NCT05701709	369	NR	Median: 59	NR	NR
[221] (J Clin Oncol)	Non-randomized interventional	NR	Disitamab vedotin	Urothelial carcinoma, locally advanced or metastatic	NCT04264936	32	43.8	Median: 67	NR	NR
[220] (J Thorac Oncol)	Non-randomized interventional	China	GQ1005	Advanced solid tumors, locally advanced or metastatic	NCT06154343	131	NR	Median: 56	NR	Systemic therapy

*NR* not reported

**Fig. 2:**
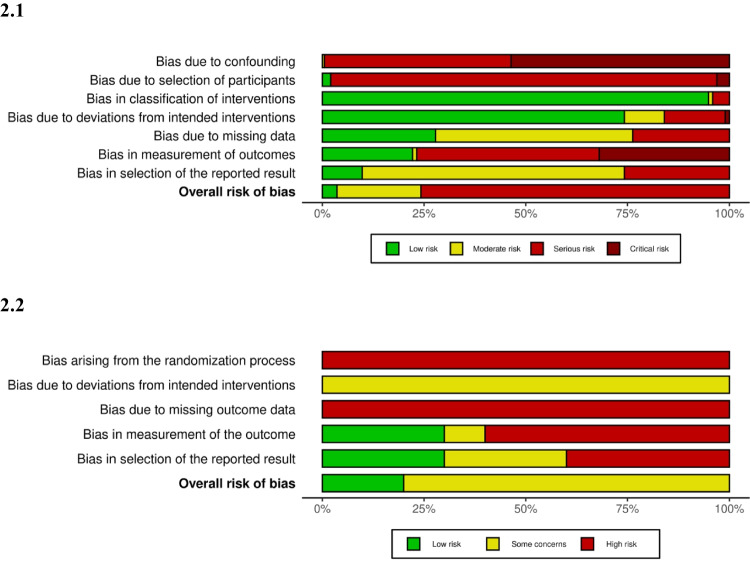
Quality assessment of included studies. (**2.1**) Interventional non-randomized studies. (**2.2**) Interventional randomized studies

The majority of studies (*n* = 175, 84%) were non-randomized interventional studies. Ten (5%) were randomized controlled trials. The remaining were observational studies. Quality assessment is summarized in Fig. 2 and detailed by study in Appendix Table 2.

**Fig. 3:**
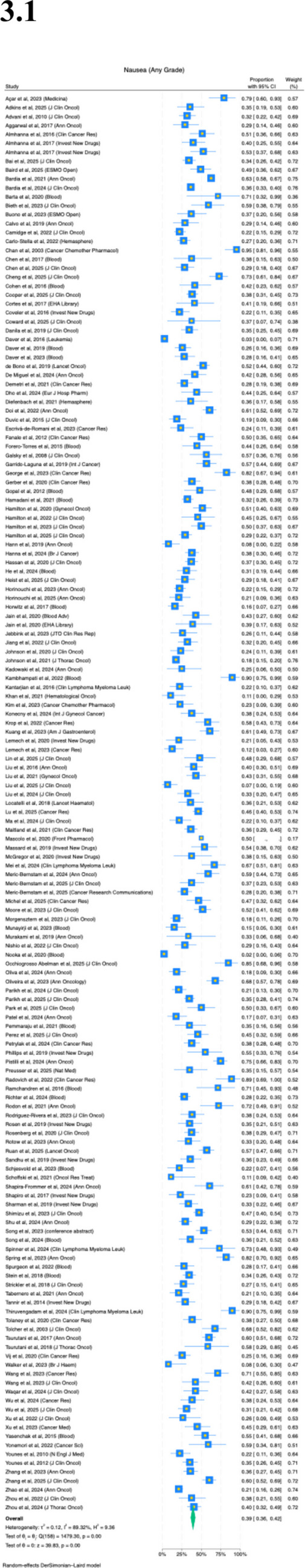

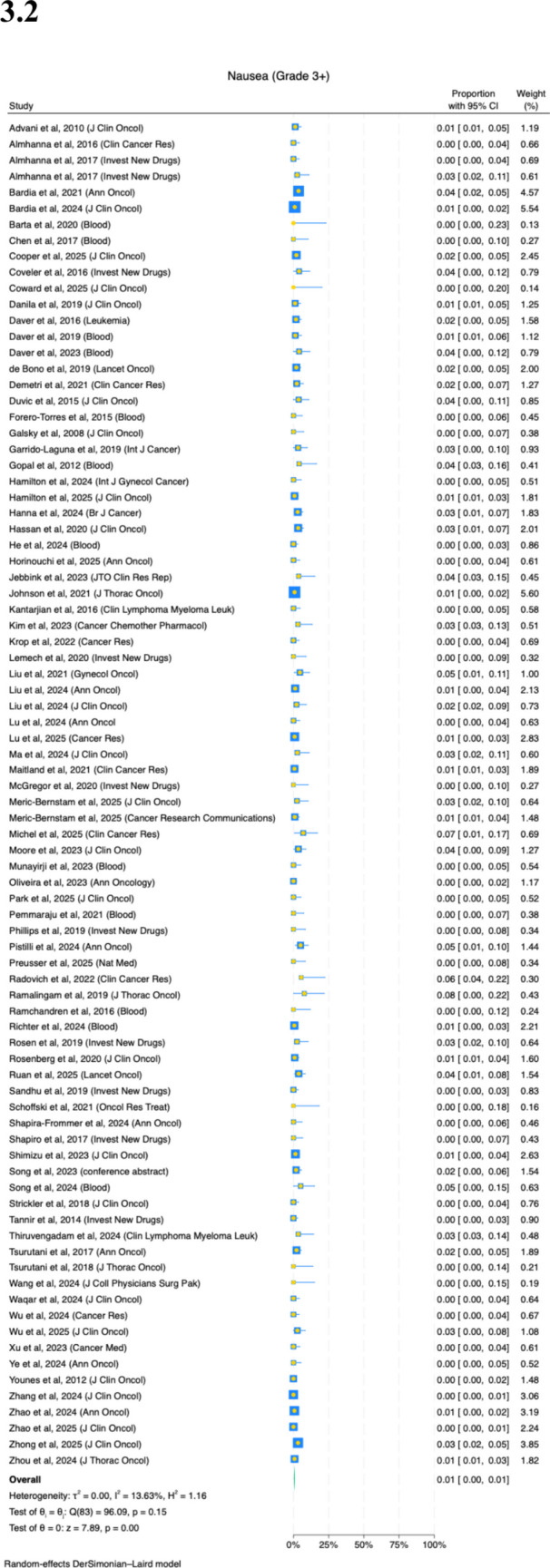
Prevalence of (**3.1**) any nausea and (**3.2**) severe nausea

A total of 159 studies reported on the prevalence of nausea. Thirty-nine percent (95%CI, 36–42%) of patients experience any grade of nausea (Fig. 3(3.1); *I*^2^ = 89%). There is variation by ADCs (Appendix Fig. 5). ADCs with higher relative risks of nausea are patritumab deruxtecan (59%, *n* = 4), trastuzumab deruxtecan (55%, *n* = 4), sacituzumab govitecan (51%, *n* = 8) and brentuximabvedotin (47%, *n* = 19). ADCs with lower relative risks of nausea are disitamab vedotin (28%, *n* = 4), telisotuzumab vedotin (22%, *n* = 5), rovalpituzumab tesirine (19%, *n* = 4) and belantamab mafodotin (6%, *n* = 3) (Table 2). There is some variation by cancer site, but not significant (Appendix Fig. 6(4.1); *p* = 0.06). Older patients experience lower rates of nausea (Appendix Fig. 6(4.2); *p* < 0.001); each 10-year increase in age is associated with a 12% decrease in nausea rates. Female patients may be at higher risk of ADCINV (Appendix Fig. 6(4.3); *p* = 0.072). Also, studies with longer median follow-up reported greater prevalence rates of nausea (Appendix Fig. 6(4.5); *p* = 0.035).

**Table 2:** Emetogenicity of antibody drug conjugated

Proposed emetogenic risk	Antibody drug conjugates
Higher relative risk of nausea (45% +)	• Cofetuzumab pelidotin, 63% (95%CI: 12–100%, *n* = 2)
	• Patritumab deruxtecan, 59% (95%CI: 44–74%, *n* = 4)
	• Trastuzumab deruxtecan, 55% (95%CI: 44–65%, *n* = 4)
	• Puxitatug samrotecan, 53% (95%CI: 44–63%, *n* = 2)
	• Tisotumab vedotin, 53% (95%CI: 45–61%, *n* = 2)
	• Sacituzumab govitecan, 51% (95%CI: 37–66%, *n* = 8)
	• Brentuximab vedotin, 47% (95%CI: 36–58%, *n* = 19)
	• Tak-264, 48% (95%CI: 39–57%, *n* = 3)
	• SYS6010, 46% (95%CI: 40–52%, *n* = 2)
Moderate relative risk of nausea (33–45%)	• Datopotamab deruxtecan, 43% (95%CI: 36–51%, *n* = 5)
	• JSKN003, 40% (95%CI: 24–58%, *n* = 2)
	• BL-B01D1, 36% (95%CI: 29–44%, *n* = 2)
	• Loncastuximab tesirine, 35% (95%CI: 26–44%, *n* = 2)
	• Gemtozumab ozogamicin, 34% (95%CI: 0–100%, *n* = 2)
	• Inotuzumab ozogamicin, 33% (95%CI: 23–43%, *n* = 2)
Lower relative risk of nausea (< 33%)	• Polatuzumab vedotin, 32% (95%CI: 22–43%, *n* = 2)
	• Zilovertamab vedotin, 28% (95%CI: 21–36%, *n* = 2)
	• Disitamab vedotin, 28% (95%CI: 14–45%, *n* = 4)
	• IMGN632, 28% (95%CI: 19–37%, *n* = 2)
	• Telisotuzumab vedotin, 22% (95%CI: 18–27%, *n* = 5)
	• Camidanlumab tesirine, 22% (95%CI: 12–34%, *n* = 2)
	• Tusamitamab ravtansine, 22% (95%CI: 12–34%, *n* = 2)
	• Rovalpituzumab tesirine, 19% (95%CI: 12–28%, *n* = 4)
	• Belantamab mafodotin, 2% (95%CI: 0–9%, *n* = 2)

Eighty-four studies reported on the prevalence of severe nausea. One percent (95%CI, 0–1%) of patients experience severe nausea (Fig. 3(3.2); *I*^2^ = 11%). There is no significant difference on any subgroup analyses by ADCs, primary cancer site, age, sex, number of patients in study or follow-up duration of study (Appendix Figs. 7–8).

**Fig. 4:**
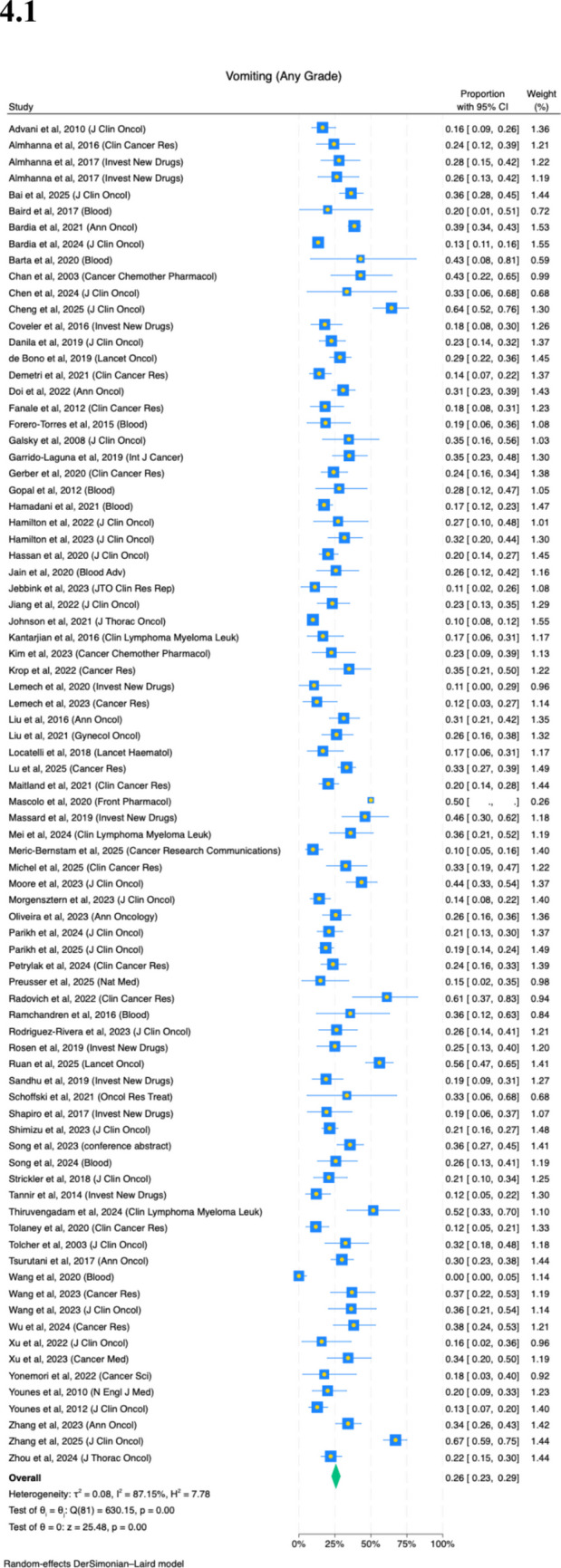

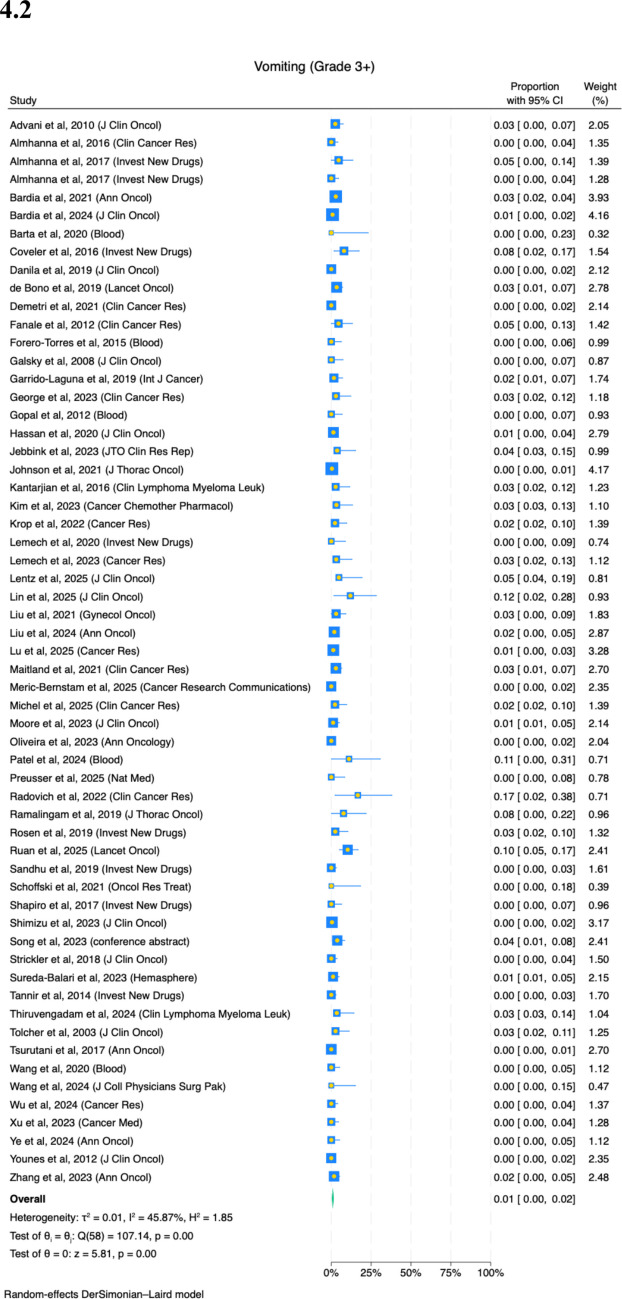
Prevalence of severe (**4.1**) nausea and (**4.2**) vomiting

Eighty-two studies reported on the prevalence of vomiting. Twenty-six percent (95%CI, 23–29%) of patients experience any grade of vomiting (Fig. 4(4.1); *I*^2^ = 87%). There is variation by ADCs (Appendix Fig. 9). ADCs with higher relative risks of vomiting were seen in cofetuzumab pelidotin (38%) and BL-B01D1 (35%), and lower rates seen in loncastuximab tesirine (19%) and datopotamab deruxtecan (21%). There is variation by primary cancer site, with gynecologic patients experiencing higher rates (Appendix Fig. 10(8.1), 39%). There is no difference by age, sex, number of patients in enrolled and follow-up duration of study (Appendix Fig. 10(8.2–8.5)).

Fifty-nine studies reported on the prevalence of severe vomiting. One percent (95%CI, 0–2%) of patients experience severe vomiting (Fig. 4(4.2); *I*^2^ = 45%). There is no significant difference on any subgroup analyses by ADCs, primary cancer site, age, sex, number of patients in study or follow-up duration of study (Appendices 11–12).

## Discussion

To our knowledge, this is the first pooled prevalence rate of ADCINV. We report that nausea occurs overall in 39% of patients, and vomiting in 26% of patients. The prevalence of severe nausea and vomiting is 1%.

Rates of CINV are reported to be 25–50% for patients receiving highly emetogenic chemotherapy, and up to 40% for patients receiving moderately emetogenic chemotherapy, despite antiemetic prophylaxis, which are comparable to rates of nausea and vomiting seen in our review [2]. The majority of the trials included in this review do not report use of antiemetic prophylaxis. A small proportion report ADCs used alongside other systemic therapies, but most report ADCs used alone. The reported rate of ADCINV of 26–40% from this study suggests that it is as prevalent issue that should be studied further as a prominent adverse effect and to tailor antiemetic prophylactic regimens towards.

There is much room for research on ADCINV. The data presented herein are not subdivided into the conventional nausea and vomiting time phases of acute, delayed or long delayed. This is expected, as the intent of clinical trials is much broader than just focusing on ADCINV. However, with future research in this field, efforts should be made to classify ADCINV into similar time phases of acute, delayed and long-delayed ADCINV. Also, future work can evaluate the use of rescue antiemetics, a topic that has been sparsely studied in ADCINV thus far.

In contrast with conventional chemotherapy, for which decades of research have defined emetogenic risk and established antiemetic prophylaxis guidelines, no specific guidance exists for ADCINV [3, 4]. ADCs have been added to emetic classification alongside chemotherapies, but they may warrant separate studies altogether. It is unknown whether ADCs trigger the same central and gastrointestinal emetic pathway as for CINV, and whether the same prophylactic and rescue options are applicable.

Our results suggest that there may be some difference in ADCINV by drug. A preliminary framework of relative emetogenic potential among ADCs can be proposed based on the current pooled prevalence data. If one were to evaluate the ADCs relative to one another in terms of emetogenicity and classify them as having higher/moderate/lower emetogenic risk relative to the other ADCs, whereby those deemed as having higher or lower emetogenic risk have a prevalence rate greater than 2 standard deviation from the pooled mean, a preliminary emetogenicity risk ladder can be formulated (Table 2), where trastuzumab deruxtecan (55%), sacituzumab govitecan (51%) and brentuximab vedotin (47%) are higher emetogenic risk compared to disitimab vedotin (28%), telisotuzumab vedotin (22%) and rovalpituzumab tesirine (19%). While these exploratory categorizations, derived from heterogeneous prophylactic settings, do not yet correspond to established guideline-based risk levels such as HEC/MEC/LEC, they may provide a foundation for future consensus development of ADC-specific emetogenic risk classification. Future work can aim to examine for differences in ADCINV between ADCs, and further evaluate emetogenic risks to establish a framework for future work on antiemetic prophylaxis.

Also, there is no research on predictors for ADCINV, understandably because this phenomenon is only just now being described/highlighted as prevalent in this manuscript. Our results suggest that there may be some patients at higher risk of ADCINV, namely that those who are older experience lower rates of nausea—every 10 years is associated with a 12% decrease in the prevalence of nausea. Female patients may also be at greater risk. As with CINV where patients who are younger, female and have a history of nausea/vomiting are known to be higher risk of CINV [224, 225], it can likewise be important to identify patients at higher risk of ADCINV and to provide appropriate prophylactic care for them.

There are strengths and limitations of this review. Strengths include a prospectively registered protocol, comprehensive search strategy across multiple databases, and duplicate independent screening and data extraction using both human reviewer and a large language model–assisted environment. Detailed quality assessment are presented transparently. Limitations include heterogeneity across studies in reporting nausea and vomiting, with inconsistent unstandardized documentation of antiemetic prophylaxis in clinical trials. Also, many studies were deemed to be of high risk of bias as they were conservatively evaluated and there were oftentimes insufficient granular detail to adhere to the Cochrane’s high standard of transparent reporting. For example, concerns were found for Domain 1 and 7 of ROBINS-I, as the statistical plan is oftentimes not reported a priori for observational studies and are not rigorously detailed to report controlling for all possible confounders. However, it is important to mention that this pertains to the study and its design as a whole, with notable issues known for statistical analysis; the concern of bias may or may not be directly attributed to the adverse effect outcome of nausea and vomiting. Finally, the screening criteria required manuscript’s abstracts to explicitly report nausea and/or vomiting to be included in this review, and there are likely other trials that also report nausea and/or vomiting as side effects. Some landmark trials report much higher rates of nausea (70%) and vomiting [223, 226]. Unfortunately, it is impractical to include every trial ever conducted in level 2 full text screening to review all reported toxicities, and therefore the best compromise is to include articles whose abstracts report nausea, which has nevertheless generated a large collection of studies. Finally, it is important to note that these nausea/vomiting rates are observed during ADC therapy, but may not necessarily be unilaterally causal; there may be other concomitant therapies, disease burden and supportive medications that may also induce nausea/vomiting. Conservatively, these results should be viewed as hypothesis-generating, whereby ADCINV is identified as a prevalent issue warranting future research.

Our findings have implications for clinicians and researchers. For clinicians, ADCINV and specifically nausea should be anticipated as a common adverse event and discussed with patients considering ADCs. As with chemotherapy, prophylactic antiemetic strategies may improve tolerability, adherence and quality of life. There are no guidelines specifically focused on exclusively ADCINV, with some ADCs suggested into grouping with other chemotherapies based on emetogenic profiles [4]. For researchers, future research should be conducted to capture nausea and vomiting using standardized phase-specific definitions, and look to evaluate for variability in rates by ADC and individual patient risk factors. With this, preliminary guidelines may be formed to guide future clinical practice.

In conclusion, ADCINV is a prevalent and clinically significant toxicity, akin to CINV. Further research is needed to characterize phase-specific nausea and vomiting, categorize ADCs by emetogenicity, investigate patient related individual risk factors, and ultimately develop optimal management strategies.

## Supplementary Information

Below is the link to the electronic supplementary material.

ESM1 (DOCX 10.4 MB)

## Data Availability

Data derived from a source in public domain (systematic review of published articles’ data).
