# Supplementary material for: Prevalence of antibody drug conjugated–induced nausea and vomiting (ADCINV) in patients with cancer
Source: Support Care Cancer. 2026 May 2;34(5):486. doi: 10.1007/s00520-026-10674-2 (PMC13135589; doi:10.1007/s00520-026-10674-2)
Supplement: Supplementary file 1 — (DOCX 10.4 MB) [file 520_2026_10674_MOESM1_ESM.docx]

**Appendix 1.** Search Strategies

**Ovid MEDLINE(R) ALL 1946 to September 24, 2025**

| **#** | **Searches** | **Results** | **Type** |  |
| --- | --- | --- | --- | --- |
| 1 | Nausea/ | 18223 | Advanced |  |
| 2 | Vomiting/ | 26262 | Advanced |  |
| 3 | Emetics/ | 898 | Advanced |  |
| 4 | nause*.mp,kw. | 89664 | Advanced |  |
| 5 | vomit*.mp,kw. | 105144 | Advanced |  |
| 6 | emesis*.mp,kw. | 7954 | Advanced |  |
| 7 | emetic?.mp,kw. | 5324 | Advanced |  |
| 8 | barf*.mp,kw. | 788 | Advanced |  |
| 9 | bilious*.mp,kw. | 1353 | Advanced |  |
| 10 | (feel* adj3 sick).mp,kw. | 305 | Advanced |  |
| 11 | (gag or gagging*).mp,kw. | 26171 | Advanced |  |
| 12 | heaving*.mp,kw. | 126 | Advanced |  |
| 13 | hyperemesis*.mp,kw. | 3206 | Advanced |  |
| 14 | (puke? or puking).mp,kw. | 10 | Advanced |  |
| 15 | (queasy or queasiness*).mp,kw. | 36 | Advanced |  |
| 16 | (retch or retching).mp,kw. | 1293 | Advanced |  |
| 17 | throw* up.mp,kw. | 165 | Advanced |  |
| 18 | or/1-17 | 172137 | Advanced |  |
| 19 | Antineoplastic Agents, Immunological/ | 12276 | Advanced |  |
| 20 | Immunoconjugates/ | 9888 | Advanced |  |
| 21 | Ado-Trastuzumab Emtansine/ | 732 | Advanced |  |
| 22 | Brentuximab Vedotin/ | 978 | Advanced |  |
| 23 | Gemtuzumab/ | 647 | Advanced |  |
| 24 | Inotuzumab Ozogamicin/ | 287 | Advanced |  |
| 25 | ((antineoplas* or anti-neoplas*) adj6 immun*).mp,kw. | 20775 | Advanced |  |
| 26 | ((antincancer* or anti-cancer*) adj6 immun*).mp,kw. | 3326 | Advanced |  |
| 27 | ((antitumo?r* or anti-tumo?r*) adj6 immun*).mp,kw. | 44491 | Advanced |  |
| 28 | ((antineoplas* or anti-neoplas*) adj6 mab?).mp,kw. | 16 | Advanced |  |
| 29 | ((antincancer* or anti-cancer*) adj6 mab?).mp,kw. | 62 | Advanced |  |
| 30 | ((antitumo?r* or anti-tumo?r*) adj6 mab?).mp,kw. | 691 | Advanced |  |
| 31 | (antibody adj3 drug? adj3 conjugat*).mp,kw. | 8671 | Advanced |  |
| 32 | trastuzumab emtansine*.mp,kw. | 1336 | Advanced |  |
| 33 | adotrastuzumab*.mp,kw. | 6 | Advanced |  |
| 34 | (hun901-dm1 or hun901dm1).mp,kw. | 5 | Advanced |  |
| 35 | kadcyla*.mp,kw. | 146 | Advanced |  |
| 36 | (pro-132365 or pro132365).mp,kw. | 2 | Advanced |  |
| 37 | (rg-3502 or rg3502).mp,kw. | 0 | Advanced |  |
| 38 | (ro-5304020 or ro5304020).mp,kw. | 0 | Advanced |  |
| 39 | (conjugat* adj3 (dm-1 or dm1)).mp,kw. | 160 | Advanced |  |
| 40 | (tmab adj3 (dm-1 or dm1)).mp,kw. | 9 | Advanced |  |
| 41 | (t dm-1 or t dm1).mp,kw. | 960 | Advanced |  |
| 42 | (tdm-1 or tdm1).mp,kw. | 121 | Advanced |  |
| 43 | (trastuzumab* adj3 (dm-1 or dm1)).mp,kw. | 736 | Advanced |  |
| 44 | ujvira*.mp,kw. | 1 | Advanced |  |
| 45 | (zrc 3256 or zrc3256).mp,kw. | 1 | Advanced |  |
| 46 | se2kh7t06f.rn. | 730 | Advanced |  |
| 47 | 1018448-65-1.rn. | 0 | Advanced |  |
| 48 | brentuximab vedotin*.mp,kw. | 1709 | Advanced |  |
| 49 | adcetris*.mp,kw. | 104 | Advanced |  |
| 50 | (cac10-1006 or cac101006).mp,kw. | 0 | Advanced |  |
| 51 | (cac10-vcmmae* or cac10vcmmae*).mp,kw. | 5 | Advanced |  |
| 52 | (sgn-35 or sgn35).mp,kw. | 46 | Advanced |  |
| 53 | 7xl5iss668.rn. | 977 | Advanced |  |
| 54 | 914088-09-8.rn. | 0 | Advanced |  |
| 55 | trastuzumab deruxtecan*.mp,kw. | 1100 | Advanced |  |
| 56 | famtrastuzumab*.mp,kw. | 1 | Advanced |  |
| 57 | (ds-8201 or ds8201).mp,kw. | 51 | Advanced |  |
| 58 | (ds-8201a or ds8201a).mp,kw. | 62 | Advanced |  |
| 59 | enhertu*.mp,kw. | 61 | Advanced |  |
| 60 | (who-10516 or who10516).mp,kw. | 1 | Advanced |  |
| 61 | 5384hk7574.rn. | 353 | Advanced |  |
| 62 | 1826843-81-5.rn. | 0 | Advanced |  |
| 63 | gemtuzumab ozogamicin*.mp,kw. | 818 | Advanced |  |
| 64 | (cdp771 or cdp-771).mp,kw. | 1 | Advanced |  |
| 65 | (cma676 or cma-676).mp,kw. | 31 | Advanced |  |
| 66 | (hP676 or hP-676).mp,kw. | 16 | Advanced |  |
| 67 | mylotarg*.mp,kw. | 168 | Advanced |  |
| 68 | 8gzg754x6m.rn. | 0 | Advanced |  |
| 69 | 220578-59-6.rn. | 0 | Advanced |  |
| 70 | inotuzumab ozogamicin*.mp,kw. | 480 | Advanced |  |
| 71 | besponsa*.mp,kw. | 19 | Advanced |  |
| 72 | calicheamicin*.mp,kw. | 463 | Advanced |  |
| 73 | (cmc544 or cmc-544).mp,kw. | 24 | Advanced |  |
| 74 | (pf05208773 or pf-05208773).mp,kw. | 0 | Advanced |  |
| 75 | (pf5208773 or pf-5208773).mp,kw. | 0 | Advanced |  |
| 76 | (way207294 or way-207294).mp,kw. | 1 | Advanced |  |
| 77 | p93ruu11p7.rn. | 287 | Advanced |  |
| 78 | 635715-01-4.rn. | 0 | Advanced |  |
| 79 | or/19-78 | 82864 | Advanced |  |
| 80 | 18 and 79 | 579 | Advanced |  |
| 81 | limit 80 to "humans only (removes records about animals)" | 568 | Advanced |  |
| 82 | limit 81 to "all child (0 to 18 years)" | 35 | Advanced |  |
| 83 | limit 81 to "all adult (19 plus years)" | 348 | Advanced |  |
| 84 | 82 not 83 | 11 | Advanced |  |
| 85 | 81 not 84 | 557 | Advanced |  |
| 86 | 85 not ((clinical trial, veterinary or clinical trial protocol or news or newspaper article or study guide or preprint).pt. or (medrxiv or biorxiv or research square).so. or (AHRQ* or StatPearls or Genereviewsovidsup).bt. or chapter.pr. or NB*.bk.) | 552 | Advanced |  |

**Ovid Embase Classic +Embase 1947 to 2025 September 24**

| **#** | **Searches** | **Results** | **Type** |  |
| --- | --- | --- | --- | --- |
| 1 | "nausea and vomiting"/ | 73085 | Advanced |  |
| 2 | nausea/ | 290223 | Advanced |  |
| 3 | chemotherapy induced nausea/ | 404 | Advanced |  |
| 4 | retching/ | 2027 | Advanced |  |
| 5 | vomiting/ | 276510 | Advanced |  |
| 6 | bilious vomiting/ | 1382 | Advanced |  |
| 7 | chemotherapy induced emesis/ | 6150 | Advanced |  |
| 8 | hyperemesis/ | 430 | Advanced |  |
| 9 | nause*.mp,kw. | 403602 | Advanced |  |
| 10 | vomit*.mp,kw. | 405786 | Advanced |  |
| 11 | emesis*.mp,kw. | 21419 | Advanced |  |
| 12 | emetic?.mp,kw. | 8974 | Advanced |  |
| 13 | barf*.mp,kw. | 984 | Advanced |  |
| 14 | bilious*.mp,kw. | 3819 | Advanced |  |
| 15 | (feel* adj3 sick).mp,kw. | 498 | Advanced |  |
| 16 | (gag or gagging*).mp,kw. | 35025 | Advanced |  |
| 17 | heaving*.mp,kw. | 225 | Advanced |  |
| 18 | hyperemesis*.mp,kw. | 6004 | Advanced |  |
| 19 | (puke? or puking).mp,kw. | 60 | Advanced |  |
| 20 | (queasy or queasiness*).mp,kw. | 72 | Advanced |  |
| 21 | (retch or retching).mp,kw. | 3183 | Advanced |  |
| 22 | throw* up.mp,kw. | 256 | Advanced |  |
| 23 | or/1-22 | 606867 | Advanced |  |
| 24 | immunological antineoplastic agent/ | 5167 | Advanced |  |
| 25 | antibody drug conjugate/ | 12071 | Advanced |  |
| 26 | trastuzumab deruxtecan/ | 3285 | Advanced |  |
| 27 | trastuzumab emtansine/ | 5555 | Advanced |  |
| 28 | brentuximab vedotin/ | 7192 | Advanced |  |
| 29 | gemtuzumab ozogamicin/ | 4744 | Advanced |  |
| 30 | inotuzumab ozogamicin/ | 2433 | Advanced |  |
| 31 | ((antineoplas* or anti-neoplas*) adj6 immun*).mp,kw. | 13512 | Advanced |  |
| 32 | ((antincancer* or anti-cancer*) adj6 immun*).mp,kw. | 5445 | Advanced |  |
| 33 | ((antitumo?r* or anti-tumo?r*) adj6 immun*).mp,kw. | 69180 | Advanced |  |
| 34 | ((antineoplas* or anti-neoplas*) adj6 mab?).mp,kw. | 17 | Advanced |  |
| 35 | ((antincancer* or anti-cancer*) adj6 mab?).mp,kw. | 101 | Advanced |  |
| 36 | ((antitumo?r* or anti-tumo?r*) adj6 mab?).mp,kw. | 1196 | Advanced |  |
| 37 | (antibody adj3 drug? adj3 conjugat*).mp,kw. | 18574 | Advanced |  |
| 38 | trastuzumab emtansine*.mp,kw. | 5767 | Advanced |  |
| 39 | adotrastuzumab*.mp,kw. | 35 | Advanced |  |
| 40 | (hun901-dm1 or hun901dm1).mp,kw. | 18 | Advanced |  |
| 41 | kadcyla*.mp,kw. | 756 | Advanced |  |
| 42 | (pro-132365 or pro132365).mp,kw. | 5 | Advanced |  |
| 43 | (rg-3502 or rg3502).mp,kw. | 2 | Advanced |  |
| 44 | (ro-5304020 or ro5304020).mp,kw. | 2 | Advanced |  |
| 45 | (conjugat* adj3 (dm-1 or dm1)).mp,kw. | 315 | Advanced |  |
| 46 | (tmab adj3 (dm-1 or dm1)).mp,kw. | 13 | Advanced |  |
| 47 | (t dm-1 or t dm1).mp,kw. | 2471 | Advanced |  |
| 48 | (tdm-1 or tdm1).mp,kw. | 554 | Advanced |  |
| 49 | (trastuzumab* adj3 (dm-1 or dm1)).mp,kw. | 1548 | Advanced |  |
| 50 | ujvira*.mp,kw. | 3 | Advanced |  |
| 51 | (zrc 3256 or zrc3256).mp,kw. | 2 | Advanced |  |
| 52 | se2kh7t06f.rn. | 0 | Advanced |  |
| 53 | 1018448-65-1.rn. | 4992 | Advanced |  |
| 54 | brentuximab vedotin*.mp,kw. | 7428 | Advanced |  |
| 55 | adcetris*.mp,kw. | 721 | Advanced |  |
| 56 | (cac10-1006 or cac101006).mp,kw. | 0 | Advanced |  |
| 57 | (cac10-vcmmae* or cac10vcmmae*).mp,kw. | 10 | Advanced |  |
| 58 | (sgn-35 or sgn35).mp,kw. | 433 | Advanced |  |
| 59 | 7xl5iss668.rn. | 0 | Advanced |  |
| 60 | 914088-09-8.rn. | 6402 | Advanced |  |
| 61 | trastuzumab deruxtecan*.mp,kw. | 3521 | Advanced |  |
| 62 | famtrastuzumab*.mp,kw. | 10 | Advanced |  |
| 63 | (ds-8201 or ds8201).mp,kw. | 254 | Advanced |  |
| 64 | (ds-8201a or ds8201a).mp,kw. | 287 | Advanced |  |
| 65 | enhertu*.mp,kw. | 273 | Advanced |  |
| 66 | (who-10516 or who10516).mp,kw. | 0 | Advanced |  |
| 67 | 5384hk7574.rn. | 0 | Advanced |  |
| 68 | 1826843-81-5.rn. | 3052 | Advanced |  |
| 69 | gemtuzumab ozogamicin*.mp,kw. | 4895 | Advanced |  |
| 70 | (cdp771 or cdp-771).mp,kw. | 6 | Advanced |  |
| 71 | (cma676 or cma-676).mp,kw. | 117 | Advanced |  |
| 72 | (hP676 or hP-676).mp,kw. | 25 | Advanced |  |
| 73 | mylotarg*.mp,kw. | 1456 | Advanced |  |
| 74 | 8gzg754x6m.rn. | 0 | Advanced |  |
| 75 | 220578-59-6.rn. | 609 | Advanced |  |
| 76 | inotuzumab ozogamicin*.mp,kw. | 2472 | Advanced |  |
| 77 | besponsa*.mp,kw. | 142 | Advanced |  |
| 78 | calicheamicin*.mp,kw. | 1437 | Advanced |  |
| 79 | (cmc544 or cmc-544).mp,kw. | 179 | Advanced |  |
| 80 | (pf05208773 or pf-05208773).mp,kw. | 2 | Advanced |  |
| 81 | (pf5208773 or pf-5208773).mp,kw. | 0 | Advanced |  |
| 82 | (way207294 or way-207294).mp,kw. | 0 | Advanced |  |
| 83 | p93ruu11p7.rn. | 0 | Advanced |  |
| 84 | 635715-01-4.rn. | 2255 | Advanced |  |
| 85 | or/24-84 | 120956 | Advanced |  |
| 86 | 23 and 85 | 4445 | Advanced |  |
| 87 | limit 86 to "humans only (removes records about animals)" | 4397 | Advanced |  |
| 88 | limit 87 to (embryo <first trimester> or infant <to one year> or child <unspecified age> or preschool child <1 to 6 years> or school child <7 to 12 years> or adolescent <13 to 17 years>) | 199 | Advanced |  |
| 89 | limit 87 to (adult <18 to 64 years> or aged <65+ years>) | 2405 | Advanced |  |
| 90 | 88 not 89 | 78 | Advanced |  |
| 91 | 87 not 90 | 4319 | Advanced |  |
| 92 | 91 not ((books or chapter or preprint or tombstone or clinical trial).pt. or "clinicaltrials.gov".jw.) | 4266 | Advanced |  |

**Ovid Cochrane Central Register of Controlled Trials August 2025**

| **#** | **Searches** | **Results** | **Type** |
| --- | --- | --- | --- |
| 1 | Nausea/ | 4717 | Advanced |
| 2 | Vomiting/ | 4387 | Advanced |
| 3 | Emetics/ | 52 | Advanced |
| 6 | nause*.mp,kw. | 63515 | Advanced |
| 7 | vomit*.mp,kw. | 50790 | Advanced |
| 8 | emesis*.mp,kw. | 3169 | Advanced |
| 9 | emetic?.mp,kw. | 2119 | Advanced |
| 10 | barf*.mp,kw. | 46 | Advanced |
| 11 | bilious*.mp,kw. | 53 | Advanced |
| 12 | (feel* adj3 sick).mp,kw. | 83 | Advanced |
| 13 | (gag or gagging*).mp,kw. | 1137 | Advanced |
| 14 | heaving*.mp,kw. | 16 | Advanced |
| 15 | hyperemesis*.mp,kw. | 260 | Advanced |
| 16 | (puke? or puking).mp,kw. | 7 | Advanced |
| 17 | (queasy or queasiness*).mp,kw. | 17 | Advanced |
| 18 | (retch or retching).mp,kw. | 923 | Advanced |
| 19 | throw* up.mp,kw. | 9 | Advanced |
| 20 | or/1-19 | 73661 | Advanced |
| 21 | Antineoplastic Agents, Immunological/ | 957 | Advanced |
| 22 | Immunoconjugates/ | 388 | Advanced |
| 23 | Ado-Trastuzumab Emtansine/ | 126 | Advanced |
| 24 | Brentuximab Vedotin/ | 87 | Advanced |
| 25 | Gemtuzumab/ | 91 | Advanced |
| 26 | Inotuzumab Ozogamicin/ | 37 | Advanced |
| 27 | ((antineoplas* or anti-neoplas*) adj6 immun*).mp,kw. | 2946 | Advanced |
| 28 | ((antincancer* or anti-cancer*) adj6 immun*).mp,kw. | 112 | Advanced |
| 29 | ((antitumo?r* or anti-tumo?r*) adj6 immun*).mp,kw. | 1365 | Advanced |
| 30 | ((antineoplas* or anti-neoplas*) adj6 mab?).mp,kw. | 5 | Advanced |
| 31 | ((antincancer* or anti-cancer*) adj6 mab?).mp,kw. | 0 | Advanced |
| 32 | ((antitumo?r* or anti-tumo?r*) adj6 mab?).mp,kw. | 24 | Advanced |
| 33 | (antibody adj3 drug? adj3 conjugat*).mp,kw. | 905 | Advanced |
| 34 | trastuzumab emtansine*.mp,kw. | 411 | Advanced |
| 35 | adotrastuzumab*.mp,kw. | 7 | Advanced |
| 36 | (hun901-dm1 or hun901dm1).mp,kw. | 1 | Advanced |
| 37 | kadcyla*.mp,kw. | 29 | Advanced |
| 38 | (pro-132365 or pro132365).mp,kw. | 0 | Advanced |
| 39 | (rg-3502 or rg3502).mp,kw. | 1 | Advanced |
| 40 | (ro-5304020 or ro5304020).mp,kw. | 18 | Advanced |
| 41 | (conjugat* adj3 (dm-1 or dm1)).mp,kw. | 13 | Advanced |
| 42 | (tmab adj3 (dm-1 or dm1)).mp,kw. | 0 | Advanced |
| 43 | (t dm-1 or t dm1).mp,kw. | 385 | Advanced |
| 44 | (tdm-1 or tdm1).mp,kw. | 68 | Advanced |
| 45 | (trastuzumab* adj3 (dm-1 or dm1)).mp,kw. | 271 | Advanced |
| 46 | ujvira*.mp,kw. | 1 | Advanced |
| 47 | (zrc 3256 or zrc3256).mp,kw. | 2 | Advanced |
| 48 | brentuximab vedotin*.mp,kw. | 426 | Advanced |
| 49 | adcetris*.mp,kw. | 28 | Advanced |
| 50 | (cac10-1006 or cac101006).mp,kw. | 0 | Advanced |
| 51 | (cac10-vcmmae* or cac10vcmmae*).mp,kw. | 3 | Advanced |
| 52 | (sgn-35 or sgn35).mp,kw. | 20 | Advanced |
| 53 | trastuzumab deruxtecan*.mp,kw. | 303 | Advanced |
| 54 | famtrastuzumab*.mp,kw. | 1 | Advanced |
| 55 | (ds-8201 or ds8201).mp,kw. | 39 | Advanced |
| 56 | (ds-8201a or ds8201a).mp,kw. | 52 | Advanced |
| 57 | enhertu*.mp,kw. | 9 | Advanced |
| 58 | (who-10516 or who10516).mp,kw. | 0 | Advanced |
| 59 | gemtuzumab ozogamicin*.mp,kw. | 256 | Advanced |
| 60 | (cdp771 or cdp-771).mp,kw. | 0 | Advanced |
| 61 | (cma676 or cma-676).mp,kw. | 2 | Advanced |
| 62 | (hP676 or hP-676).mp,kw. | 2 | Advanced |
| 63 | mylotarg*.mp,kw. | 52 | Advanced |
| 64 | inotuzumab ozogamicin*.mp,kw. | 121 | Advanced |
| 65 | besponsa*.mp,kw. | 5 | Advanced |
| 66 | calicheamicin*.mp,kw. | 76 | Advanced |
| 67 | (cmc544 or cmc-544).mp,kw. | 8 | Advanced |
| 68 | (pf05208773 or pf-05208773).mp,kw. | 4 | Advanced |
| 69 | (pf5208773 or pf-5208773).mp,kw. | 0 | Advanced |
| 70 | (way207294 or way-207294).mp,kw. | 0 | Advanced |
| 71 | or/21-70 | 6538 | Advanced |
| 72 | 20 and 71 | 533 | Advanced |
| 73 | 72 not (book or book article or book note or "book review" or book series article or book series article in press or book series chapter or book series or book series letter or "book series review" or book series short survey or chapter or clinical trial protocol or dissertation thesis or preprint or trial registry record).pt. | 452 | Advanced |

**Clarivate Web of Science Core Collection 1900 to September 24, 2025**

| **#** | **Search** | **Results** | **Type** |
| --- | --- | --- | --- |
| 1 | TS=(nause* OR vomit* OR emesis* OR emetic OR barf* OR bilious* OR (feel* NEAR/3 sick) OR gag OR gagging* OR heaving* OR hyperemesis* OR puke OR puking OR queasy OR queasiness* OR retch OR retching OR "throw* up") | 164,631 | Advanced |
| 2 | TS=(((antineoplas* or "anti-neoplas*") NEAR/6 immun*) OR ((antincancer* or "anti-cancer*") NEAR/6 immun*) OR ((antitumour* or "anti-tumour*") NEAR/6 immun*) OR ((antitumor* or "anti-tumor*") NEAR/6 immun*) OR ((antineoplas* or "anti-neoplas*") NEAR/6 mab) OR ((antincancer* or "anti-cancer*") NEAR/6 mab) OR ((antitumour* or "anti-tumour*") NEAR/6 mab) OR ((antitumor* or "anti-tumor*") NEAR/6 mab) OR (antibody NEAR/3 drug NEAR/3 conjugat*) OR "trastuzumab emtansine*" OR adotrastuzumab* OR "hun901-dm1" OR hun901dm1 OR kadcyla* OR "pro-132365" OR "pro132365" OR "rg-3502" OR rg3502 OR "ro-5304020" OR ro5304020 OR (conjugat* NEAR/3 ("dm-1" or dm1)) OR (tmab NEAR/3 ("dm-1" or dm1)) OR "t dm-1" OR "t dm1" OR "tdm-1" OR "tdm1" OR (trastuzumab* NEAR/3 ("dm-1" or dm1)) OR ujvira* OR "zrc 3256" OR zrc3256 OR "brentuximab vedotin*" OR adcetris* OR "cac10-1006" OR cac101006 OR "cac10-vcmmae*" OR cac10vcmmae* OR "sgn-35" OR sgn35 OR "trastuzumab deruxtecan*" OR famtrastuzumab* OR "ds-8201" OR ds8201 OR "ds-8201a" OR ds8201a OR enhertu* OR "who-10516" OR who10516 OR "gemtuzumab ozogamicin*" OR cdp771 OR "cdp-771" OR cma676 OR "cma-676"OR hP676 OR "hP-676" OR mylotarg* OR "inotuzumab ozogamicin*" OR besponsa* OR calicheamicin* OR cmc544 OR "cmc-544" OR pf05208773 OR "pf-05208773" OR pf5208773 OR "pf-5208773" OR way207294 OR "way-207294") | 80,440 | Advanced |
| 3 | #1 AND #2 | 444 | Advanced |

**Appendix 2.** Detailed Quality Assessment of **2.1** Interventional Non-Randomized Studies **2.2** Interventional Randomized Studies

**2.1**

| Study | Overall | D1 | D1 Notes | D2 | D2 Notes | D3 | D3 Notes | D4 | D4 Notes | D5 | D5 Notes | D6 | D6 Notes | D7 | D7 Notes |
| --- | --- | --- | --- | --- | --- | --- | --- | --- | --- | --- | --- | --- | --- | --- | --- |
| Acar et al 2023 | Serious | Serious | 1.1 SN; 1.4 N | Low | 2.1 N; 2.3 Y; 2.4 N; 2.5 Y | Serious | 3.1 N; 3.3 N; 3.4 N; 3.5 N | Low | 4.1 N; 4.5 Y | Low | 5.1 Y; 5.2 Y; 5.3 PY | Low | 6.1 N; 6.2 Y; 6.3 PN | Serious | 7.1 NI; 7.2 NI; 7.3 NI; 7.4 NI |
| Adkins et al 2025 | Critical | Serious | 1.1 SN; 1.4 N | Low | 2.1 N; 2.3 Y; 2.4 N; 2.5 Y | Serious | 3.1 N; 3.3 Y; 3.5 Y; 3.6 Y; 3.7 Y | Moderate | 4.1 Y; 4.2 N; 4.3 NI; 4.5 WN | Critical | 5.1 Y; 5.2 N; 5.3 NI; 5.4 Y; 5.5 PY; 5.6 SN; 5.11 N | Moderate | 6.1 N; 6.2 Y; 6.3 WY | Serious | 7.1 NI; 7.2 NI; 7.3 NI; 7.4 NI |
| Advani et al 2010 | Serious | Serious | 1.1 SN; 1.4 N | Low | 2.1 N; 2.3 Y; 2.4 N; 2.5 Y | Low | 3.1 N; 3.3 Y; 3.5 N | Moderate | 4.1 Y; 4.2 N; 4.3 N; 4.5 NI | Low | 5.1 Y; 5.2 PY; 5.3 PY | Moderate | 6.1 N; 6.2 Y; 6.3 WY | Serious | 7.1 NI; 7.2 NI; 7.3 NI; 7.4 NI |
| Aggarwal et al 2017 | Critical | Critical | 1.1 SN; 1.4 PY | Serious | 2.1 Y; 2.2 N; 2.4 N | Low | 3.2 N; 3.3 Y; 3.5 N | Serious | 4.1 N; 4.5 SN | Serious | 5.1 Y; 5.2 N; 5.3 NI; 5.4 NI; 5.5 NI; 5.6 NI; 5.11 N | Moderate | 6.1 PN; 6.2 Y; 6.3 WY | Serious | 7.1 NI; 7.2 NI; 7.3 NI; 7.4 NI |
| Almhanna et al 2016 | Serious | Serious | 1.1 SN; 1.4 N | Low | 2.1 N; 2.3 Y; 2.4 N; 2.5 Y | Low | 3.1 N; 3.3 Y; 3.5 N | Moderate | 4.1 Y; 4.2 N; 4.3 N; 4.5 WN | Low | 5.1 Y; 5.2 PY; 5.3 PY | Serious | 6.1 N; 6.2 Y; 6.3 SY | Serious | 7.1 NI; 7.2 NI; 7.3 NI; 7.4 NI |
| Almhanna et al 2017 | Critical | Serious | 1.1 SN; 1.4 N | Low | 2.1 N; 2.3 Y; 2.4 N; 2.5 Y | Low | 3.1 N; 3.3 Y; 3.5 N | Moderate | 4.1 Y; 4.2 N; 4.3 NI; 4.5 WN | Critical | 5.1 Y; 5.2 N; 5.3 NI; 5.4 Y; 5.5 PY; 5.6 SN; 5.11 N | Moderate | 6.1 N; 6.2 Y; 6.3 WY | Moderate | 7.1 NI; 7.2 PN; 7.3 N; 7.4 N |
| Almhanna et al 2017 | Critical | Serious | 1.1 SN; 1.4 N | Low | 2.1 N; 2.3 Y; 2.4 N; 2.5 Y | Serious | 3.1 N; 3.3 Y; 3.5 Y; 3.6 Y; 3.7 Y | Moderate | 4.1 Y; 4.2 N; 4.3 N; 4.5 WN | Critical | 5.1 Y; 5.2 N; 5.3 NI; 5.4 Y; 5.5 PY; 5.6 SN; 5.11 N | Moderate | 6.1 N; 6.2 Y; 6.3 WY | Moderate | 7.1 NI; 7.2 NI; 7.3 NI; 7.4 N |
| Bai et al 2025 | Serious | Serious | 1.1 SN; 1.4 N | Low | 2.1 N; 2.3 PY; 2.4 N; 2.5 Y | Low | 3.1 N; 3.3 PY; 3.5 N | Moderate | 4.1 Y; 4.2 N; 4.3 NI; 4.5 NI | Serious | 5.1 Y; 5.2 N; 5.3 NI; 5.4 PY; 5.5 NI; 5.6 NI; 5.11 N | Moderate | 6.1 N; 6.2 Y; 6.3 WY | Serious | 7.1 NI; 7.2 NI; 7.3 NI; 7.4 NI |
| Baird et al 2017 | Serious | Serious | 1.1 SN; 1.4 N | Low | 2.1 N; 2.3 Y; 2.4 N; 2.5 Y | Low | 3.1 N; 3.3 Y; 3.5 N | Moderate | 4.1 Y; 4.2 N; 4.3 N; 4.5 WN | Low | 5.1 Y; 5.2 N; 5.3 NI; 5.4 NI; 5.5 N | Moderate | 6.1 N; 6.2 Y; 6.3 WY | Serious | 7.1 NI; 7.2 NI; 7.3 NI; 7.4 NI |
| Baird et al 2025 | Critical | Serious | 1.1 SN; 1.4 N | Low | 2.1 N; 2.3 Y; 2.4 N; 2.5 Y | Serious | 3.1 N; 3.3 Y; 3.5 Y; 3.6 PY; 3.7 Y | Serious | 4.1 Y; 4.2 N; 4.3 NI; 4.5 SN | Critical | 5.1 Y; 5.2 N; 5.3 NI; 5.4 Y; 5.5 PY; 5.6 SN; 5.11 N | Moderate | 6.1 N; 6.2 Y; 6.3 WY | Serious | 7.1 NI; 7.2 NI; 7.3 NI; 7.4 NI |
| Bardia et al 2021 | Critical | Serious | 1.1 SN; 1.4 N | Low | 2.1 N; 2.3 Y; 2.4 N; 2.5 Y | Low | 3.1 N; 3.3 Y; 3.5 N | Moderate | 4.1 Y; 4.2 N; 4.3 N; 4.5 NI | Critical | 5.1 Y; 5.2 N; 5.3 N; 5.4 N; 5.7 N; 5.10 SN; 5.11 N | Moderate | 6.1 N; 6.2 Y; 6.3 WY | Moderate | 7.1 NI; 7.2 PN; 7.3 NI; 7.4 PN |
| Barta et al 2020 | Critical | Serious | 1.1 SN; 1.4 N | Low | 2.1 N; 2.3 Y; 2.4 N; 2.5 Y | Low | 3.1 N; 3.3 Y; 3.5 N | Serious | 4.1 Y; 4.2 N; 4.3 N; 4.5 SN | Critical | 5.1 Y; 5.2 N; 5.3 PY; 5.4 Y; 5.5 PY; 5.6 SN; 5.11 N | Serious | 6.1 N; 6.2 PY; 6.3 SY | Moderate | 7.1 NI; 7.2 NI; 7.3 NI; 7.4 PN |
| Bieth et al 2023 | Serious | Serious | 1.1 SN; 1.4 N | Low | 2.1 N; 2.3 Y; 2.4 N; 2.5 PY | Low | 3.1 N; 3.3 PY; 3.5 N | Moderate | 4.1 N; 4.5 NI | Serious | 5.1 Y; 5.2 Y; 5.3 NI; 5.4 NI; 5.5 NI; 5.6 NI; 5.11 N | Moderate | 6.1 N; 6.2 PY; 6.3 WY | Serious | 7.1 NI; 7.2 NI; 7.3 NI; 7.4 NI |
| Blotta et al 2023 | Critical | Critical | 1.1 SN; 1.4 Y | Low | 2.1 N; 2.3 Y; 2.4 N; 2.5 PY | Critical | 3.1 N; 3.3 Y; 3.5 Y; 3.6 Y; 3.7 Y | Moderate | 4.1 N; 4.5 NI | Serious | 5.1 Y; 5.2 NI; 5.3 NI; 5.4 NI; 5.5 NI; 5.6 NI; 5.11 N | Moderate | 6.1 N; 6.2 PY; 6.3 WY | Serious | 7.1 NI; 7.2 NI; 7.3 NI; 7.4 NI |
| Buono et al 2023 | Serious | Serious | 1.1 SN; 1.4 N | Low | 2.1 N; 2.3 PY; 2.4 N; 2.5 PY | Moderate | 3.1 N; 3.3 NI; 3.5 N | Moderate | 4.1 N; 4.5 NI | Serious | 5.1 Y; 5.2 PY; 5.3 NI; 5.4 NI; 5.5 NI; 5.6 NI; 5.11 N | Moderate | 6.1 N; 6.2 Y; 6.3 WY | Serious | 7.1 NI; 7.2 NI; 7.3 NI; 7.4 NI |
| Calvo et al 2019 | Critical | Serious | 1.1 SN; 1.4 N | Low | 2.1 N; 2.3 Y; 2.4 N; 2.5 Y | Low | 3.1 N; 3.3 PY; 3.5 N | Moderate | 4.1 Y; 4.2 N; 4.3 N; 4.5 WN | Critical | 5.1 Y; 5.2 PN; 5.3 NI; 5.4 PY; 5.5 PY; 5.6 SN; 5.11 N | Moderate | 6.1 N; 6.2 Y; 6.3 WY | Serious | 7.1 NI; 7.2 NI; 7.3 NI; 7.4 NI |
| Camidge et al 2022 | Serious | Serious | 1.1 SN; 1.4 N | Low | 2.1 N; 2.3 Y; 2.4 N; 2.5 Y | Low | 3.1 N; 3.3 Y; 3.5 N | Low | 4.1 Y; 4.2 N; 4.3 N; 4.5 Y | Low | 5.1 Y; 5.2 Y; 5.3 Y | Low | 6.1 N; 6.2 PN | Low | 7.1 PY |
| Carlo-Stella et al 2022 | Critical | Serious | 1.1 N; 1.4 N; 1.5 N | Low | 2.1 N; 2.3 Y; 2.4 N; 2.5 Y | Low | 3.1 N; 3.3 Y; 3.5 N | Serious | 4.1 Y; 4.2 N; 4.3 N; 4.5 SN | Serious | 5.1 Y; 5.2 PY; 5.3 NI; 5.4 N; 5.7 N; 5.10 NI; 5.11 N | Low | 6.1 N; 6.2 NI; 6.3 PN | Serious | 7.1 NI; 7.2 NI; 7.3 NI; 7.4 NI |
| Chan et al 2003 | Critical | Serious | 1.1 Y; 1.3 N; 1.2 NI | Low | 2.1 N; 2.3 Y; 2.4 N; 2.5 Y | Serious | 3.1 N; 3.3 Y; 3.5 Y; 3.6 Y; 3.7 Y | Moderate | 4.1 Y; 4.2 N; 4.3 N; 4.5 WN | Critical | 5.1 Y; 5.2 Y; 5.3 NI; 5.4 Y; 5.5 NI; 5.6 SN; 5.11 N | Moderate | 6.1 N; 6.2 Y; 6.3 WY | Moderate | 7.1 NI; 7.2 N; 7.3 PN; 7.4 N |
| Chen et al 2017 | Serious | Serious | 1.1 SN; 1.4 N | Low | 2.1 N; 2.3 Y; 2.4 N; 2.5 Y | Low | 3.1 N; 3.3 PY; 3.5 N | Moderate | 4.1 Y; 4.2 N; 4.3 NI; 4.5 NI | Low | 5.1 Y; 5.2 N; 5.3 PY; 5.4 PY; 5.5 N | Moderate | 6.1 N; 6.2 NI; 6.3 WY | Serious | 7.1 NI; 7.2 NI; 7.3 NI; 7.4 NI |
| Chen et al 2024 | Critical | Serious | 1.1 SN; 1.4 N | Low | 2.1 N; 2.3 Y; 2.4 N; 2.5 Y | Low | 3.1 N; 3.3 Y; 3.5 N | Moderate | 4.1 Y; 4.2 N; 4.3 NI; 4.5 NI | Critical | 5.1 Y; 5.2 N; 5.3 NI; 5.4 PY; 5.5 NI; 5.6 SN; 5.11 N | Moderate | 6.1 N; 6.2 Y; 6.3 WY | Serious | 7.1 NI; 7.2 NI; 7.3 NI; 7.4 NI |
| Chen et al 2025 | Serious | Serious | 1.1 SN; 1.4 N | Low | 2.1 N; 2.3 PY; 2.4 N; 2.5 PY | Low | 3.1 N; 3.3 PY; 3.5 N | Moderate | 4.1 N; 4.5 NI | Serious | 5.1 Y; 5.2 NI; 5.3 N; 5.4 NI; 5.5 NI; 5.6 NI; 5.11 N | Moderate | 6.1 N; 6.2 PY; 6.3 WY | Serious | 7.1 NI; 7.2 NI; 7.3 NI; 7.4 NI |
| Cheng et al 2025 | Critical | Serious | 1.1 SN; 1.4 N | Low | 2.1 N; 2.3 Y; 2.4 N; 2.5 Y | Low | 3.1 N; 3.3 Y; 3.5 N | Serious | 4.1 Y; 4.2 N; 4.3 NI; 4.5 SN | Critical | 5.1 Y; 5.2 N; 5.3 NI; 5.4 PY; 5.5 PY; 5.6 SN; 5.11 N | Moderate | 6.1 N; 6.2 Y; 6.3 WY | Serious | 7.1 NI; 7.2 NI; 7.3 NI; 7.4 NI |
| Cohen et al 2016 | Serious | Serious | 1.1 SN; 1.4 N | Low | 2.1 N; 2.3 Y; 2.4 N; 2.5 Y | Low | 3.1 N; 3.3 Y; 3.5 N | Moderate | 4.1 Y; 4.2 N; 4.3 N; 4.5 NI | Low | 5.1 Y; 5.2 Y; 5.3 NI; 5.4 NI; 5.5 N | Moderate | 6.1 N; 6.2 Y; 6.3 WY | Serious | 7.1 NI; 7.2 NI; 7.3 NI; 7.4 NI |
| Cooper et al 2025 | Serious | Serious | 1.1 SN; 1.4 N | Low | 2.1 N; 2.3 Y; 2.4 N; 2.5 Y | Low | 3.1 N; 3.3 PY; 3.5 N | Low | 4.1 Y; 4.2 N; 4.3 NI; 4.5 PY | Serious | 5.1 PY; 5.2 PY; 5.3 NI; 5.4 NI; 5.5 NI; 5.6 NI; 5.11 PN | Moderate | 6.1 N; 6.2 Y; 6.3 WY | Serious | 7.1 NI; 7.2 NI; 7.3 NI; 7.4 NI |
| Cortes et al 2017 | Serious | Serious | 1.1 SN; 1.4 N | Low | 2.1 N; 2.3 Y; 2.4 N; 2.5 Y | Low | 3.1 N; 3.3 PY; 3.5 N | Moderate | 4.1 Y; 4.2 N; 4.3 NI; 4.5 NI | Serious | 5.1 Y; 5.2 NI; 5.3 NI; 5.4 NI; 5.5 NI; 5.6 NI; 5.11 N | Moderate | 6.1 N; 6.2 Y; 6.3 WY | Serious | 7.1 NI; 7.2 NI; 7.3 NI; 7.4 NI |
| Coveler et al 2016 | Critical | Serious | 1.1 SN; 1.4 N | Low | 2.1 N; 2.3 Y; 2.4 N; 2.5 Y | Low | 3.1 N; 3.3 Y; 3.5 N | Moderate | 4.1 Y; 4.2 N; 4.3 N; 4.5 NI | Critical | 5.1 Y; 5.2 N; 5.3 PN; 5.4 N; 5.7 N; 5.10 SN; 5.11 N | Serious | 6.1 N; 6.2 Y; 6.3 SY | Serious | 7.1 NI; 7.2 NI; 7.3 NI; 7.4 NI |
| Coward et al 2025 | Serious | Serious | 1.1 SN; 1.4 N | Low | 2.1 N; 2.3 Y; 2.4 N; 2.5 Y | Low | 3.1 N; 3.3 Y; 3.5 N | Moderate | 4.1 Y; 4.2 N; 4.3 NI; 4.5 NI | Serious | 5.1 Y; 5.2 N; 5.3 NI; 5.4 NI; 5.5 NI; 5.6 NI; 5.11 N | Moderate | 6.1 PN; 6.2 Y; 6.3 WY | Serious | 7.1 NI; 7.2 NI; 7.3 NI; 7.4 NI |
| Danila et al 2019 | Serious | Serious | 1.1 SN; 1.4 N | Low | 2.1 N; 2.3 Y; 2.4 N; 2.5 Y | Low | 3.1 N; 3.3 Y; 3.5 N | Low | 4.1 Y; 4.2 N; 4.3 N; 4.5 PY | Low | 5.1 Y; 5.2 PY; 5.3 PY | Serious | 6.1 Y | Serious | 7.1 NI; 7.2 NI; 7.3 NI; 7.4 NI |
| Daver et al 2016 | Serious | Serious | 1.1 SN; 1.4 N | Low | 2.1 N; 2.3 Y; 2.4 N; 2.5 Y | Low | 3.1 N; 3.3 Y; 3.5 N | Low | 4.1 Y; 4.2 N; 4.3 NI; 4.5 Y | Serious | 5.1 Y; 5.2 Y; 5.3 PN; 5.4 NI; 5.5 NI; 5.6 NI; 5.11 N | Low | 6.1 N; 6.2 Y; 6.3 N | Moderate | 7.1 NI; 7.2 NI; 7.3 NI; 7.4 N |
| Daver et al 2019 | Critical | Serious | 1.1 SN; 1.4 N | Low | 2.1 N; 2.3 Y; 2.4 N; 2.5 Y | Low | 3.1 N; 3.3 PY; 3.5 N | Moderate | 4.1 Y; 4.2 N; 4.3 N; 4.5 WN | Serious | 5.1 Y; 5.2 Y; 5.3 N; 5.4 PY; 5.5 NI; 5.6 NI; 5.11 N | Serious | 6.1 Y | Serious | 7.1 NI; 7.2 NI; 7.3 NI; 7.4 NI |
| Daver et al 2023 | Critical | Serious | 1.1 SN; 1.4 N | Low | 2.1 N; 2.3 Y; 2.4 N; 2.5 Y | Low | 3.1 N; 3.3 Y; 3.5 N | Low | 4.1 Y; 4.2 N; 4.3 N; 4.5 Y | Critical | 5.1 Y; 5.2 Y; 5.3 N; 5.4 N; 5.7 N; 5.10 SN; 5.11 N | Moderate | 6.1 N; 6.2 Y; 6.3 WY | Serious | 7.1 NI; 7.2 NI; 7.3 NI; 7.4 NI |
| de Bono et al 2019 | Critical | Serious | 1.1 SN; 1.4 N | Low | 2.1 N; 2.3 Y; 2.4 N; 2.5 Y | Low | 3.1 N; 3.3 Y; 3.5 N | Low | 4.1 Y; 4.2 N; 4.3 N; 4.5 Y | Critical | 5.1 Y; 5.2 N; 5.3 PY; 5.4 N; 5.7 N; 5.10 SN; 5.11 N | Serious | 6.1 N; 6.2 Y; 6.3 SY | Moderate | 7.1 NI; 7.2 PN; 7.3 PN; 7.4 N |
| De Miguel et al 2024 | Serious | Serious | 1.1 SN; 1.4 N | Low | 2.1 N; 2.3 Y; 2.4 N; 2.5 Y | Low | 3.1 N; 3.3 Y; 3.5 N | Low | 4.1 Y; 4.2 N; 4.3 N; 4.5 Y | Serious | 5.1 Y; 5.2 Y; 5.3 NI; 5.4 N; 5.7 PY; 5.8 PN | Moderate | 6.1 PN; 6.2 PY; 6.3 WY | Serious | 7.1 NI; 7.2 NI; 7.3 NI; 7.4 NI |
| Demetri et al 2021 | Critical | Serious | 1.1 SN; 1.4 N | Low | 2.1 N; 2.3 Y; 2.4 N; 2.5 Y | Low | 3.1 N; 3.3 Y; 3.5 N | Serious | 4.1 Y; 4.2 N; 4.3 N; 4.5 SN | Critical | 5.1 Y; 5.2 N; 5.3 NI; 5.4 N; 5.7 N; 5.10 SN; 5.11 N | Moderate | 6.1 N; 6.2 Y; 6.3 WY | Moderate | 7.1 NI; 7.2 NI; 7.3 N; 7.4 NI |
| Dho et al 2024 | Serious | Serious | 1.1 SN; 1.4 N | Low | 2.1 N; 2.3 PY; 2.4 N; 2.5 PY | Low | 3.1 N; 3.3 PY; 3.5 N | Moderate | 4.1 N; 4.5 NI | Serious | 5.1 Y; 5.2 PY; 5.3 NI; 5.4 NI; 5.5 NI; 5.6 NI; 5.11 N | Moderate | 6.1 N; 6.2 Y; 6.3 WY | Serious | 7.1 NI; 7.2 NI; 7.3 NI; 7.4 NI |
| Diefenbach et al 2021 | Serious | Serious | 1.1 SN; 1.4 N | Low | 2.1 N; 2.3 Y; 2.4 N; 2.5 Y | Low | 3.1 N; 3.3 Y; 3.5 N | Moderate | 4.1 Y; 4.2 N; 4.3 NI; 4.5 NI | Serious | 5.1 Y; 5.2 Y; 5.3 NI; 5.4 NI; 5.5 NI; 5.6 NI; 5.11 N | Moderate | 6.1 N; 6.2 Y; 6.3 WY | Serious | 7.1 NI; 7.2 NI; 7.3 NI; 7.4 NI |
| Doi et al 2022 | Serious | Serious | 1.1 SN; 1.4 N | Low | 2.1 N; 2.3 Y; 2.4 N; 2.5 Y | Serious | 3.1 N; 3.3 Y; 3.5 Y; 3.6 Y; 3.7 Y | Moderate | 4.1 Y; 4.2 N; 4.3 NI; 4.5 NI | Low | 5.1 Y; 5.2 N; 5.3 NI; 5.4 Y; 5.5 PN | Moderate | 6.1 PN; 6.2 PY; 6.3 WY | Serious | 7.1 NI; 7.2 NI; 7.3 NI; 7.4 NI |
| Domingo Domenech et al 2021 | Critical | Critical | 1.1 SN; 1.4 PY | Serious | 2.1 Y; 2.2 N; 2.4 N | Moderate | 3.2 N; 3.3 NI; 3.5 N | Moderate | 4.1 N; 4.5 NI | Serious | 5.1 Y; 5.2 N; 5.3 NI; 5.4 Y; 5.5 PY; 5.6 NI; 5.11 N | Moderate | 6.1 NI; 6.2 NI; 6.3 N | Serious | 7.1 NI; 7.2 NI; 7.3 NI; 7.4 NI |
| Duvic et al 2015 | Critical | Serious | 1.1 SN; 1.4 N | Low | 2.1 N; 2.3 Y; 2.4 N; 2.5 Y | Serious | 3.1 N; 3.3 Y; 3.5 Y; 3.6 Y; 3.7 Y | Serious | 4.1 Y; 4.2 N; 4.3 N; 4.5 SN | Critical | 5.1 Y; 5.2 N; 5.3 NI; 5.4 Y; 5.5 Y; 5.6 SN; 5.11 N | Serious | 6.1 N; 6.2 Y; 6.3 SY | Serious | 7.1 NI; 7.2 NI; 7.3 NI; 7.4 NI |
| Escriva-de-Romani et al 2023 | Serious | Serious | 1.1 SN; 1.4 N | Low | 2.1 N; 2.3 Y; 2.4 N; 2.5 Y | Low | 3.1 N; 3.3 Y; 3.5 N | Moderate | 4.1 Y; 4.2 N; 4.3 N; 4.5 NI | Serious | 5.1 Y; 5.2 NI; 5.3 NI; 5.4 NI; 5.5 NI; 5.6 NI; 5.11 PN | Moderate | 6.1 N; 6.2 Y; 6.3 WY | Serious | 7.1 NI; 7.2 NI; 7.3 NI; 7.4 NI |
| Fanale et al 2012 | Critical | Serious | 1.1 SN; 1.4 N | Low | 2.1 N; 2.3 Y; 2.4 N; 2.5 Y | Serious | 3.1 N; 3.3 Y; 3.5 Y; 3.6 PY; 3.7 Y | Moderate | 4.1 Y; 4.2 N; 4.3 N; 4.5 WN | Low | 5.1 Y; 5.2 Y; 5.3 PY | Serious | 6.1 N; 6.2 Y; 6.3 SY | Serious | 7.1 NI; 7.2 NI; 7.3 NI; 7.4 NI |
| Forero-Torres et al 2015 | Serious | Serious | 1.1 SN; 1.4 N | Low | 2.1 N; 2.3 Y; 2.4 N; 2.5 Y | Low | 3.1 N; 3.3 Y; 3.5 N | Low | 4.1 Y; 4.2 N; 4.3 N; 4.5 PY | Low | 5.1 Y; 5.2 Y; 5.3 Y | Moderate | 6.1 N; 6.2 Y; 6.3 WY | Moderate | 7.1 NI; 7.2 NI; 7.3 NI; 7.4 N |
| Funt et al 2025 | Critical | Serious | 1.1 SN; 1.4 N | Low | 2.1 N; 2.3 Y; 2.4 N; 2.5 Y | Serious | 3.1 N; 3.3 Y; 3.5 Y; 3.6 PY; 3.7 PY | Serious | 4.1 Y; 4.2 N; 4.3 NI; 4.5 SN | Serious | 5.1 Y; 5.2 N; 5.3 NI; 5.4 Y; 5.5 NI; 5.6 NI; 5.11 N | Moderate | 6.1 N; 6.2 Y; 6.3 WY | Serious | 7.1 NI; 7.2 NI; 7.3 NI; 7.4 NI |
| Galsky et al 2008 | Serious | Serious | 1.1 SN; 1.4 N | Low | 2.1 N; 2.3 Y; 2.4 N; 2.5 Y | Low | 3.1 N; 3.3 Y; 3.5 N | Moderate | 4.1 Y; 4.2 N; 4.3 N; 4.5 NI | Low | 5.1 Y; 5.2 Y; 5.3 PY | Moderate | 6.1 N; 6.2 Y; 6.3 WY | Serious | 7.1 NI; 7.2 NI; 7.3 NI; 7.4 NI |
| Gao et al 2025 | Critical | Serious | 1.1 SN; 1.4 N | Low | 2.1 N; 2.3 Y; 2.4 N; 2.5 Y | Low | 3.1 N; 3.3 PY; 3.5 N | Moderate | 4.1 Y; 4.2 N; 4.3 NI; 4.5 WN | Serious | 5.1 Y; 5.2 Y; 5.3 NI; 5.4 Y; 5.5 PY; 5.6 NI; 5.11 N | Serious | 6.1 NI; 6.2 PY; 6.3 SY | Serious | 7.1 NI; 7.2 NI; 7.3 NI; 7.4 NI |
| Garrido-Laguna et al 2019 | Critical | Serious | 1.1 SN; 1.4 N | Low | 2.1 N; 2.3 Y; 2.4 N; 2.5 Y | Low | 3.1 N; 3.3 Y; 3.5 N | Low | 4.1 Y; 4.2 N; 4.3 N; 4.5 Y | Critical | 5.1 Y; 5.2 N; 5.3 PY; 5.4 N; 5.7 N; 5.10 SN; 5.11 N | Serious | 6.1 PY | Moderate | 7.1 NI; 7.2 N; 7.3 NI; 7.4 NI |
| Geller et al 2020 | Moderate | Low | 1.1 Y; 1.3 N; 1.2 Y; 1.4 N | Low | 2.1 N; 2.3 Y; 2.4 N; 2.5 Y | Low | 3.1 N; 3.3 Y; 3.5 N | Moderate | 4.1 Y; 4.2 N; 4.3 N; 4.5 WN | Low | 5.1 Y; 5.2 Y; 5.3 N; 5.4 Y; 5.5 PN | Moderate | 6.1 N; 6.2 Y; 6.3 WY | Moderate | 7.1 NI; 7.2 PN; 7.3 PN; 7.4 PN |
| George et al 2023 | Serious | Serious | 1.1 SN; 1.4 N | Low | 2.1 N; 2.3 Y; 2.4 N; 2.5 Y | Low | 3.1 N; 3.3 Y; 3.5 N | Moderate | 4.1 Y; 4.2 N; 4.3 N; 4.5 NI | Serious | 5.1 Y; 5.2 N; 5.3 N; 5.4 N; 5.7 N; 5.10 NI; 5.11 N | Moderate | 6.1 N; 6.2 Y; 6.3 WY | Moderate | 7.1 NI; 7.2 PN; 7.3 PN; 7.4 NI |
| Gerber et al 2020 | Serious | Serious | 1.1 SN; 1.4 N | Low | 2.1 N; 2.3 Y; 2.4 N; 2.5 Y | Low | 3.1 N; 3.3 Y; 3.5 N | Moderate | 4.1 Y; 4.2 N; 4.3 N; 4.5 WN | Low | 5.1 Y; 5.2 Y; 5.3 NI; 5.4 Y; 5.5 N | Serious | 6.1 N; 6.2 Y; 6.3 SY | Serious | 7.1 NI; 7.2 NI; 7.3 NI; 7.4 NI |
| Gopal et al 2012 | Critical | Serious | 1.1 SN; 1.4 N | Low | 2.1 N; 2.3 Y; 2.4 N; 2.5 Y | Low | 3.1 N; 3.3 Y; 3.5 N | Moderate | 4.1 Y; 4.2 N; 4.3 N; 4.5 WN | Critical | 5.1 Y; 5.2 Y; 5.3 N; 5.4 Y; 5.5 Y; 5.6 SN; 5.11 N | Moderate | 6.1 N; 6.2 Y; 6.3 WY | Moderate | 7.1 NI; 7.2 NI; 7.3 NI; 7.4 PN |
| Hamadani et al 2021 | Critical | Serious | 1.1 SN; 1.4 N | Low | 2.1 N; 2.3 Y; 2.4 N; 2.5 Y | Low | 3.1 N; 3.3 Y; 3.5 N | Moderate | 4.1 Y; 4.2 N; 4.3 N; 4.5 WN | Critical | 5.1 Y; 5.2 Y; 5.3 NI; 5.4 Y; 5.5 PY; 5.6 SN; 5.11 N | Moderate | 6.1 N; 6.2 Y; 6.3 WY | Moderate | 7.1 NI; 7.2 N; 7.3 NI; 7.4 NI |
| Hamilton et al 2020 | Serious | Serious | 1.1 SN; 1.4 N | Low | 2.1 N; 2.3 Y; 2.4 N; 2.5 Y | Low | 3.1 N; 3.3 Y; 3.5 N | Low | 4.1 Y; 4.2 N; 4.3 N; 4.5 Y | Serious | 5.1 Y; 5.2 N; 5.3 NI; 5.4 N; 5.7 N; 5.10 SN; 5.11 PY | Moderate | 6.1 PN; 6.2 Y; 6.3 WY | Serious | 7.1 NI; 7.2 NI; 7.3 NI; 7.4 NI |
| Hamilton et al 2022 | Critical | Serious | 1.1 SN; 1.4 N | Low | 2.1 N; 2.3 Y; 2.4 N; 2.5 Y | Low | 3.1 N; 3.3 Y; 3.5 N | Moderate | 4.1 Y; 4.2 N; 4.3 NI; 4.5 NI | Serious | 5.1 Y; 5.2 N; 5.3 NI; 5.4 Y; 5.5 NI; 5.6 NI; 5.11 N | Serious | 6.1 PN; 6.2 Y; 6.3 SY | Serious | 7.1 NI; 7.2 NI; 7.3 NI; 7.4 NI |
| Hamilton et al 2023 | Serious | Serious | 1.1 SN; 1.4 N | Low | 2.1 N; 2.3 Y; 2.4 N; 2.5 PY | Low | 3.1 N; 3.3 PY; 3.5 N | Moderate | 4.1 Y; 4.2 N; 4.3 N; 4.5 NI | Serious | 5.1 Y; 5.2 NI; 5.3 N; 5.4 NI; 5.5 NI; 5.6 NI; 5.11 N | Moderate | 6.1 N; 6.2 Y; 6.3 WY | Serious | 7.1 NI; 7.2 NI; 7.3 NI; 7.4 NI |
| Hamilton et al 2024 | Critical | Serious | 1.1 SN; 1.4 N | Low | 2.1 N; 2.3 PY; 2.4 N; 2.5 PY | Low | 3.1 N; 3.3 Y; 3.5 N | Moderate | 4.1 Y; 4.2 N; 4.3 NI; 4.5 WN | Serious | 5.1 Y; 5.2 PY; 5.3 NI; 5.4 Y; 5.5 NI; 5.6 NI; 5.11 N | Serious | 6.1 PN; 6.2 PY; 6.3 SY | Serious | 7.1 NI; 7.2 NI; 7.3 NI; 7.4 NI |
| Hamilton et al 2025 | Serious | Serious | 1.1 SN; 1.4 N | Low | 2.1 N; 2.3 Y; 2.4 N; 2.5 Y | Low | 3.1 N; 3.3 PY; 3.5 N | Moderate | 4.1 Y; 4.2 N; 4.3 NI; 4.5 WN | Serious | 5.1 Y; 5.2 N; 5.3 N; 5.4 PY; 5.5 NI; 5.6 NI; 5.11 N | Moderate | 6.1 NI; 6.2 Y; 6.3 WY | Serious | 7.1 NI; 7.2 NI; 7.3 NI; 7.4 NI |
| Hann et al 2019 | Serious | Serious | 1.1 SN; 1.4 N | Low | 2.1 N; 2.3 Y; 2.4 N; 2.5 Y | Moderate | 3.1 N; 3.3 N; 3.4 NI; 3.5 N | Low | 4.1 Y; 4.2 N; 4.3 NI; 4.5 Y | Serious | 5.1 Y; 5.2 PY; 5.3 NI; 5.4 NI; 5.5 NI; 5.6 NI; 5.11 N | Moderate | 6.1 PN; 6.2 Y; 6.3 WY | Serious | 7.1 NI; 7.2 NI; 7.3 NI; 7.4 NI |
| Hanna et al 2024 | Serious | Serious | 1.1 SN; 1.4 N | Low | 2.1 N; 2.3 PY; 2.4 N; 2.5 PY | Low | 3.1 N; 3.3 PY; 3.5 N | Low | 4.1 N; 4.5 PY | Low | 5.1 Y; 5.2 Y; 5.3 Y | Moderate | 6.1 N; 6.2 Y; 6.3 WY | Serious | 7.1 NI; 7.2 NI; 7.3 NI; 7.4 NI |
| Hassan et al 2020 | Critical | Serious | 1.1 SN; 1.4 N | Low | 2.1 N; 2.3 Y; 2.4 N; 2.5 Y | Low | 3.1 N; 3.3 Y; 3.5 N | Serious | 4.1 Y; 4.2 N; 4.3 N; 4.5 SN | Critical | 5.1 Y; 5.2 N; 5.3 N; 5.4 N; 5.7 N; 5.10 SN; 5.11 N | Moderate | 6.1 PN; 6.2 Y; 6.3 WY | Serious | 7.1 NI; 7.2 NI; 7.3 NI; 7.4 NI |
| He et al 2024 | Serious | Serious | 1.1 SN; 1.4 N | Low | 2.1 N; 2.3 PY; 2.4 PN; 2.5 PY | Moderate | 3.1 N; 3.3 NI; 3.5 N | Moderate | 4.1 N; 4.5 NI | Serious | 5.1 Y; 5.2 PY; 5.3 NI; 5.4 PY; 5.5 NI; 5.6 NI; 5.11 N | Moderate | 6.1 PN; 6.2 PY; 6.3 WY | Serious | 7.1 NI; 7.2 NI; 7.3 NI; 7.4 NI |
| Heist et al 2025 | Critical | Serious | 1.1 SN; 1.4 N | Low | 2.1 N; 2.3 Y; 2.4 N; 2.5 Y | Serious | 3.1 N; 3.3 Y; 3.5 Y; 3.6 PY; 3.7 Y | Moderate | 4.1 Y; 4.2 N; 4.3 NI; 4.5 NI | Critical | 5.1 Y; 5.2 N; 5.3 NI; 5.4 Y; 5.5 PY; 5.6 SN; 5.11 N | Serious | 6.1 N; 6.2 PY; 6.3 SY | Serious | 7.1 NI; 7.2 NI; 7.3 NI; 7.4 NI |
| Horinouchi et al 2023 | Serious | Serious | 1.1 SN; 1.4 N | Low | 2.1 N; 2.3 Y; 2.4 N; 2.5 Y | Low | 3.1 N; 3.3 PY; 3.5 N | Serious | 4.1 Y; 4.2 N; 4.3 NI; 4.5 SN | Serious | 5.1 Y; 5.2 N; 5.3 NI; 5.4 Y; 5.5 PY; 5.6 NI; 5.11 N | Low | 6.1 N; 6.2 PN | Moderate | 7.1 NI; 7.2 PN; 7.3 NI; 7.4 NI |
| Horwitz et al 2017 | Critical | Serious | 1.1 SN; 1.4 N | Low | 2.1 N; 2.3 Y; 2.4 N; 2.5 Y | Low | 3.1 N; 3.3 Y; 3.5 N | Serious | 4.1 Y; 4.2 N; 4.3 NI; 4.5 SN | Serious | 5.1 PY; 5.2 N; 5.3 NI; 5.4 NI; 5.5 PY; 5.6 NI; 5.11 N | Moderate | 6.1 PN; 6.2 Y; 6.3 WY | Serious | 7.1 NI; 7.2 NI; 7.3 NI; 7.4 NI |
| Jain et al 2020 | Critical | Serious | 1.1 SN; 1.4 N | Low | 2.1 N; 2.3 Y; 2.4 N; 2.5 Y | Low | 3.1 N; 3.3 Y; 3.5 N | Moderate | 4.1 Y; 4.2 N; 4.3 N; 4.5 NI | Critical | 5.1 Y; 5.2 NI; 5.3 NI; 5.4 N; 5.7 N; 5.10 SN; 5.11 N | Moderate | 6.1 N; 6.2 Y; 6.3 WY | Serious | 7.1 NI; 7.2 NI; 7.3 NI; 7.4 NI |
| Jebbink et al 2023 | Serious | Serious | 1.1 SN; 1.4 N | Low | 2.1 N; 2.3 Y; 2.4 N; 2.5 Y | Low | 3.1 N; 3.3 Y; 3.5 N | Low | 4.1 Y; 4.2 N; 4.3 N; 4.5 Y | Serious | 5.1 Y; 5.2 Y; 5.3 NI; 5.4 N; 5.7 N; 5.10 SN; 5.11 PY | Moderate | 6.1 N; 6.2 Y; 6.3 WY | Low | 7.1 PY |
| Jia et al 2024 | Critical | Serious | 1.1 SN; 1.4 N | Low | 2.1 N; 2.3 Y; 2.4 N; 2.5 PY | Serious | 3.1 N; 3.3 PY; 3.5 Y; 3.6 PY; 3.7 Y | Serious | 4.1 Y; 4.2 N; 4.3 N; 4.5 SN | Critical | 5.1 Y; 5.2 N; 5.3 NI; 5.4 Y; 5.5 PY; 5.6 SN; 5.11 N | Serious | 6.1 NI; 6.2 Y; 6.3 SY | Serious | 7.1 NI; 7.2 NI; 7.3 NI; 7.4 NI |
| Jiang et al 2022 | Critical | Serious | 1.1 SN; 1.4 N | Low | 2.1 N; 2.3 Y; 2.4 N; 2.5 Y | Low | 3.1 N; 3.3 Y; 3.5 N | Serious | 4.1 Y; 4.2 N; 4.3 NI; 4.5 SN | Serious | 5.1 Y; 5.2 N; 5.3 NI; 5.4 Y; 5.5 PY; 5.6 NI; 5.11 N | Moderate | 6.1 N; 6.2 NI; 6.3 WY | Serious | 7.1 NI; 7.2 NI; 7.3 NI; 7.4 NI |
| Jiang et al 2024 | Serious | Serious | 1.1 SN; 1.4 N | Low | 2.1 N; 2.3 Y; 2.4 N; 2.5 Y | Low | 3.1 N; 3.3 Y; 3.5 N | Moderate | 4.1 Y; 4.2 N; 4.3 NI; 4.5 NI | Serious | 5.1 Y; 5.2 N; 5.3 NI; 5.4 NI; 5.5 NI; 5.6 NI; 5.11 N | Moderate | 6.1 PN; 6.2 PY; 6.3 WY | Serious | 7.1 NI; 7.2 NI; 7.3 NI; 7.4 NI |
| Johnson et al 2020 | Serious | Serious | 1.1 SN; 1.4 N | Low | 2.1 N; 2.3 PY; 2.4 N; 2.5 PY | Low | 3.1 N; 3.3 PY; 3.5 N | Moderate | 4.1 Y; 4.2 N; 4.3 NI; 4.5 WN | Serious | 5.1 PY; 5.2 N; 5.3 NI; 5.4 PY; 5.5 NI; 5.6 NI; 5.11 N | Moderate | 6.1 N; 6.2 Y; 6.3 WY | Serious | 7.1 NI; 7.2 NI; 7.3 NI; 7.4 NI |
| Kadowaki et al 2024 | Serious | Serious | 1.1 SN; 1.4 N | Low | 2.1 N; 2.3 Y; 2.4 N; 2.5 Y | Low | 3.1 N; 3.3 Y; 3.5 N | Moderate | 4.1 Y; 4.2 N; 4.3 NI; 4.5 NI | Serious | 5.1 Y; 5.2 NI; 5.3 NI; 5.4 NI; 5.5 NI; 5.6 NI; 5.11 N | Moderate | 6.1 NI; 6.2 Y; 6.3 WY | Serious | 7.1 NI; 7.2 NI; 7.3 NI; 7.4 NI |
| Kambhampati et al 2022 | Critical | Serious | 1.1 SN; 1.4 N | Low | 2.1 N; 2.3 Y; 2.4 N; 2.5 Y | Low | 3.1 N; 3.3 Y; 3.5 N | Moderate | 4.1 Y; 4.2 N; 4.3 NI; 4.5 NI | Critical | 5.1 Y; 5.2 PY; 5.3 NI; 5.4 NI; 5.5 NI; 5.6 SN; 5.11 N | Serious | 6.1 PN; 6.2 Y; 6.3 SY | Serious | 7.1 NI; 7.2 NI; 7.3 NI; 7.4 NI |
| Kantarjian et al 2016 | Critical | Serious | 1.1 SN; 1.4 N | Low | 2.1 N; 2.3 Y; 2.4 N; 2.5 Y | Serious | 3.1 N; 3.3 Y; 3.5 Y; 3.6 Y; 3.7 Y | Serious | 4.1 Y; 4.2 N; 4.3 N; 4.5 SN | Critical | 5.1 Y; 5.2 N; 5.3 N; 5.4 Y; 5.5 Y; 5.6 SN; 5.11 N | Moderate | 6.1 N; 6.2 Y; 6.3 WY | Moderate | 7.1 NI; 7.2 N; 7.3 PN; 7.4 N |
| Kelly et al 2016 | Critical | Serious | 1.1 SN; 1.4 N | Low | 2.1 N; 2.3 Y; 2.4 N; 2.5 Y | Serious | 3.1 N; 3.3 PY; 3.5 Y; 3.6 PY; 3.7 Y | Serious | 4.1 Y; 4.2 NI; 4.3 NI; 4.5 SN | Critical | 5.1 PY; 5.2 N; 5.3 NI; 5.4 Y; 5.5 PY; 5.6 SN; 5.11 N | Moderate | 6.1 PN; 6.2 Y; 6.3 WY | Serious | 7.1 NI; 7.2 NI; 7.3 NI; 7.4 NI |
| Khan et al 2021 | Serious | Serious | 1.1 SN; 1.4 N | Low | 2.1 N; 2.3 Y; 2.4 PN; 2.5 PY | Low | 3.1 N; 3.3 PY; 3.5 N | Low | 4.1 N; 4.5 PY | Low | 5.1 Y; 5.2 PY; 5.3 NI; 5.4 NI; 5.5 PN | Low | 6.1 N; 6.2 Y; 6.3 N | Serious | 7.1 NI; 7.2 NI; 7.3 NI; 7.4 NI |
| Kim et al 2023 | Critical | Serious | 1.1 SN; 1.4 N | Low | 2.1 N; 2.3 Y; 2.4 N; 2.5 Y | Low | 3.1 N; 3.3 Y; 3.5 Y; 3.6 N | Serious | 4.1 Y; 4.2 N; 4.3 N; 4.5 SN | Critical | 5.1 Y; 5.2 N; 5.3 PN; 5.4 Y; 5.5 PY; 5.6 SN; 5.11 N | Serious | 6.1 N; 6.2 Y; 6.3 SY | Moderate | 7.1 NI; 7.2 PN; 7.3 NI; 7.4 NI |
| Konecny et al 2024 | Serious | Serious | 1.1 SN; 1.4 N | Low | 2.1 N; 2.3 Y; 2.4 N; 2.5 PY | Low | 3.1 N; 3.3 PY; 3.5 N | Moderate | 4.1 Y; 4.2 N; 4.3 NI; 4.5 NI | Serious | 5.1 NI; 5.2 N; 5.3 NI; 5.4 Y; 5.5 PY; 5.6 NI; 5.11 N | Moderate | 6.1 PN; 6.2 PY; 6.3 WY | Serious | 7.1 NI; 7.2 NI; 7.3 NI; 7.4 NI |
| Kotecki et al 2023 | Serious | Serious | 1.1 SN; 1.4 N | Low | 2.1 N; 2.3 Y; 2.4 N; 2.5 Y | Low | 3.1 N; 3.3 PY; 3.5 N | Moderate | 4.1 Y; 4.2 N; 4.3 NI; 4.5 NI | Serious | 5.1 Y; 5.2 NI; 5.3 NI; 5.4 NI; 5.5 NI; 5.6 NI; 5.11 N | Moderate | 6.1 NI; 6.2 PY; 6.3 WY | Serious | 7.1 NI; 7.2 NI; 7.3 NI; 7.4 NI |
| Krop et al 2022 | Critical | Low | 1.1 Y; 1.3 N; 1.2 Y; 1.4 N | Low | 2.1 N; 2.3 Y; 2.4 N; 2.5 Y | Low | 3.1 N; 3.3 Y; 3.5 Y; 3.6 N | Moderate | 4.1 Y; 4.2 N; 4.3 N; 4.5 WN | Critical | 5.1 Y; 5.2 N; 5.3 NI; 5.4 Y; 5.5 PY; 5.6 SN; 5.11 N | Low | 6.1 N; 6.2 N | Serious | 7.1 NI; 7.2 NI; 7.3 NI; 7.4 NI |
| Kuang et al 2023 | Critical | Serious | 1.1 SN; 1.4 N | Low | 2.1 N; 2.3 PY; 2.4 PN; 2.5 PY | Serious | 3.1 N; 3.3 NI; 3.5 Y; 3.6 Y; 3.7 Y | Moderate | 4.1 N; 4.5 NI | Serious | 5.1 Y; 5.2 NI; 5.3 NI; 5.4 NI; 5.5 NI; 5.6 NI; 5.11 N | Serious | 6.1 NI; 6.2 Y; 6.3 SY | Serious | 7.1 NI; 7.2 NI; 7.3 NI; 7.4 NI |
| Lasica et al 2023 | Critical | Low | 1.1 Y; 1.3 N; 1.2 Y; 1.4 N | Low | 2.1 N; 2.3 Y; 2.4 N; 2.5 Y | Serious | 3.1 N; 3.3 PY; 3.5 Y; 3.6 Y; 3.7 Y | Serious | 4.1 Y; 4.2 N; 4.3 N; 4.5 SN | Critical | 5.1 Y; 5.2 N; 5.3 NI; 5.4 PY; 5.5 PY; 5.6 SN; 5.11 N | Serious | 6.1 N; 6.2 Y; 6.3 SY | Serious | 7.1 NI; 7.2 NI; 7.3 NI; 7.4 NI |
| Lemech et al 2020 | Serious | Serious | 1.1 SN; 1.4 N | Low | 2.1 N; 2.3 Y; 2.4 N; 2.5 Y | Low | 3.1 N; 3.3 Y; 3.5 N | Moderate | 4.1 Y; 4.2 N; 4.3 N; 4.5 NI | Low | 5.1 Y; 5.2 Y; 5.3 N; 5.4 NI; 5.5 N | Moderate | 6.1 N; 6.2 Y; 6.3 WY | Serious | 7.1 NI; 7.2 NI; 7.3 NI; 7.4 NI |
| Lemech et al 2023 | Critical | Serious | 1.1 SN; 1.4 N | Low | 2.1 N; 2.3 Y; 2.4 N; 2.5 Y | Moderate | 3.1 N; 3.3 Y; 3.5 Y; 3.6 NI | Moderate | 4.1 Y; 4.2 N; 4.3 NI; 4.5 WN | Critical | 5.1 Y; 5.2 NI; 5.3 NI; 5.4 PY; 5.5 NI; 5.6 SN; 5.11 N | Moderate | 6.1 NI; 6.2 PY; 6.3 WY | Serious | 7.1 NI; 7.2 NI; 7.3 NI; 7.4 NI |
| Lentz et al 2025 | Serious | Serious | 1.1 SN; 1.4 N | Low | 2.1 N; 2.3 Y; 2.4 N; 2.5 PY | Low | 3.1 N; 3.3 Y; 3.5 N | Low | 4.1 Y; 4.2 N; 4.3 NI; 4.5 PY | Serious | 5.1 Y; 5.2 Y; 5.3 NI; 5.4 NI; 5.5 NI; 5.6 NI; 5.11 N | Moderate | 6.1 PN; 6.2 Y; 6.3 WY | Serious | 7.1 NI; 7.2 NI; 7.3 NI; 7.4 NI |
| Li et al 2024 | Serious | Serious | 1.1 SN; 1.4 N | Low | 2.1 N; 2.3 PY; 2.4 N; 2.5 PY | Moderate | 3.1 N; 3.3 NI; 3.5 N | Low | 4.1 N; 4.5 PY | Serious | 5.1 Y; 5.2 Y; 5.3 NI; 5.4 NI; 5.5 NI; 5.6 NI; 5.11 N | Moderate | 6.1 N; 6.2 NI; 6.3 WY | Serious | 7.1 NI; 7.2 NI; 7.3 NI; 7.4 NI |
| Lin et al 2025 | Serious | Serious | 1.1 SN; 1.4 N | Low | 2.1 N; 2.3 Y; 2.4 PN; 2.5 Y | Low | 3.1 N; 3.3 PY; 3.5 N | Moderate | 4.1 Y; 4.2 N; 4.3 NI; 4.5 NI | Serious | 5.1 NI; 5.2 N; 5.3 NI; 5.4 PY; 5.5 PY; 5.6 NI; 5.11 N | Moderate | 6.1 N; 6.2 Y; 6.3 WY | Serious | 7.1 NI; 7.2 NI; 7.3 NI; 7.4 PY |
| Liu et al 2016 | Critical | Serious | 1.1 SN; 1.4 N | Low | 2.1 N; 2.3 Y; 2.4 N; 2.5 Y | Low | 3.1 N; 3.3 Y; 3.5 N | Low | 4.1 Y; 4.2 N; 4.3 N; 4.5 Y | Critical | 5.1 Y; 5.2 N; 5.3 N; 5.4 N; 5.7 PN; 5.10 SN; 5.11 N | Serious | 6.1 PY | Serious | 7.1 NI; 7.2 NI; 7.3 NI; 7.4 NI |
| Liu et al 2021 | Serious | Serious | 1.1 SN; 1.4 N | Low | 2.1 N; 2.3 Y; 2.4 N; 2.5 Y | Low | 3.1 N; 3.3 Y; 3.5 N | Low | 4.1 Y; 4.2 N; 4.3 N; 4.5 Y | Low | 5.1 Y; 5.2 PY; 5.3 PY | Serious | 6.1 N; 6.2 Y; 6.3 SY | Serious | 7.1 NI; 7.2 NI; 7.3 NI; 7.4 NI |
| Liu et al 2024 | Critical | Serious | 1.1 SN; 1.4 N | Low | 2.1 N; 2.3 Y; 2.4 N; 2.5 Y | Low | 3.1 N; 3.3 Y; 3.5 N | Serious | 4.1 Y; 4.2 N; 4.3 NI; 4.5 SN | Serious | 5.1 Y; 5.2 NI; 5.3 NI; 5.4 PY; 5.5 PY; 5.6 NI; 5.11 N | Moderate | 6.1 N; 6.2 Y; 6.3 WY | Serious | 7.1 NI; 7.2 NI; 7.3 NI; 7.4 NI |
| Liu et al 2024 | Critical | Serious | 1.1 SN; 1.4 N | Low | 2.1 N; 2.3 Y; 2.4 N; 2.5 Y | Moderate | 3.1 N; 3.3 PY; 3.5 Y; 3.6 NI | Serious | 4.1 Y; 4.2 N; 4.3 NI; 4.5 SN | Critical | 5.1 Y; 5.2 N; 5.3 NI; 5.4 Y; 5.5 PY; 5.6 SN; 5.11 N | Moderate | 6.1 N; 6.2 NI; 6.3 WY | Serious | 7.1 NI; 7.2 NI; 7.3 NI; 7.4 NI |
| Liu et al 2025 | Critical | Serious | 1.1 SN; 1.4 N | Low | 2.1 N; 2.3 PY; 2.4 PN; 2.5 PY | Moderate | 3.1 N; 3.3 NI; 3.5 N | Serious | 4.1 N; 4.5 SN | Serious | 5.1 Y; 5.2 PY; 5.3 NI; 5.4 PY; 5.5 NI; 5.6 NI; 5.11 N | Moderate | 6.1 N; 6.2 PY; 6.3 WY | Serious | 7.1 NI; 7.2 NI; 7.3 NI; 7.4 NI |
| Liu et al 2025 | Serious | Serious | 1.1 SN; 1.4 N | Low | 2.1 N; 2.3 Y; 2.4 N; 2.5 Y | Moderate | 3.1 N; 3.3 NI; 3.5 N | Low | 4.1 Y; 4.2 N; 4.3 N; 4.5 PY | Serious | 5.1 Y; 5.2 NI; 5.3 NI; 5.4 NI; 5.5 NI; 5.6 NI; 5.11 N | Moderate | 6.1 N; 6.2 PY; 6.3 WY | Serious | 7.1 NI; 7.2 NI; 7.3 NI; 7.4 NI |
| Locatelli et al 2018 | Serious | Serious | 1.1 SN; 1.4 N | Low | 2.1 N; 2.3 Y; 2.4 N; 2.5 Y | Low | 3.1 N; 3.3 Y; 3.5 N | Moderate | 4.1 Y; 4.2 N; 4.3 N; 4.5 WN | Low | 5.1 Y; 5.2 Y; 5.3 PY | Moderate | 6.1 N; 6.2 NI; 6.3 WY | Moderate | 7.1 NI; 7.2 N; 7.3 N; 7.4 NI |
| Lu et al 2024 | Critical | Serious | 1.1 SN; 1.4 N | Serious | 2.1 Y; 2.2 N; 2.4 N | Low | 3.2 N; 3.3 PY; 3.5 N | Serious | 4.1 Y; 4.2 N; 4.3 NI; 4.5 SN | Critical | 5.1 Y; 5.2 N; 5.3 NI; 5.4 Y; 5.5 PY; 5.6 SN; 5.11 N | Moderate | 6.1 N; 6.2 PY; 6.3 WY | Serious | 7.1 NI; 7.2 NI; 7.3 NI; 7.4 NI |
| Ma et al 2024 | Critical | Serious | 1.1 SN; 1.4 N | Low | 2.1 N; 2.3 Y; 2.4 N; 2.5 Y | Serious | 3.1 N; 3.3 PY; 3.5 Y; 3.6 PY; 3.7 PY | Serious | 4.1 Y; 4.2 N; 4.3 NI; 4.5 SN | Critical | 5.1 Y; 5.2 N; 5.3 NI; 5.4 Y; 5.5 PY; 5.6 SN; 5.11 N | Moderate | 6.1 N; 6.2 NI; 6.3 WY | Serious | 7.1 NI; 7.2 NI; 7.3 NI; 7.4 NI |
| Maitland et al 2021 | Serious | Serious | 1.1 SN; 1.4 N | Low | 2.1 N; 2.3 Y; 2.4 N; 2.5 Y | Low | 3.1 N; 3.3 Y; 3.5 N | Low | 4.1 Y; 4.2 N; 4.3 N; 4.5 PY | Serious | 5.1 Y; 5.2 NI; 5.3 Y; 5.4 N; 5.7 N; 5.10 NI; 5.11 N | Serious | 6.1 Y | Moderate | 7.1 NI; 7.2 PN; 7.3 NI; 7.4 NI |
| Mascolo et al 2020 | Critical | Serious | 1.1 SN; 1.4 N | Low | 2.1 N; 2.3 PY; 2.4 PN; 2.5 PY | Critical | 3.1 N; 3.3 NI; 3.5 Y; 3.6 Y; 3.7 Y | Moderate | 4.1 N; 4.5 NI | Critical | 5.1 Y; 5.2 Y; 5.3 NI; 5.4 N; 5.7 N; 5.10 SN; 5.11 N | Serious | 6.1 Y | Serious | 7.1 NI; 7.2 NI; 7.3 NI; 7.4 NI |
| Massard et al 2019 | Serious | Serious | 1.1 SN; 1.4 N | Low | 2.1 N; 2.3 Y; 2.4 N; 2.5 Y | Low | 3.1 N; 3.3 Y; 3.5 N | Low | 4.1 Y; 4.2 N; 4.3 N; 4.5 Y | Serious | 5.1 Y; 5.2 N; 5.3 PY; 5.4 N; 5.7 N; 5.10 NI; 5.11 N | Low | 6.1 N; 6.2 Y; 6.3 PN | Serious | 7.1 NI; 7.2 NI; 7.3 NI; 7.4 NI |
| McGregor et al 2020 | Serious | Serious | 1.1 SN; 1.4 N | Low | 2.1 N; 2.3 Y; 2.4 N; 2.5 Y | Low | 3.1 N; 3.3 Y; 3.5 N | Low | 4.1 Y; 4.2 N; 4.3 N; 4.5 Y | Low | 5.1 Y; 5.2 Y; 5.3 Y | Moderate | 6.1 N; 6.2 Y; 6.3 WY | Moderate | 7.1 NI; 7.2 PN; 7.3 NI; 7.4 PN |
| Mei et al 2024 | Serious | Serious | 1.1 SN; 1.4 N | Low | 2.1 N; 2.3 Y; 2.4 N; 2.5 Y | Serious | 3.1 N; 3.3 Y; 3.5 Y; 3.6 Y; 3.7 PY | Serious | 4.1 Y; 4.2 N; 4.3 N; 4.5 SN | Low | 5.1 Y; 5.2 Y; 5.3 Y | Moderate | 6.1 N; 6.2 Y; 6.3 WY | Low | 7.1 PY |
| Meric Bernstam et al 2025 | Critical | Serious | 1.1 SN; 1.4 N | Low | 2.1 N; 2.3 Y; 2.4 N; 2.5 Y | Low | 3.1 N; 3.3 Y; 3.5 N | Low | 4.1 Y; 4.2 N; 4.3 N; 4.5 PY | Critical | 5.1 Y; 5.2 N; 5.3 Y; 5.4 N; 5.7 N; 5.10 SN; 5.11 N | Low | 6.1 N; 6.2 N | Moderate | 7.1 NI; 7.2 PN; 7.3 N; 7.4 N |
| Meric-Bernstam et al 2024 | Serious | Serious | 1.1 SN; 1.4 N | Low | 2.1 N; 2.3 Y; 2.4 N; 2.5 Y | Low | 3.1 N; 3.3 Y; 3.5 N | Moderate | 4.1 Y; 4.2 N; 4.3 NI; 4.5 NI | Serious | 5.1 Y; 5.2 NI; 5.3 NI; 5.4 NI; 5.5 NI; 5.6 NI; 5.11 N | Moderate | 6.1 N; 6.2 Y; 6.3 WY | Serious | 7.1 NI; 7.2 NI; 7.3 NI; 7.4 NI |
| Meric-Bernstam et al 2025 | Critical | Serious | 1.1 Y; 1.3 N; 1.2 NI | Low | 2.1 N; 2.3 Y; 2.4 N; 2.5 Y | Low | 3.1 N; 3.3 Y; 3.5 N | Low | 4.1 Y; 4.2 N; 4.3 NI; 4.5 Y | Critical | 5.1 Y; 5.2 N; 5.3 NI; 5.4 N; 5.7 N; 5.10 SN; 5.11 N | Low | 6.1 N; 6.2 N | Serious | 7.1 NI; 7.2 NI; 7.3 NI; 7.4 NI |
| Metheny et al 2024 | Serious | Serious | 1.1 SN; 1.4 N | Serious | 2.1 Y; 2.2 N; 2.4 N | Serious | 3.2 PY; 3.3 N; 3.4 PN; 3.5 N | Low | 4.1 Y; 4.2 N; 4.3 N; 4.5 Y | Low | 5.1 Y; 5.2 Y; 5.3 Y | Low | 6.1 N; 6.2 Y; 6.3 PN | Low | 7.1 PY |
| Michel et al 2025 | Critical | Serious | 1.1 SN; 1.4 N | Low | 2.1 N; 2.3 Y; 2.4 N; 2.5 Y | Low | 3.1 N; 3.3 Y; 3.5 N | Low | 4.1 Y; 4.2 N; 4.3 N; 4.5 Y | Critical | 5.1 Y; 5.2 N; 5.3 PY; 5.4 N; 5.7 N; 5.10 SN; 5.11 N | Moderate | 6.1 N; 6.2 Y; 6.3 WY | Moderate | 7.1 NI; 7.2 PN; 7.3 NI; 7.4 NI |
| Moore et al 2017 | Critical | Serious | 1.1 SN; 1.4 N | Low | 2.1 N; 2.3 Y; 2.4 N; 2.5 Y | Low | 3.1 N; 3.3 Y; 3.5 N | Low | 4.1 Y; 4.2 N; 4.3 N; 4.5 Y | Critical | 5.1 Y; 5.2 Y; 5.3 NI; 5.4 N; 5.7 N; 5.10 SN; 5.11 N | Moderate | 6.1 N; 6.2 Y; 6.3 WY | Serious | 7.1 NI; 7.2 NI; 7.3 NI; 7.4 NI |
| Moore et al 2023 | Critical | Serious | 1.1 SN; 1.4 N | Low | 2.1 N; 2.3 Y; 2.4 N; 2.5 Y | Serious | 3.1 N; 3.3 Y; 3.5 Y; 3.6 PY; 3.7 Y | Moderate | 4.1 Y; 4.2 N; 4.3 NI; 4.5 NI | Serious | 5.1 Y; 5.2 N; 5.3 NI; 5.4 Y; 5.5 PY; 5.6 NI; 5.11 N | Moderate | 6.1 N; 6.2 Y; 6.3 WY | Serious | 7.1 NI; 7.2 NI; 7.3 NI; 7.4 NI |
| Morgensztern et al 2023 | Critical | Serious | 1.1 SN; 1.4 N | Low | 2.1 N; 2.3 Y; 2.4 N; 2.5 Y | Low | 3.1 N; 3.3 Y; 3.5 N | Moderate | 4.1 Y; 4.2 N; 4.3 NI; 4.5 NI | Serious | 5.1 Y; 5.2 PY; 5.3 NI; 5.4 NI; 5.5 NI; 5.6 NI; 5.11 N | Serious | 6.1 N; 6.2 Y; 6.3 SY | Serious | 7.1 NI; 7.2 NI; 7.3 NI; 7.4 NI |
| Munayirji et al 2023 | Critical | Serious | 1.1 SN; 1.4 N | Low | 2.1 N; 2.3 Y; 2.4 N; 2.5 PY | Low | 3.1 N; 3.3 Y; 3.5 N | Low | 4.1 Y; 4.2 N; 4.3 NI; 4.5 PY | Serious | 5.1 Y; 5.2 PY; 5.3 NI; 5.4 NI; 5.5 NI; 5.6 NI; 5.11 N | Serious | 6.1 N; 6.2 PY; 6.3 SY | Serious | 7.1 NI; 7.2 NI; 7.3 NI; 7.4 NI |
| Murakami et al 2019 | Critical | Serious | 1.1 SN; 1.4 N | Moderate | 2.1 N; 2.3 NI; 2.4 PN; 2.5 Y | Low | 3.1 N; 3.3 Y; 3.5 N | Moderate | 4.1 Y; 4.2 N; 4.3 NI; 4.5 NI | Serious | 5.1 Y; 5.2 PY; 5.3 NI; 5.4 NI; 5.5 NI; 5.6 NI; 5.11 N | Serious | 6.1 PN; 6.2 Y; 6.3 SY | Serious | 7.1 NI; 7.2 NI; 7.3 NI; 7.4 NI |
| Nishio et al 2022 | Serious | Serious | 1.1 SN; 1.4 N | Low | 2.1 N; 2.3 Y; 2.4 N; 2.5 Y | Low | 3.1 N; 3.3 PY; 3.5 N | Low | 4.1 Y; 4.2 N; 4.3 NI; 4.5 PY | Low | 5.1 Y; 5.2 Y; 5.3 NI; 5.4 PY; 5.5 N | Serious | 6.1 PN; 6.2 Y; 6.3 SY | Serious | 7.1 NI; 7.2 NI; 7.3 NI; 7.4 NI |
| Occhiogrosso Abelman et al 2025 | Serious | Serious | 1.1 SN; 1.4 N | Low | 2.1 N; 2.3 Y; 2.4 N; 2.5 Y | Low | 3.1 N; 3.3 Y; 3.5 N | Low | 4.1 Y; 4.2 N; 4.3 N; 4.5 Y | Low | 5.1 Y; 5.2 Y; 5.3 NI; 5.4 PY; 5.5 N | Low | 6.1 N; 6.2 PY; 6.3 PN | Moderate | 7.1 NI; 7.2 PN; 7.3 NI; 7.4 NI |
| Oliva et al 2024 | Serious | Serious | 1.1 SN; 1.4 N | Low | 2.1 N; 2.3 PY; 2.4 N; 2.5 Y | Low | 3.1 N; 3.3 Y; 3.5 N | Moderate | 4.1 Y; 4.2 N; 4.3 NI; 4.5 NI | Serious | 5.1 Y; 5.2 NI; 5.3 NI; 5.4 NI; 5.5 NI; 5.6 NI; 5.11 N | Moderate | 6.1 PN; 6.2 Y; 6.3 WY | Serious | 7.1 NI; 7.2 NI; 7.3 NI; 7.4 NI |
| Oliveira et al 2023 | Serious | Serious | 1.1 SN; 1.4 N | Low | 2.1 N; 2.3 Y; 2.4 N; 2.5 Y | Low | 3.1 N; 3.3 Y; 3.5 N | Moderate | 4.1 Y; 4.2 N; 4.3 N; 4.5 WN | Moderate | 5.1 Y; 5.2 Y; 5.3 N; 5.4 Y; 5.5 NI; 5.6 NI; 5.11 PY | Moderate | 6.1 N; 6.2 NI; 6.3 WY | Moderate | 7.1 NI; 7.2 N; 7.3 NI; 7.4 PN |
| Ooki et al 2023 | Critical | Serious | 1.1 SN; 1.4 N | Low | 2.1 N; 2.3 PY; 2.4 PN; 2.5 PY | Low | 3.1 N; 3.3 PY; 3.5 N | Moderate | 4.1 N; 4.5 NI | Serious | 5.1 Y; 5.2 NI; 5.3 NI; 5.4 NI; 5.5 NI; 5.6 NI; 5.11 N | Serious | 6.1 N; 6.2 Y; 6.3 SY | Serious | 7.1 NI; 7.2 NI; 7.3 NI; 7.4 NI |
| Ott et al 2016 | Serious | Serious | 1.1 SN; 1.4 N | Low | 2.1 N; 2.3 Y; 2.4 N; 2.5 Y | Moderate | 3.1 N; 3.3 Y; 3.5 Y; 3.6 NI | Moderate | 4.1 Y; 4.2 N; 4.3 NI; 4.5 WN | Low | 5.1 PY; 5.2 PN; 5.3 NI; 5.4 Y; 5.5 PN | Moderate | 6.1 N; 6.2 Y; 6.3 WY | Moderate | 7.1 NI; 7.2 PN; 7.3 NI; 7.4 NI |
| Parikh et al 2024 | Critical | Serious | 1.1 SN; 1.4 N | Low | 2.1 N; 2.3 Y; 2.4 N; 2.5 PY | Low | 3.1 N; 3.3 PY; 3.5 N | Low | 4.1 N; 4.5 PY | Serious | 5.1 Y; 5.2 PY; 5.3 NI; 5.4 NI; 5.5 NI; 5.6 NI; 5.11 N | Serious | 6.1 N; 6.2 Y; 6.3 SY | Serious | 7.1 NI; 7.2 NI; 7.3 NI; 7.4 NI |
| Parikh et al 2025 | Critical | Serious | 1.1 SN; 1.4 N | Low | 2.1 N; 2.3 Y; 2.4 N; 2.5 PY | Low | 3.1 N; 3.3 Y; 3.5 N | Moderate | 4.1 N; 4.5 NI | Critical | 5.1 PY; 5.2 NI; 5.3 NI; 5.4 N; 5.7 N; 5.10 SN; 5.11 N | Serious | 6.1 N; 6.2 Y; 6.3 SY | Serious | 7.1 NI; 7.2 NI; 7.3 NI; 7.4 NI |
| Park et al 2025 | Serious | Serious | 1.1 SN; 1.4 N | Low | 2.1 N; 2.3 Y; 2.4 N; 2.5 Y | Low | 3.1 N; 3.3 PY; 3.5 N | Low | 4.1 Y; 4.2 N; 4.3 NI; 4.5 PY | Low | 5.1 Y; 5.2 Y; 5.3 NI; 5.4 Y; 5.5 N | Serious | 6.1 PN; 6.2 Y; 6.3 SY | Serious | 7.1 NI; 7.2 NI; 7.3 NI; 7.4 NI |
| Patel et al 2024 | Serious | Serious | 1.1 SN; 1.4 N | Low | 2.1 N; 2.3 Y; 2.4 N; 2.5 PY | Low | 3.1 N; 3.3 Y; 3.5 N | Moderate | 4.1 Y; 4.2 N; 4.3 N; 4.5 NI | Serious | 5.1 Y; 5.2 NI; 5.3 PY; 5.4 NI; 5.5 NI; 5.6 NI; 5.11 N | Moderate | 6.1 PN; 6.2 Y; 6.3 WY | Serious | 7.1 NI; 7.2 NI; 7.3 NI; 7.4 NI |
| Patel et al 2024 | Serious | Serious | 1.1 SN; 1.4 N | Low | 2.1 N; 2.3 Y; 2.4 N; 2.5 Y | Low | 3.1 N; 3.3 Y; 3.5 N | Low | 4.1 Y; 4.2 N; 4.3 N; 4.5 PY | Low | 5.1 Y; 5.2 Y; 5.3 N; 5.4 Y; 5.5 N | Low | 6.1 N; 6.2 NI; 6.3 PN | Serious | 7.1 NI; 7.2 NI; 7.3 NI; 7.4 NI |
| Pemmaraju et al 2021 | Serious | Serious | 1.1 SN; 1.4 N | Low | 2.1 N; 2.3 Y; 2.4 N; 2.5 Y | Low | 3.1 N; 3.3 Y; 3.5 N | Low | 4.1 Y; 4.2 N; 4.3 NI; 4.5 PY | Low | 5.1 PY; 5.2 PY; 5.3 NI; 5.4 NI; 5.5 N | Moderate | 6.1 N; 6.2 Y; 6.3 WY | Serious | 7.1 NI; 7.2 NI; 7.3 NI; 7.4 NI |
| Perez et al 2025 | Critical | Serious | 1.1 SN; 1.4 N | Low | 2.1 N; 2.3 Y; 2.4 N; 2.5 PY | Low | 3.1 N; 3.3 PY; 3.5 N | Serious | 4.1 Y; 4.2 N; 4.3 NI; 4.5 SN | Critical | 5.1 Y; 5.2 N; 5.3 NI; 5.4 Y; 5.5 PY; 5.6 SN; 5.11 N | Moderate | 6.1 N; 6.2 Y; 6.3 WY | Serious | 7.1 NI; 7.2 NI; 7.3 NI; 7.4 NI |
| Petrylak et al 2024 | Critical | Serious | 1.1 SN; 1.4 N | Low | 2.1 N; 2.3 Y; 2.4 N; 2.5 Y | Low | 3.1 N; 3.3 Y; 3.5 N | Low | 4.1 Y; 4.2 N; 4.3 N; 4.5 Y | Critical | 5.1 Y; 5.2 N; 5.3 NI; 5.4 N; 5.7 N; 5.10 SN; 5.11 PN | Moderate | 6.1 N; 6.2 Y; 6.3 WY | Serious | 7.1 NI; 7.2 NI; 7.3 NI; 7.4 NI |
| Phillips et al 2019 | Critical | Serious | 1.1 SN; 1.4 N | Low | 2.1 N; 2.3 Y; 2.4 N; 2.5 Y | Low | 3.1 N; 3.3 Y; 3.5 N | Low | 4.1 Y; 4.2 N; 4.3 NI; 4.5 Y | Critical | 5.1 Y; 5.2 Y; 5.3 N; 5.4 N; 5.7 N; 5.10 SN; 5.11 N | Serious | 6.1 Y | Serious | 7.1 NI; 7.2 NI; 7.3 NI; 7.4 NI |
| Pistilli et al 2024 | Critical | Serious | 1.1 SN; 1.4 N | Low | 2.1 N; 2.3 PY; 2.4 N; 2.5 Y | Low | 3.1 N; 3.3 Y; 3.5 N | Moderate | 4.1 Y; 4.2 N; 4.3 N; 4.5 NI | Serious | 5.1 PY; 5.2 NI; 5.3 NI; 5.4 NI; 5.5 NI; 5.6 NI; 5.11 N | Serious | 6.1 N; 6.2 Y; 6.3 SY | Serious | 7.1 NI; 7.2 NI; 7.3 NI; 7.4 NI |
| Poggio et al 2023 | Critical | Serious | 1.1 SN; 1.4 N | Low | 2.1 N; 2.3 Y; 2.4 N; 2.5 Y | Low | 3.1 N; 3.3 PY; 3.5 N | Low | 4.1 N; 4.5 PY | Serious | 5.1 Y; 5.2 PY; 5.3 N; 5.4 NI; 5.5 NI; 5.6 NI; 5.11 N | Serious | 6.1 N; 6.2 Y; 6.3 SY | Serious | 7.1 NI; 7.2 NI; 7.3 NI; 7.4 NI |
| Preusser et al 2025 | Serious | Serious | 1.1 SN; 1.4 N | Low | 2.1 N; 2.3 Y; 2.4 N; 2.5 Y | Low | 3.1 N; 3.3 Y; 3.5 N | Low | 4.1 Y; 4.2 N; 4.3 N; 4.5 Y | Low | 5.1 Y; 5.2 Y; 5.3 PY | Low | 6.1 N; 6.2 Y; 6.3 N | Low | 7.1 PY |
| Radovich et al 2022 | Critical | Serious | 1.1 SN; 1.4 N | Low | 2.1 N; 2.3 Y; 2.4 N; 2.5 Y | Low | 3.1 N; 3.3 Y; 3.5 N | Low | 4.1 Y; 4.2 N; 4.3 N; 4.5 PY | Critical | 5.1 Y; 5.2 N; 5.3 PY; 5.4 N; 5.7 N; 5.10 SN; 5.11 N | Moderate | 6.1 N; 6.2 Y; 6.3 WY | Moderate | 7.1 NI; 7.2 N; 7.3 NI; 7.4 PN |
| Ramalingam et al 2019 | Critical | Critical | 1.1 N; 1.4 N; 1.5 Y | Low | 2.1 N; 2.3 Y; 2.4 N; 2.5 PY | Low | 3.1 N; 3.3 PY; 3.5 N | Serious | 4.1 N; 4.5 SN | Serious | 5.1 PY; 5.2 NI; 5.3 NI; 5.4 NI; 5.5 NI; 5.6 NI; 5.11 N | Serious | 6.1 Y | Serious | 7.1 NI; 7.2 NI; 7.3 NI; 7.4 NI |
| Ramchandren et al 2016 | Critical | Low | 1.1 Y; 1.3 N; 1.2 Y; 1.4 N | Low | 2.1 N; 2.3 PY; 2.4 N; 2.5 PY | Serious | 3.1 N; 3.3 Y; 3.5 Y; 3.6 Y; 3.7 PY | Serious | 4.1 Y; 4.2 N; 4.3 NI; 4.5 SN | Critical | 5.1 Y; 5.2 N; 5.3 NI; 5.4 Y; 5.5 PY; 5.6 SN; 5.11 N | Moderate | 6.1 N; 6.2 Y; 6.3 WY | Serious | 7.1 NI; 7.2 NI; 7.3 NI; 7.4 NI |
| Ren et al 2025 | Critical | Serious | 1.1 SN; 1.4 N | Low | 2.1 N; 2.3 Y; 2.4 PN; 2.5 PY | Low | 3.1 N; 3.3 PY; 3.5 N | Moderate | 4.1 N; 4.5 NI | Serious | 5.1 Y; 5.2 NI; 5.3 NI; 5.4 NI; 5.5 NI; 5.6 NI; 5.11 N | Serious | 6.1 PY | Serious | 7.1 NI; 7.2 NI; 7.3 NI; 7.4 NI |
| Richardson et al 2020 | Critical | Serious | 1.1 SN; 1.4 N | Serious | 2.1 NI; 2.2 NI; 2.4 NI | Moderate | 3.1 NI; 3.3 PY; 3.5 Y; 3.6 NI | Moderate | 4.1 Y; 4.2 N; 4.3 NI; 4.5 NI | Serious | 5.1 Y; 5.2 NI; 5.3 NI; 5.4 NI; 5.5 NI; 5.6 NI; 5.11 N | Moderate | 6.1 NI; 6.2 PY; 6.3 WY | Serious | 7.1 NI; 7.2 NI; 7.3 NI; 7.4 NI |
| Richter et al 2024 | Serious | Serious | 1.1 SN; 1.4 N | Low | 2.1 N; 2.3 Y; 2.4 N; 2.5 Y | Moderate | 3.1 N; 3.3 Y; 3.5 Y; 3.6 NI | Moderate | 4.1 Y; 4.2 N; 4.3 N; 4.5 WN | Serious | 5.1 Y; 5.2 Y; 5.3 N; 5.4 NI; 5.5 NI; 5.6 NI; 5.11 N | Low | 6.1 N; 6.2 PY; 6.3 PN | Serious | 7.1 NI; 7.2 NI; 7.3 NI; 7.4 NI |
| Rodon et al 2021 | Critical | Serious | 1.1 SN; 1.4 N | Low | 2.1 N; 2.3 Y; 2.4 N; 2.5 Y | Low | 3.1 N; 3.3 Y; 3.5 N | Serious | 4.1 Y; 4.2 N; 4.3 NI; 4.5 SN | Serious | 5.1 Y; 5.2 N; 5.3 NI; 5.4 Y; 5.5 PY; 5.6 NI; 5.11 N | Moderate | 6.1 N; 6.2 Y; 6.3 WY | Serious | 7.1 NI; 7.2 NI; 7.3 NI; 7.4 NI |
| Rodriguez-Rivera et al 2023 | Critical | Serious | 1.1 SN; 1.4 N | Low | 2.1 N; 2.3 PY; 2.4 N; 2.5 PY | Moderate | 3.1 N; 3.3 PY; 3.5 NI | Moderate | 4.1 Y; 4.2 N; 4.3 NI; 4.5 NI | Serious | 5.1 PY; 5.2 NI; 5.3 NI; 5.4 NI; 5.5 NI; 5.6 NI; 5.11 N | Serious | 6.1 PY | Serious | 7.1 NI; 7.2 NI; 7.3 NI; 7.4 NI |
| Rosen et al 2019 | Critical | Serious | 1.1 SN; 1.4 N | Low | 2.1 N; 2.3 Y; 2.4 N; 2.5 Y | Low | 3.1 N; 3.3 Y; 3.5 N | Serious | 4.1 Y; 4.2 N; 4.3 N; 4.5 SN | Critical | 5.1 Y; 5.2 N; 5.3 NI; 5.4 Y; 5.5 PY; 5.6 SN; 5.11 N | Moderate | 6.1 N; 6.2 Y; 6.3 WY | Moderate | 7.1 NI; 7.2 PN; 7.3 NI; 7.4 NI |
| Rosenberg et al 2020 | Serious | Serious | 1.1 SN; 1.4 N | Low | 2.1 N; 2.3 Y; 2.4 N; 2.5 Y | Low | 3.1 N; 3.3 Y; 3.5 N | Low | 4.1 Y; 4.2 N; 4.3 N; 4.5 Y | Low | 5.1 Y; 5.2 PY; 5.3 PY | Moderate | 6.1 N; 6.2 Y; 6.3 WY | Moderate | 7.1 NI; 7.2 NI; 7.3 PN; 7.4 PN |
| Rotow et al 2023 | Serious | Serious | 1.1 SN; 1.4 N | Low | 2.1 N; 2.3 Y; 2.4 N; 2.5 Y | Low | 3.1 N; 3.3 PY; 3.5 N | Moderate | 4.1 Y; 4.2 N; 4.3 NI; 4.5 NI | Moderate | 5.1 Y; 5.2 Y; 5.3 NI; 5.4 Y; 5.5 NI; 5.6 NI; 5.11 Y | Moderate | 6.1 N; 6.2 PY; 6.3 WY | Moderate | 7.1 NI; 7.2 PN; 7.3 NI; 7.4 NI |
| Ruan et al 2025 | Serious | Serious | 1.1 SN; 1.4 N | Low | 2.1 N; 2.3 Y; 2.4 N; 2.5 Y | Low | 3.1 N; 3.3 Y; 3.5 Y; 3.6 N | Low | 4.1 Y; 4.2 N; 4.3 N; 4.5 PY | Low | 5.1 Y; 5.2 PY; 5.3 PY | Moderate | 6.1 N; 6.2 Y; 6.3 WY | Low | 7.1 PY |
| Sandhu et al 2019 | Critical | Serious | 1.1 SN; 1.4 N | Low | 2.1 N; 2.3 Y; 2.4 N; 2.5 Y | Low | 3.1 N; 3.3 Y; 3.5 N | Moderate | 4.1 Y; 4.2 N; 4.3 N; 4.5 NI | Critical | 5.1 Y; 5.2 PY; 5.3 N; 5.4 N; 5.7 N; 5.10 SN; 5.11 N | Moderate | 6.1 N; 6.2 Y; 6.3 WY | Moderate | 7.1 NI; 7.2 NI; 7.3 PN; 7.4 NI |
| Schjesvold et al 2023 | Serious | Serious | 1.1 SN; 1.4 N | Low | 2.1 N; 2.3 Y; 2.4 N; 2.5 Y | Low | 3.1 N; 3.3 Y; 3.5 N | Low | 4.1 Y; 4.2 N; 4.3 N; 4.5 PY | Serious | 5.1 Y; 5.2 Y; 5.3 NI; 5.4 N; 5.7 NI; 5.10 NI; 5.11 N | Moderate | 6.1 N; 6.2 Y; 6.3 WY | Serious | 7.1 NI; 7.2 NI; 7.3 NI; 7.4 NI |
| Schoffski et al 2021 | Serious | Serious | 1.1 SN; 1.4 N | Low | 2.1 N; 2.3 Y; 2.4 N; 2.5 Y | Low | 3.1 N; 3.3 Y; 3.5 N | Moderate | 4.1 Y; 4.2 N; 4.3 N; 4.5 NI | Low | 5.1 Y; 5.2 Y; 5.3 PY | Serious | 6.1 N; 6.2 Y; 6.3 SY | Serious | 7.1 NI; 7.2 NI; 7.3 NI; 7.4 NI |
| Shah et al 2019 | Critical | Serious | 1.1 SN; 1.4 N | Low | 2.1 N; 2.3 Y; 2.4 N; 2.5 Y | Serious | 3.1 N; 3.3 Y; 3.5 Y; 3.6 PY; 3.7 Y | Moderate | 4.1 Y; 4.2 N; 4.3 N; 4.5 NI | Serious | 5.1 Y; 5.2 N; 5.3 NI; 5.4 PY; 5.5 NI; 5.6 NI; 5.11 N | Moderate | 6.1 N; 6.2 Y; 6.3 WY | Serious | 7.1 NI; 7.2 NI; 7.3 NI; 7.4 NI |
| Shapira-Frommer et al 2024 | Serious | Serious | 1.1 SN; 1.4 N | Low | 2.1 N; 2.3 Y; 2.4 N; 2.5 Y | Low | 3.1 N; 3.3 Y; 3.5 N | Moderate | 4.1 Y; 4.2 N; 4.3 NI; 4.5 NI | Serious | 5.1 Y; 5.2 NI; 5.3 NI; 5.4 PY; 5.5 NI; 5.6 NI; 5.11 N | Moderate | 6.1 PN; 6.2 Y; 6.3 WY | Serious | 7.1 NI; 7.2 NI; 7.3 NI; 7.4 NI |
| Shapiro et al 2017 | Critical | Serious | 1.1 SN; 1.4 N | Low | 2.1 N; 2.3 Y; 2.4 N; 2.5 Y | Low | 3.1 N; 3.3 Y; 3.5 N | Moderate | 4.1 Y; 4.2 N; 4.3 N; 4.5 NI | Critical | 5.1 Y; 5.2 N; 5.3 N; 5.4 NI; 5.5 Y; 5.6 SN; 5.11 N | Serious | 6.1 N; 6.2 Y; 6.3 SY | Serious | 7.1 NI; 7.2 NI; 7.3 NI; 7.4 NI |
| Sharman et al 2019 | Critical | Serious | 1.1 SN; 1.4 N | Low | 2.1 N; 2.3 Y; 2.4 N; 2.5 Y | Low | 3.1 N; 3.3 Y; 3.5 N | Moderate | 4.1 Y; 4.2 N; 4.3 N; 4.5 WN | Critical | 5.1 Y; 5.2 N; 5.3 N; 5.4 Y; 5.5 PY; 5.6 SN; 5.11 N | Moderate | 6.1 N; 6.2 Y; 6.3 WY | Moderate | 7.1 NI; 7.2 N; 7.3 NI; 7.4 NI |
| Shimizu et al 2023 | Critical | Serious | 1.1 SN; 1.4 N | Low | 2.1 N; 2.3 Y; 2.4 N; 2.5 Y | Low | 3.1 N; 3.3 Y; 3.5 N | Low | 4.1 Y; 4.2 N; 4.3 N; 4.5 Y | Critical | 5.1 Y; 5.2 N; 5.3 Y; 5.4 N; 5.7 N; 5.10 SN; 5.11 N | Low | 6.1 N; 6.2 N | Moderate | 7.1 NI; 7.2 PN; 7.3 NI; 7.4 NI |
| Shu et al 2024 | Critical | Serious | 1.1 SN; 1.4 N | Low | 2.1 N; 2.3 Y; 2.4 N; 2.5 Y | Serious | 3.1 N; 3.3 PY; 3.5 Y; 3.6 PY; 3.7 Y | Moderate | 4.1 Y; 4.2 N; 4.3 NI; 4.5 WN | Serious | 5.1 Y; 5.2 Y; 5.3 NI; 5.4 Y; 5.5 PY; 5.6 NI; 5.11 N | Moderate | 6.1 N; 6.2 Y; 6.3 WY | Serious | 7.1 NI; 7.2 NI; 7.3 NI; 7.4 NI |
| Sievers et al 2001 | Serious | Serious | 1.1 SN; 1.4 N | Low | 2.1 N; 2.3 Y; 2.4 N; 2.5 PY | Low | 3.1 N; 3.3 Y; 3.5 PN | Moderate | 4.1 Y; 4.2 N; 4.3 NI; 4.5 NI | Serious | 5.1 Y; 5.2 NI; 5.3 NI; 5.4 NI; 5.5 NI; 5.6 NI; 5.11 N | Moderate | 6.1 N; 6.2 Y; 6.3 WY | Serious | 7.1 NI; 7.2 NI; 7.3 NI; 7.4 NI |
| Song et al 2003 | Critical | Serious | 1.1 SN; 1.4 N | Low | 2.1 N; 2.3 Y; 2.4 N; 2.5 Y | Serious | 3.1 N; 3.3 PY; 3.5 PY; 3.6 PY; 3.7 Y | Serious | 4.1 Y; 4.2 N; 4.3 NI; 4.5 SN | Critical | 5.1 Y; 5.2 N; 5.3 NI; 5.4 Y; 5.5 PY; 5.6 SN; 5.11 N | Serious | 6.1 PY | Serious | 7.1 NI; 7.2 NI; 7.3 NI; 7.4 NI |
| Song et al 2024 | Critical | Serious | 1.1 SN; 1.4 N | Low | 2.1 N; 2.3 Y; 2.4 N; 2.5 PY | Low | 3.1 N; 3.3 PY; 3.5 N | Serious | 4.1 Y; 4.2 N; 4.3 N; 4.5 SN | Critical | 5.1 Y; 5.2 N; 5.3 NI; 5.4 Y; 5.5 PY; 5.6 SN; 5.11 N | Moderate | 6.1 PN; 6.2 NI; 6.3 WY | Serious | 7.1 NI; 7.2 NI; 7.3 NI; 7.4 NI |
| Spinner et al 2024 | Critical | Serious | 1.1 SN; 1.4 N | Serious | 2.1 NI; 2.2 NI; 2.4 NI | Moderate | 3.1 NI; 3.3 NI; 3.5 N | Serious | 4.1 N; 4.5 SN | Serious | 5.1 Y; 5.2 N; 5.3 NI; 5.4 NI; 5.5 NI; 5.6 NI; 5.11 N | Moderate | 6.1 N; 6.2 Y; 6.3 WY | Serious | 7.1 NI; 7.2 NI; 7.3 NI; 7.4 NI |
| Spring et al 2023 | Serious | Serious | 1.1 SN; 1.4 N | Low | 2.1 N; 2.3 Y; 2.4 N; 2.5 Y | Low | 3.1 N; 3.3 Y; 3.5 N | Low | 4.1 Y; 4.2 N; 4.3 N; 4.5 Y | Low | 5.1 Y; 5.2 N; 5.3 PY; 5.4 PY; 5.5 N | Low | 6.1 N; 6.2 PY; 6.3 PN | Moderate | 7.1 NI; 7.2 PN; 7.3 PN; 7.4 NI |
| Spurgeon et al 2022 | Serious | Serious | 1.1 SN; 1.4 N | Low | 2.1 N; 2.3 Y; 2.4 N; 2.5 Y | Low | 3.1 N; 3.3 Y; 3.5 N | Moderate | 4.1 Y; 4.2 N; 4.3 N; 4.5 NI | Serious | 5.1 Y; 5.2 PY; 5.3 NI; 5.4 NI; 5.5 NI; 5.6 NI; 5.11 N | Moderate | 6.1 N; 6.2 PY; 6.3 WY | Serious | 7.1 NI; 7.2 NI; 7.3 NI; 7.4 NI |
| Stein et al 2018 | Critical | Serious | 1.1 SN; 1.4 N | Low | 2.1 N; 2.3 Y; 2.4 N; 2.5 Y | Low | 3.1 N; 3.3 Y; 3.5 N | Moderate | 4.1 Y; 4.2 N; 4.3 N; 4.5 WN | Critical | 5.1 Y; 5.2 PY; 5.3 N; 5.4 N; 5.7 N; 5.10 SN; 5.11 N | Low | 6.1 N; 6.2 Y; 6.3 PN | Moderate | 7.1 NI; 7.2 NI; 7.3 PN; 7.4 PN |
| Strickler et al 2018 | Serious | Serious | 1.1 SN; 1.4 N | Low | 2.1 N; 2.3 Y; 2.4 N; 2.5 Y | Low | 3.1 N; 3.3 Y; 3.5 N | Low | 4.1 Y; 4.2 N; 4.3 N; 4.5 Y | Serious | 5.1 Y; 5.2 N; 5.3 PN; 5.4 N; 5.7 Y; 5.8 N | Serious | 6.1 N; 6.2 Y; 6.3 SY | Moderate | 7.1 NI; 7.2 PN; 7.3 NI; 7.4 NI |
| Tabernero et al 2021 | Critical | Serious | 1.1 SN; 1.4 N | Moderate | 2.1 N; 2.3 PY; 2.4 PN; 2.5 NI | Low | 3.1 N; 3.3 Y; 3.5 N | Moderate | 4.1 N; 4.5 NI | Serious | 5.1 Y; 5.2 N; 5.3 NI; 5.4 NI; 5.5 NI; 5.6 NI; 5.11 N | Serious | 6.1 Y | Serious | 7.1 NI; 7.2 NI; 7.3 NI; 7.4 NI |
| Tannir et al 2014 | Critical | Serious | 1.1 SN; 1.4 N | Low | 2.1 N; 2.3 Y; 2.4 N; 2.5 Y | Low | 3.1 N; 3.3 Y; 3.5 N | Moderate | 4.1 Y; 4.2 N; 4.3 N; 4.5 NI | Critical | 5.1 Y; 5.2 N; 5.3 NI; 5.4 N; 5.7 N; 5.10 SN; 5.11 N | Serious | 6.1 Y | Moderate | 7.1 NI; 7.2 NI; 7.3 NI; 7.4 PN |
| Thiruvengadam et al 2024 | Critical | Serious | 1.1 SN; 1.4 N | Low | 2.1 N; 2.3 Y; 2.4 N; 2.5 Y | Low | 3.1 N; 3.3 Y; 3.5 N | Serious | 4.1 Y; 4.2 N; 4.3 N; 4.5 SN | Critical | 5.1 Y; 5.2 N; 5.3 PY; 5.4 Y; 5.5 Y; 5.6 SN; 5.11 N | Moderate | 6.1 N; 6.2 Y; 6.3 WY | Serious | 7.1 NI; 7.2 NI; 7.3 NI; 7.4 NI |
| Thwaites et al 2023 | Critical | Serious | 1.1 SN; 1.4 N | Serious | 2.1 Y; 2.2 NI; 2.4 N | Serious | 3.2 Y; 3.3 Y; 3.5 Y; 3.6 Y; 3.7 PY | Serious | 4.1 N; 4.5 SN | Critical | 5.1 Y; 5.2 PY; 5.3 NI; 5.4 PY; 5.5 PY; 5.6 SN; 5.11 N | Low | 6.1 N; 6.2 Y; 6.3 N | Serious | 7.1 NI; 7.2 NI; 7.3 NI; 7.4 NI |
| Tolaney et al 2020 | Serious | Serious | 1.1 SN; 1.4 N | Low | 2.1 N; 2.3 Y; 2.4 N; 2.5 Y | Low | 3.1 N; 3.3 Y; 3.5 N | Low | 4.1 Y; 4.2 N; 4.3 N; 4.5 Y | Serious | 5.1 Y; 5.2 N; 5.3 PY; 5.4 N; 5.7 Y; 5.8 N | Moderate | 6.1 N; 6.2 Y; 6.3 WY | Serious | 7.1 NI; 7.2 NI; 7.3 NI; 7.4 NI |
| Tolcher et al 2003 | Serious | Serious | 1.1 SN; 1.4 N | Low | 2.1 N; 2.3 Y; 2.4 N; 2.5 Y | Low | 3.1 N; 3.3 Y; 3.5 N | Moderate | 4.1 Y; 4.2 N; 4.3 N; 4.5 NI | Low | 5.1 Y; 5.2 PY; 5.3 PY | Moderate | 6.1 N; 6.2 Y; 6.3 WY | Moderate | 7.1 NI; 7.2 NI; 7.3 NI; 7.4 PN |
| Tsai et al 2021 | Serious | Serious | 1.1 SN; 1.4 N | Low | 2.1 N; 2.3 Y; 2.4 PN; 2.5 Y | Low | 3.1 N; 3.3 Y; 3.5 N | Moderate | 4.1 Y; 4.2 N; 4.3 NI; 4.5 NI | Serious | 5.1 N; 5.2 NI; 5.3 N; 5.4 NI; 5.5 NI; 5.6 NI; 5.11 N | Moderate | 6.1 N; 6.2 Y; 6.3 WY | Serious | 7.1 NI; 7.2 NI; 7.3 NI; 7.4 NI |
| Tsurutani et al 2017 | Critical | Serious | 1.1 SN; 1.4 N | Low | 2.1 N; 2.3 Y; 2.4 N; 2.5 Y | Moderate | 3.1 N; 3.3 Y; 3.5 Y; 3.6 NI | Serious | 4.1 Y; 4.2 N; 4.3 N; 4.5 SN | Serious | 5.1 Y; 5.2 N; 5.3 NI; 5.4 PY; 5.5 PY; 5.6 NI; 5.11 N | Moderate | 6.1 N; 6.2 PY; 6.3 WY | Serious | 7.1 NI; 7.2 NI; 7.3 NI; 7.4 NI |
| Tsurutani et al 2018 | Critical | Serious | 1.1 SN; 1.4 N | Low | 2.1 N; 2.3 PY; 2.4 N; 2.5 Y | Serious | 3.1 N; 3.3 PY; 3.5 Y; 3.6 Y; 3.7 Y | Serious | 4.1 Y; 4.2 N; 4.3 N; 4.5 SN | Critical | 5.1 Y; 5.2 N; 5.3 NI; 5.4 Y; 5.5 PY; 5.6 SN; 5.11 N | Moderate | 6.1 N; 6.2 Y; 6.3 WY | Serious | 7.1 NI; 7.2 NI; 7.3 NI; 7.4 NI |
| Vij et al 2020 | Serious | Serious | 1.1 SN; 1.4 N | Low | 2.1 N; 2.3 Y; 2.4 N; 2.5 Y | Low | 3.1 N; 3.3 Y; 3.5 N | Low | 4.1 Y; 4.2 N; 4.3 N; 4.5 Y | Low | 5.1 Y; 5.2 Y; 5.3 N; 5.4 Y; 5.5 N | Serious | 6.1 PY | Moderate | 7.1 NI; 7.2 NI; 7.3 PN; 7.4 NI |
| Walker et al 2023 | Critical | Critical | 1.1 SN; 1.4 Y | Low | 2.1 N; 2.3 Y; 2.4 N; 2.5 Y | Moderate | 3.1 N; 3.3 NI; 3.5 N | Moderate | 4.1 N; 4.5 NI | Critical | 5.1 Y; 5.2 Y; 5.3 NI; 5.4 Y; 5.5 NI; 5.6 SN; 5.11 N | Moderate | 6.1 N; 6.2 Y; 6.3 WY | Serious | 7.1 NI; 7.2 NI; 7.3 NI; 7.4 NI |
| Wang et al 2020 | Serious | Serious | 1.1 SN; 1.4 N | Low | 2.1 N; 2.3 Y; 2.4 N; 2.5 Y | Low | 3.1 N; 3.3 Y; 3.5 N | Low | 4.1 Y; 4.2 N; 4.3 N; 4.5 PY | Low | 5.1 Y; 5.2 PY; 5.3 PY | Moderate | 6.1 N; 6.2 PY; 6.3 WY | Serious | 7.1 NI; 7.2 NI; 7.3 NI; 7.4 NI |
| Wang et al 2023 | Critical | Serious | 1.1 SN; 1.4 N | Low | 2.1 N; 2.3 PY; 2.4 N; 2.5 PY | Moderate | 3.1 N; 3.3 PY; 3.5 NI | Moderate | 4.1 Y; 4.2 N; 4.3 NI; 4.5 WN | Serious | 5.1 PY; 5.2 N; 5.3 NI; 5.4 Y; 5.5 NI; 5.6 NI; 5.11 N | Serious | 6.1 N; 6.2 PY; 6.3 SY | Serious | 7.1 NI; 7.2 NI; 7.3 NI; 7.4 NI |
| Wang et al 2023 | Critical | Serious | 1.1 SN; 1.4 N | Low | 2.1 N; 2.3 Y; 2.4 N; 2.5 Y | Moderate | 3.1 N; 3.3 Y; 3.5 Y; 3.6 NI | Serious | 4.1 Y; 4.2 N; 4.3 NI; 4.5 SN | Critical | 5.1 Y; 5.2 N; 5.3 NI; 5.4 Y; 5.5 PY; 5.6 SN; 5.11 N | Moderate | 6.1 PN; 6.2 Y; 6.3 WY | Serious | 7.1 NI; 7.2 NI; 7.3 NI; 7.4 NI |
| Wang et al 2023 | Critical | Serious | 1.1 SN; 1.4 N | Low | 2.1 N; 2.3 PY; 2.4 N; 2.5 Y | Serious | 3.1 N; 3.3 PY; 3.5 Y; 3.6 PY; 3.7 PY | Serious | 4.1 Y; 4.2 N; 4.3 NI; 4.5 SN | Serious | 5.1 Y; 5.2 N; 5.3 NI; 5.4 Y; 5.5 PY; 5.6 NI; 5.11 N | Serious | 6.1 N; 6.2 Y; 6.3 SY | Serious | 7.1 NI; 7.2 NI; 7.3 NI; 7.4 NI |
| Wang et al 2024 | Serious | Serious | 1.1 SN; 1.4 N | Low | 2.1 N; 2.3 Y; 2.4 N; 2.5 PY | Low | 3.1 N; 3.3 PY; 3.5 N | Low | 4.1 N; 4.5 Y | Low | 5.1 Y; 5.2 Y; 5.3 Y | Moderate | 6.1 N; 6.2 Y; 6.3 WY | Serious | 7.1 NI; 7.2 NI; 7.3 NI; 7.4 NI |
| Waqar et al 2024 | Serious | Serious | 1.1 SN; 1.4 N | Low | 2.1 N; 2.3 Y; 2.4 N; 2.5 Y | Low | 3.1 N; 3.3 Y; 3.5 N | Low | 4.1 Y; 4.2 N; 4.3 N; 4.5 PY | Low | 5.1 Y; 5.2 Y; 5.3 NI; 5.4 NI; 5.5 N | Serious | 6.1 N; 6.2 Y; 6.3 SY | Serious | 7.1 NI; 7.2 NI; 7.3 NI; 7.4 NI |
| Westervelt et al 2022 | Serious | Serious | 1.1 SN; 1.4 N | Low | 2.1 N; 2.3 Y; 2.4 N; 2.5 Y | Low | 3.1 N; 3.3 Y; 3.5 N | Moderate | 4.1 Y; 4.2 N; 4.3 N; 4.5 NI | Serious | 5.1 Y; 5.2 N; 5.3 NI; 5.4 PY; 5.5 Y; 5.6 NI; 5.11 N | Moderate | 6.1 PN; 6.2 Y; 6.3 WY | Serious | 7.1 NI; 7.2 NI; 7.3 NI; 7.4 NI |
| Williams et al 2023 | Critical | Critical | 1.1 SN; 1.4 PY | Serious | 2.1 Y; 2.2 N; 2.4 PN | Low | 3.2 N; 3.3 PY; 3.5 N | Moderate | 4.1 N; 4.5 NI | Low | 5.1 Y; 5.2 Y; 5.3 NI; 5.4 NI; 5.5 N | Serious | 6.1 PY | Serious | 7.1 NI; 7.2 NI; 7.3 NI; 7.4 NI |
| Winer et al 2025 | Critical | Serious | 1.1 SN; 1.4 N | Low | 2.1 N; 2.3 Y; 2.4 N; 2.5 Y | Serious | 3.1 N; 3.3 Y; 3.5 PY; 3.6 Y; 3.7 PY | Serious | 4.1 Y; 4.2 N; 4.3 N; 4.5 SN | Serious | 5.1 Y; 5.2 N; 5.3 NI; 5.4 Y; 5.5 PY; 5.6 NI; 5.11 N | Moderate | 6.1 PN; 6.2 Y; 6.3 WY | Serious | 7.1 NI; 7.2 NI; 7.3 NI; 7.4 NI |
| Wu et al 2024 | Critical | Serious | 1.1 SN; 1.4 N | Low | 2.1 N; 2.3 Y; 2.4 N; 2.5 Y | Low | 3.1 N; 3.3 PY; 3.5 N | Serious | 4.1 Y; 4.2 N; 4.3 NI; 4.5 SN | Serious | 5.1 Y; 5.2 N; 5.3 NI; 5.4 Y; 5.5 PY; 5.6 NI; 5.11 N | Moderate | 6.1 NI; 6.2 NI; 6.3 WY | Serious | 7.1 NI; 7.2 NI; 7.3 NI; 7.4 NI |
| Wu et al 2025 | Serious | Serious | 1.1 SN; 1.4 N | Low | 2.1 N; 2.3 Y; 2.4 N; 2.5 Y | Low | 3.1 N; 3.3 Y; 3.5 N | Moderate | 4.1 Y; 4.2 N; 4.3 N; 4.5 NI | Serious | 5.1 Y; 5.2 NI; 5.3 NI; 5.4 PY; 5.5 NI; 5.6 NI; 5.11 N | Moderate | 6.1 N; 6.2 Y; 6.3 WY | Serious | 7.1 NI; 7.2 NI; 7.3 NI; 7.4 NI |
| Xu et al 2022 | Serious | Serious | 1.1 SN; 1.4 N | Low | 2.1 N; 2.3 Y; 2.4 N; 2.5 Y | Low | 3.1 N; 3.3 Y; 3.5 N | Low | 4.1 Y; 4.2 N; 4.3 NI; 4.5 PY | Low | 5.1 Y; 5.2 Y; 5.3 NI; 5.4 Y; 5.5 N | Serious | 6.1 N; 6.2 Y; 6.3 SY | Serious | 7.1 NI; 7.2 NI; 7.3 NI; 7.4 NI |
| Xu et al 2023 | Serious | Serious | 1.1 SN; 1.4 N | Low | 2.1 N; 2.3 PY; 2.4 N; 2.5 Y | Low | 3.1 N; 3.3 Y; 3.5 N | Moderate | 4.1 Y; 4.2 N; 4.3 NI; 4.5 NI | Serious | 5.1 PY; 5.2 N; 5.3 NI; 5.4 PY; 5.5 NI; 5.6 NI; 5.11 N | Moderate | 6.1 NI; 6.2 NI; 6.3 WY | Serious | 7.1 NI; 7.2 NI; 7.3 NI; 7.4 NI |
| Xu et al 2025 | Serious | Serious | 1.1 SN; 1.4 N | Low | 2.1 N; 2.3 Y; 2.4 N; 2.5 PY | Low | 3.1 N; 3.3 PY; 3.5 N | Moderate | 4.1 Y; 4.2 N; 4.3 NI; 4.5 NI | Low | 5.1 Y; 5.2 Y; 5.3 NI; 5.4 Y; 5.5 N | Moderate | 6.1 N; 6.2 NI; 6.3 WY | Serious | 7.1 NI; 7.2 NI; 7.3 NI; 7.4 NI |
| Yao et al 2025 | Critical | Serious | 1.1 SN; 1.4 N | Low | 2.1 N; 2.3 Y; 2.4 N; 2.5 Y | Low | 3.1 N; 3.3 PY; 3.5 N | Serious | 4.1 Y; 4.2 N; 4.3 N; 4.5 SN | Critical | 5.1 Y; 5.2 N; 5.3 NI; 5.4 PY; 5.5 NI; 5.6 SN; 5.11 N | Moderate | 6.1 PN; 6.2 PY; 6.3 WY | Serious | 7.1 NI; 7.2 NI; 7.3 NI; 7.4 NI |
| Ye et al 2024 | Critical | Serious | 1.1 SN; 1.4 N | Low | 2.1 N; 2.3 PY; 2.4 N; 2.5 PY | Low | 3.1 N; 3.3 PY; 3.5 N | Serious | 4.1 Y; 4.2 N; 4.3 NI; 4.5 SN | Serious | 5.1 PY; 5.2 N; 5.3 NI; 5.4 PY; 5.5 PY; 5.6 NI; 5.11 N | Serious | 6.1 NI; 6.2 NI; 6.3 SY | Serious | 7.1 NI; 7.2 NI; 7.3 NI; 7.4 NI |
| Yonemori et al 2022 | Critical | Serious | 1.1 SN; 1.4 N | Low | 2.1 N; 2.3 Y; 2.4 N; 2.5 Y | Low | 3.1 N; 3.3 Y; 3.5 N | Low | 4.1 Y; 4.2 N; 4.3 N; 4.5 Y | Critical | 5.1 Y; 5.2 N; 5.3 PY; 5.4 N; 5.7 N; 5.10 SN; 5.11 N | Moderate | 6.1 N; 6.2 Y; 6.3 WY | Moderate | 7.1 NI; 7.2 PN; 7.3 PN; 7.4 NI |
| Younes et al 2010 | Serious | Serious | 1.1 SN; 1.4 N | Low | 2.1 N; 2.3 Y; 2.4 N; 2.5 Y | Low | 3.1 N; 3.3 Y; 3.5 N | Low | 4.1 Y; 4.2 N; 4.3 N; 4.5 PY | Low | 5.1 Y; 5.2 PY; 5.3 PY | Moderate | 6.1 N; 6.2 Y; 6.3 WY | Low | 7.1 PY |
| Younes et al 2012 | Serious | Serious | 1.1 SN; 1.4 N | Low | 2.1 N; 2.3 Y; 2.4 N; 2.5 Y | Low | 3.1 N; 3.3 Y; 3.5 N | Moderate | 4.1 Y; 4.2 N; 4.3 N; 4.5 WN | Low | 5.1 Y; 5.2 Y; 5.3 PY | Moderate | 6.1 N; 6.2 NI; 6.3 WY | Moderate | 7.1 NI; 7.2 PN; 7.3 NI; 7.4 PN |
| Zhang et al 2023 | Critical | Serious | 1.1 SN; 1.4 N | Low | 2.1 N; 2.3 Y; 2.4 N; 2.5 Y | Serious | 3.1 N; 3.3 PY; 3.5 Y; 3.6 PY; 3.7 Y | Serious | 4.1 Y; 4.2 N; 4.3 N; 4.5 SN | Critical | 5.1 Y; 5.2 N; 5.3 NI; 5.4 Y; 5.5 PY; 5.6 SN; 5.11 N | Moderate | 6.1 N; 6.2 Y; 6.3 WY | Serious | 7.1 NI; 7.2 NI; 7.3 NI; 7.4 NI |
| Zhang et al 2024 | Critical | Serious | 1.1 SN; 1.4 N | Low | 2.1 N; 2.3 Y; 2.4 N; 2.5 Y | Serious | 3.1 N; 3.3 Y; 3.5 Y; 3.6 PY; 3.7 PY | Serious | 4.1 Y; 4.2 N; 4.3 NI; 4.5 SN | Serious | 5.1 Y; 5.2 N; 5.3 NI; 5.4 Y; 5.5 PY; 5.6 NI; 5.11 N | Moderate | 6.1 N; 6.2 NI; 6.3 WY | Serious | 7.1 NI; 7.2 NI; 7.3 NI; 7.4 NI |
| Zhao et al 2024 | Serious | Serious | 1.1 SN; 1.4 N | Low | 2.1 N; 2.3 Y; 2.4 N; 2.5 Y | Moderate | 3.1 N; 3.3 Y; 3.5 Y; 3.6 NI | Moderate | 4.1 Y; 4.2 N; 4.3 NI; 4.5 WN | Serious | 5.1 Y; 5.2 NI; 5.3 NI; 5.4 PY; 5.5 PY; 5.6 NI; 5.11 N | Moderate | 6.1 N; 6.2 Y; 6.3 WY | Serious | 7.1 NI; 7.2 NI; 7.3 NI; 7.4 NI |
| Zhao et al 2025 | Critical | Serious | 1.1 SN; 1.4 N | Low | 2.1 N; 2.3 Y; 2.4 N; 2.5 Y | Low | 3.1 N; 3.3 Y; 3.5 PN | Serious | 4.1 Y; 4.2 N; 4.3 NI; 4.5 SN | Critical | 5.1 Y; 5.2 NI; 5.3 NI; 5.4 PY; 5.5 NI; 5.6 SN; 5.11 N | Moderate | 6.1 N; 6.2 Y; 6.3 WY | Serious | 7.1 NI; 7.2 NI; 7.3 NI; 7.4 NI |
| Zhong et al 2025 | Critical | Serious | 1.1 SN; 1.4 N | Low | 2.1 N; 2.3 Y; 2.4 N; 2.5 Y | Low | 3.1 N; 3.3 Y; 3.5 N | Serious | 4.1 Y; 4.2 N; 4.3 NI; 4.5 SN | Serious | 5.1 Y; 5.2 N; 5.3 NI; 5.4 PY; 5.5 PY; 5.6 NI; 5.11 N | Moderate | 6.1 N; 6.2 PY; 6.3 WY | Serious | 7.1 NI; 7.2 NI; 7.3 NI; 7.4 NI |
| Zhou et al 2022 | Serious | Serious | 1.1 SN; 1.4 N | Low | 2.1 N; 2.3 Y; 2.4 N; 2.5 Y | Low | 3.1 N; 3.3 PY; 3.5 N | Moderate | 4.1 Y; 4.2 N; 4.3 NI; 4.5 NI | Serious | 5.1 Y; 5.2 NI; 5.3 NI; 5.4 NI; 5.5 NI; 5.6 NI; 5.11 N | Moderate | 6.1 N; 6.2 Y; 6.3 WY | Serious | 7.1 NI; 7.2 NI; 7.3 NI; 7.4 NI |
| Zhou et al 2024 | Critical | Serious | 1.1 SN; 1.4 N | Low | 2.1 N; 2.3 PY; 2.4 N; 2.5 Y | Low | 3.1 N; 3.3 Y; 3.5 N | Serious | 4.1 Y; 4.2 N; 4.3 NI; 4.5 SN | Serious | 5.1 PY; 5.2 PY; 5.3 NI; 5.4 PY; 5.5 PY; 5.6 NI; 5.11 N | Moderate | 6.1 N; 6.2 Y; 6.3 WY | Serious | 7.1 NI; 7.2 NI; 7.3 NI; 7.4 NI |

**2.2**

| Study | Overall | D1 | D1 Notes | D2 | D2 Notes | D3 | D3 Notes | D4 | D4 Notes | D5 | D5 Notes |
| --- | --- | --- | --- | --- | --- | --- | --- | --- | --- | --- | --- |
| Bardia et al 2024 | High risk | Some concerns | 1.2 Y; 1.1 Y; 1.3 N | High risk | 2.1 Y; 2.2 Y; 2.3 PY; 2.4 PN; 2.6 Y; 2.1 Y; 2.2 Y; 2.3 N; 2.6 N | Low risk | 3.1 Y | Low risk | 4.1 N; 4.2 N; 4.3 N | Low risk | 5.2 PN; 5.3 PN; 5.1 PY |
| Johnson et al 2021 | High risk | Some concerns | 1.2 PY; 1.1 NI; 1.3 N | High risk | 2.1 N; 2.2 N; 2.6 Y; 2.1 N; 2.2 PY; 2.3 PY; 2.4 N; 2.5 Y; 2.6 N | Low risk | 3.1 Y | Low risk | 4.1 N; 4.2 N; 4.3 N | Low risk | 5.2 PN; 5.3 PN; 5.1 PY |
| Kollmannsberger et al 2021 | High risk | Some concerns | 1.2 NI; 1.3 N | High risk | 2.1 Y; 2.2 Y; 2.3 PN; 2.6 Y; 2.1 Y; 2.2 Y; 2.3 NI; 2.6 N | Low risk | 3.1 Y | Some concerns | 4.1 N; 4.2 N; 4.3 Y; 4.4 Y; 4.5 PN | Some concerns | 5.2 N; 5.3 PN; 5.1 NI |
| Loibl et al 2022 | High risk | Some concerns | 1.2 NI; 1.3 N | High risk | 2.1 Y; 2.2 Y; 2.3 PN; 2.6 Y; 2.1 Y; 2.2 Y; 2.3 NI; 2.6 N | High risk | 3.1 N; 3.2 N; 3.3 PY; 3.4 Y | High risk | 4.1 N; 4.2 N; 4.3 Y; 4.4 Y; 4.5 Y | Some concerns | 5.2 PN; 5.3 NI |
| Marme et al 2023 | High risk | Some concerns | 1.2 NI; 1.3 NI | High risk | 2.1 PY; 2.2 PY; 2.3 NI; 2.6 Y; 2.1 Y; 2.2 Y; 2.3 NI; 2.6 N | High risk | 3.1 N; 3.2 N; 3.3 PY; 3.4 Y | High risk | 4.1 N; 4.2 PN; 4.3 Y; 4.4 Y; 4.5 PY | Some concerns | 5.2 NI; 5.3 NI |
| Nookda et al 2020 | High risk | Some concerns | 1.2 NI; 1.3 NI | High risk | 2.1 PY; 2.2 Y; 2.3 N; 2.6 NI; 2.7 NI; 2.1 Y; 2.2 Y; 2.3 NA; 2.4 N; 2.5 N | Some concerns | 3.1 NI; 3.2 NI; 3.3 PN | High risk | 4.1 N; 4.2 N; 4.3 Y; 4.4 Y; 4.5 PY | Some concerns | 5.2 NI; 5.3 NI |
| Sureda-Balari et al 2022 | High risk | Some concerns | 1.2 NI; 1.3 NI | High risk | 2.1 PY; 2.2 Y; 2.3 PN; 2.6 N; 2.7 Y; 2.1 Y; 2.2 Y; 2.3 NI; 2.6 N | High risk | 3.1 N; 3.2 N; 3.3 PY; 3.4 Y | Some concerns | 4.1 N; 4.2 PN; 4.3 NI; 4.4 Y; 4.5 PN | Some concerns | 5.2 PN; 5.3 NI |
| Wu et al 2023 | High risk | Some concerns | 1.2 NI; 1.3 NI | High risk | 2.1 Y; 2.2 Y; 2.3 NI; 2.6 NI; 2.7 NI; 2.1 Y; 2.2 Y; 2.3 NI; 2.6 N | High risk | 3.1 NI; 3.2 NI; 3.3 PY; 3.4 NI | Low risk | 4.1 N; 4.2 PN; 4.3 PY; 4.4 PN | Some concerns | 5.2 PN; 5.3 NI |
| Yasenchak et al 2015 | High risk | Some concerns | 1.2 NI; 1.3 NI | High risk | 2.1 PY; 2.2 PY; 2.3 NI; 2.6 NI; 2.7 NI; 2.1 PY; 2.2 PY; 2.3 NI; 2.6 N | High risk | 3.1 NI; 3.2 NI; 3.3 PY; 3.4 NI | Some concerns | 4.1 N; 4.2 N; 4.3 PY; 4.4 PY; 4.5 PN | Some concerns | 5.2 NI; 5.3 NI |
| Zhang et al 2025 | High risk | Some concerns | 1.2 NI; 1.3 NI | High risk | 2.1 PY; 2.2 PY; 2.3 NI; 2.6 NI; 2.7 Y; 2.1 PY; 2.2 Y; 2.3 NI; 2.6 N | High risk | 3.1 N; 3.2 N; 3.3 PY; 3.4 Y | High risk | 4.1 PN; 4.2 PN; 4.3 PY; 4.4 Y; 4.5 PY | Some concerns | 5.2 NI; 5.3 NI |

**Appendix 3.** Prevalence of Nausea by Antibody Drug Conjugate **3.1** Brentuximab Vedotin **3.2** Sacituzumab Govitecan **3.3** Datopotamab Deruxtecan **3.4** Telisotuzumab Vedotin **3.5** Disitamab Vedotin **3.6** Patritumab Deruxtecan **3.7** Rovalpituzumab Tesirine **3.8** Trastuzumab Deruxtecan **3.9** Belantamab Mafodotin **3.10** Inotuzumab Ozogamicin **3.11** Tak-264 **3.12** BL-B01D1 **3.13** Camidanlumab Tesirine **3.14** Cofetuzumab Pelidotin **3.15** Gemtuzumab Ozogamicin **3.16** IMGN632 **3.17** JSKN003 **3.18** Loncastuximab Tesirine **3.19** Polatuzumab Vedotin **3.20** Puxitatub Samrotecan **3.21** SYS6010 **3.22** Tisotumab Vedotin **3.23** Tesumitamab Ravtansine **3.24** Zilvertamab Vedotin

**3.1**

**
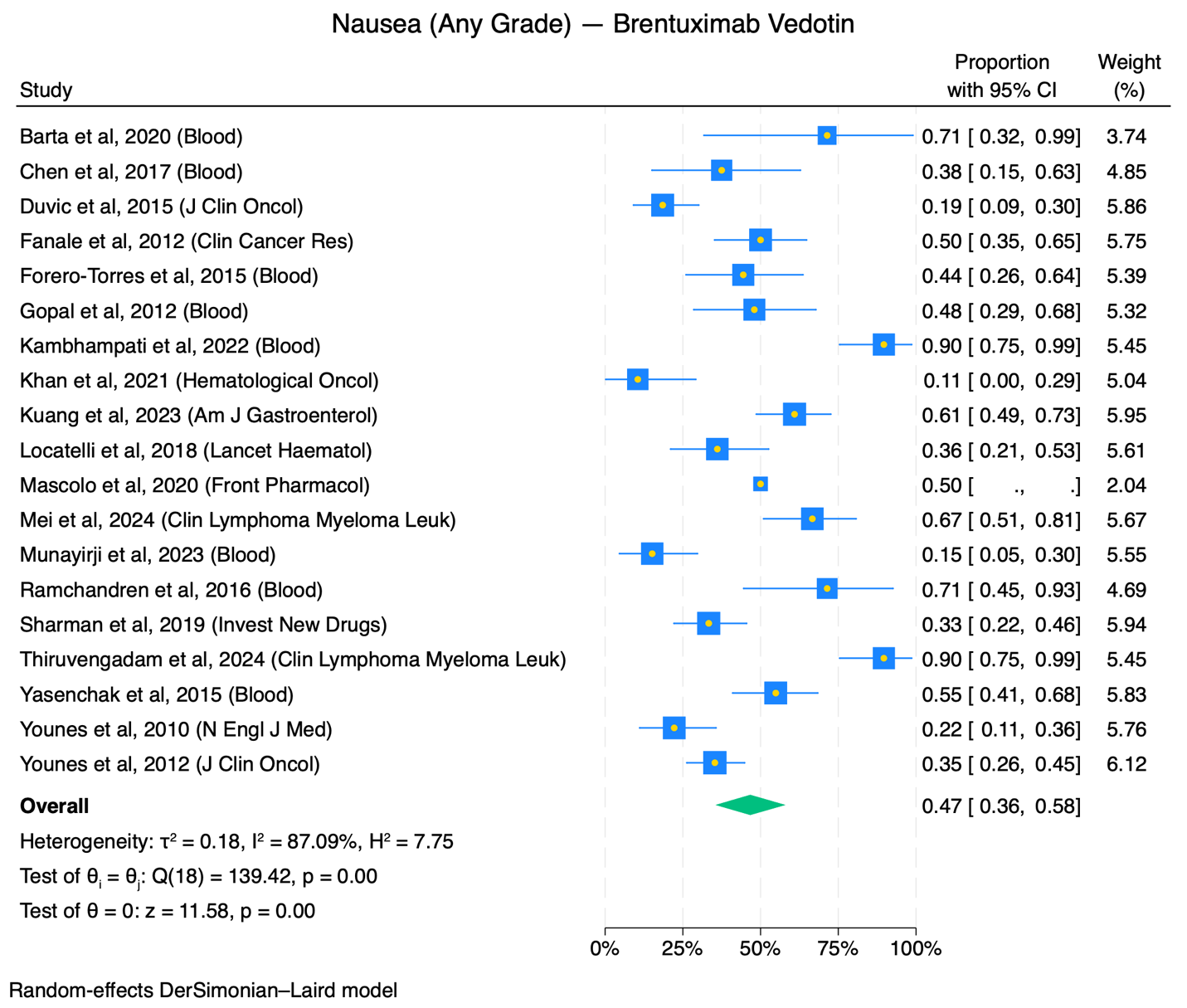
**

**3.2**

**
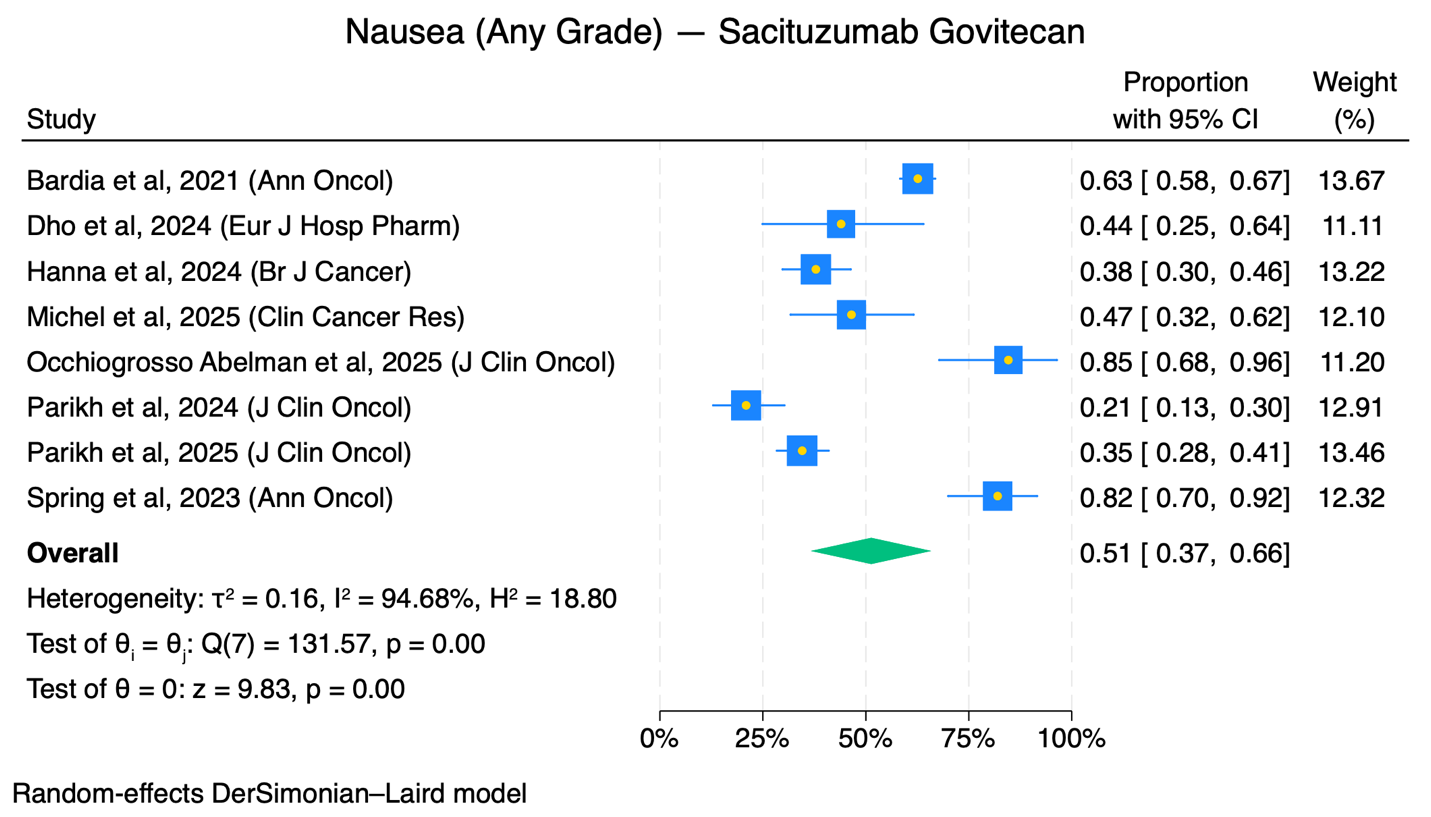
**

**3.3**


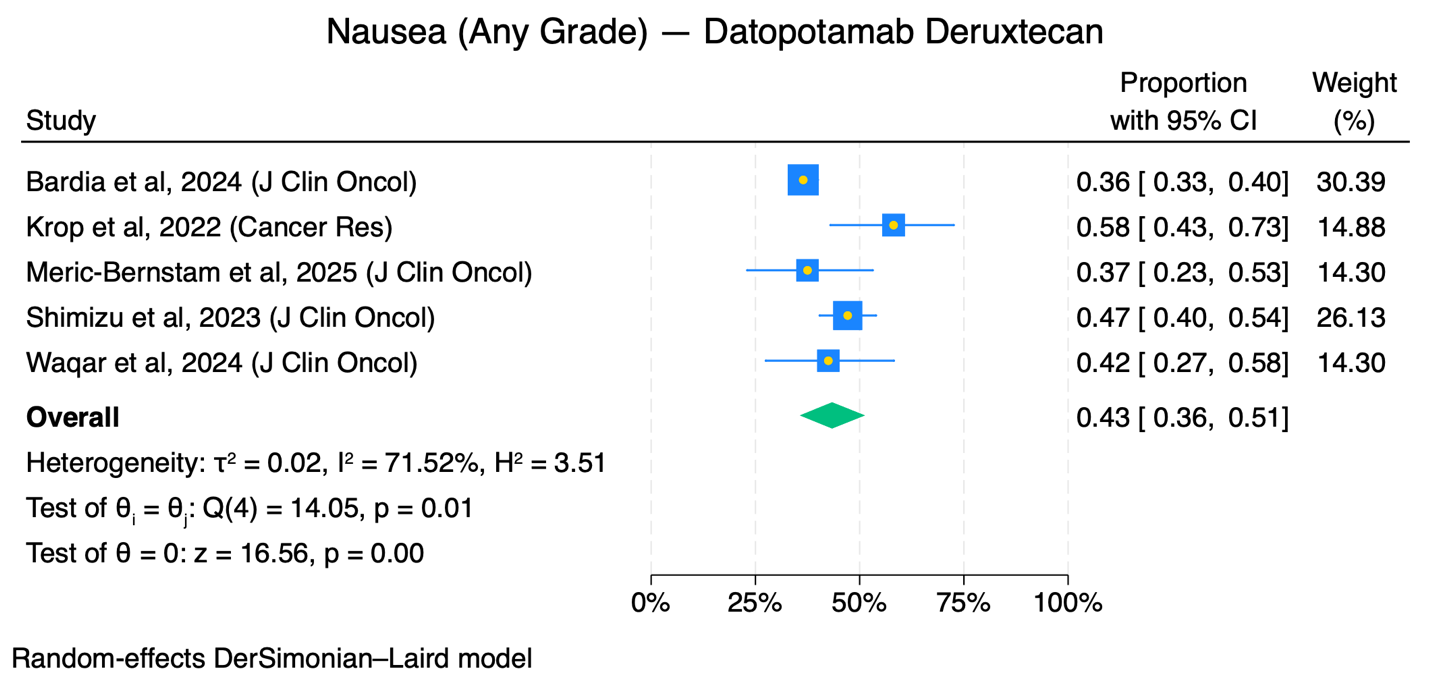


**3.4**


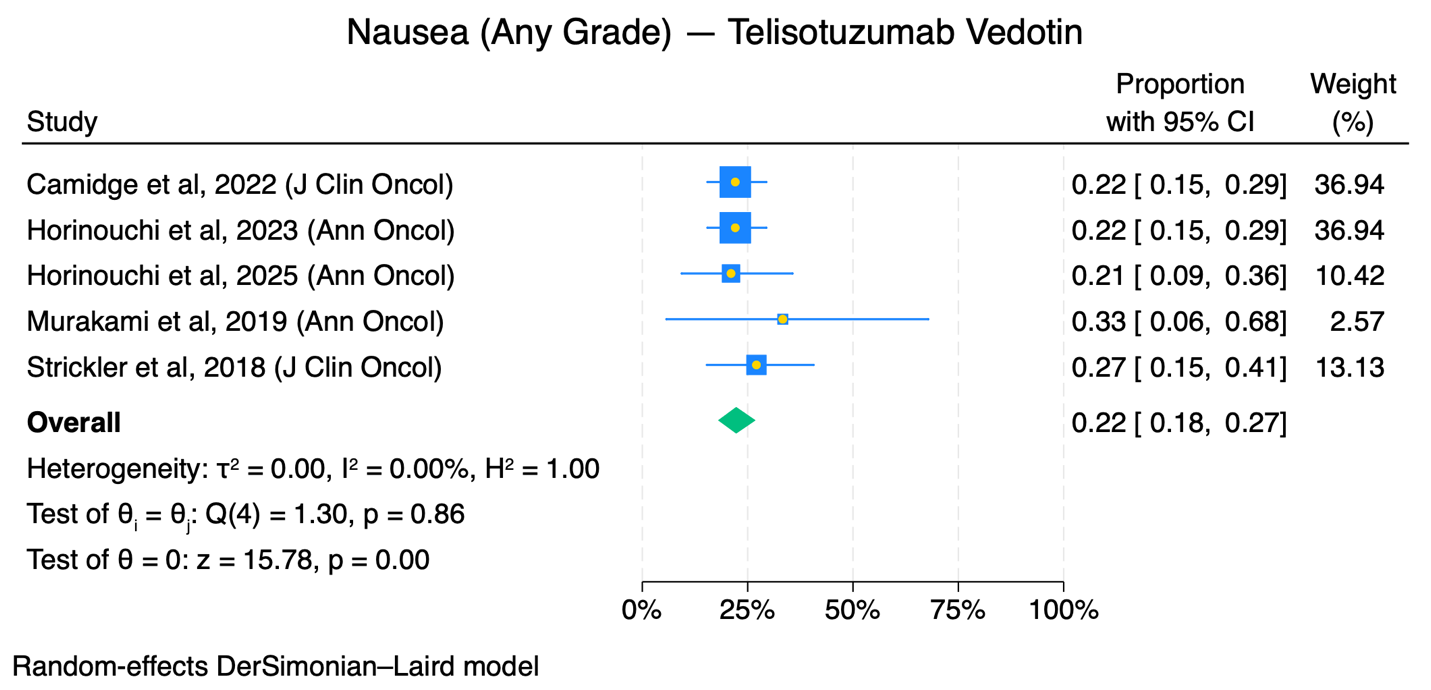


**3.5**

**
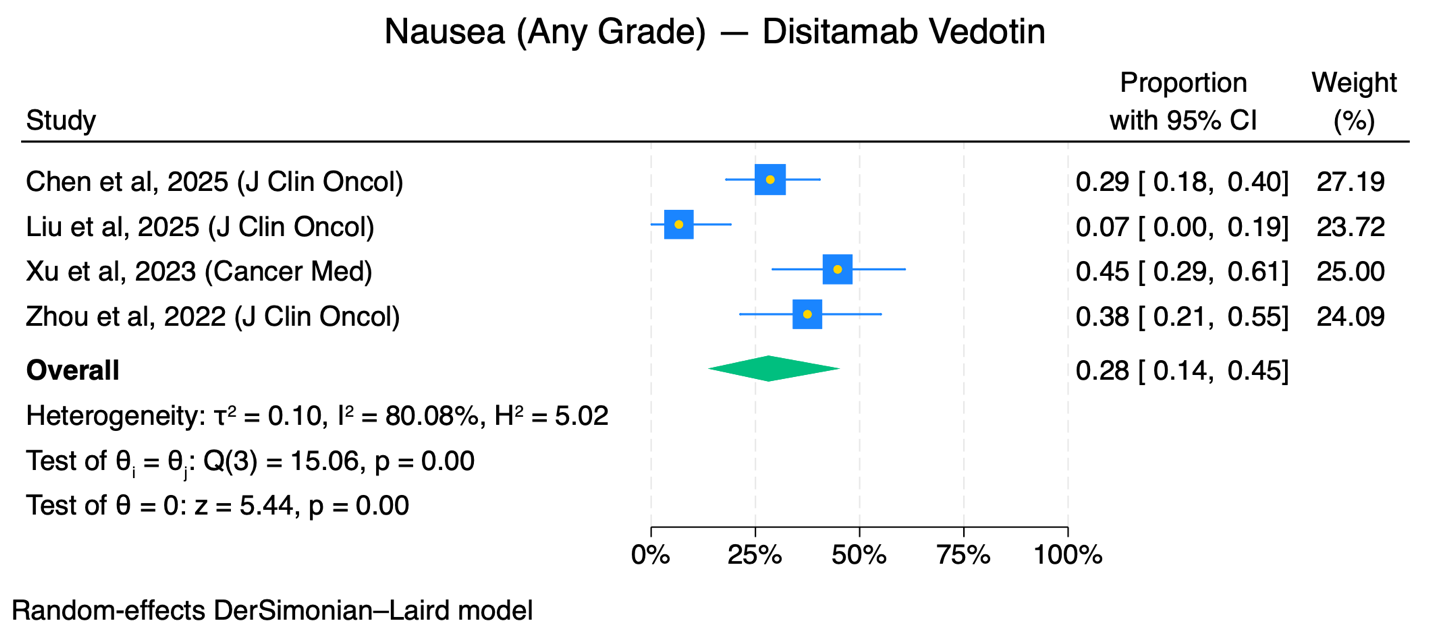
**

**3.6**

**
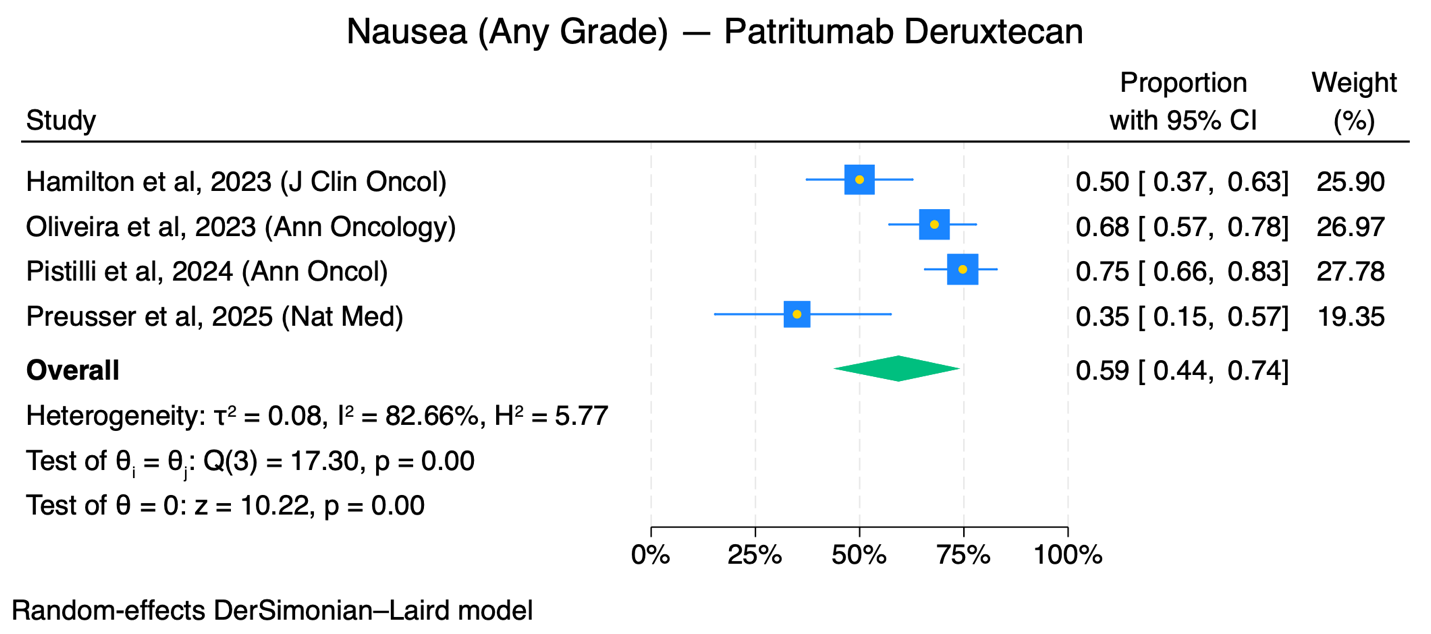
**

**3.7**

**
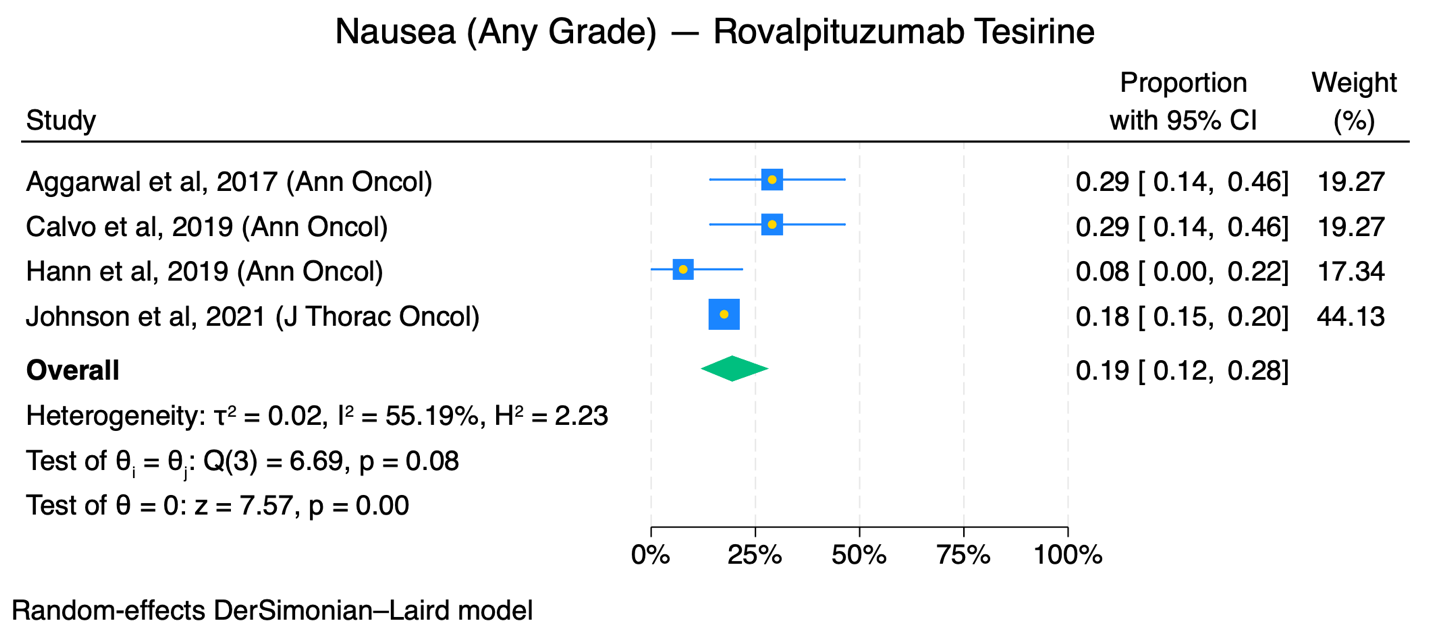
**

**3.8**

**
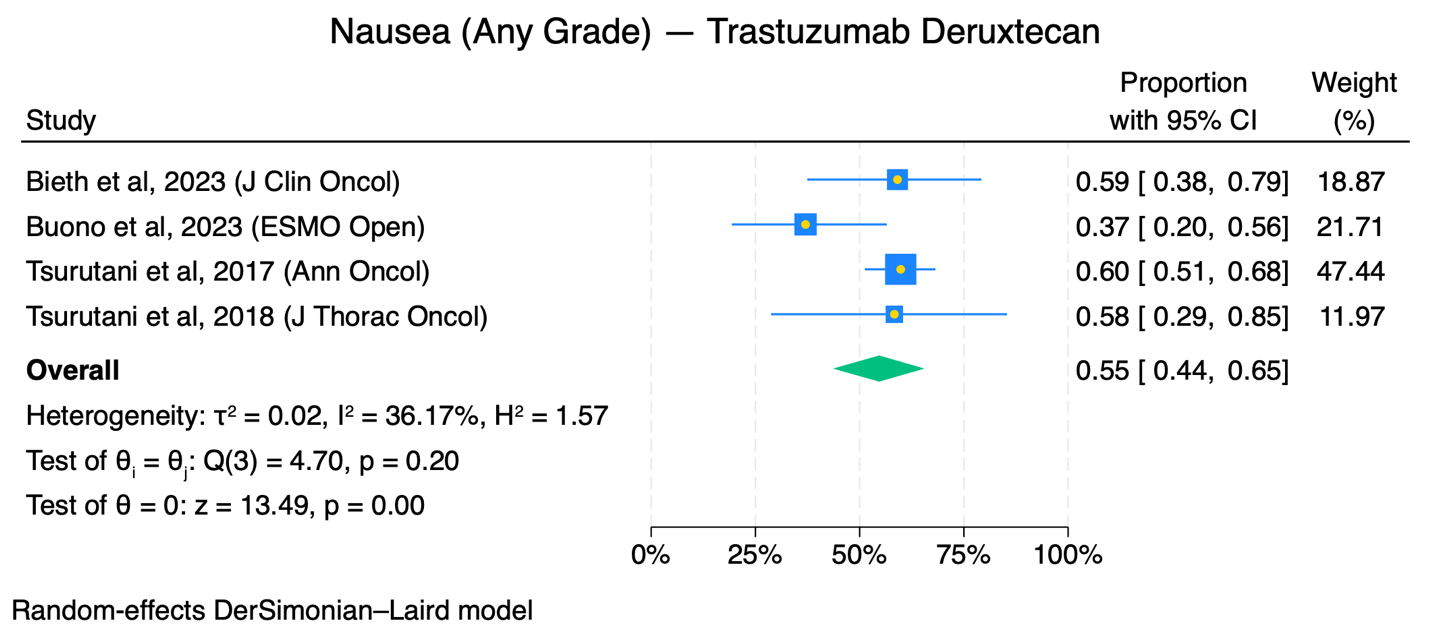
**

**3.9**


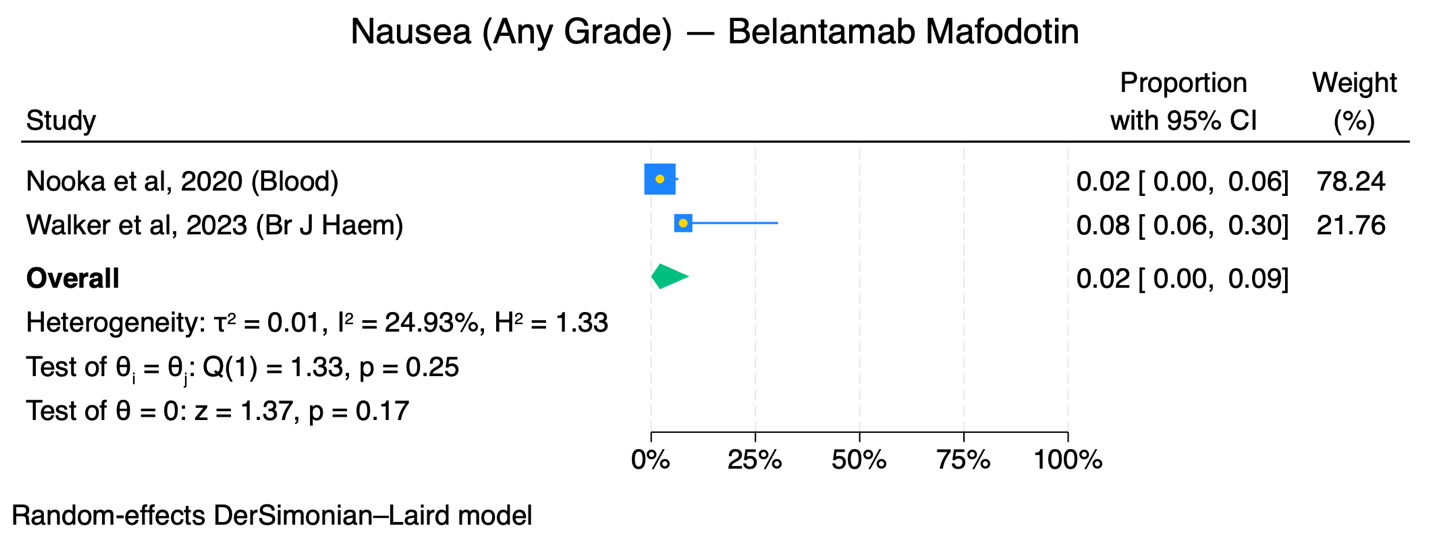


**3.10**


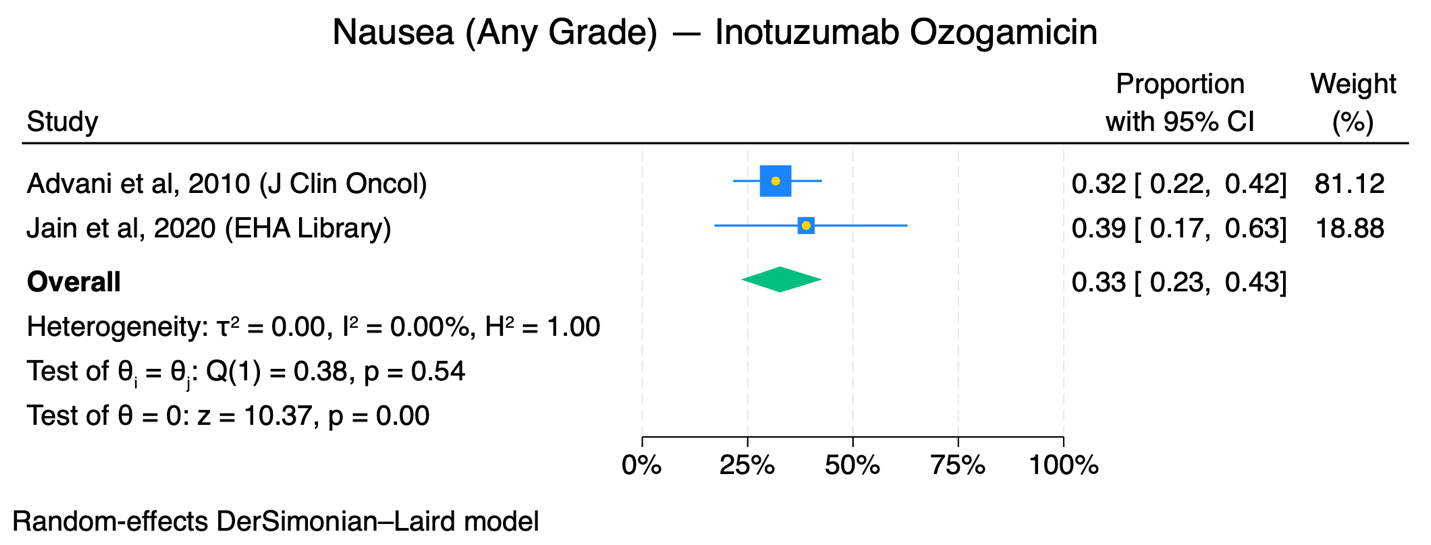


**3.11**


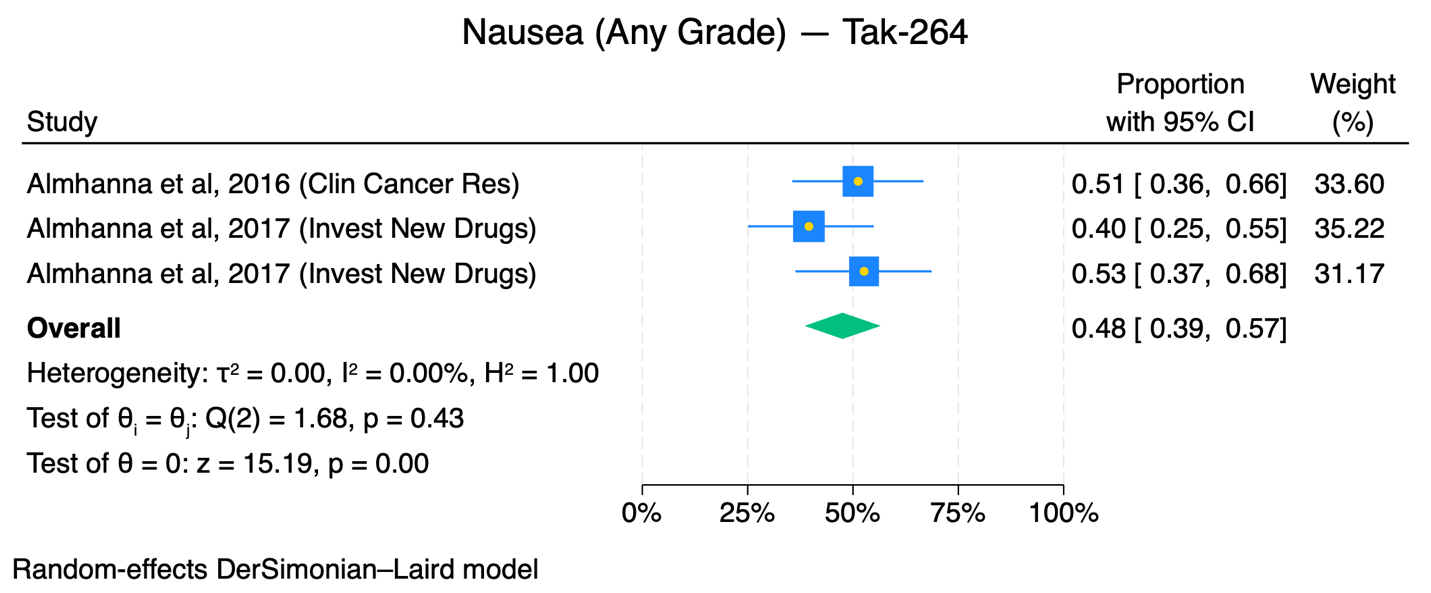


**3.12**


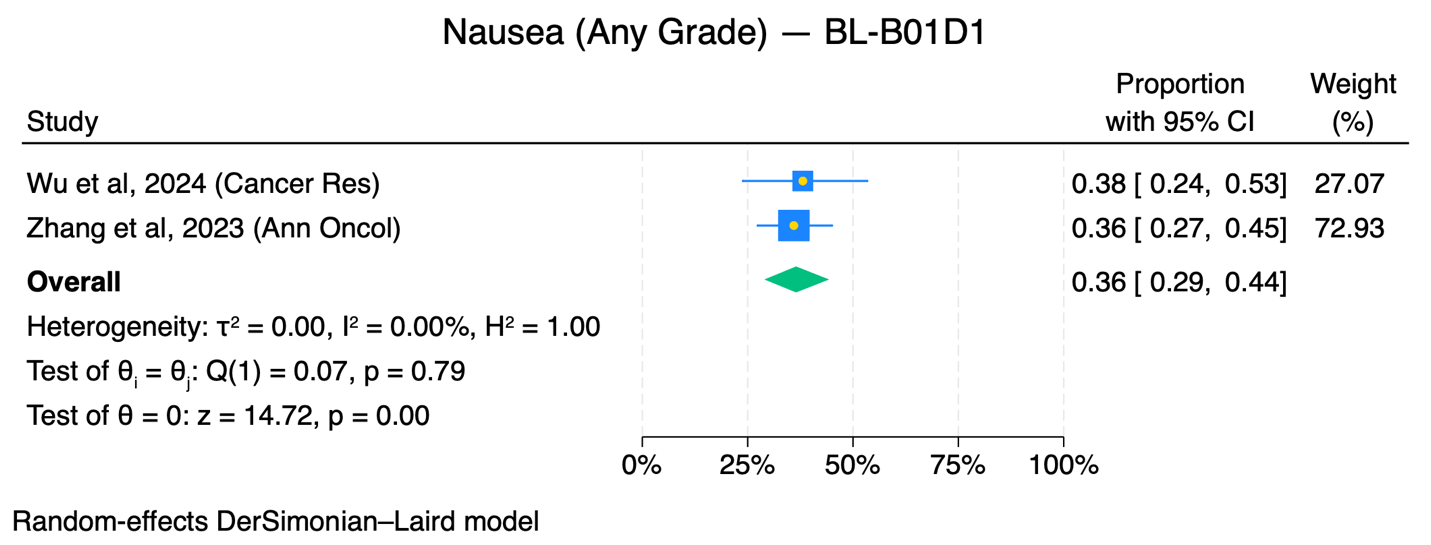


**3.13**


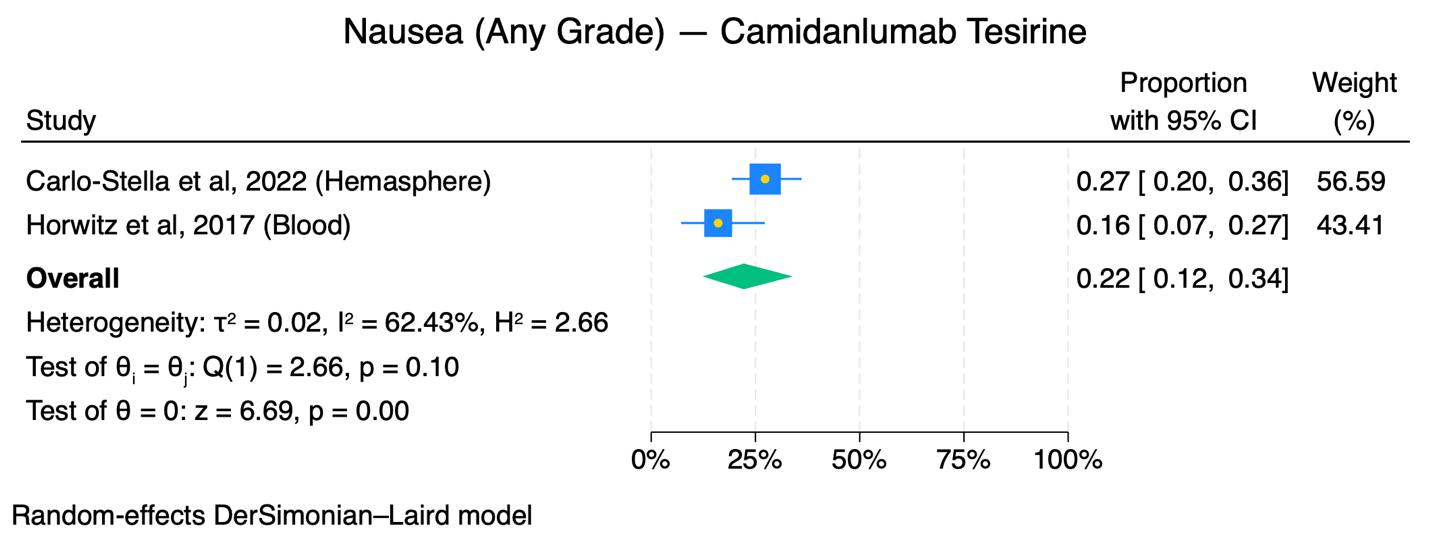


**3.14
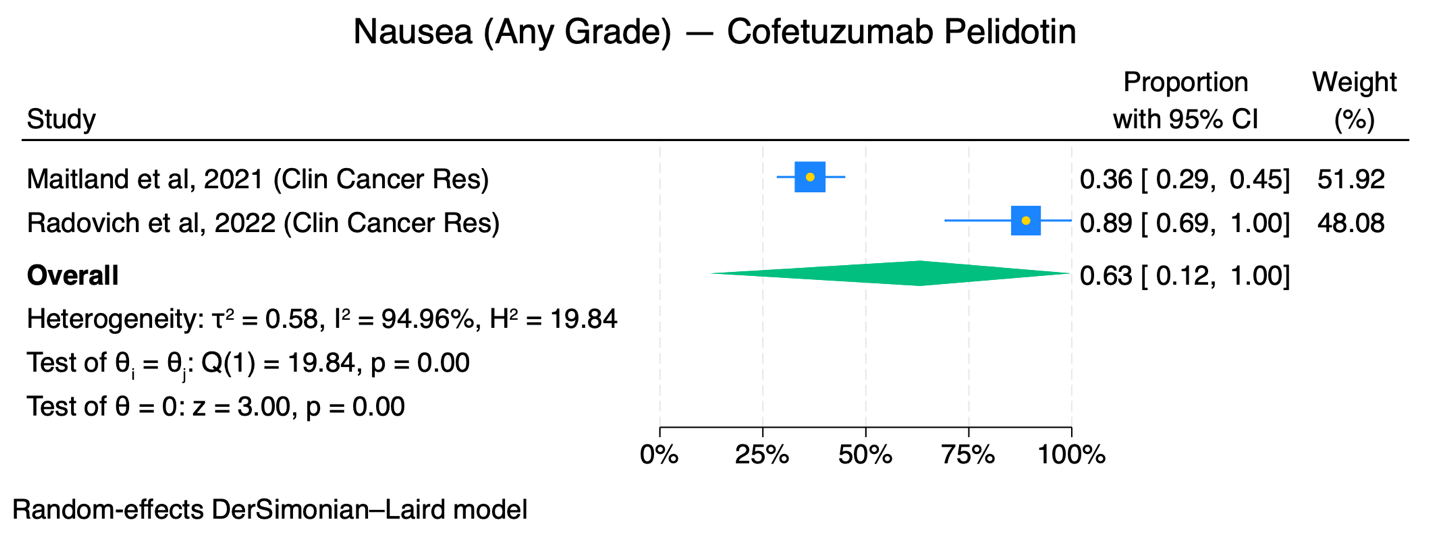
**

**3.15**


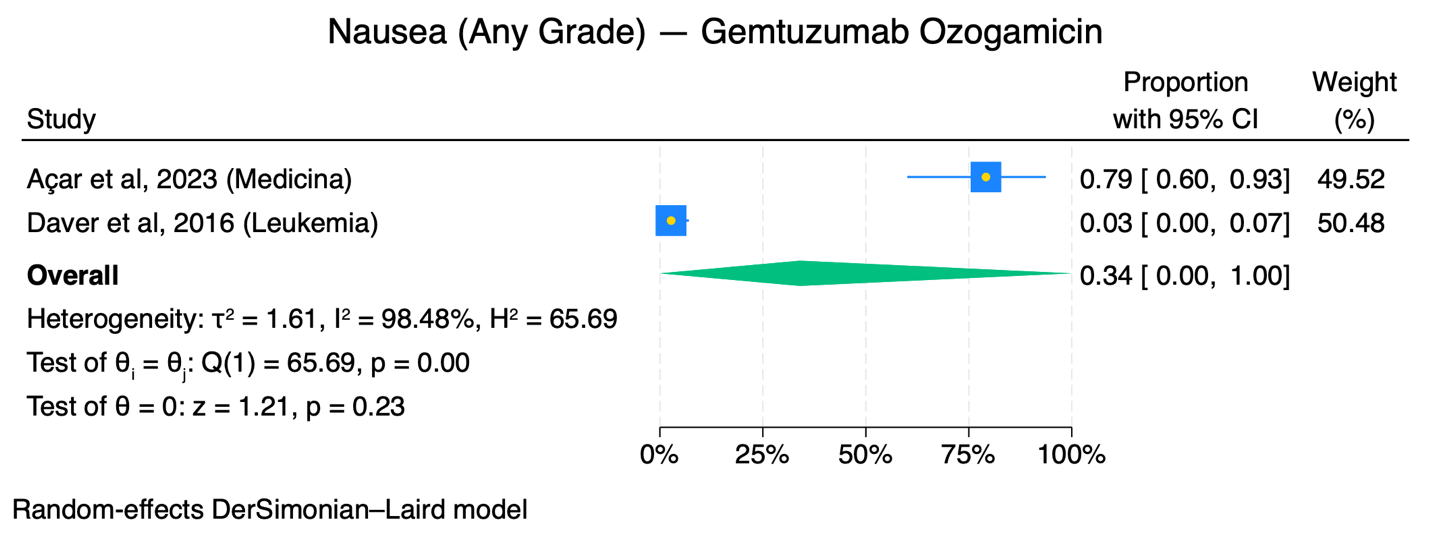


**3.16**


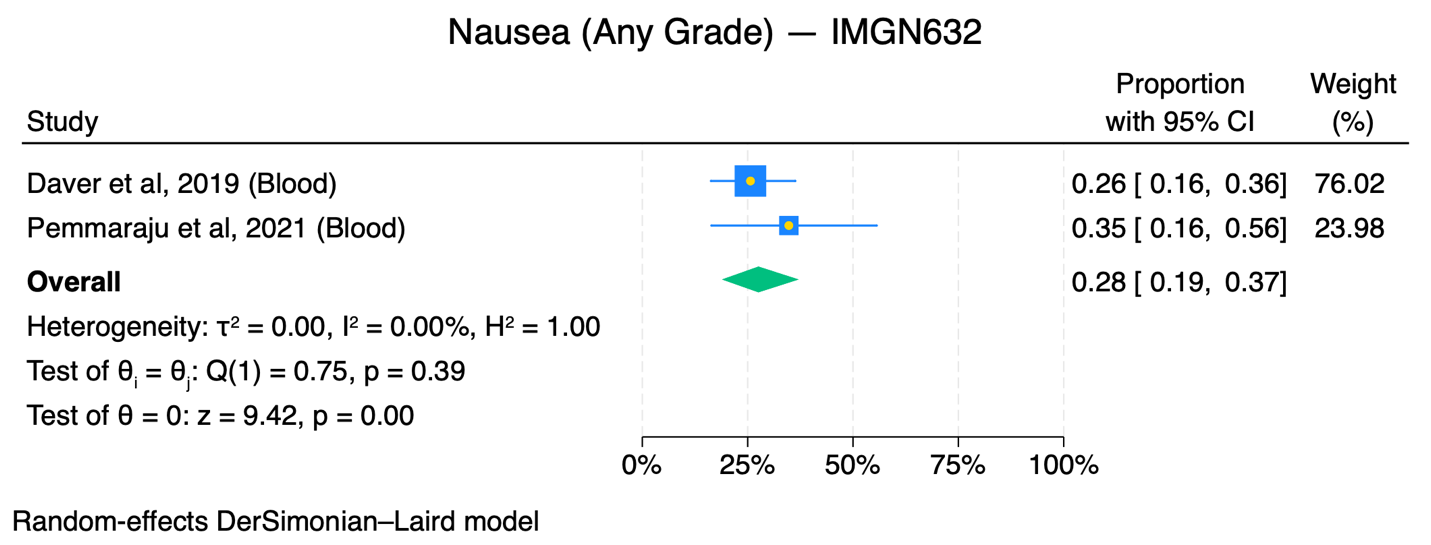


**3.17**


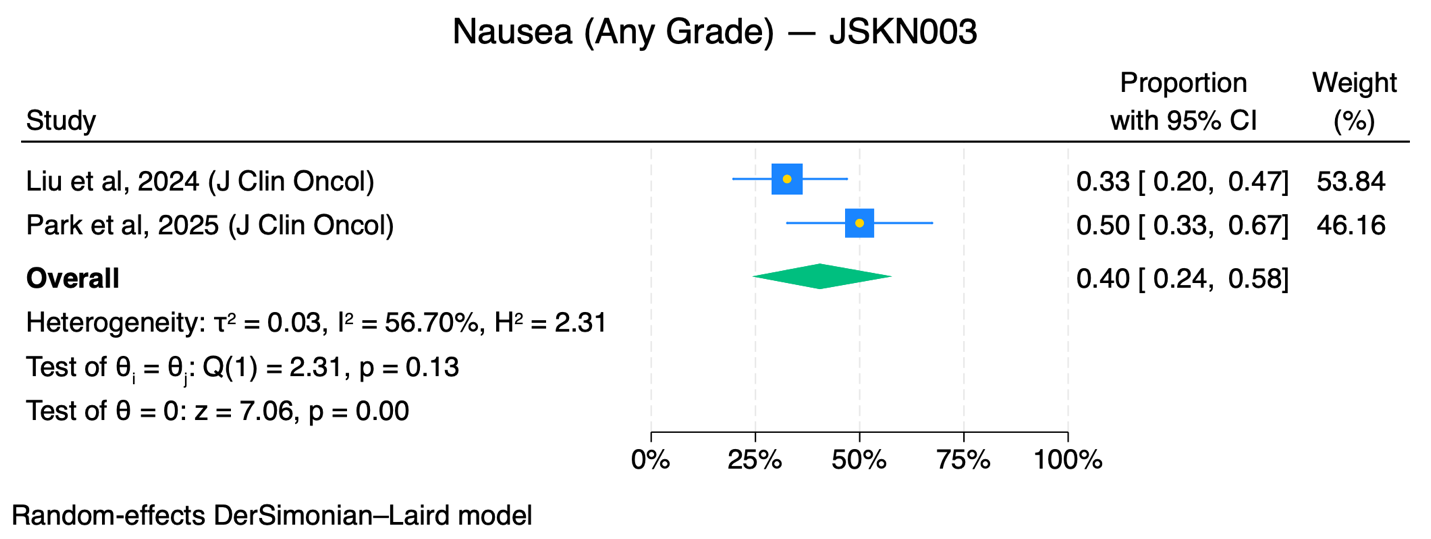


**3.18**


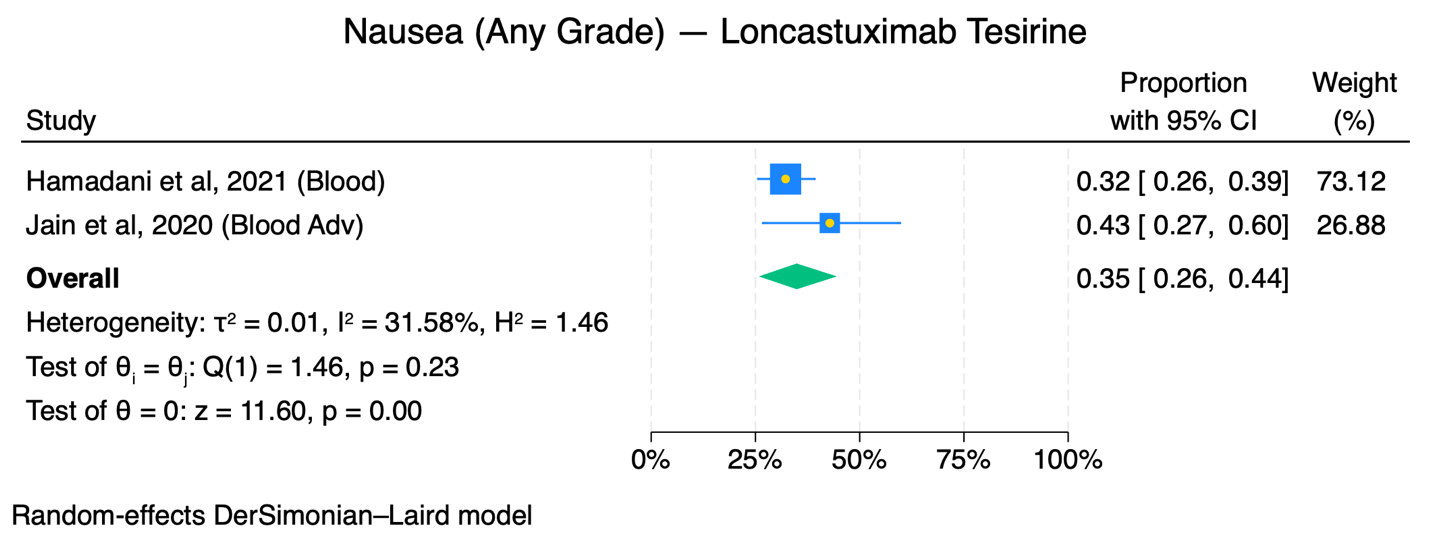


**3.19**


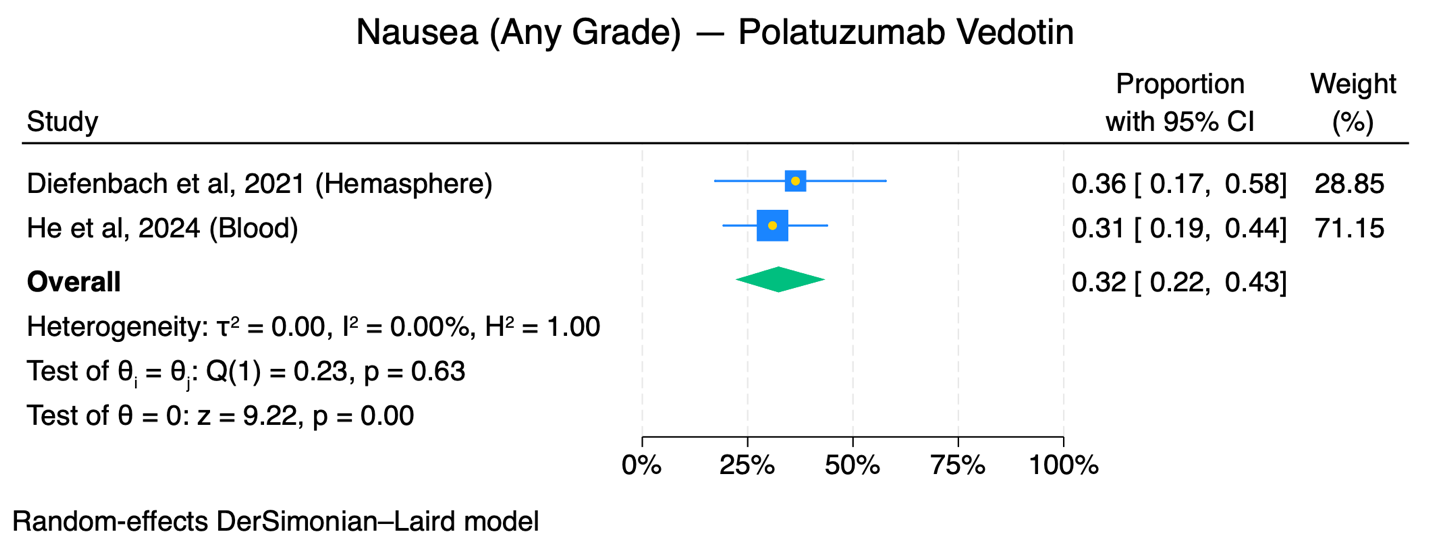


**3.20**


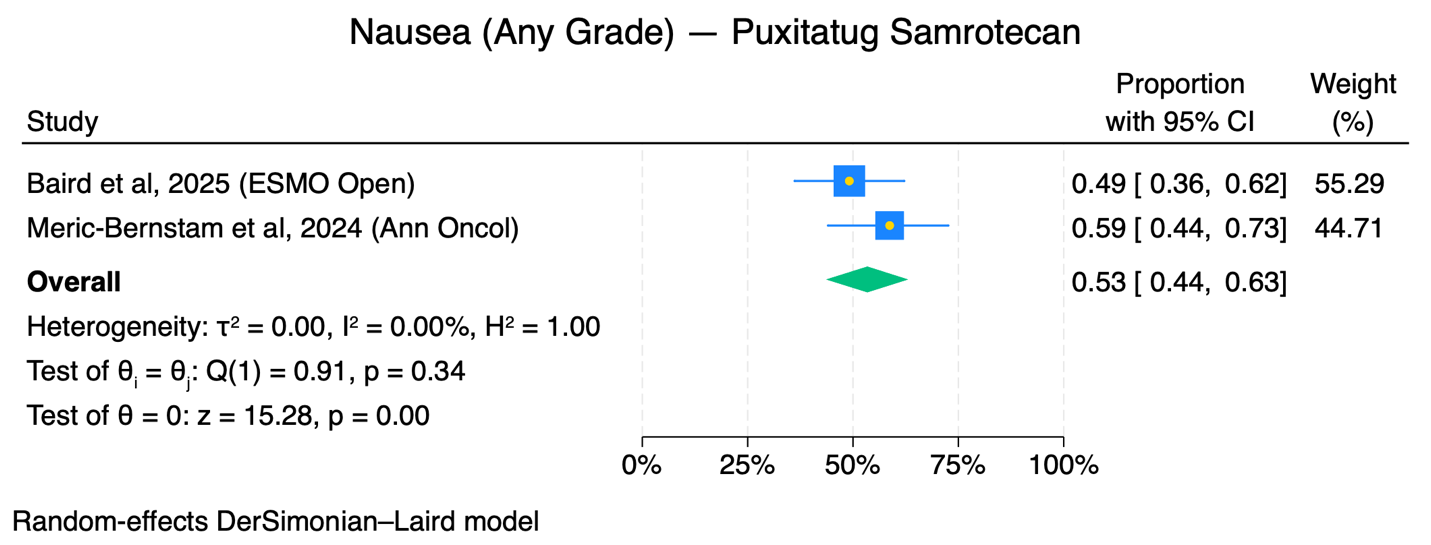


**3.21
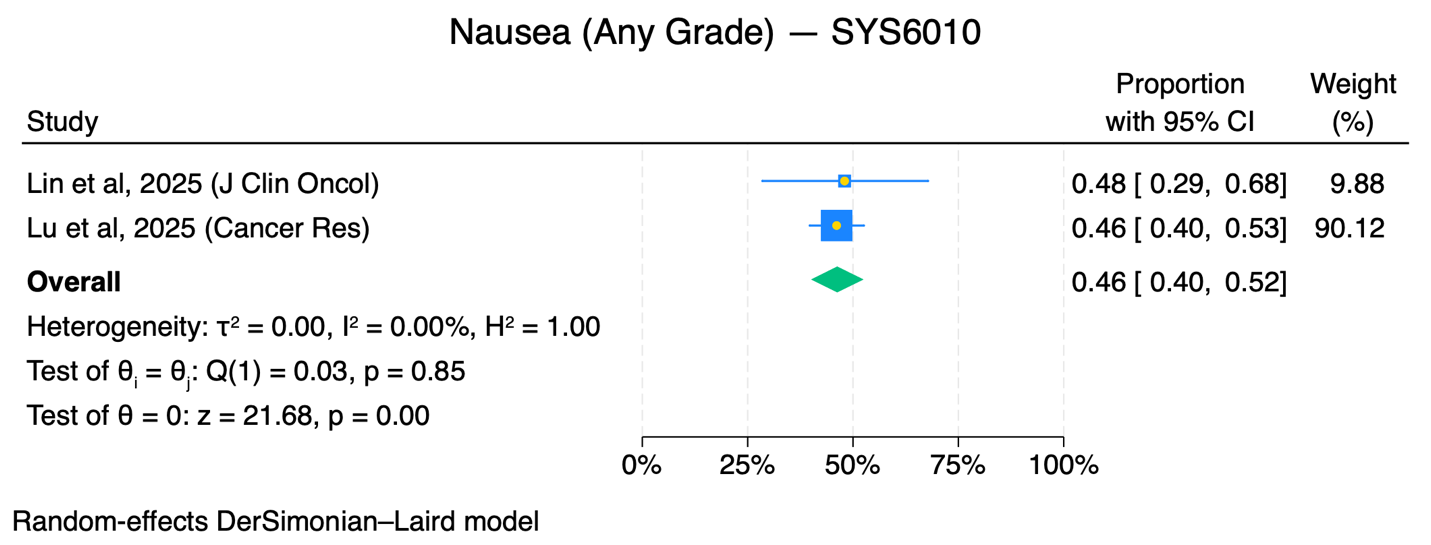
**

**3.22
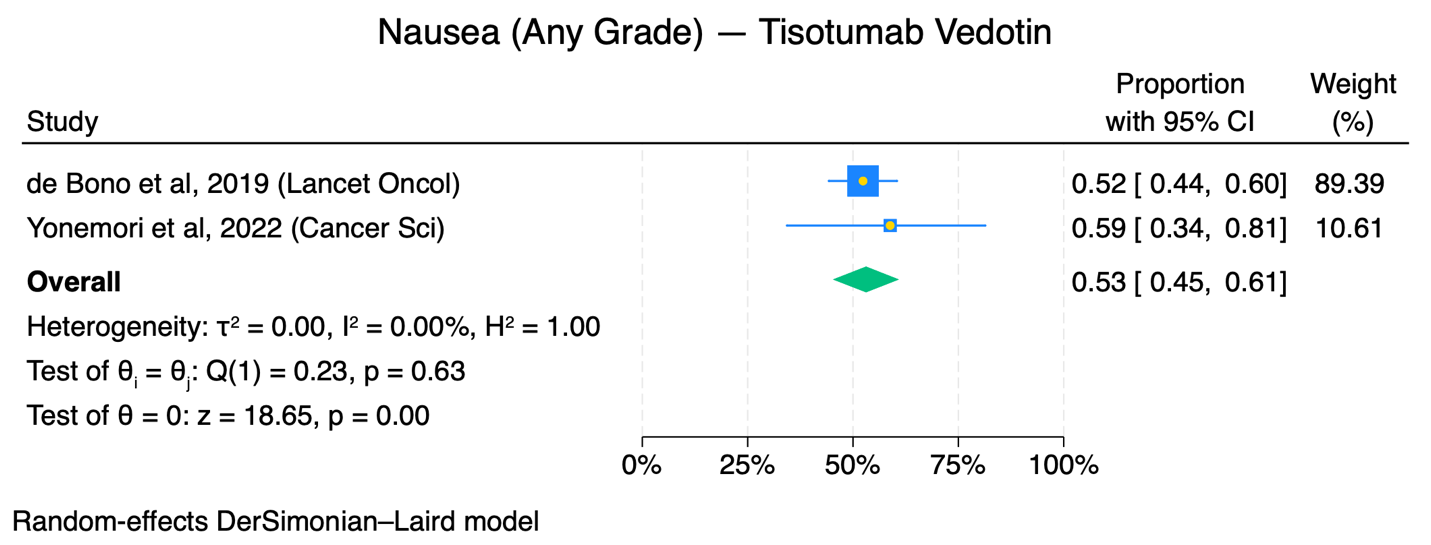
**

**3.23**


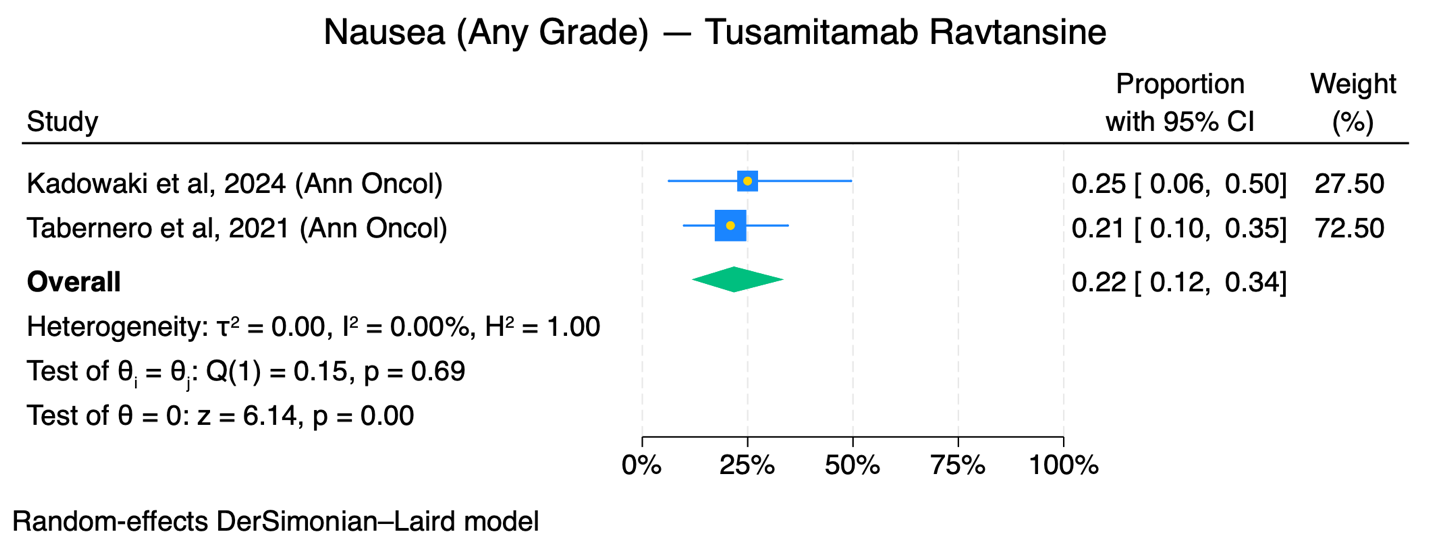


**3.24**


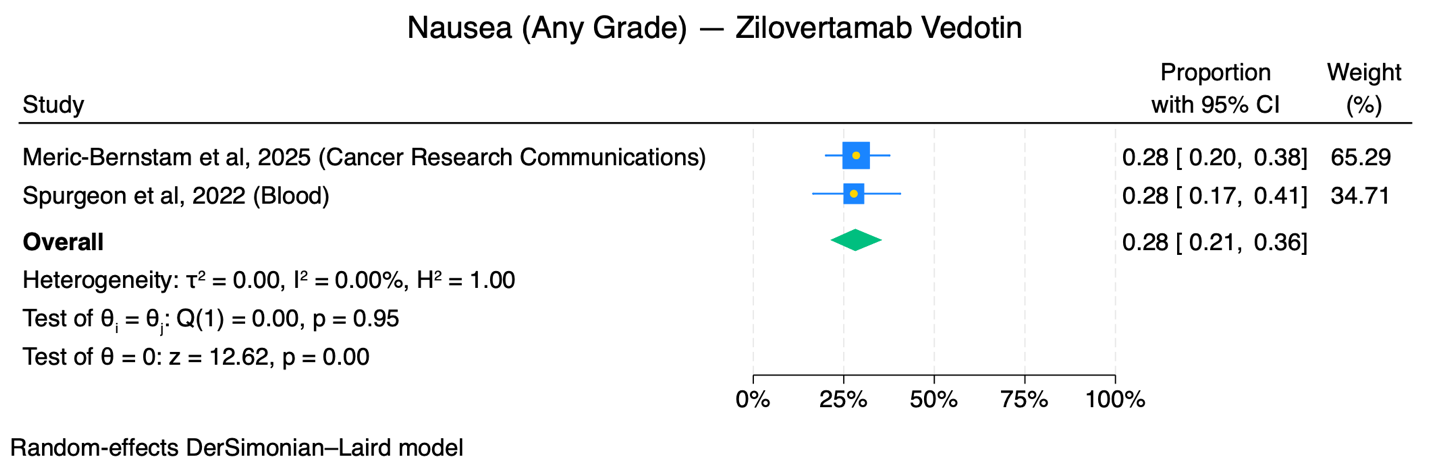


**Appendix 4.** Prevalence of Nausea by **4.1** Primary Cancer **4.2** Average Age of Patients (p < 0.001) **4.3** Percentage Female Patients (p = 0.072) **4.4** Number of Patients in Study (p = 0.207) **4.5** Follow-Up Duration of Study (p = 0.020)

**4.1**

**
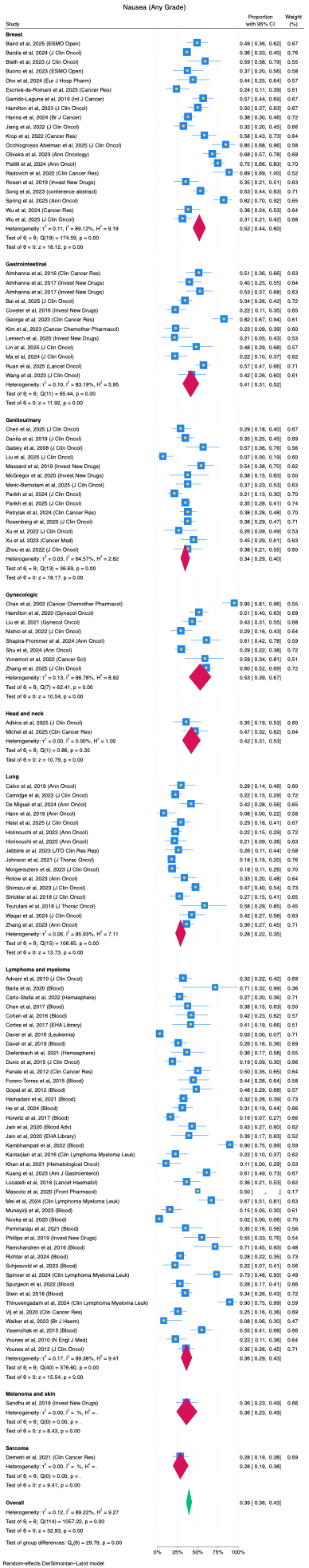
**

**4.2**

**
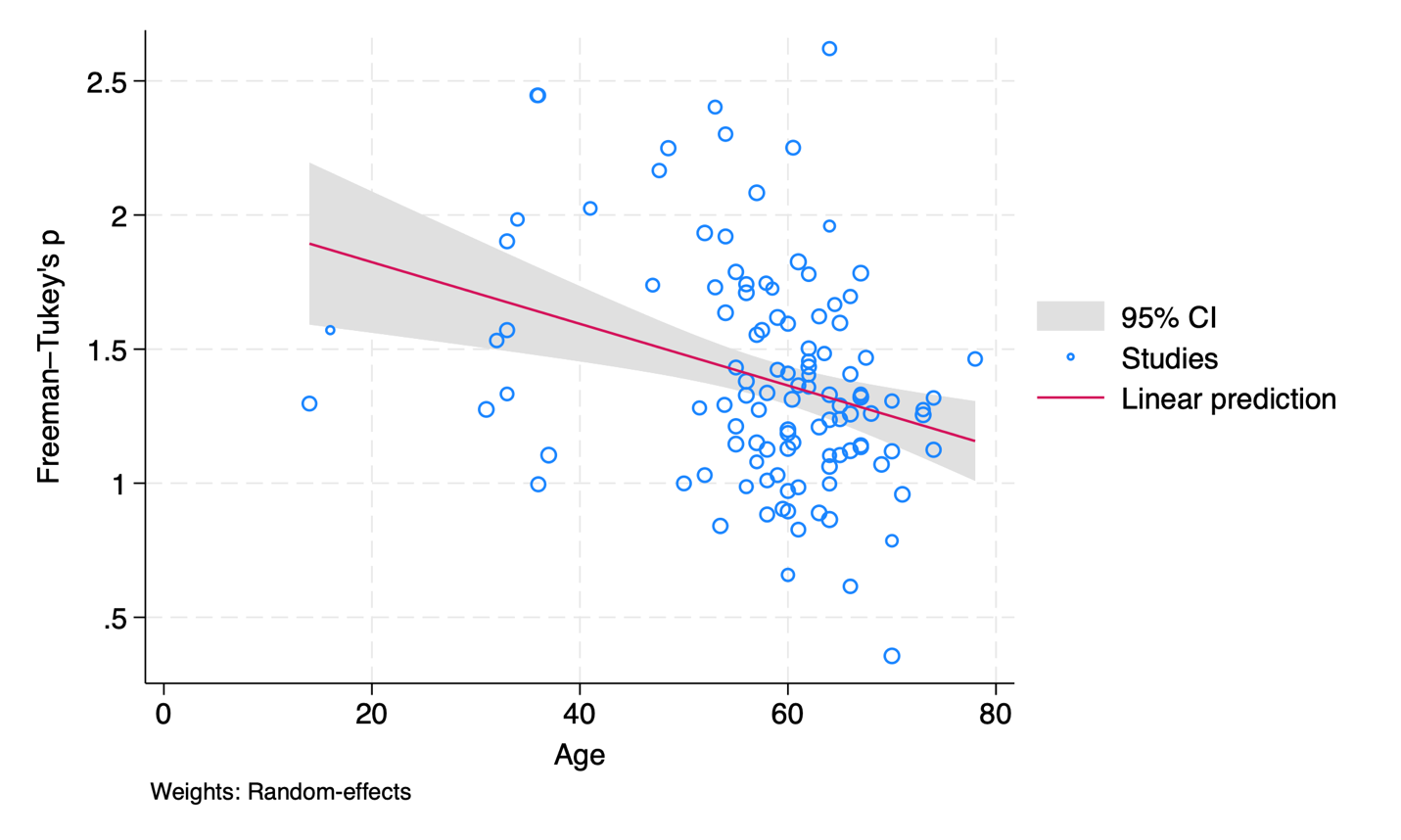
**

**4.3**

**
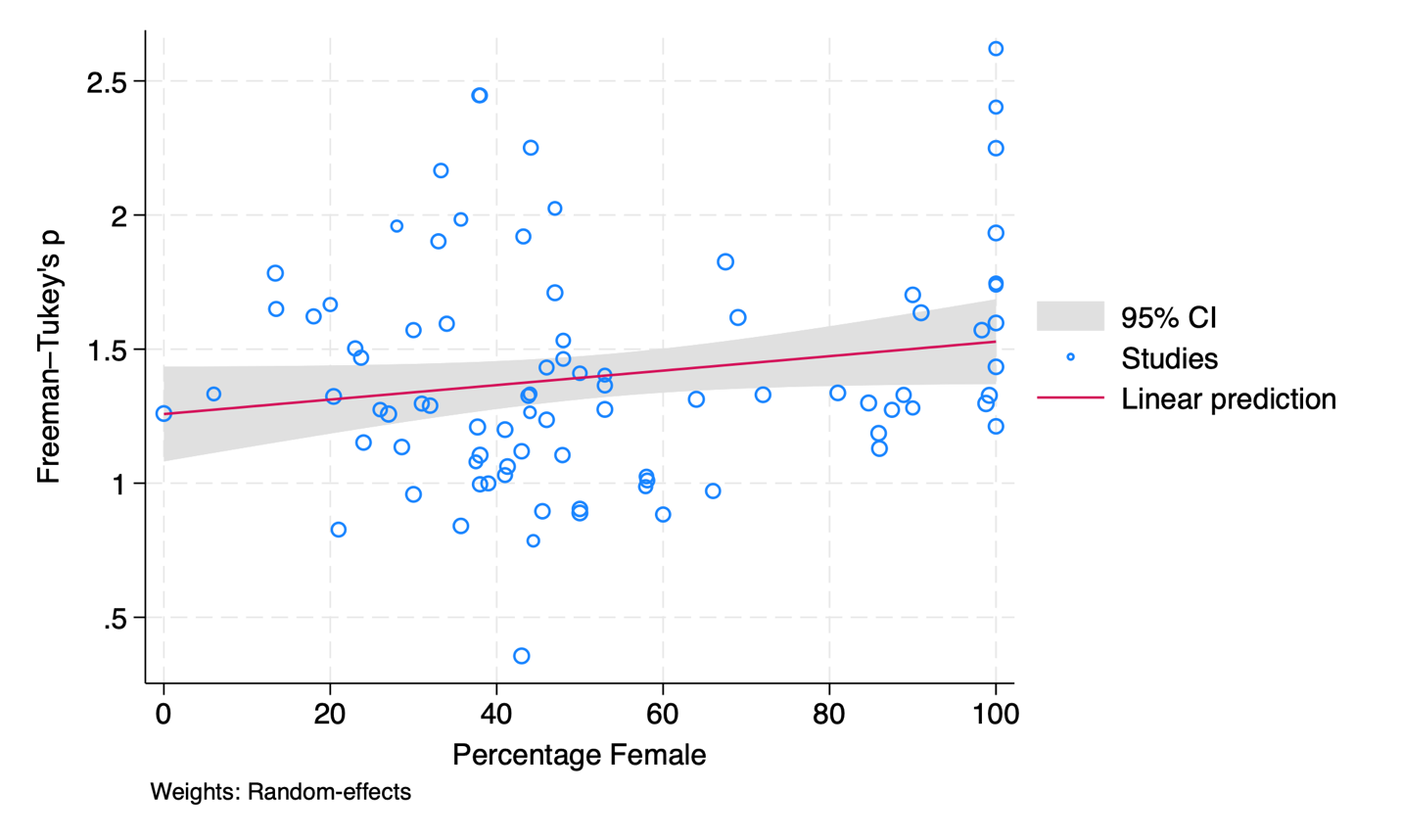
**

**4.4**

**
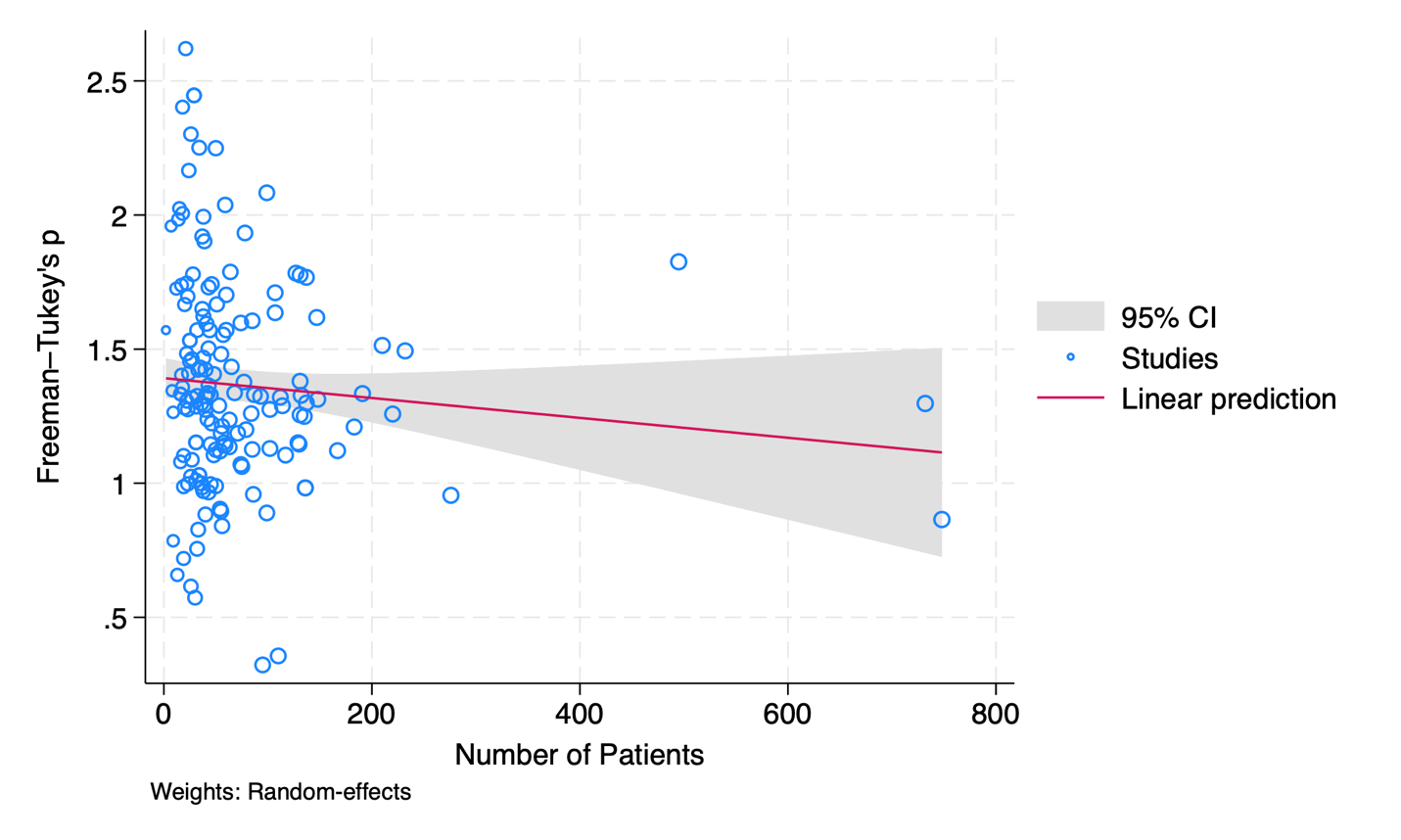
**

**4.5**

**
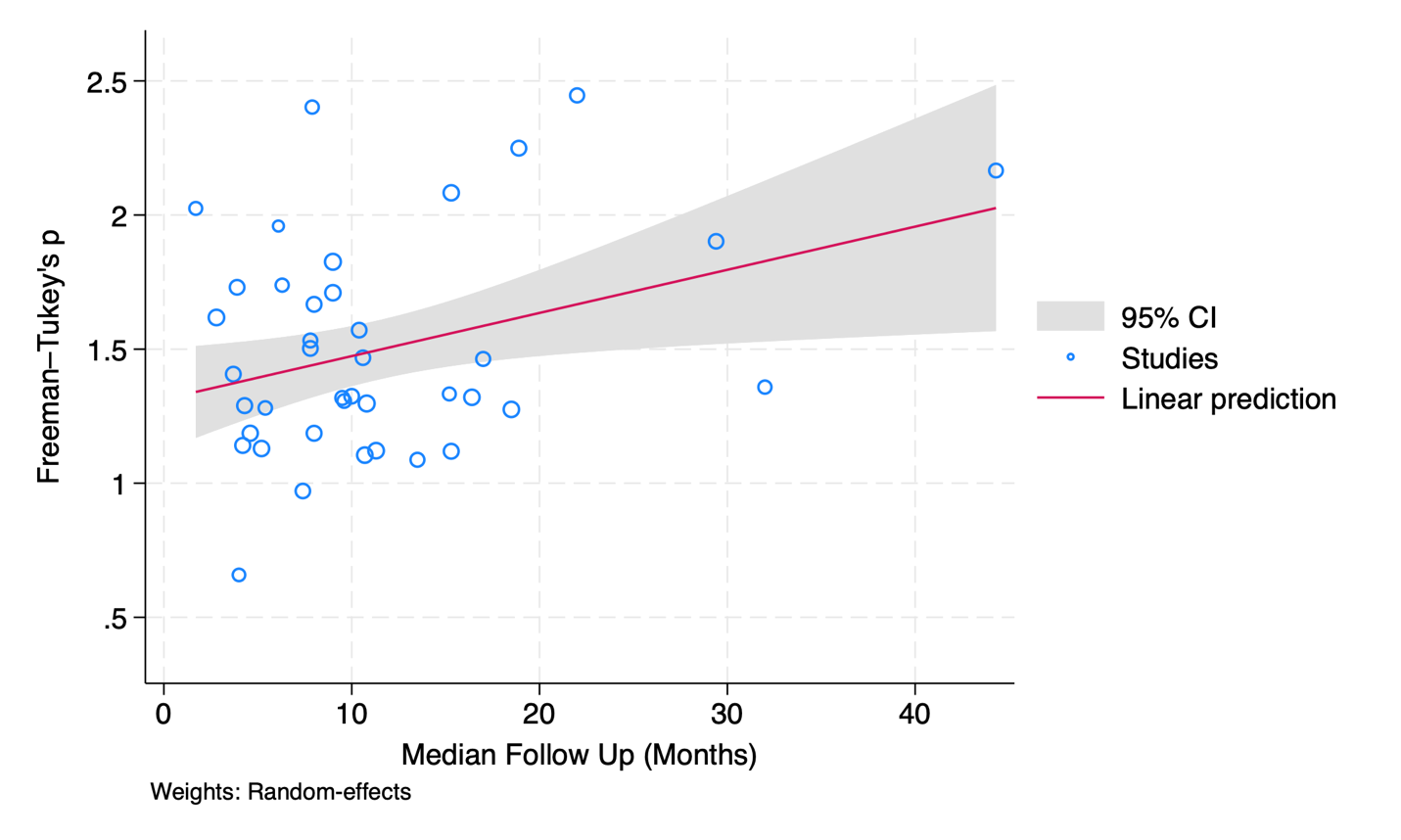
**

**Appendix 5.** Prevalence of Severe Nausea by Antibody Drug Conjugate **5.1** BrentuximabVedotin **5.2** Datopotamab Deruxtecan **5.3** BL-B01D1 **5.4** Patritumab Deruxtecan **5.5** Sacituzumab Govitecan **5.6** Tak-264 **5.7** Trastuzumab Deruxtecan **5.8** Cofetuzumab Pelidotin **5.9** IMGN632 **5.10** JSKN003 **5.11** Telisotuzumab Vedotin

**5.1**


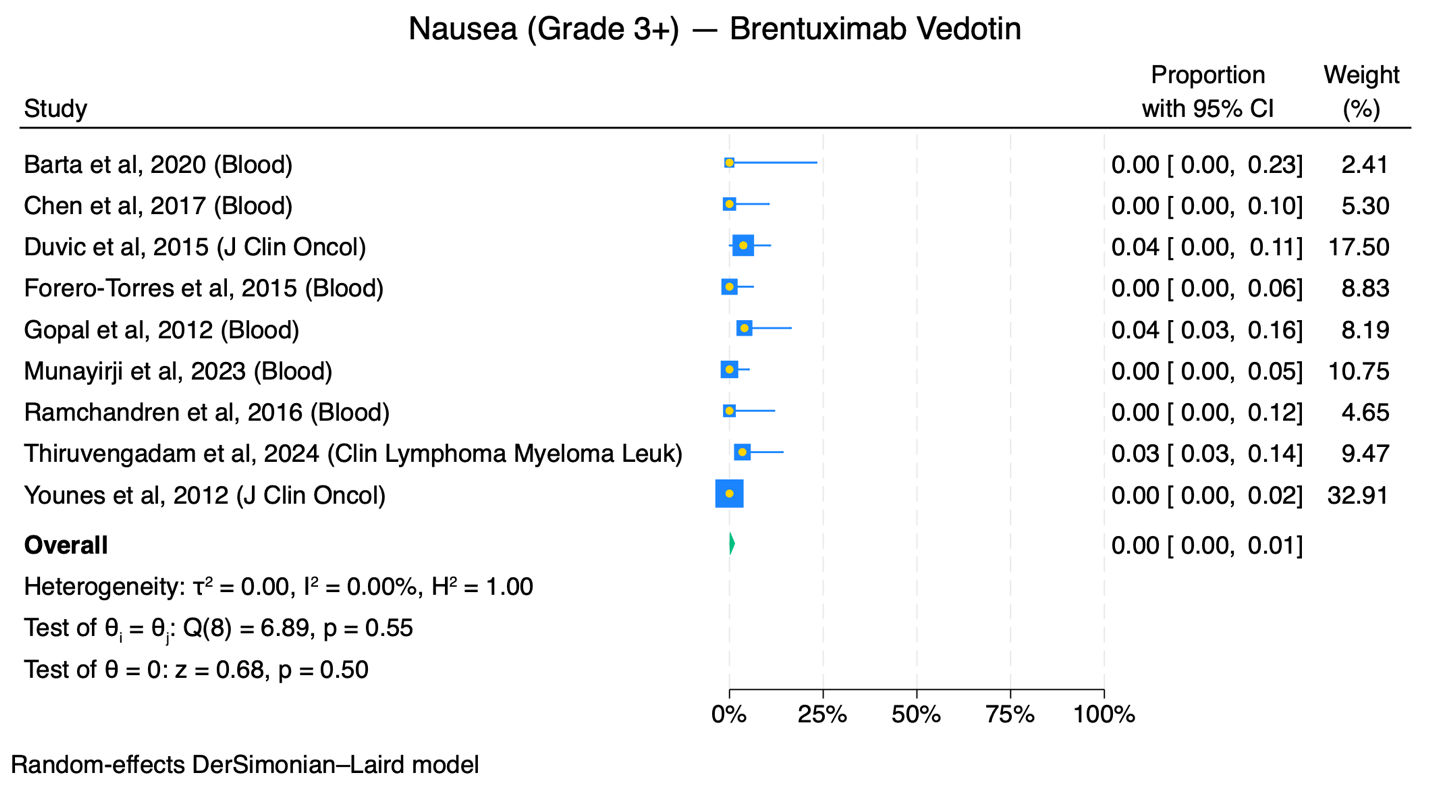


**5.2**


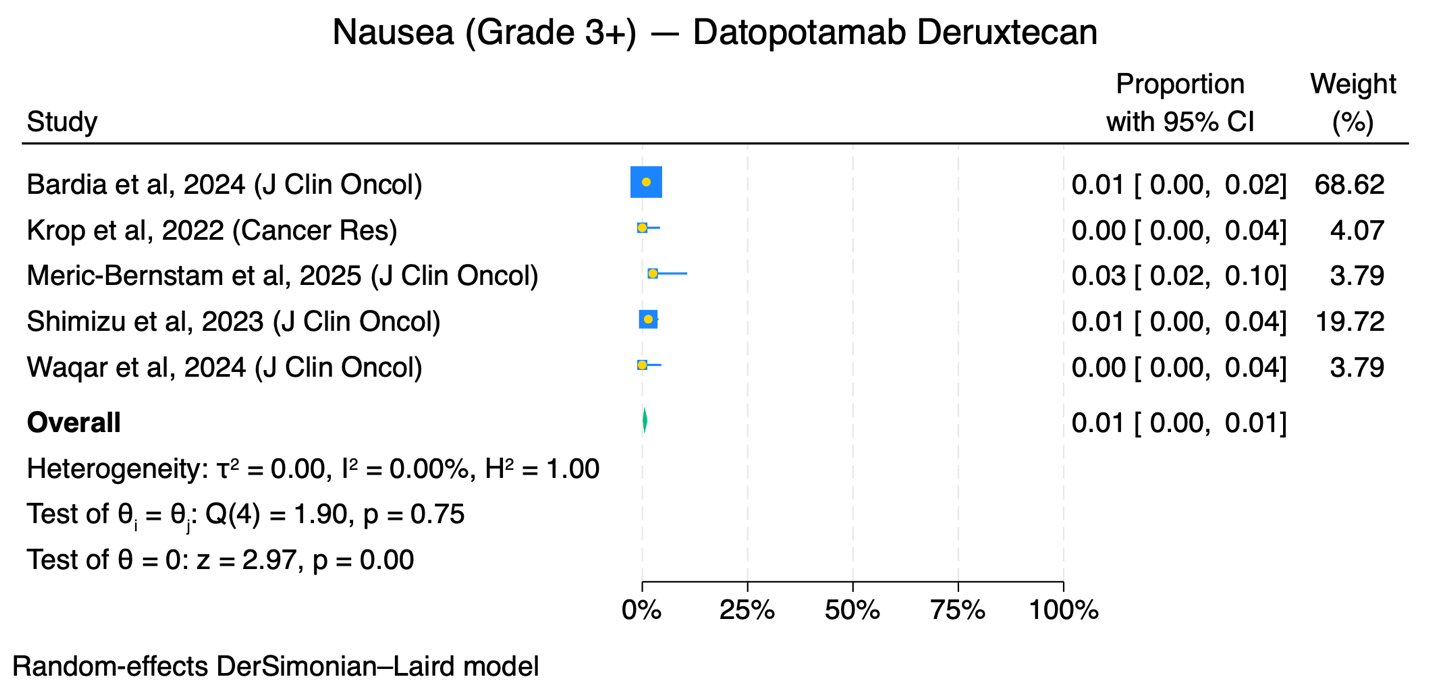


**5.3**


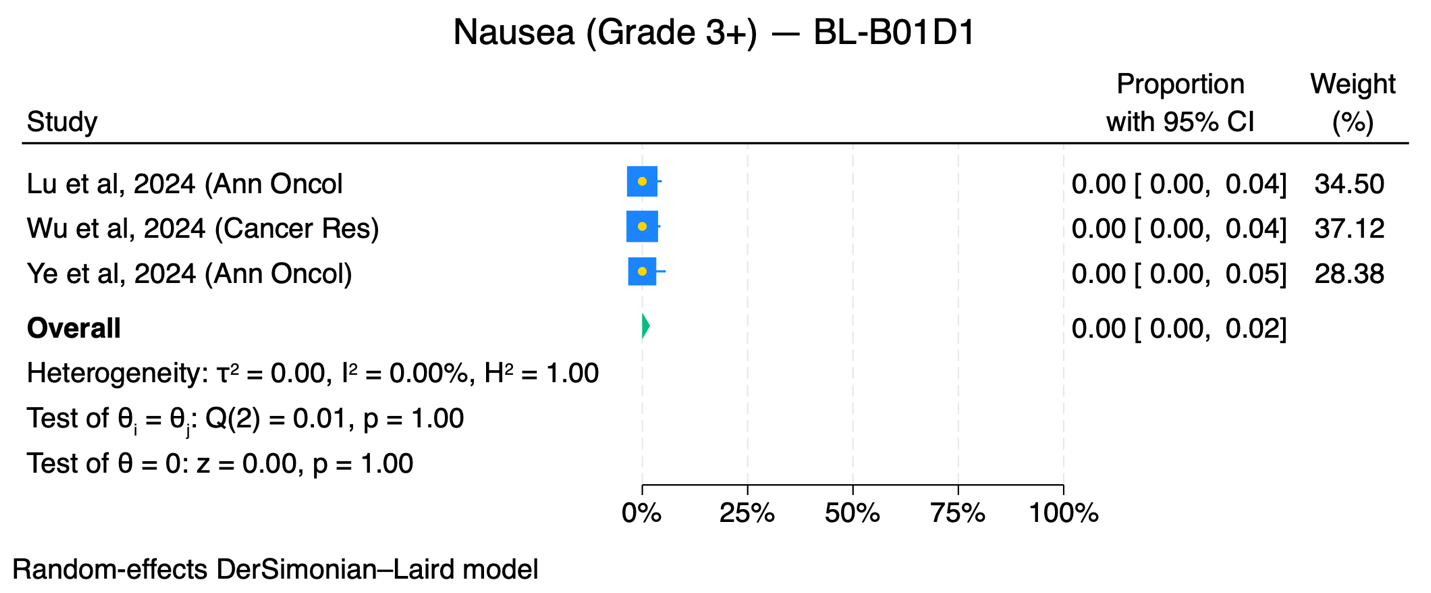


**5.4**


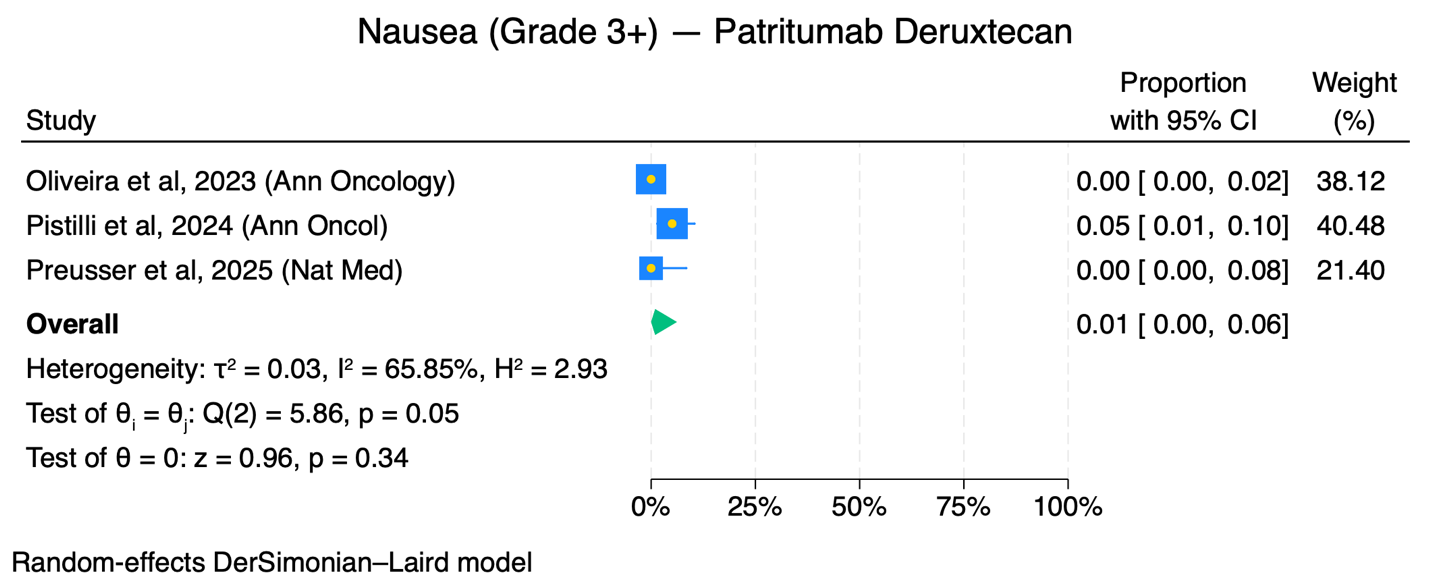


**5.5**


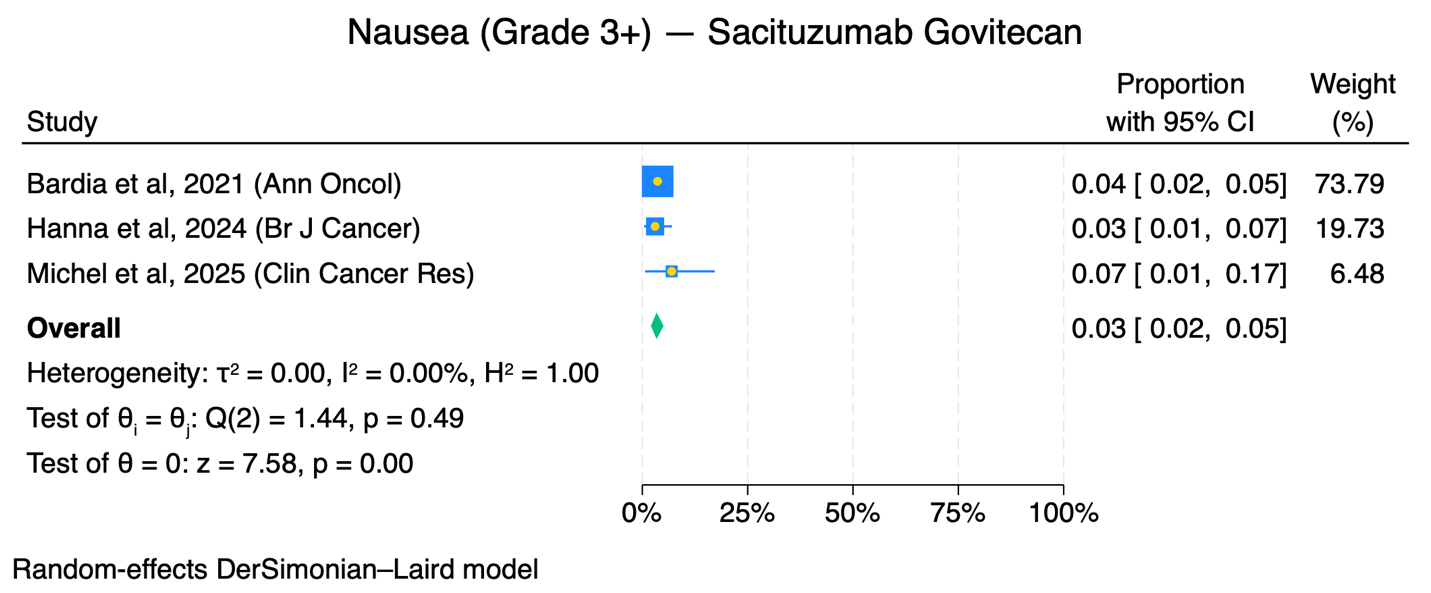


**5.6**


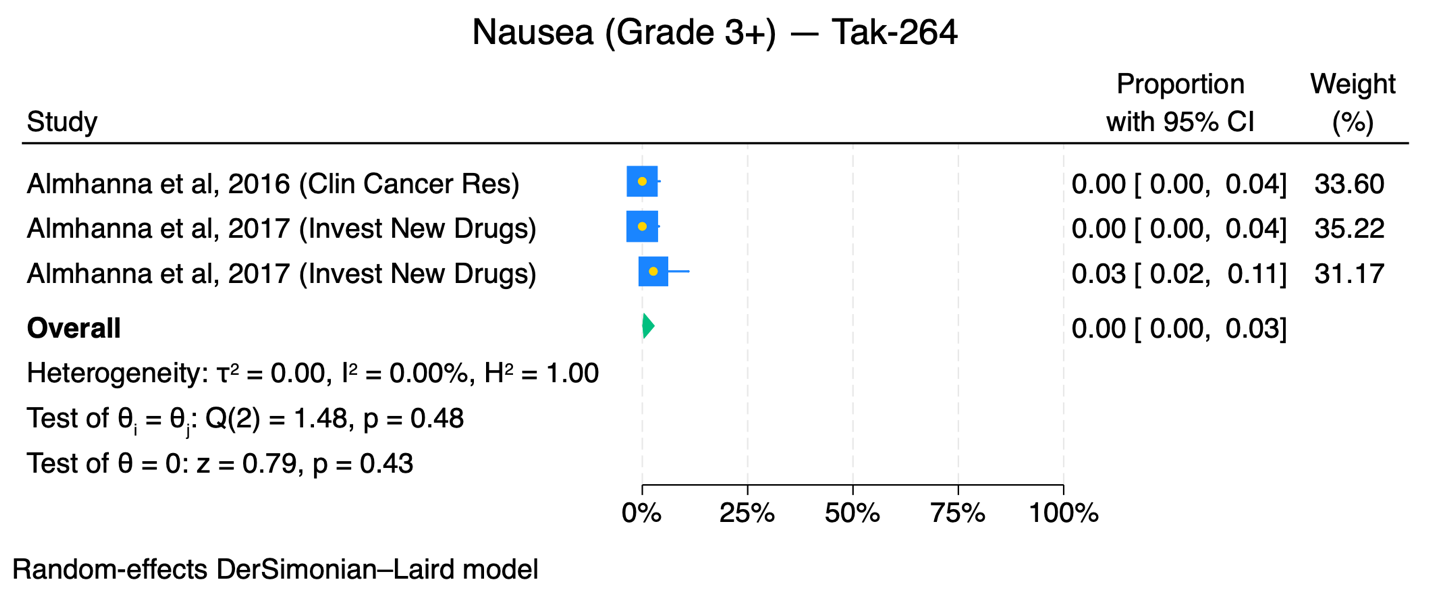


**5.7**


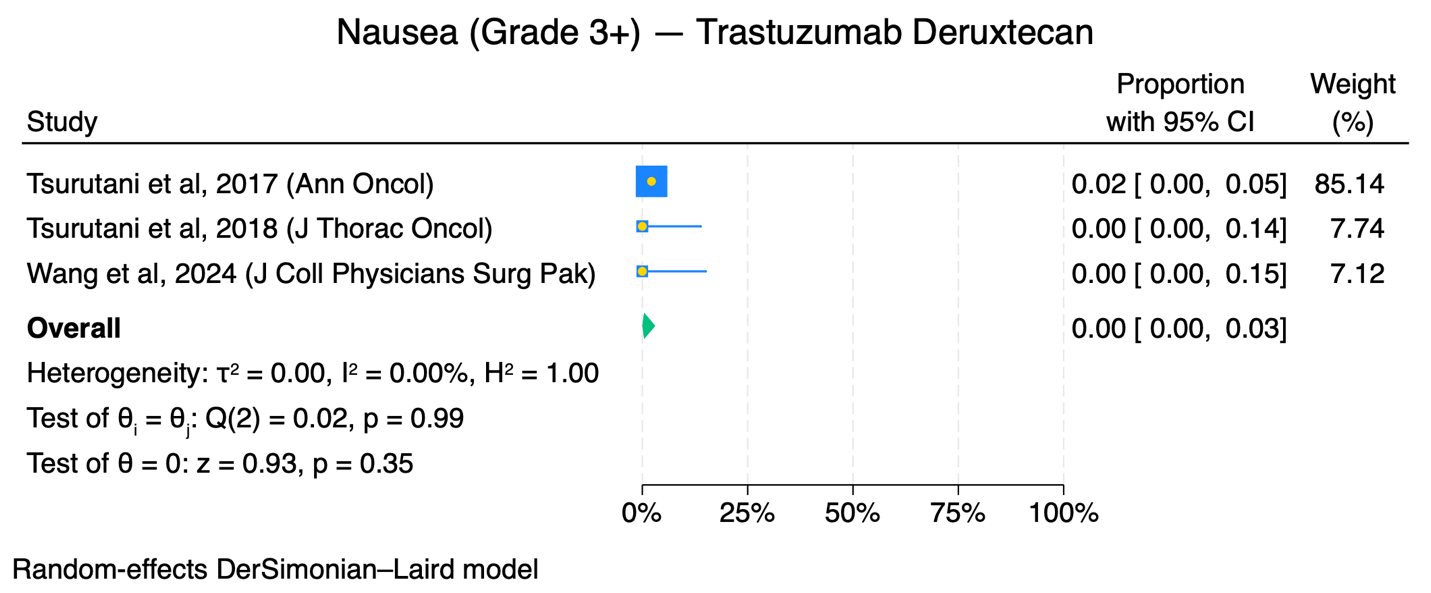


**5.8**


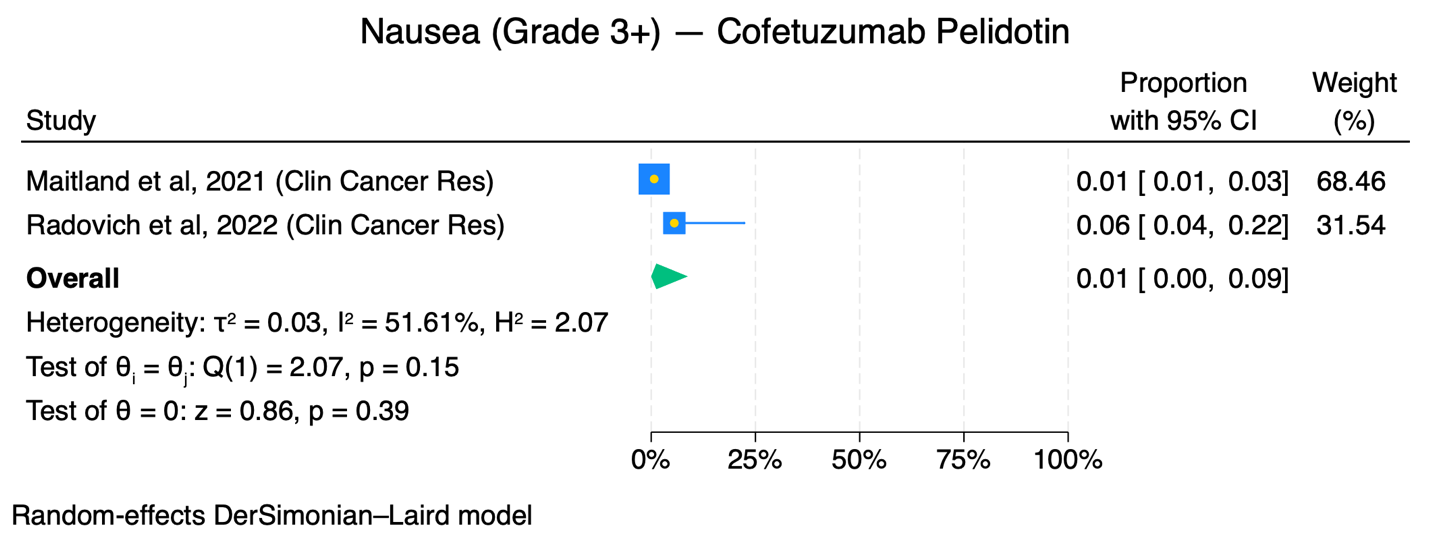


**5.9**


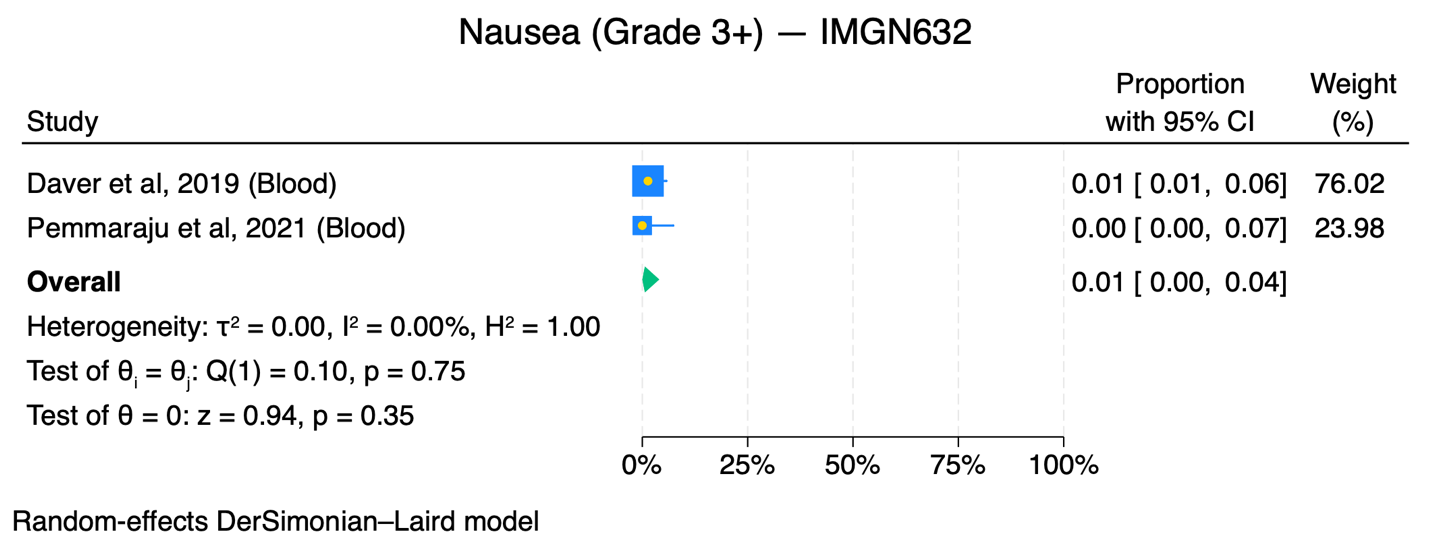


**5.10**


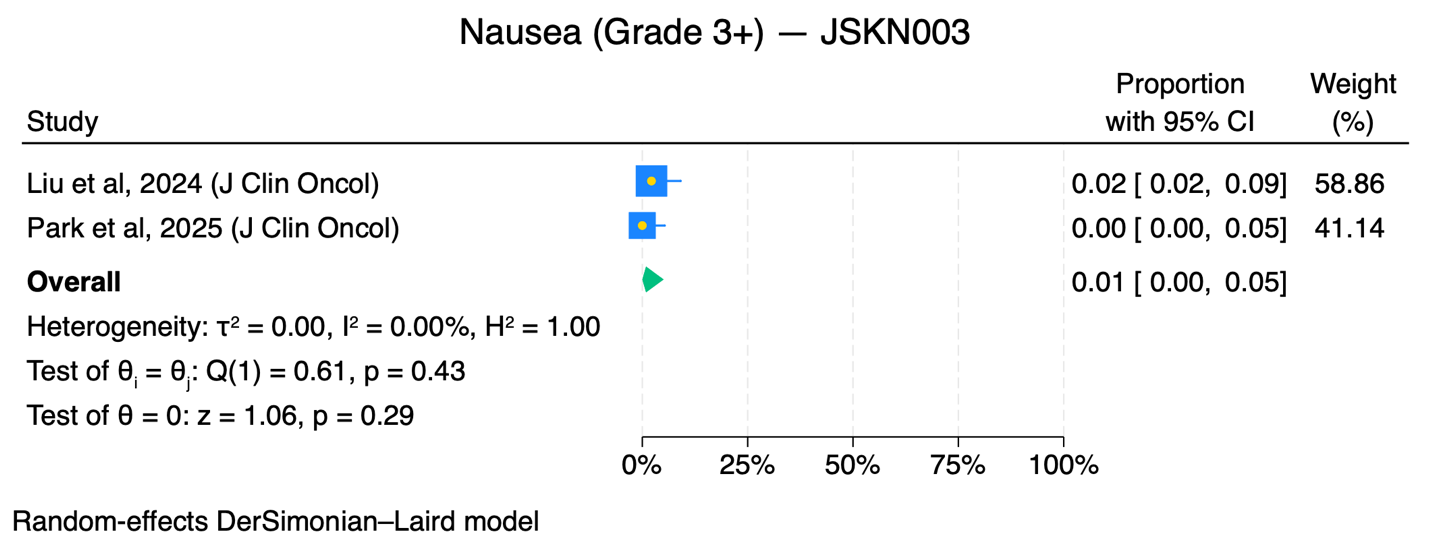


**5.11**


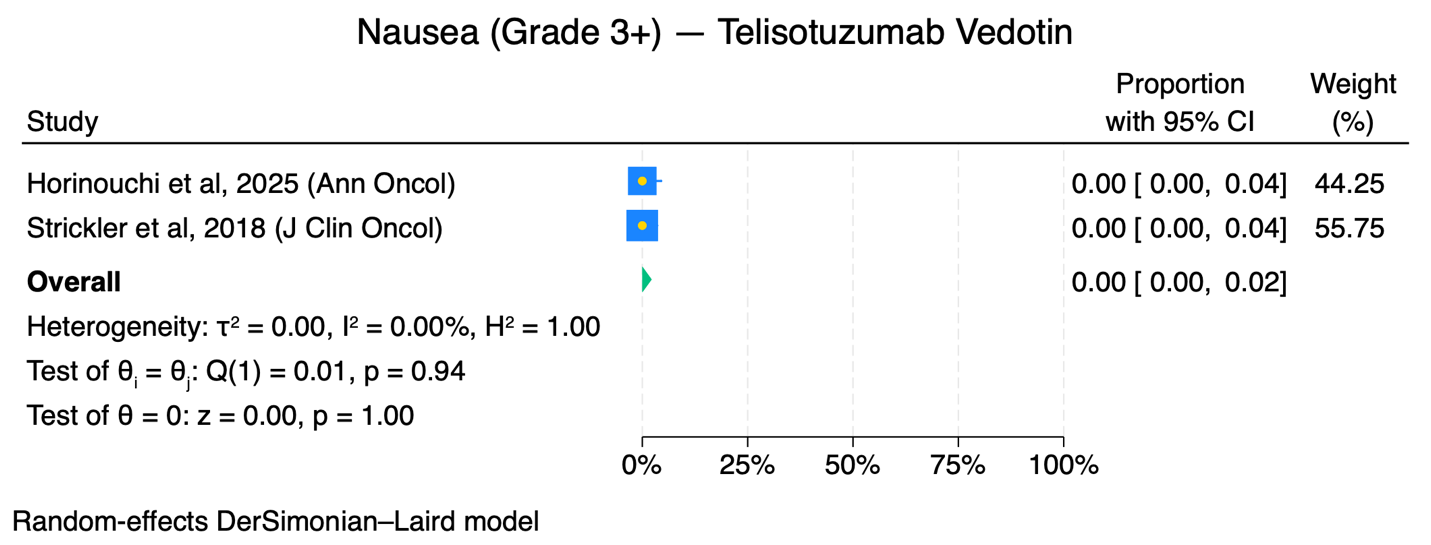


**Appendix 6.** Prevalence of Severe Nausea by **6.1** Primary Cancer **6.2** Average Age of Patients (p = 0.991) **6.3** Percentage Female Patients (p = 0.659) **6.4** Number of Patients in Study (p = 0.137) **6.5** Follow-Up Duration of Study (p = 0.612)

**6.1**

**
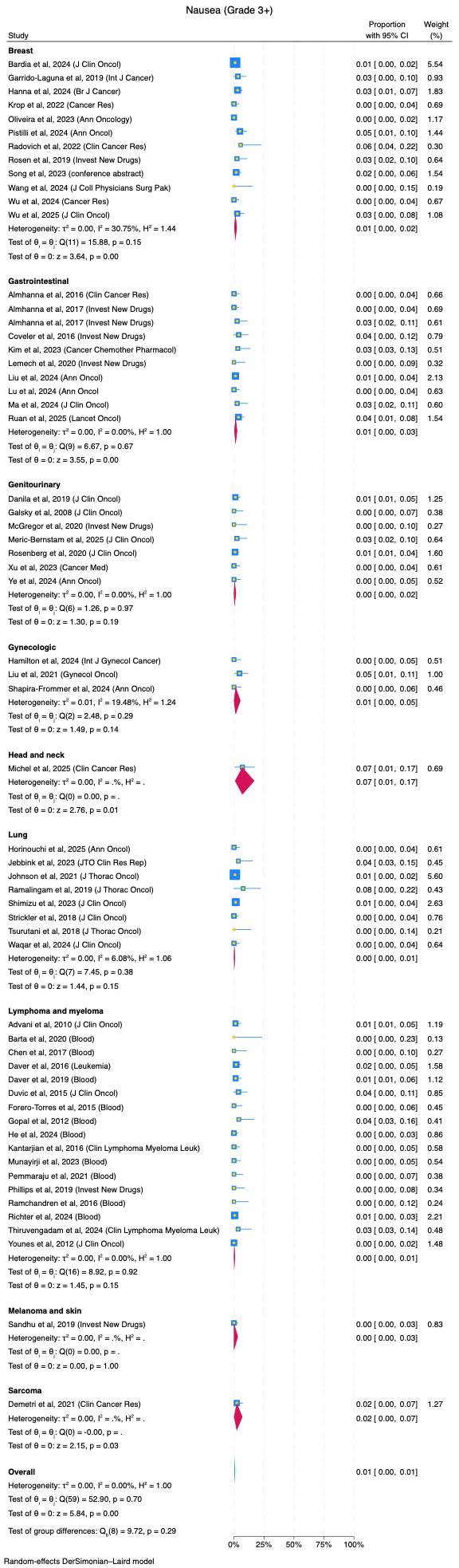
**

**6.2**

**
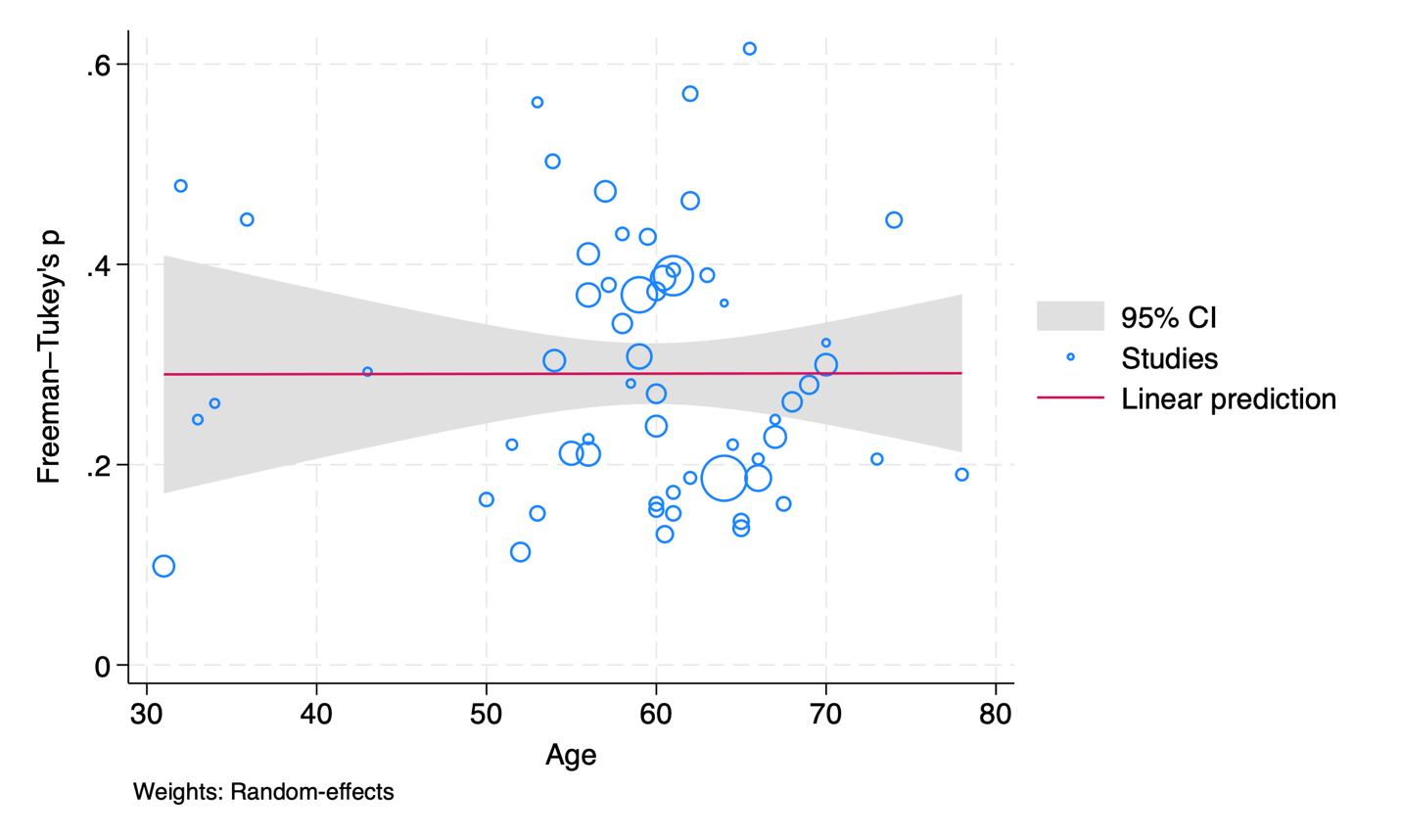
**

**6.3**

**
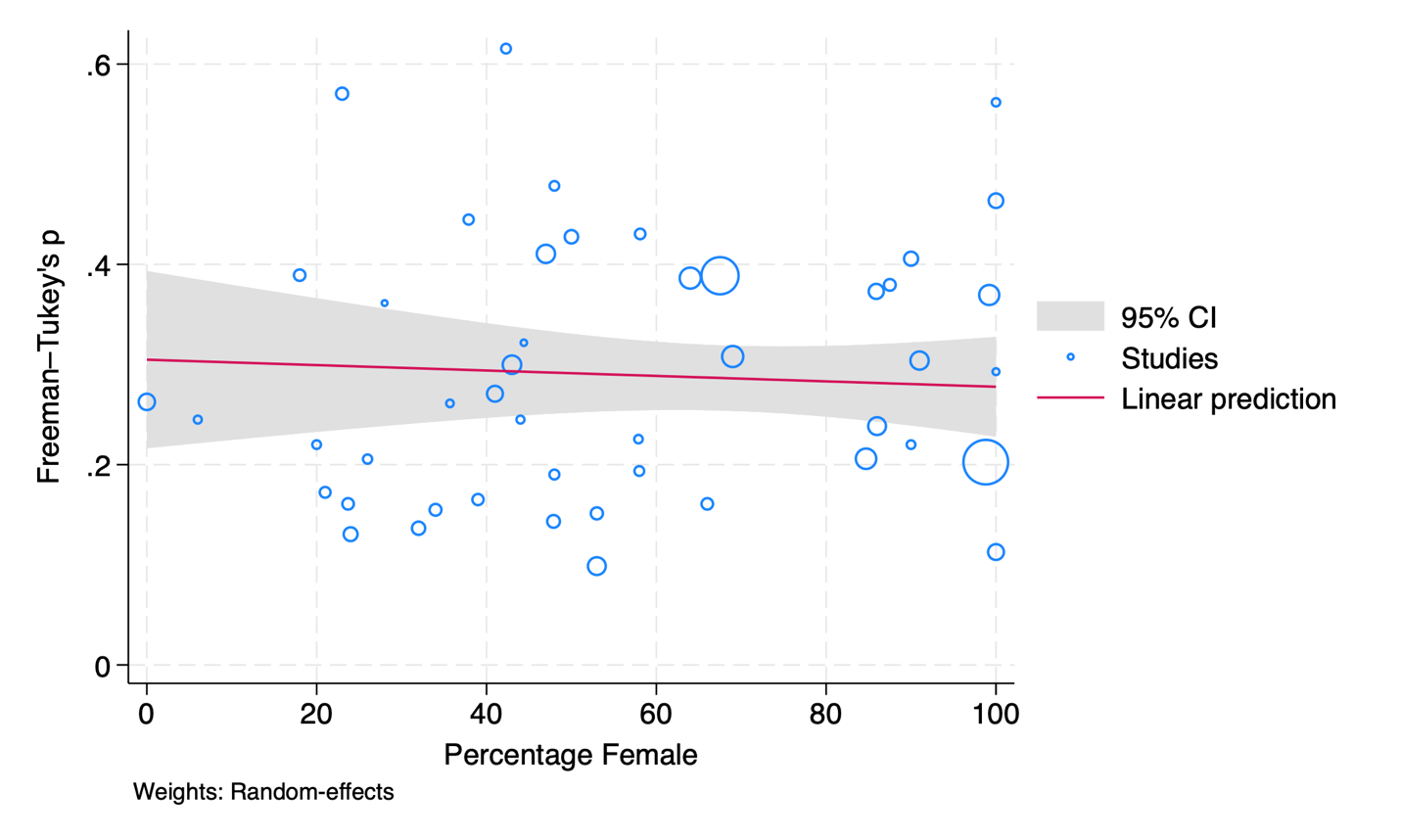
**

**6.4**

**
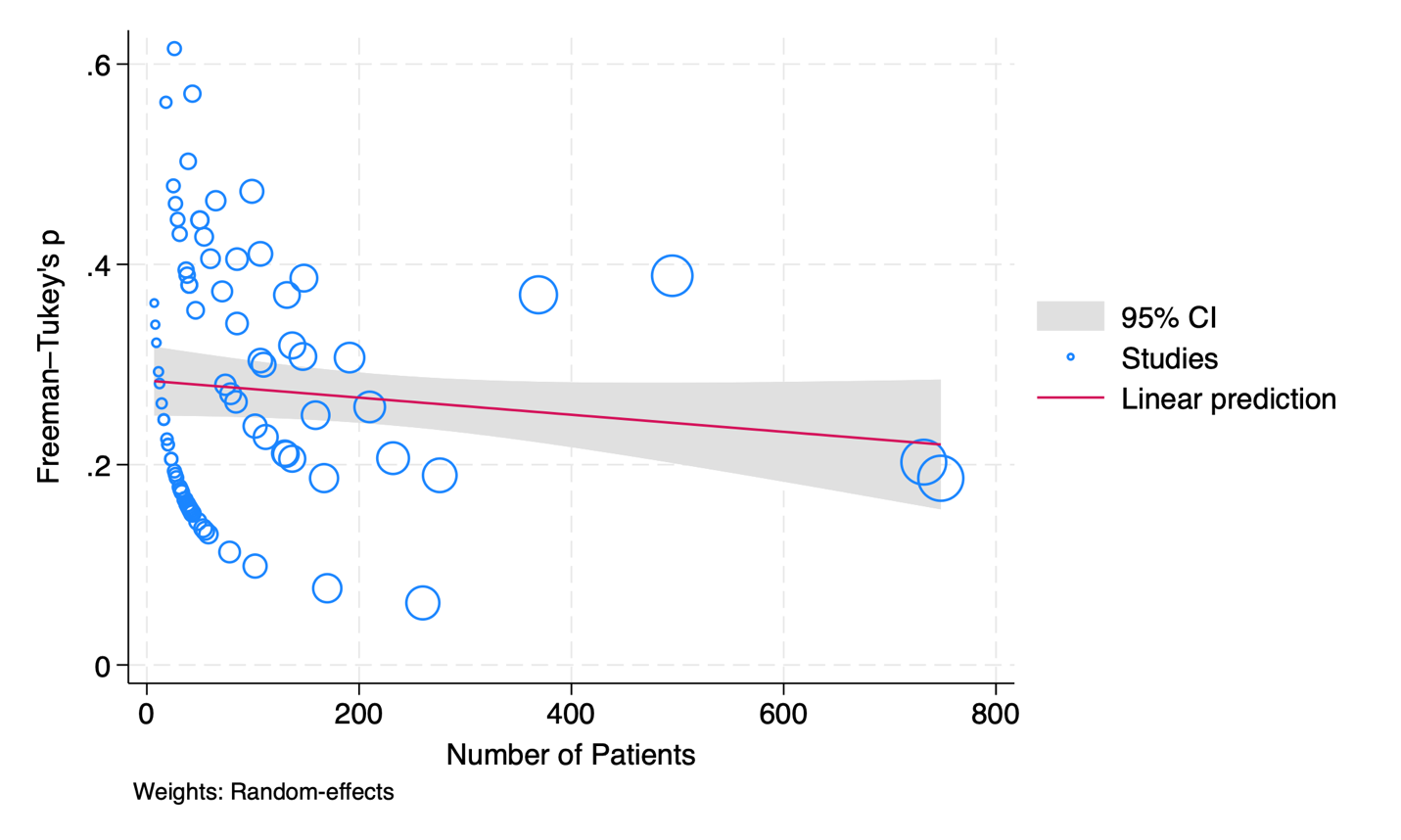
**

**6.5**

**
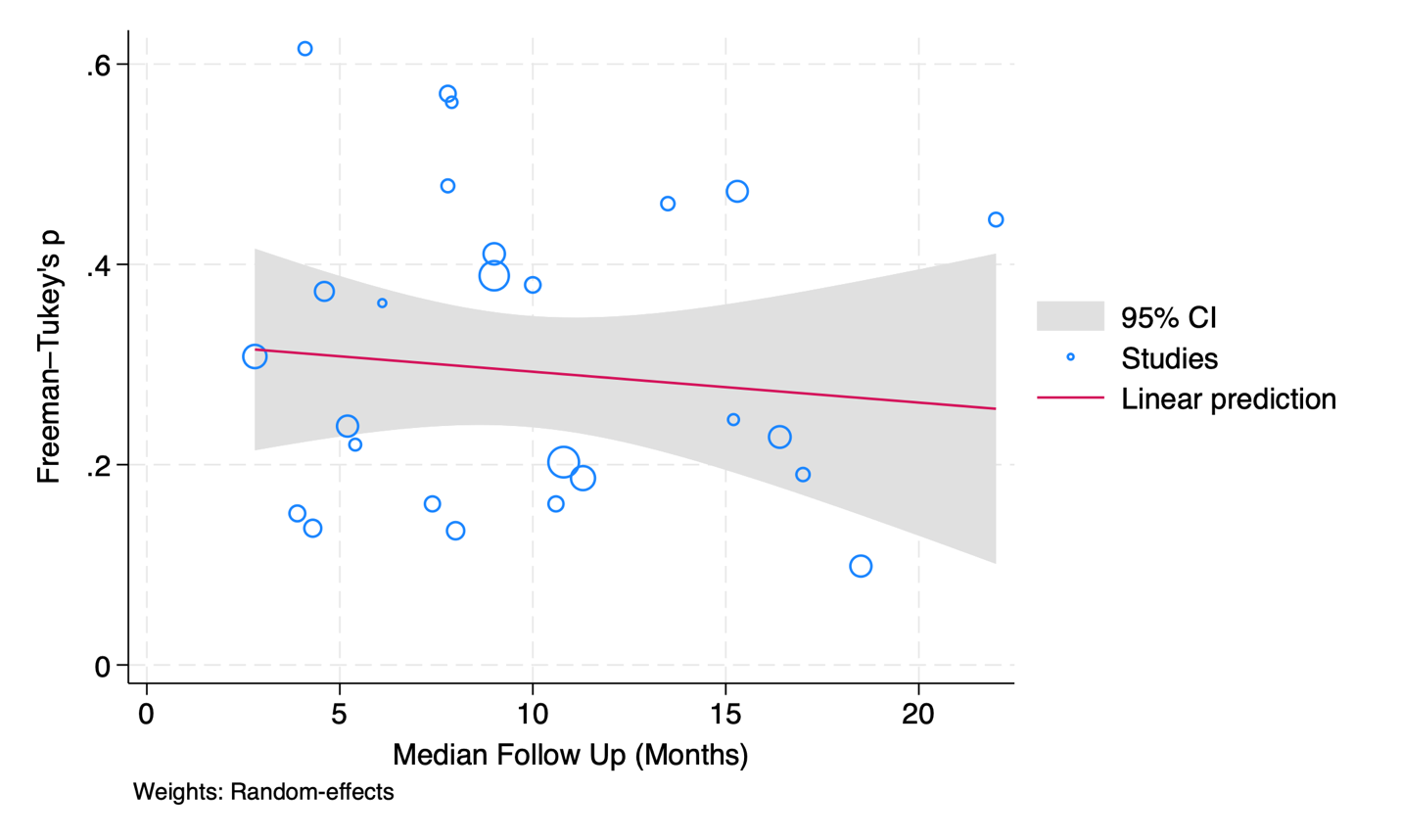
**

**Appendix 7.** Prevalence of Vomiting by Antibody Drug Conjugate **7.1** Brentuximab **7.2** Sacituzumab Govitecan **7.3** Datopotamab Deruxtecan **7.4** Patritumab Deruxtecan **7.5** Tak-264 **7.6** BL-B01D1 **7.7** Cofetuzumab Pelidotin **7.8** Loncastuximab Tesirine **7.9** Tisotumab Vedotin

**7.1**


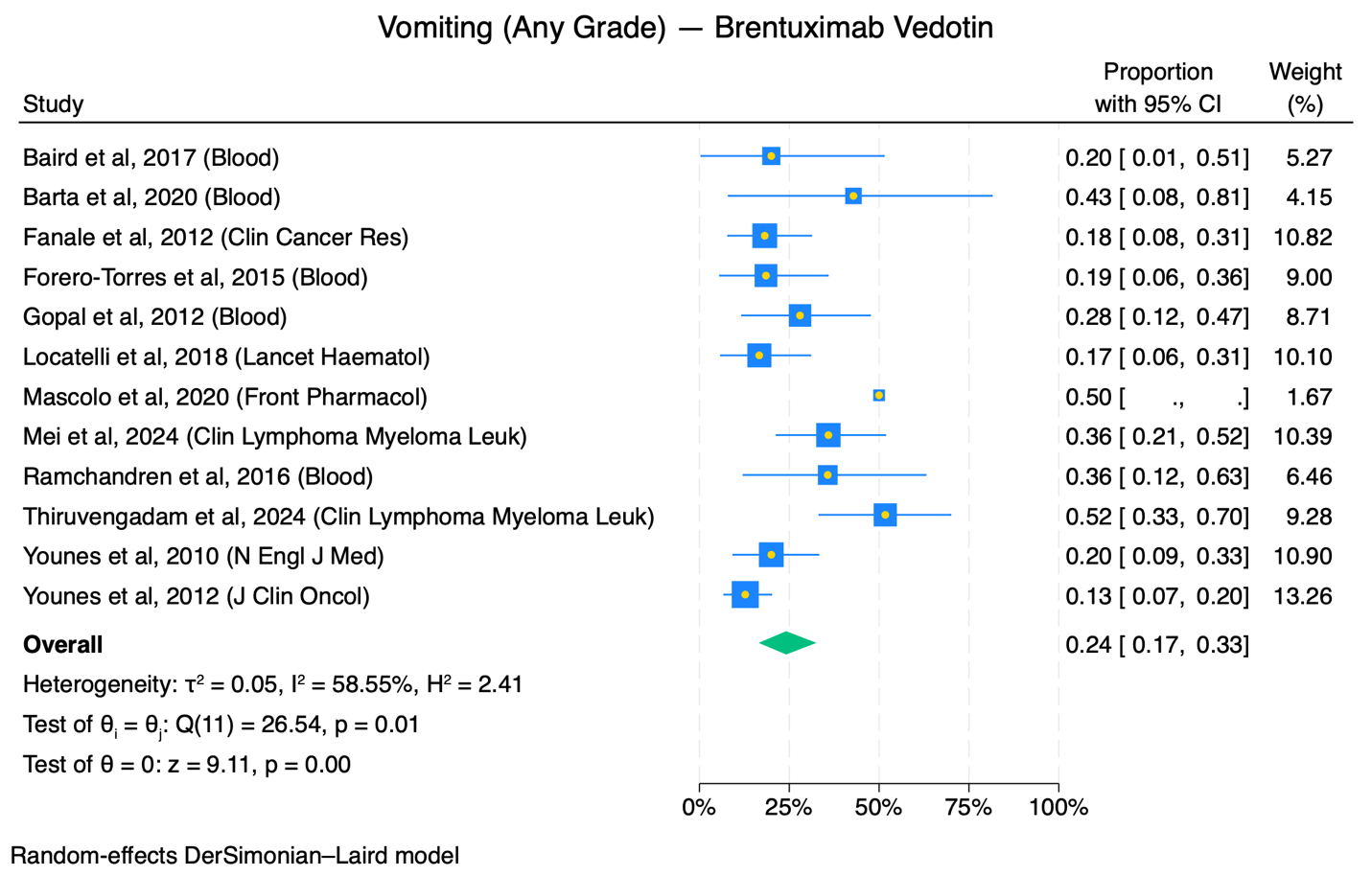


**7.2**


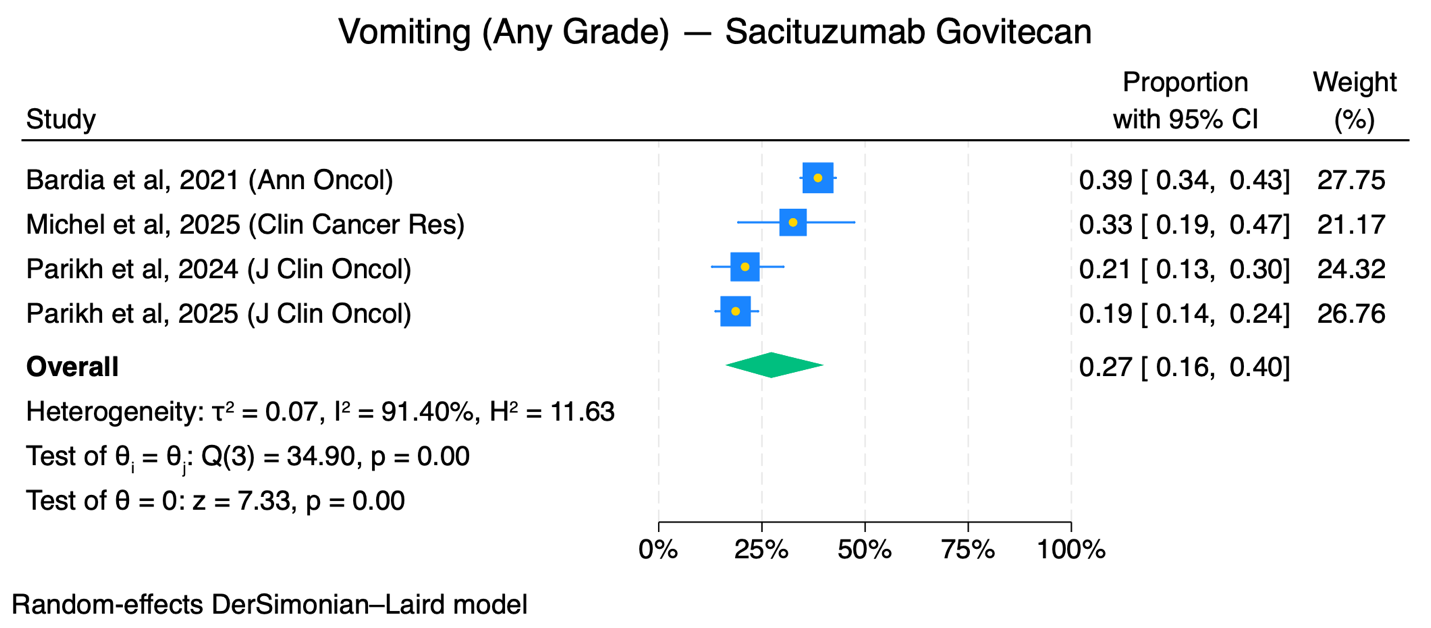


**7.3**


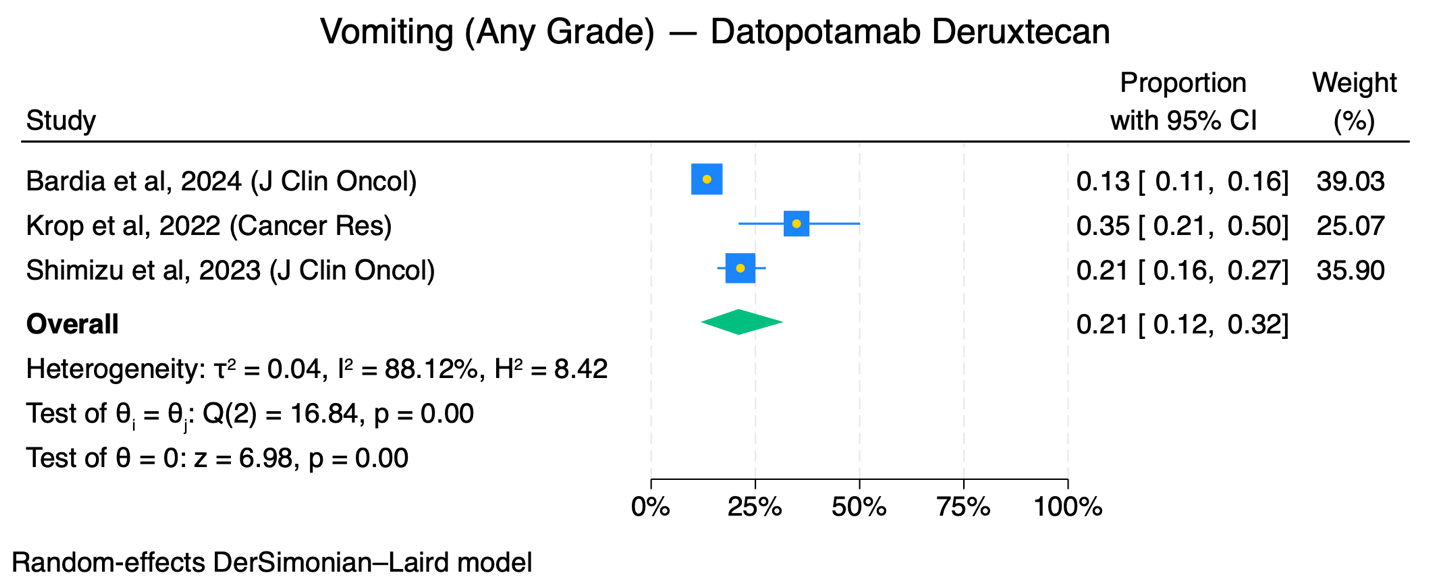


**7.4**


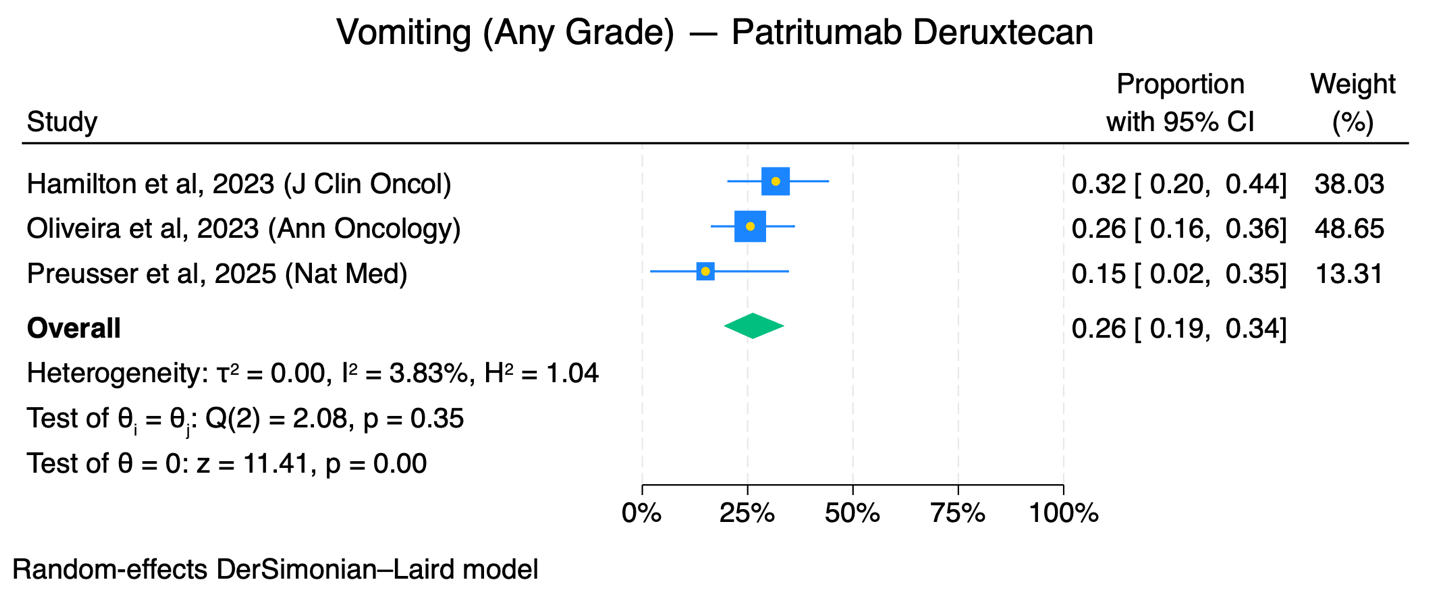


**7.5**


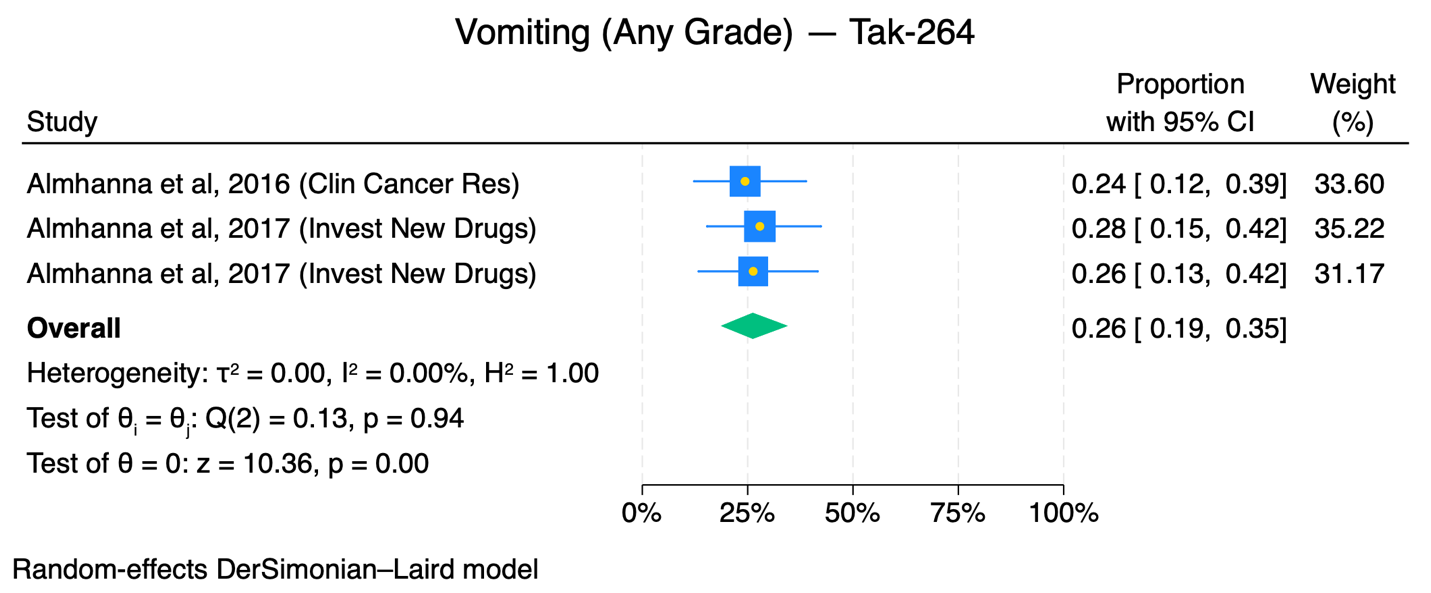


**7.6**


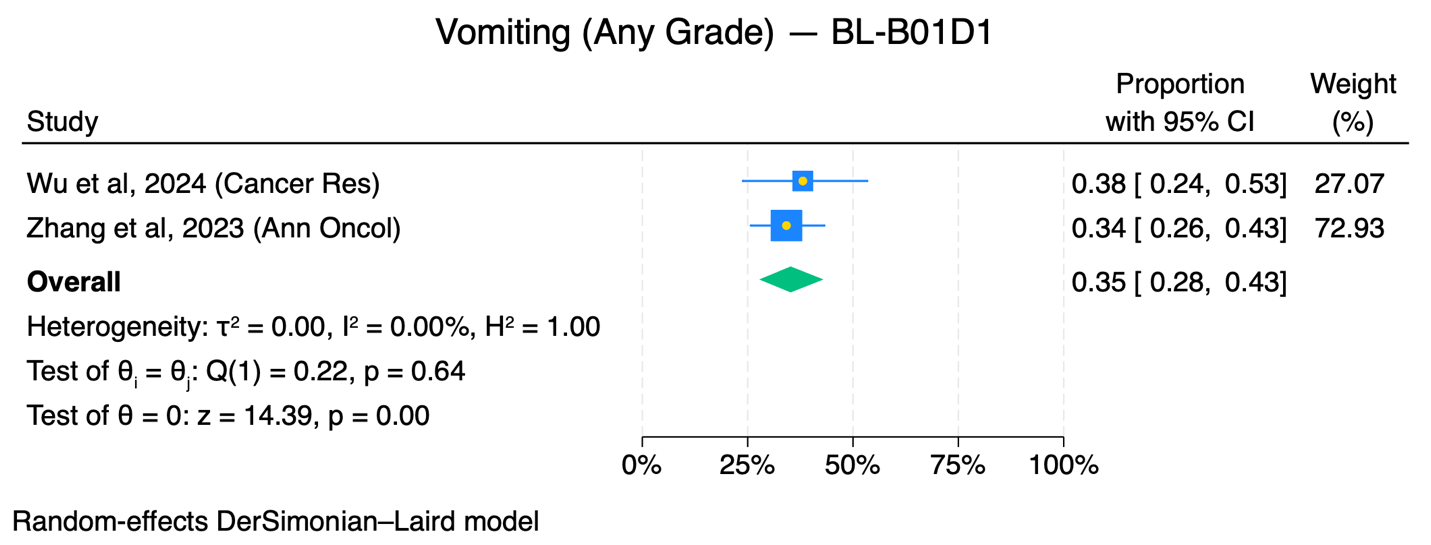


**7.7**
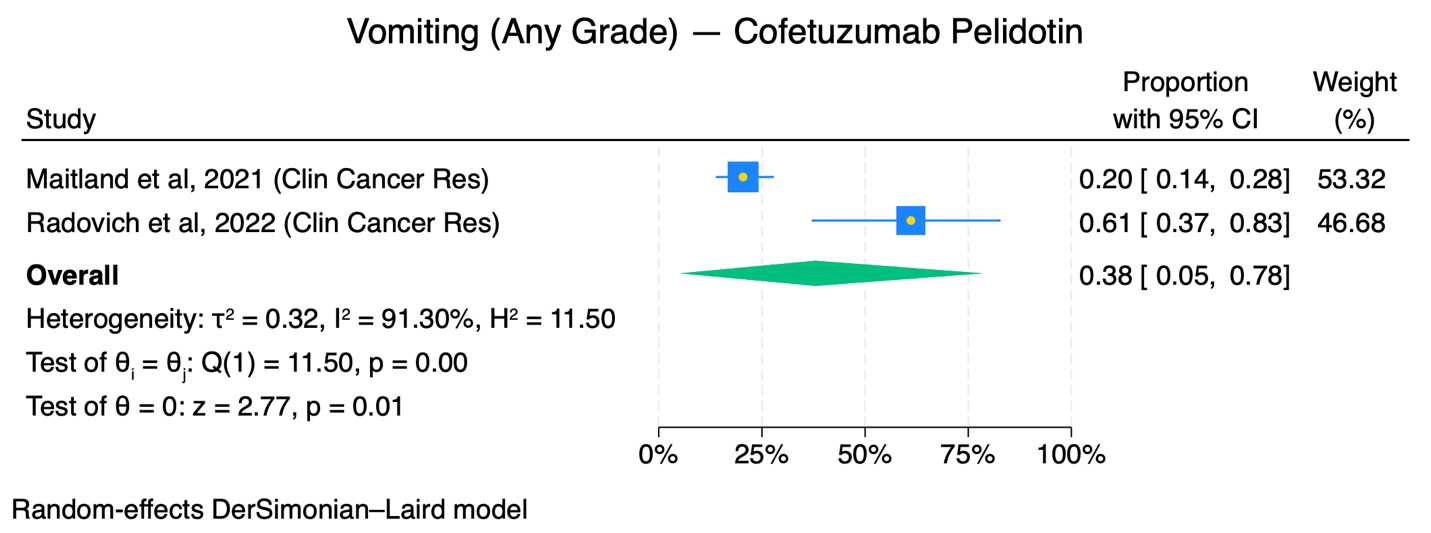


**7.8**


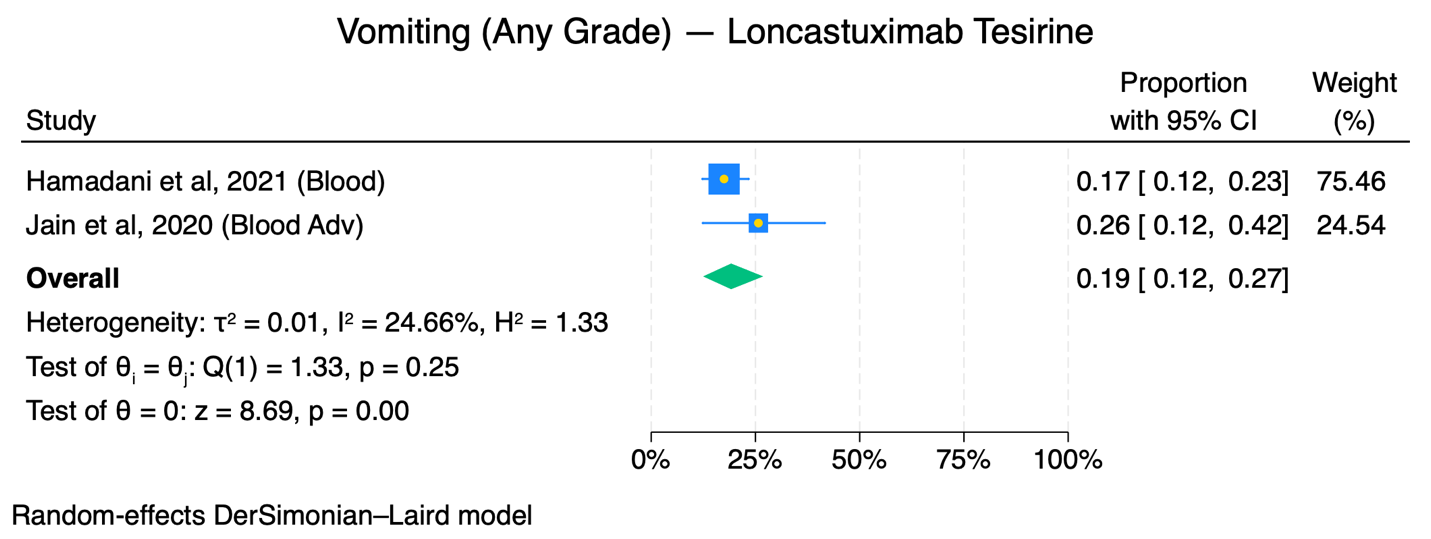


**7.9**
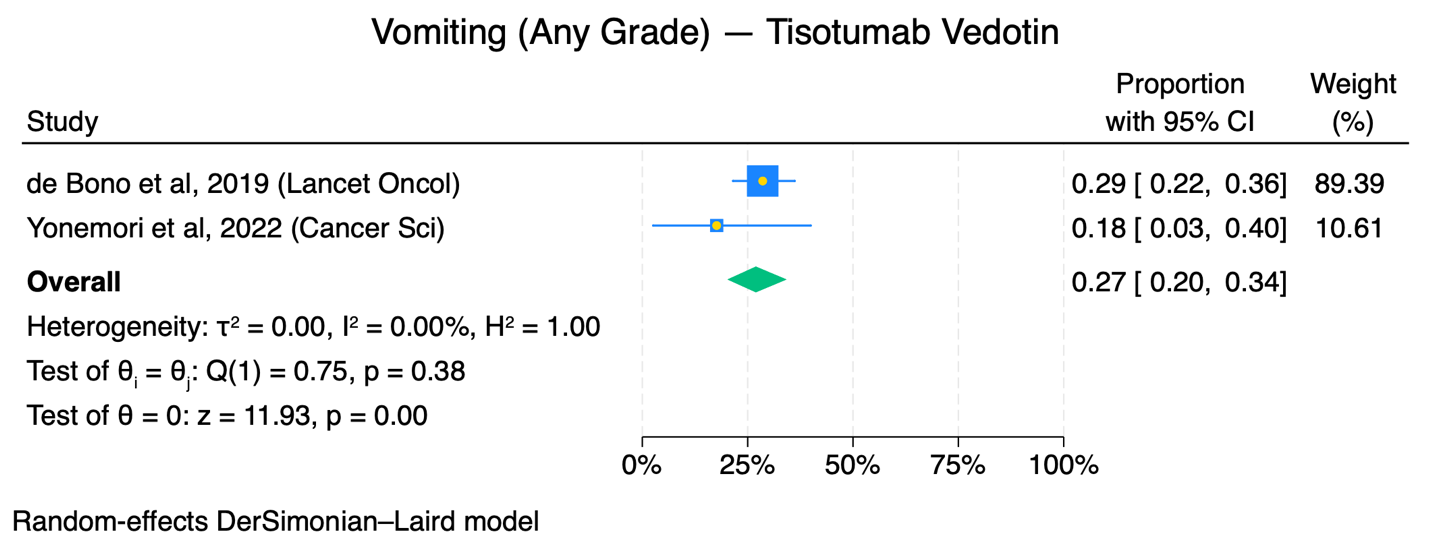


**Appendix 8.** Prevalence of Vomiting by **8.1** Primary Cancer **8.2** Average Age of Patients (p = 0.410) **8.3** Percentage Female Patients (p = 0.957) **8.4** Number of Patients in Study (p = 0.135) **8.5** Follow-Up Duration of Study (p = 0.704)

**8.1**

**
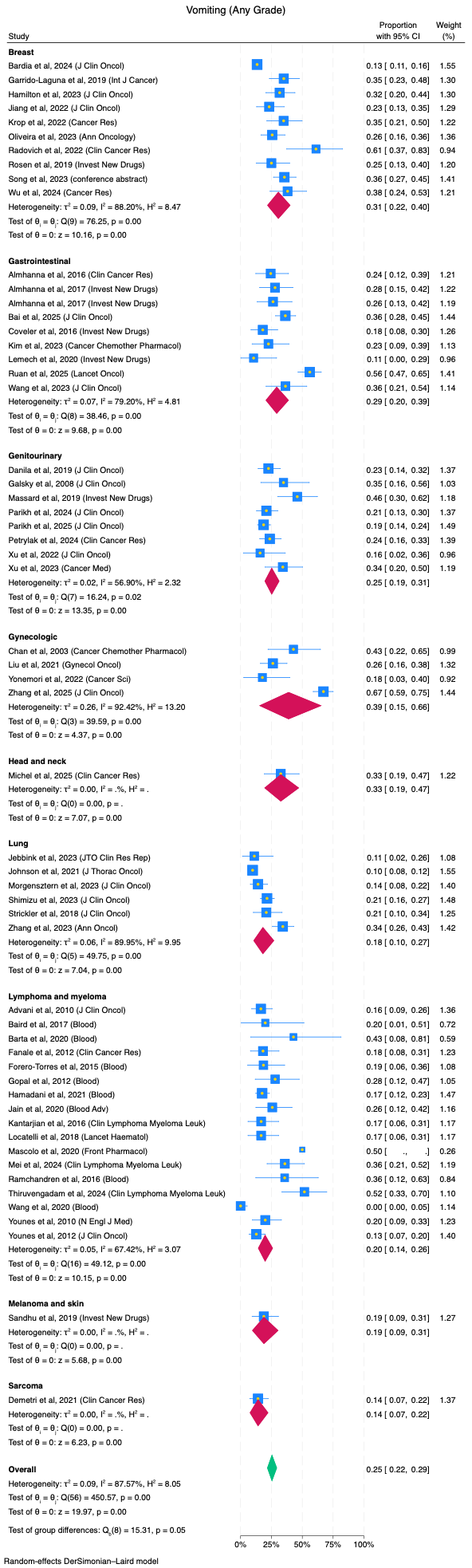
**

**8.2**

**
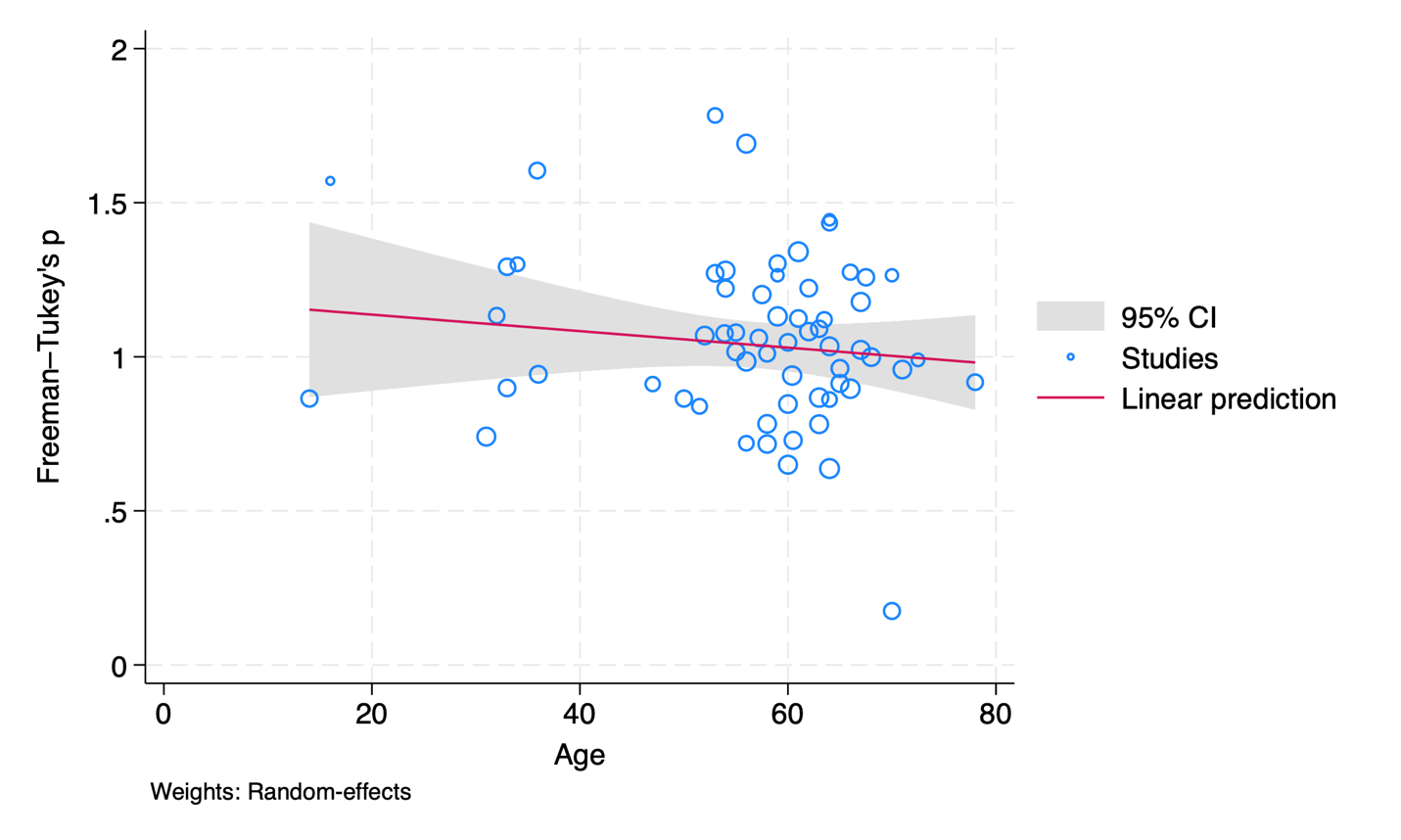
**

**8.3**

**
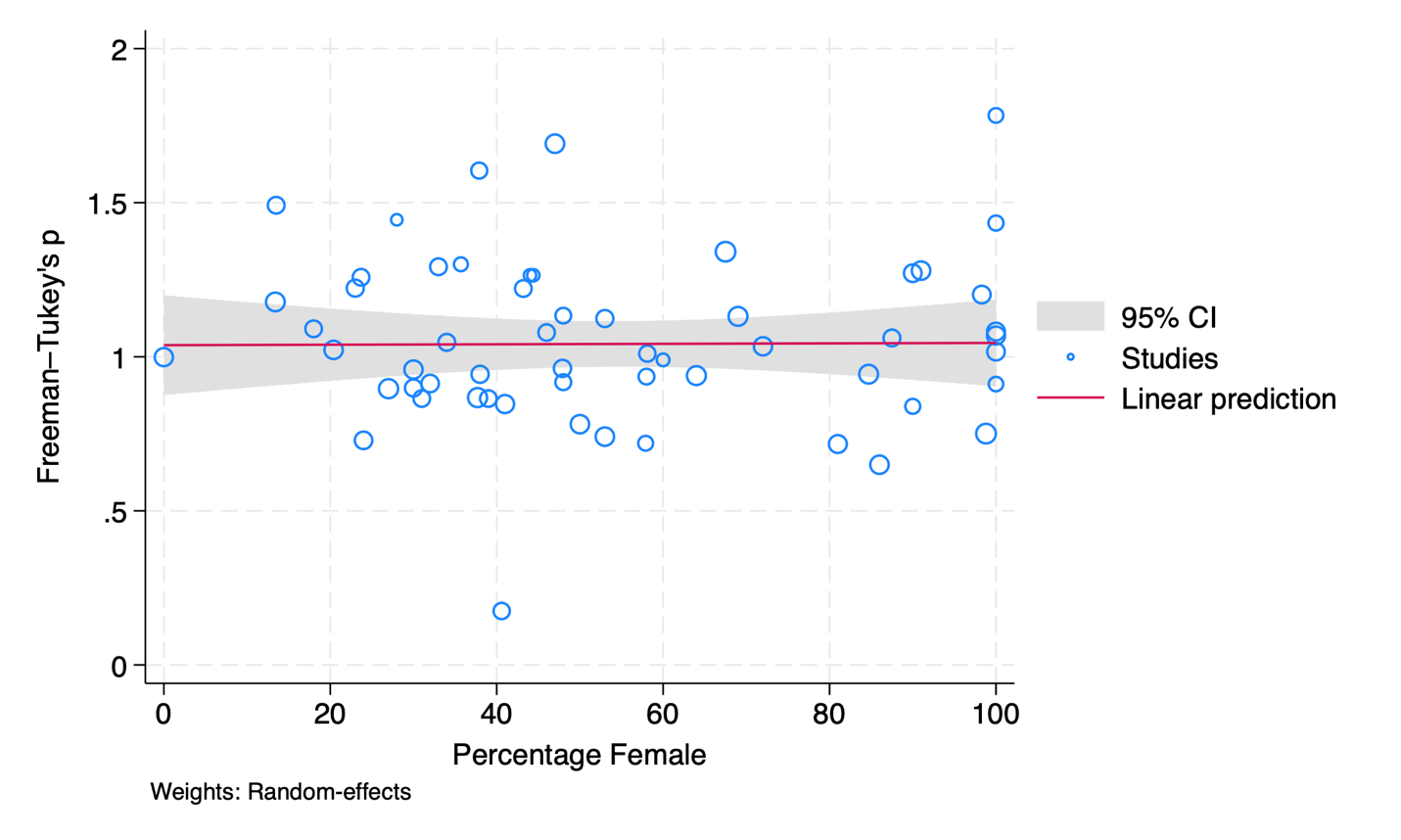
**

**8.4**

**
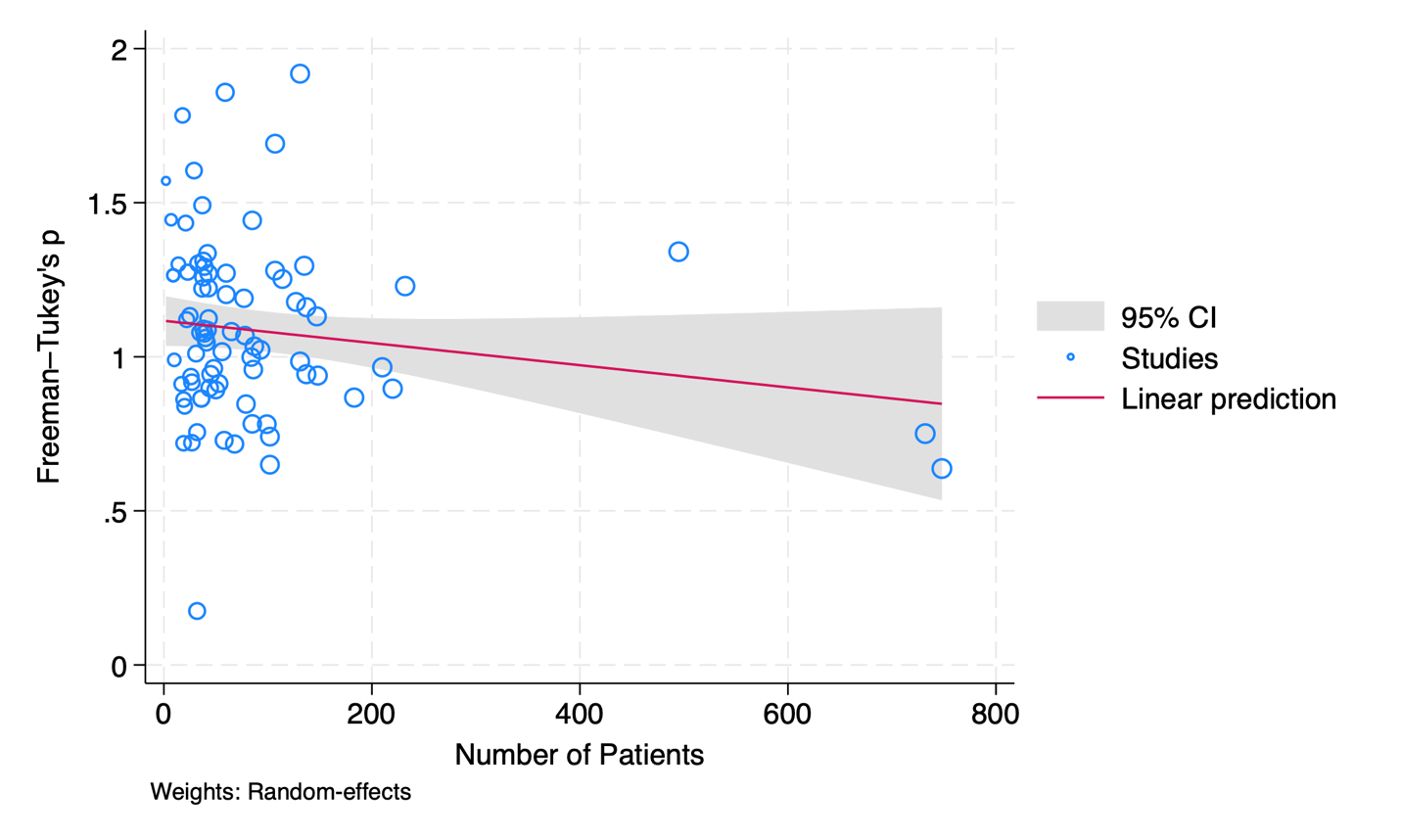
**

**8.5**

**
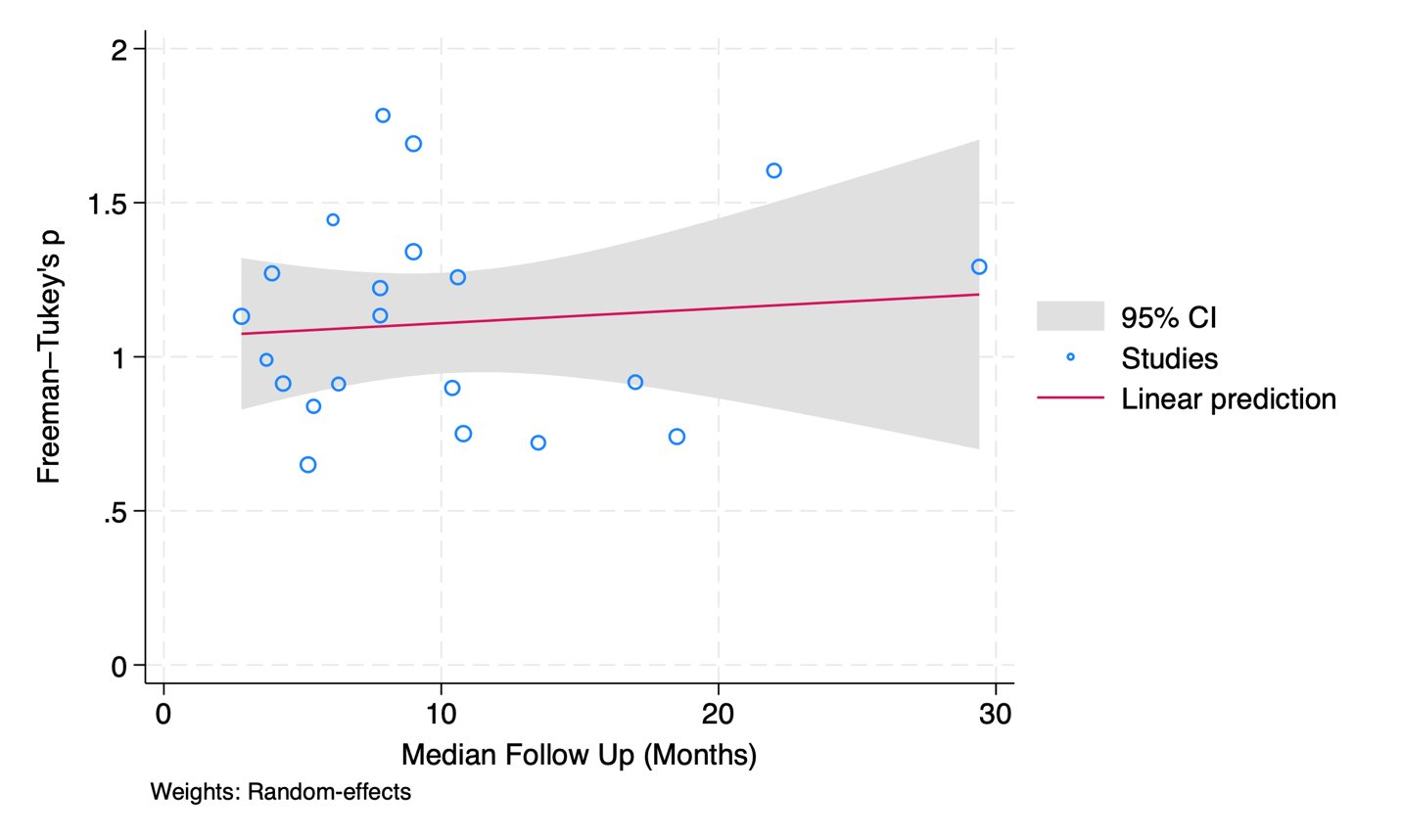
**

**Appendix 9.** Prevalence of Severe Vomiting by Antibody Drug Conjugate **9.1** Trentuximab Vedotin **9.2** BL-B01D1 **9.3** Datopotamab Deruxtecan **9.4** Tak-264 **9.5** Cofetuzumab Pelidotin **9.6** Inotuzumab Ozogamicin **9.7** Patritumab Deruxtecan **9.8** Sacituzumab Govitecan **9.9** SYS6010 **9.10** Trasutuzmab Deruxtecan

**9.1**


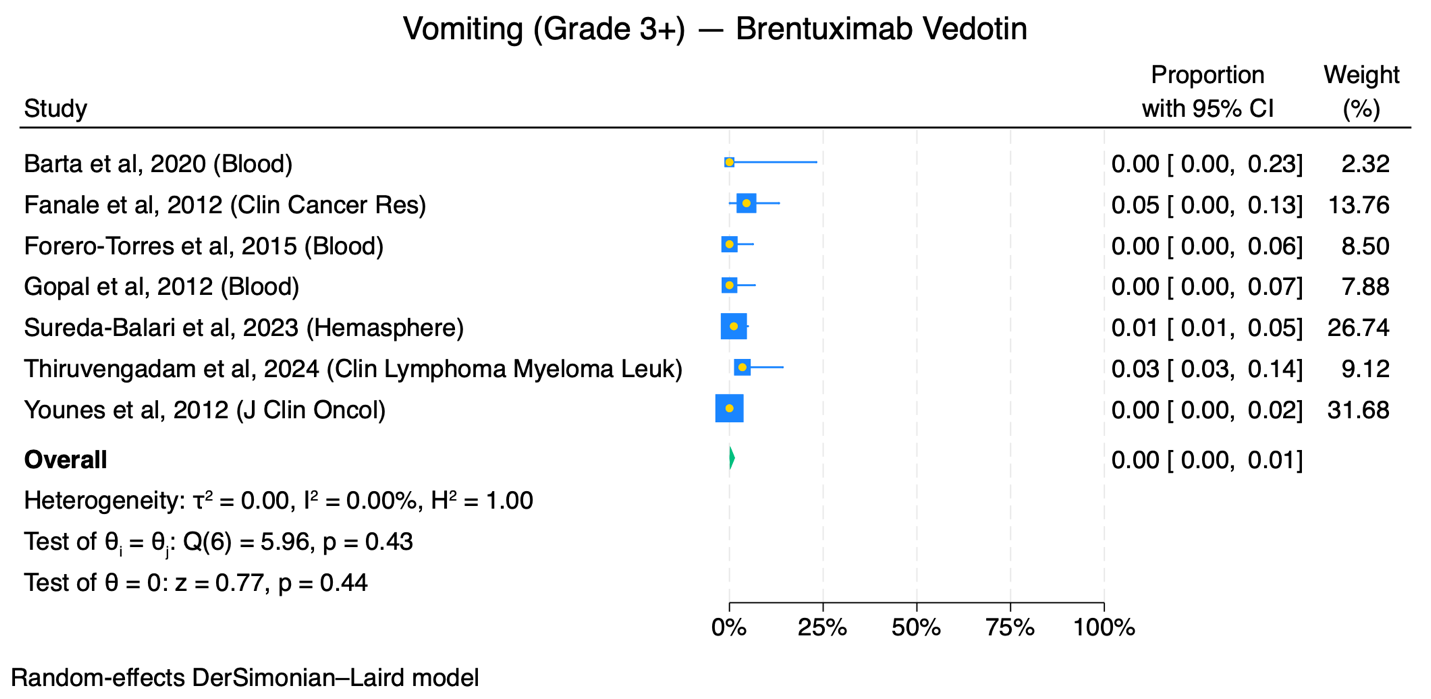


**9.2**


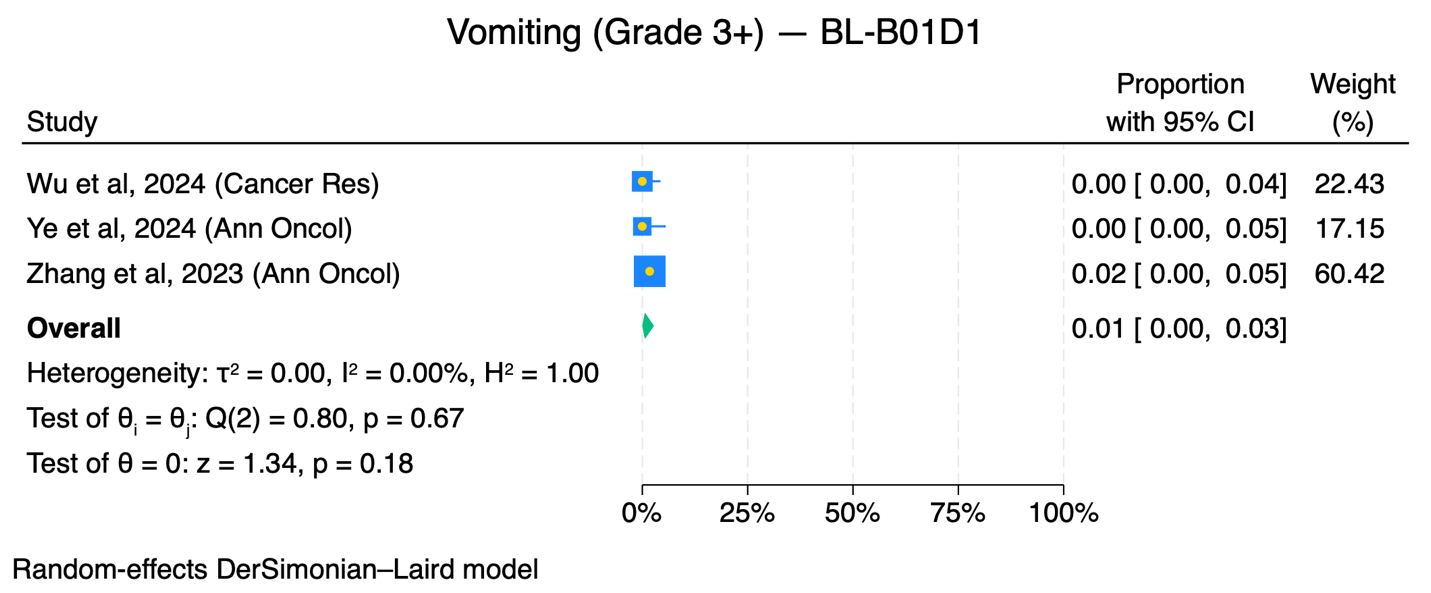


**9.3**


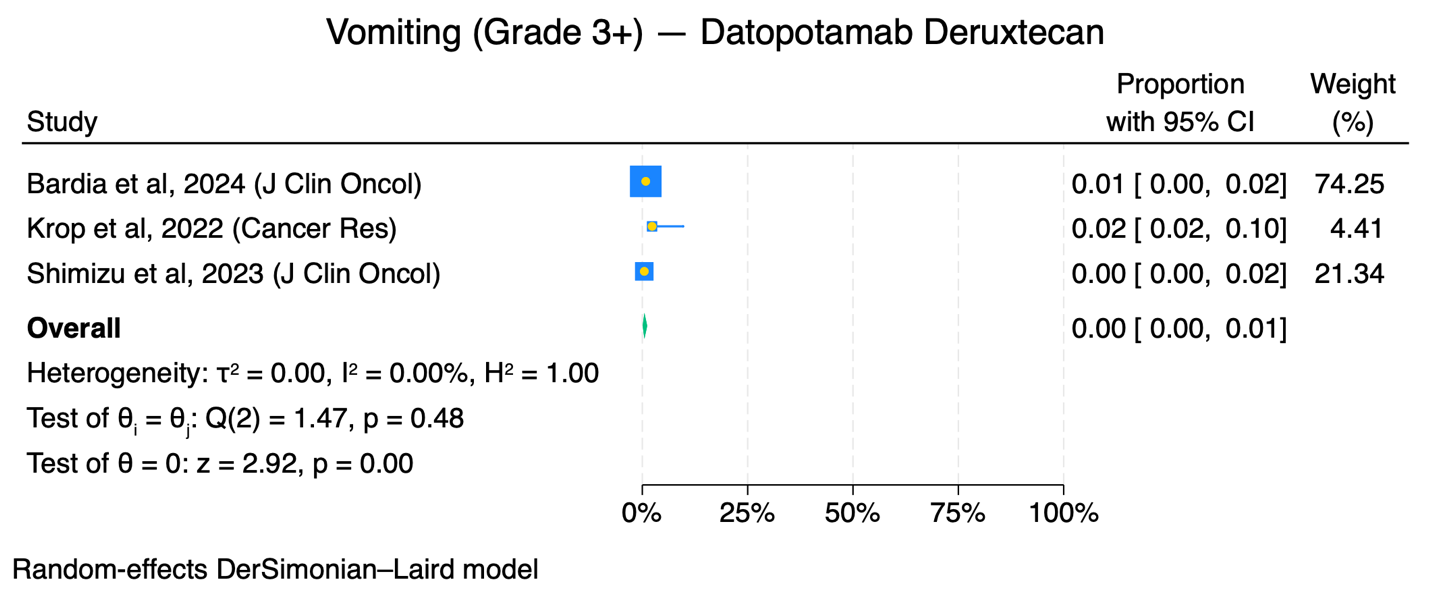


**9.4**


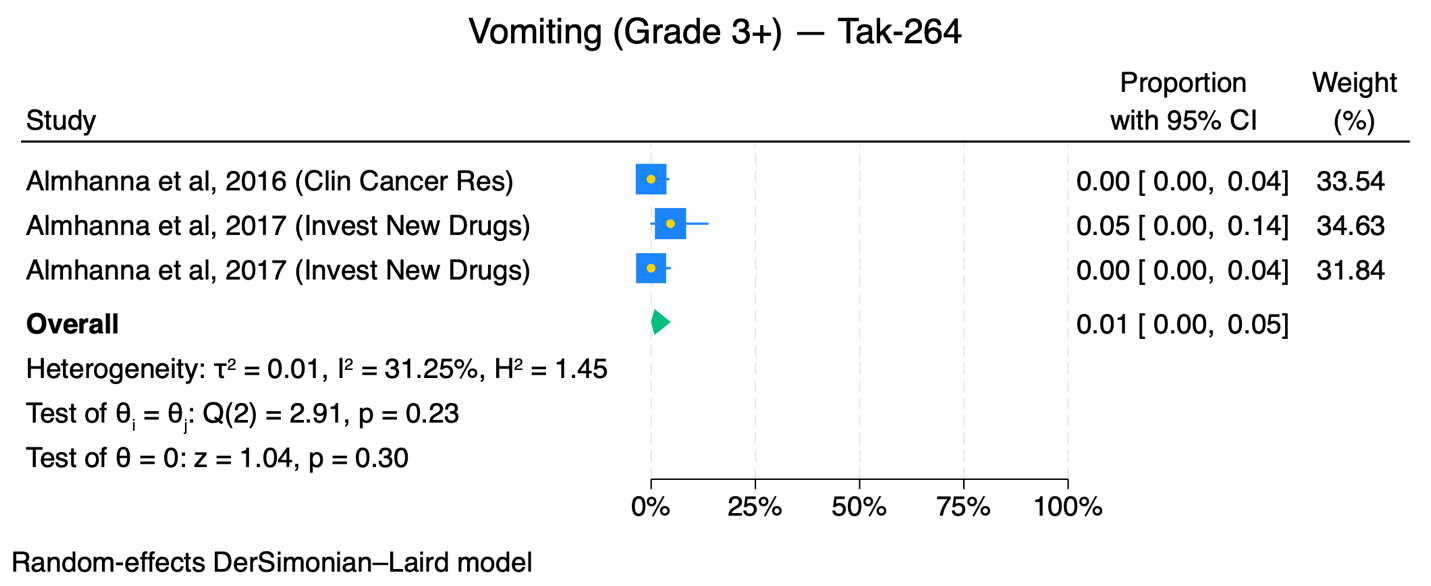


**9.5**


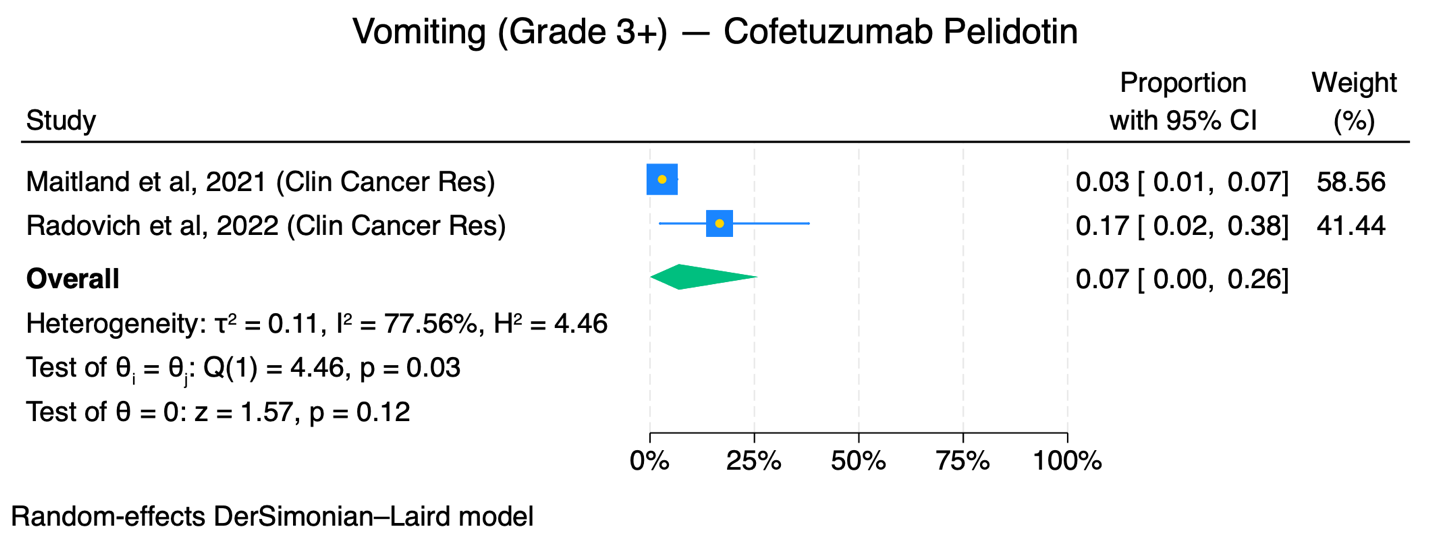


**9.6**


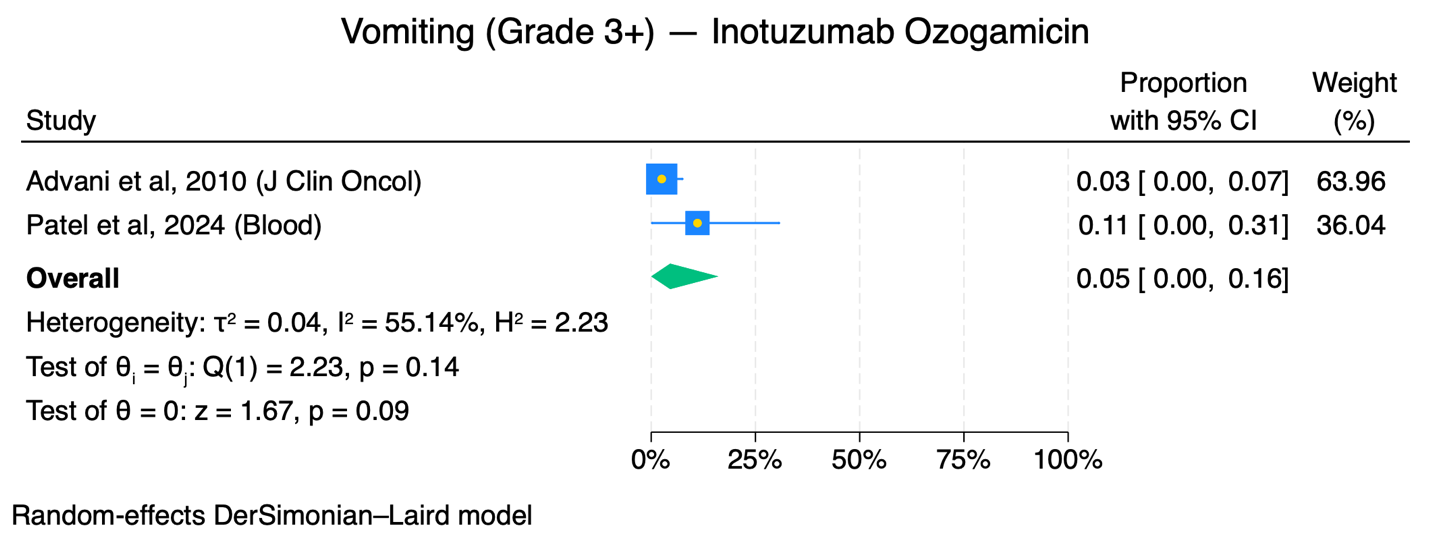


**9.7**


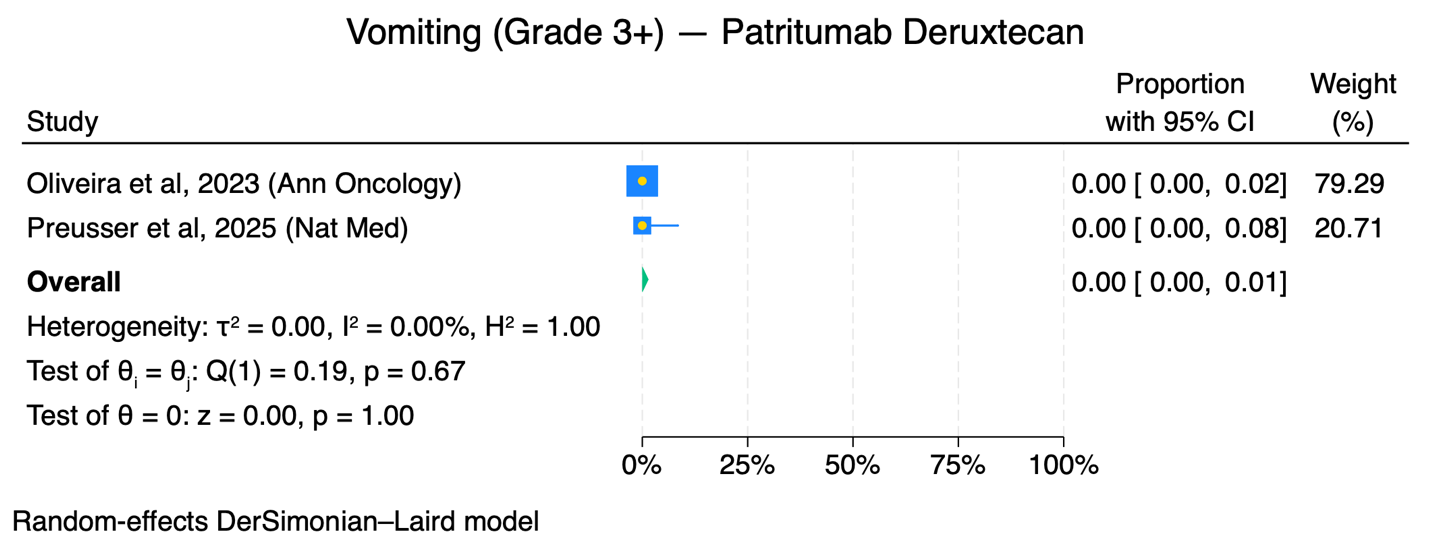


**9.8**


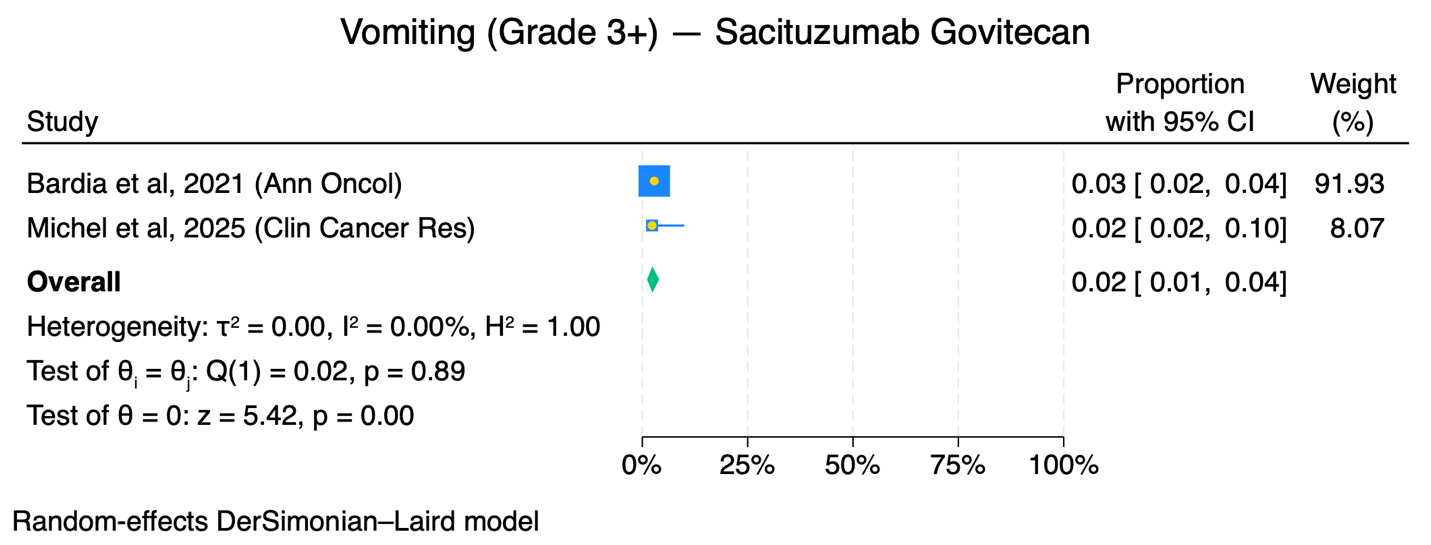


**9.9**


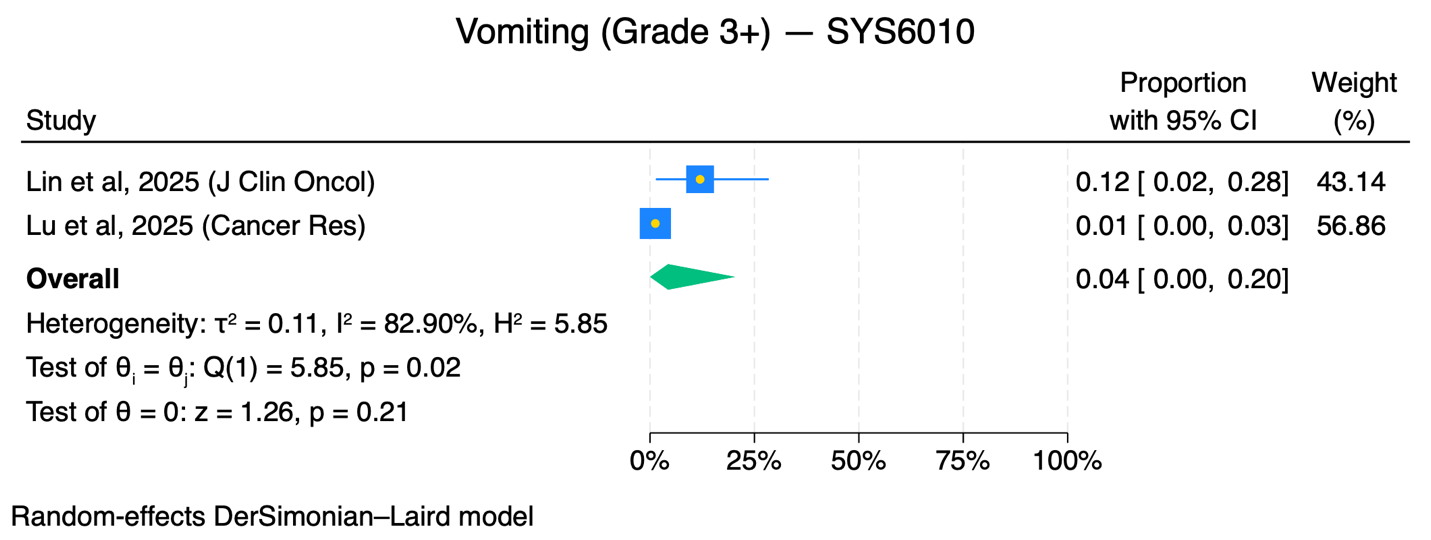


**9.10**


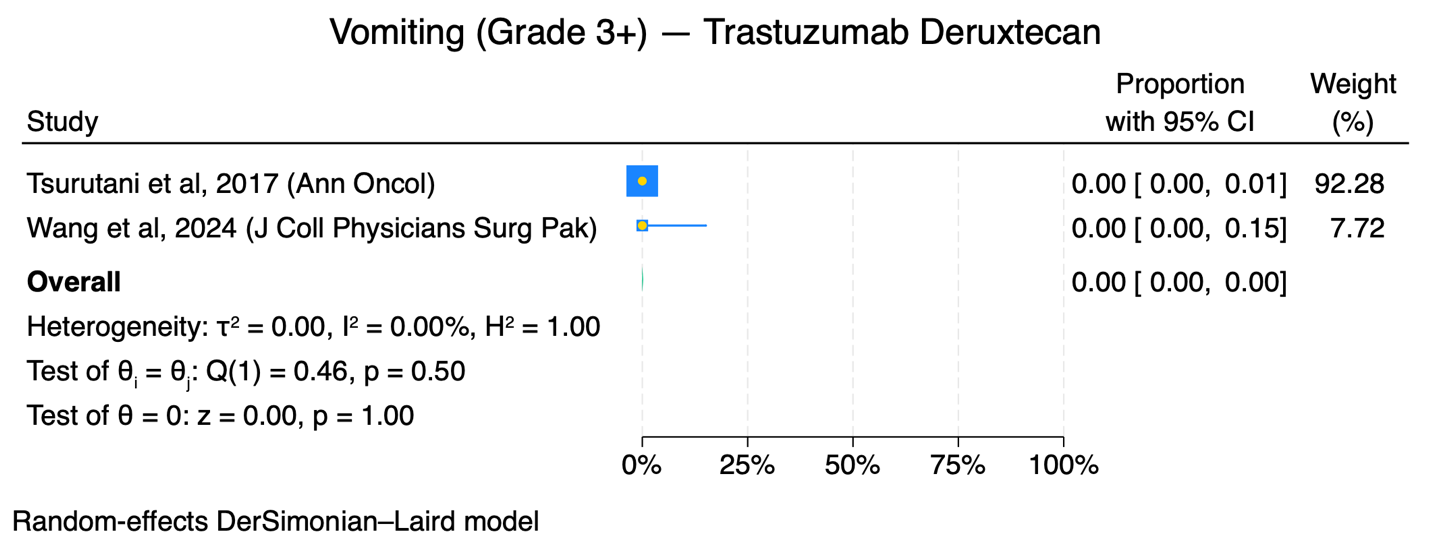


**Appendix 10.** Prevalence of Severe Vomiting by **10.1** Primary Cancer **10.2** Average Age of Patients (p = 0.556) **10.3** Percentage Female Patients (p = 0.611) **10.4** Number of Patients in Study (p = 0.051) **10.5** Follow-Up Duration of Study (p = 0.658)

**10.1**

**
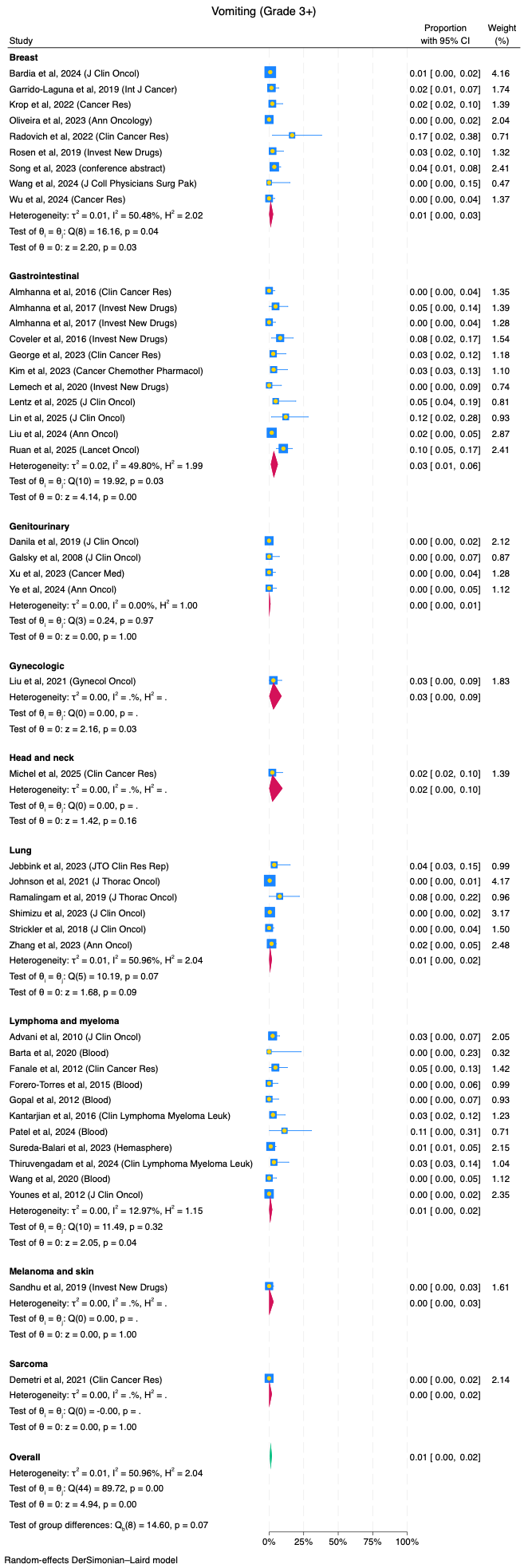
**

**10.2**

**
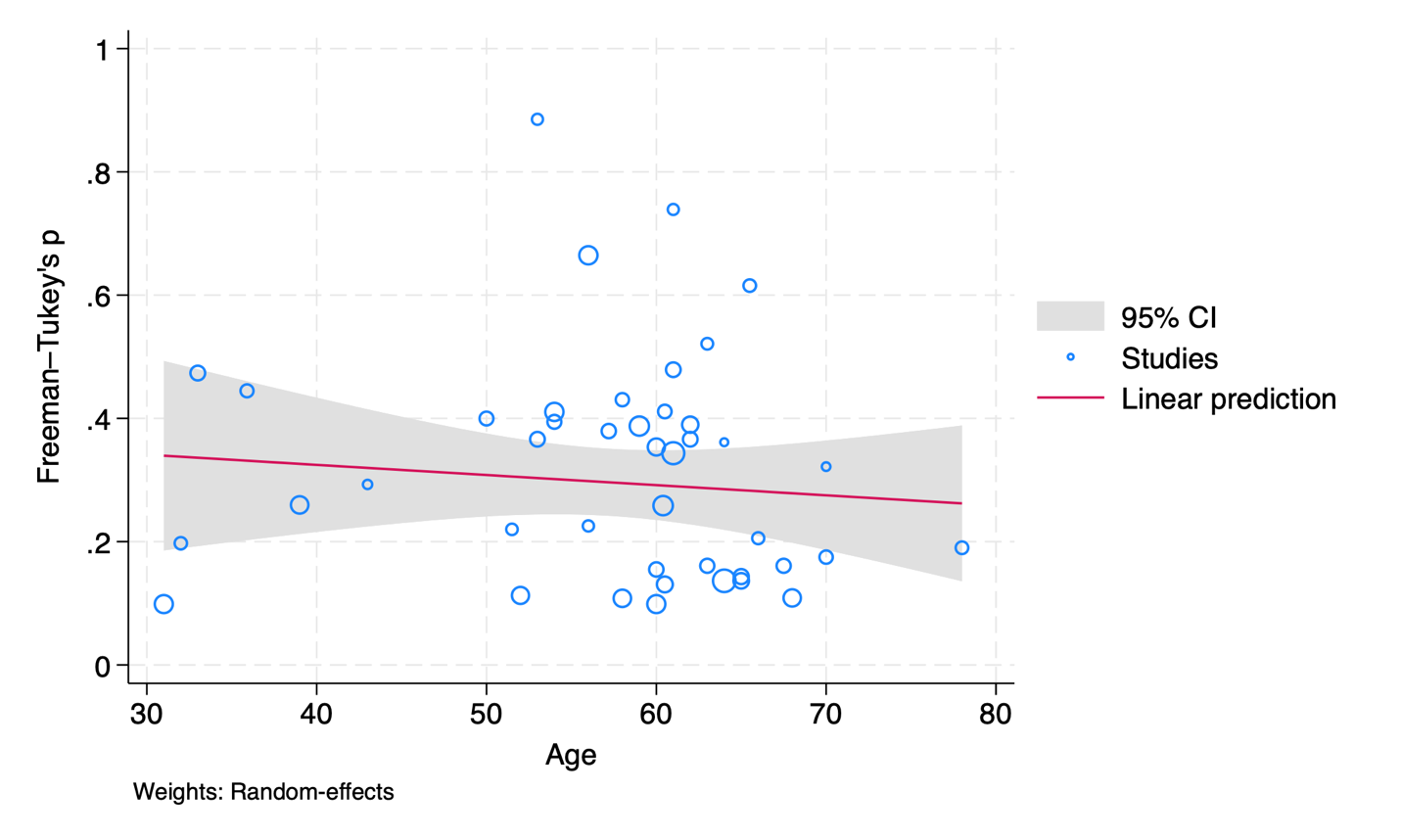
**

**10.3**

**
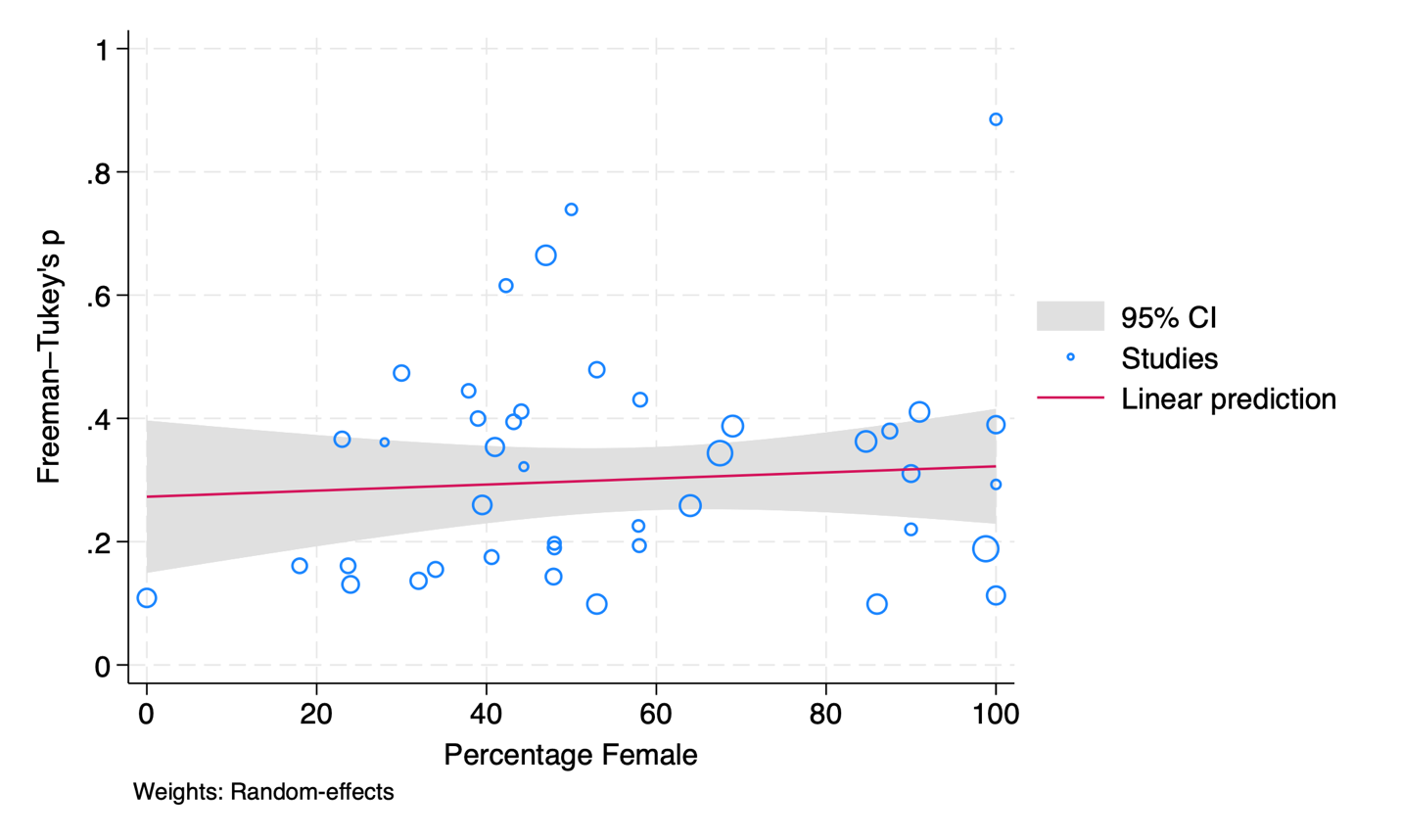
**

**10.4**

**
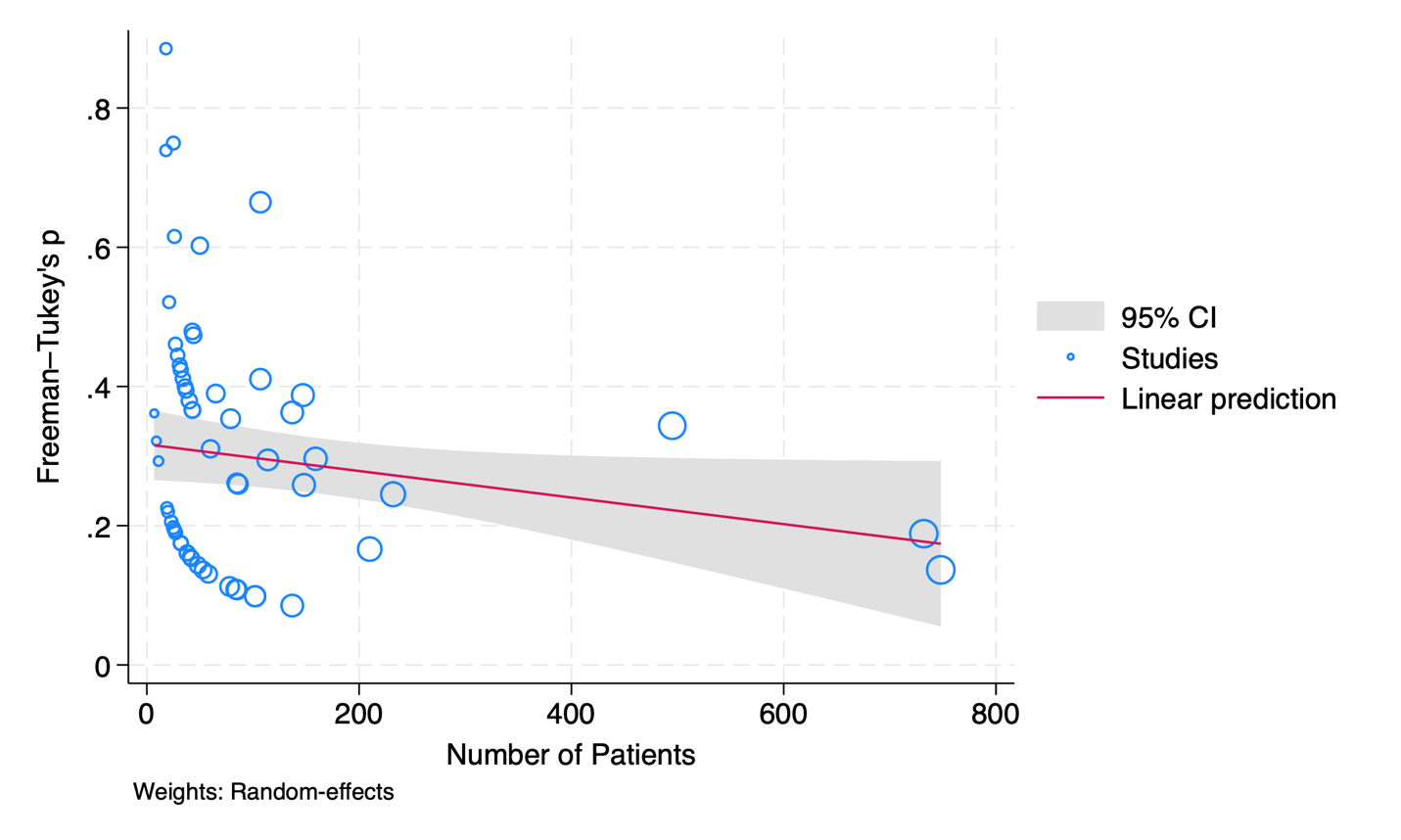
**

**10.5**

**
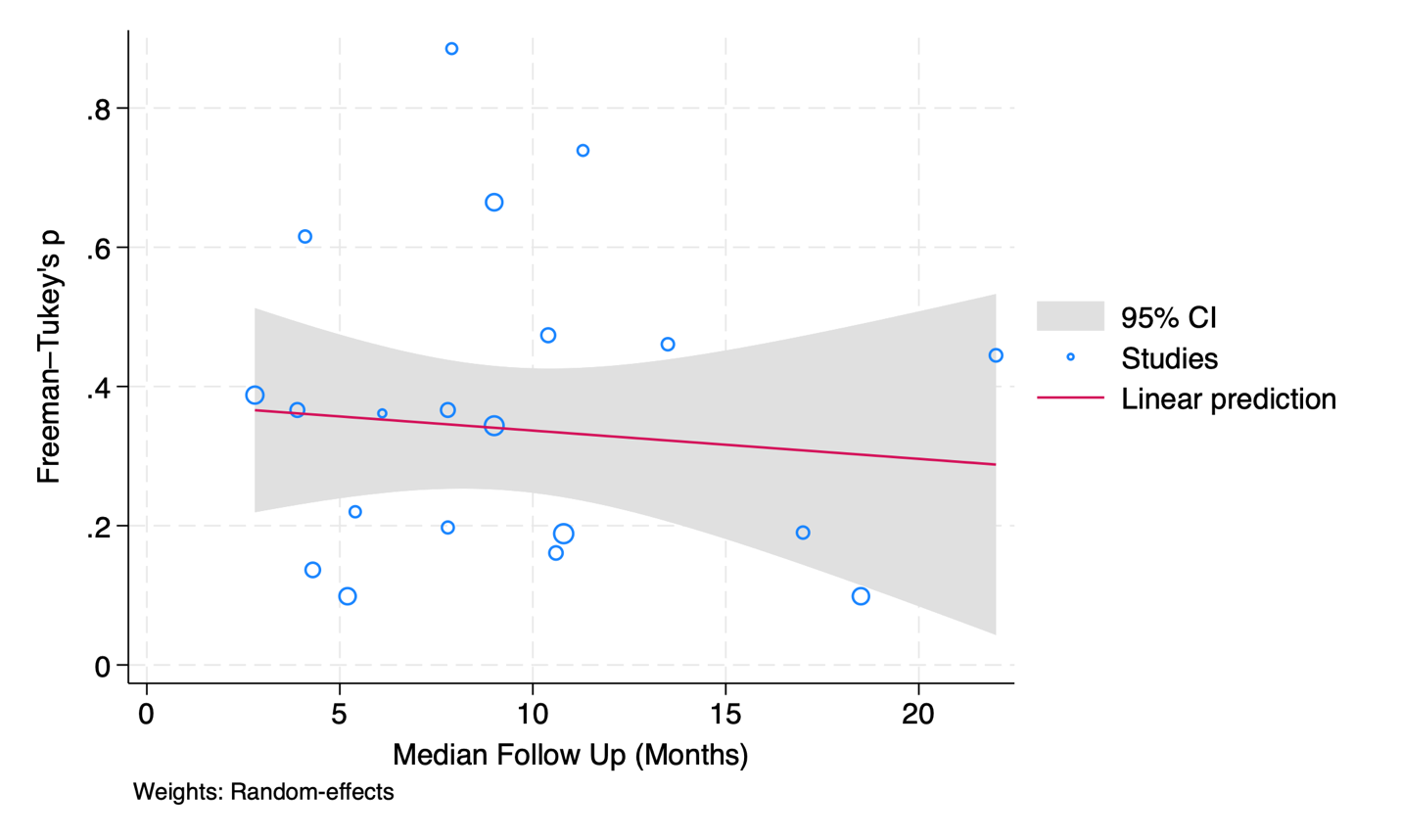
**
